# Assisted Tandem Pd Catalysis Enables Regiodivergent Heck Arylation of Transiently Generated Substituted Enol Ethers

**DOI:** 10.1021/jacsau.2c00645

**Published:** 2023-01-12

**Authors:** Thomas Duhamel, Simone Scaringi, Baptiste Leforestier, Amalia I. Poblador-Bahamonde, Clément Mazet

**Affiliations:** Department of Organic Chemistry, University of Geneva, 30 quai Ernest Ansermet, 1211 Geneva, Switzerland

**Keywords:** assisted tandem catalysis, palladium catalysis, isomerization, regiodivergent catalysis, enol ethers, Heck reaction, DFT calculations, stereoselectivity

## Abstract

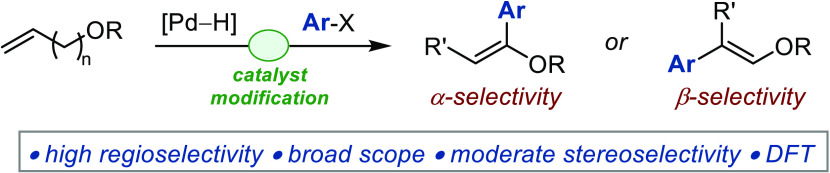

Two complementary regiodivergent Pd-catalyzed assisted tandem [isomerization/Heck arylation] reactions are reported. They provide access to a broad array of acyclic trisubstituted vinyl ethers starting from readily available alkenyl ethers. In both cases, the isomerization is conducted with a [Pd–H] precatalyst supported by tris-*tert*-butyl phosphine ligands. When the catalyst is modified by the addition of a chelating bisphosphine ligand (dppp), an organic base (Cy_2_NMe), sodium acetate, and aryl triflates are used as electrophiles, the α-arylation pathway is promoted preferentially. The β-arylation pathway is favored for electron-deficient and electron-neutral aryl halides when the catalyst is simply modified by the addition of an excess of an organic base (Et_3_N) after completion of the isomerization reaction. Electron-rich aryl halides lead to reduced levels of regiocontrol. The moderate stereoselectivity obtained are proposed to reflect the absence of stereocontrol in the isomerization step. Computational analyses suggest that migratory insertion is selectivity-determining for both the arylations. For the β-selective arylation, an energy decomposition analysis underscored that electronic factors favor α-regioselectivity and steric effects favor β-regioselectivity. Preliminary investigations show that high levels of stereoselectivity can be achieved for the α-selective arylation by ligand control. Complementarily, reaction conditions for postcatalytic stereo-correction have also been identified for each catalytic system.

## Introduction

**Figure 1:**
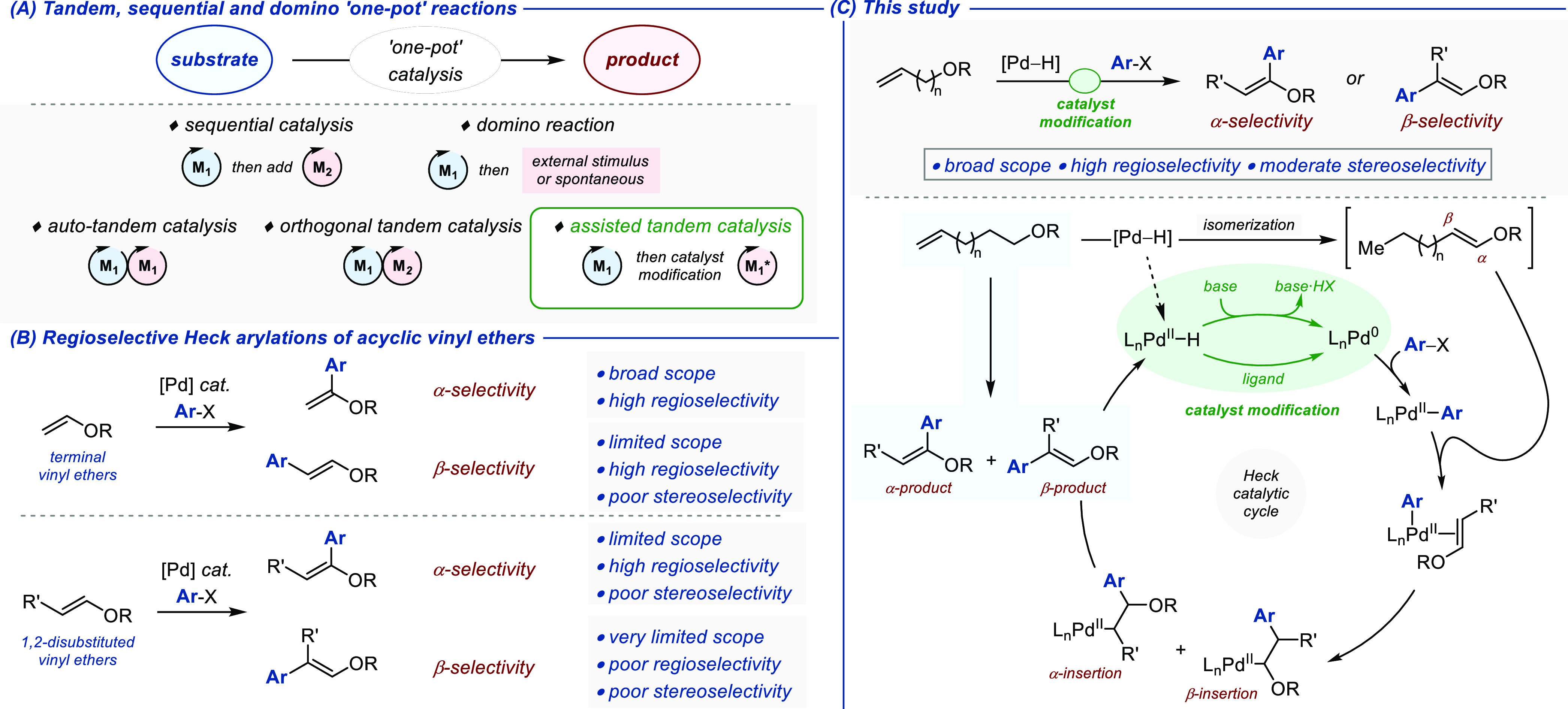
(A) Conceptual representation of one-pot sequential, domino, and tandem catalysis. (B) Pd-catalyzed Heck arylation of terminal and substituted acyclic vinyl ethers. (C) Development of assisted tandem Pd-catalyzed α- and β-arylations of transiently generated substituted vinyl ethers starting from alkenyl ethers and working mechanistic model.

The development of multistep “one-pot” catalytic transformations has gained important momentum in recent years, driven by contemporary economic and ecological imperatives (Figure 1A).^1−10^ These strategies are attractive because they lead to time- and cost-savings, step- and atom-economy, waste reduction, and overall substantial decrease in energy consumption. Moreover, they allow the handling of potentially highly reactive intermediates without isolating them and, therefore, offer access to uncharted chemical space. Whether the protocol is based on a sequential, a domino, or a tandem approach, a key requirement for its development is to identify reaction conditions that render all reactants and catalysts compatible and allow their action in a predetermined and controlled order.^6^ This is particularly true for tandem catalytic processes, which must combine two mechanistically distinct transformations in a noninterfering manner. One elegant strategy consists in associating a metal-catalyzed isomerization of alkenes to achieve functional group interconversion with a subsequent transformation judiciously designed.^7^ While examples of auto-tandem catalysis built on this approach are quite common, systems based on orthogonal tandem or assisted tandem catalysis are scarce.^8−10^

Within the portfolio of electron-rich alkenes employed in Pd-catalyzed Heck cross-couplings, cyclic enol ethers have been used routinely for the development of intermolecular enantioselective reactions, with insertion occurring exclusively at the α position.^11−25^ Because of the difficulty associated with their preparation and isolation, acyclic enol ethers have been investigated to a much lesser extent (Figure 1B).^26−46^ Moreover, current Heck arylation protocols are limited to terminal vinyl ethers.^33,34^ Although α-arylation of these substrates is well documented, for ease of purification, the cross-coupling products are often isolated as the corresponding ketones after acidic workup.^35−39^ Highly regioselective β-arylations are less common, often poorly stereoselective, and limited in scope. They either require installation of a directing group or selective hydrolysis of mixtures of α- and β-arylation products.^40−42^ In addition, the nature of the electrophilic coupling partner (aryl triflates vs aryl halides) and its electronic properties (e-rich vs e-neutral vs e-deficient) strongly influence the regioselective outcome of the catalytic transformation.^43−48^ Importantly, catalytic arylations of 1,2-disubstituted vinyl ethers in a perfectly regio- and stereoselective manner still present a considerable challenge to date.

We have recently initiated a program toward the development of one-pot processes elaborated around (long-range) alkene isomerization reactions.^49−52^ In this context, because they are notoriously difficult to synthesize and isolate, acyclic enol ethers constitute perfect candidates for in situ generation and use in tandem catalysis.^49,53,54^ Examples of auto-tandem Co-catalyzed isomerization/polymerization and Rh-catalyzed isomerization/Claisen rearrangement/hydroacylation built on this approach have been reported.^8,55,56^ At the outset of our study, we hypothesized that a Pd-catalyzed assisted tandem isomerization/regiodivergent Heck cross-coupling process could be developed by capitalizing on the [Pd–H] intermediate, which is common to the mechanism of each catalytic reaction taken individually (Figure 1C).^57−60^ Our primary objectives were (i) to simplify access to acyclic internal enol ethers by their in situ generation from readily available alkenyl ethers and (ii) to identify conditions for their regioselective α- and β-arylation. We anticipated that implementation of this approach would require identification of reaction conditions compatible with each catalytic step, as well as catalysts or ligands capable of dictating the regioselectivity of insertion of the putative [Pd–Ar] intermediate across the C=C bond without narrowing the scope of applications. Herein, we report the realization of our objectives with the development of two complementary highly regioselective Pd-catalyzed assisted tandem protocols that provide access to a broad array of trisubstituted vinyl ethers starting from readily available alkenyl ethers. Moreover, theoretical calculations have been used to glean insights into the factors responsible for the high levels of selectivity obtained for both systems. Finally, we also disclose our preliminary efforts in achieving high levels of *E*/*Z* stereoselectivity by either a ligand-controlled approach or a postcatalytic stereo-correction.

## Results and Discussion

### Regiodivergent Heck Arylation of 1,2-Disusbtituted Vinyl Ethers

Given the paucity of literature precedents, our investigations were initially focused on the development of Pd-catalyzed regiodivergent Heck arylations of 1,2-disubstituted vinyl ethers using (*Z*)-**1a** as model substrate and electron-neutral aryl triflate **2a** and aryl bromide **3a** (Table 1). Using prototypical precatalysts and reaction conditions, no product formation was observed in the coupling reaction between (*Z*)-**1a** and **2a** (entries 1–3). Next, a series of chelating bidentate phosphine ligands were evaluated. In anticipation of their subsequent implementation in a tandem process, candidates that had been used in Pd-catalyzed isomerization processes were selected.^49,50,61−65^ Although dipp and dcpe did not prove competent as ligand (entries 4–5), dppe and *rac*-Binap afforded the α-arylation product **4aa** in 18 and 29% conversion, respectively. No traces of the β-arylation product **5aa** were observed (*rr*_α/β_ > 20:1), and promising levels of stereocontrol were achieved (*E*/*Z* = 74:26 and 86:14, respectively) (entries 6–7). Using dppp as ligand led to a significant reactivity improvement without compromising the perfect α-regioselectivity but with a much reduced stereoselectivity (**4aa**: 90% conv., *rr*_α/β_ > 20:1, *E*/*Z* = 52:48) (entry 8). At the end of this experiment, **1a** was recovered as a 40:60 *E*/*Z* stereoisomeric mixture. Of important note, a similar result was obtained starting from a stereoisomeric mixture of vinyl ether **1a** (entry 9), thus suggesting that a rapid *E*/*Z* isomerization precedes the C–C bond-forming process. In our attempts to generate preferentially the β-regioisomer, we found that a nearly 1:1 mixture of regioisomers **4aa**/**5aa** was generated using aryl bromide **3a** and a catalytic combination of [Pd_2_(dba)_3_/P*t*Bu_3_] in dioxane at 50 °C (entry 10). Under similar reaction conditions, the reactivity and β-regioselectivity could be improved with the electron-deficient aryl bromide **3b**, producing **5ab** as a major regioisomer (*rr*_α/β_ 1:3.2) albeit as a 50:50 mixture of stereoisomers (entry 11). While the use of the sterically more demanding PAd_3_ ligand did not prove beneficial to the system, employing an excess of base had a positive impact on both the β-regioselectivity and the stereoselectivity of the reaction (entries 12–15). Finally, using the commercially available precatalyst [Pd(P*t*Bu_3_)_2_] and a large excess of Et_3_N (36 equiv) in *tert*-butyl methyl ether (TBME) (1:1 v/v) afforded **5ab** in 91% conversion and excellent stereoselectivity (*E*/*Z* = 4:96; entry 16). Of note, vinyl ether **1a** was recovered without any noticeable isomerization (*E*/*Z* > 5:95). When this reaction was repeated with a stereoisomeric mixture of **1a**, the cross-coupling product **5ab** was generated as a 43:57 *E*/*Z* mixture (entry 17). Therefore, the β-selective process is stereospecific and isomerization of the vinyl ether is likely prevented by use of an excess of the organic base.^66^

**Table 1:**
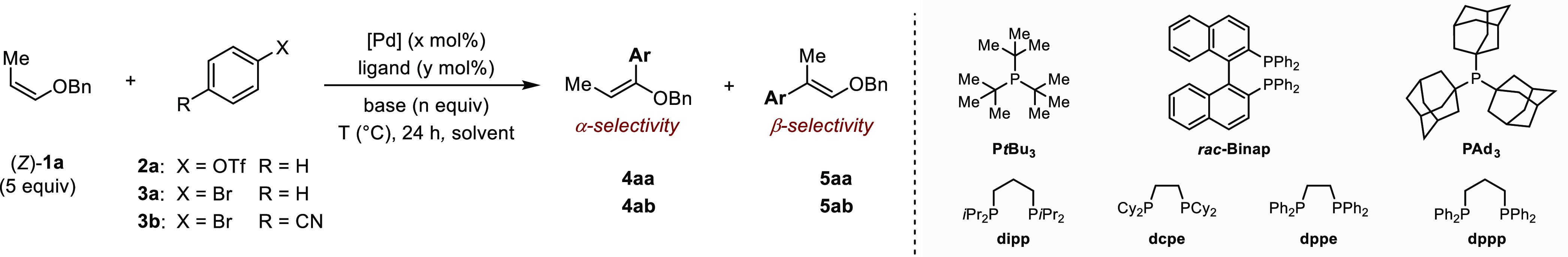
Regiodivergent Heck Arylation of 1,2-Disubstituted Vinyl Ethers: Reaction Optimization^a^

entry	Ar-X	Pd source (*x*)	ligand (*y*)	base (*n*)	solvent	*T* (°C)	conv. (%)^b^	*E*/*Z*^b^	*rr*_α/β_^b^
1	**2a**	Pd(OAc)_2_ (5)		Cy_2_NMe (1)	2-MeTHF	100	<5	nd	nd
2	**2a**	Pd(OAc)_2_ (5)	P*t*Bu_3_ (10)	Cy_2_NMe (1)	2-MeTHF	100	<5	nd	nd
3	**2a**	Pd(P*t*Bu_3_)_2_ (5)		Cy_2_NMe (1)	2-MeTHF	100	<5	nd	nd
4	**2a**	Pd(OAc)_2_ (5)	dipp (10)	Cy_2_NMe (1)	2-MeTHF	100	<5	nd	nd
5	**2a**	Pd(OAc)_2_ (5)	dcpe (10)	Cy_2_NMe (1)	2-MeTHF	100	<5	nd	nd
6	**2a**	Pd(OAc)_2_ (5)	dppe (10)	Cy_2_NMe (1)	2-MeTHF	100	18	74:26	>20:1
7	**2a**	Pd(OAc)_2_ (5)	*rac*-Binap (10)	Cy_2_NMe (1)	2-MeTHF	100	29	86:14	>20:1
8	**2a**	Pd(OAc)_2_ (5)	dppp (10)	Cy_2_NMe (1)	2-MeTHF	100	90	52:48	>20:1
9^c^	**2a**	Pd(OAc)_2_ (5)	dppp (10)	Cy_2_NMe (1)	2-MeTHF	100	87	48:52	>20:1
10	**3a**	Pd_2_(dba)_3_ (2.5)	P*t*Bu_3_ (10)	Cy_2_NMe (1)	dioxane	50	54	50:50	1:1
11	**3b**	Pd_2_(dba)_3_ (2.5)	P*t*Bu_3_ (10)	Cy_2_NMe (1)	dioxane	50	88	50:50	1:3.2
12	**3b**	Pd_2_(dba)_3_ (2.5)	PAd_3_ (10)	Cy_2_NMe (1)	dioxane	50	63	50:50	1:3.8
13	**3b**	Pd(P*t*Bu_3_)_2_ (5)		Cy_2_NMe (7)	dioxane	50	75	25:75	1:4.8
14	**3b**	Pd(P*t*Bu_3_)_2_ (5)		Et_3_N (7)	dioxane	50	74	12:88	1:4.7
15	**3b**	Pd(P*t*Bu_3_)_2_ (5)		Et_3_N (36)	dioxane	50	91	4:96	1:5.9
16	**3b**	Pd(P*t*Bu_3_)_2_ (5)		Et_3_N (36)	TBME	50	91	4:96	1:8.1
17^c^	**3b**	Pd(P*t*Bu_3_)_2_ (5)		Et_3_N (36)	TBME	50	73	43:57	1:10.4

a Reactions performed on a 0.1 mmol scale.

b Conversion based on ArX, stereoselectivity, and regioisomeric ratio determined by ^1^H NMR spectroscopy using an internal standard.

c Using a 40:60 *E*/*Z* mixture of **1a**.

### Assisted Tandem [Isomerization/Regiodivergent Heck Arylation] of Alkenyl Ethers

The identification of [Pd(P*t*Bu_3_)_2_] as an optimal precatalyst for the β-selective Heck arylation of **1a** together with the fact that related in situ generated palladium hydrides have been used in the isomerization of various alkenes prompted us to initiate the optimization of regiodivergent tandem isomerization/Heck processes with the well-defined [(*t*Bu_3_P)_2_PdHCl] precatalyst **C1**.^58,66^ By analogy with some of the Pd(II) precatalysts developed in our group for long-range isomerizations of alkenes, complex **C2** [(dppp)PdMeCl] was also prepared and evaluated using allyl ethers **6a**–**b** as model substrates (Table 2).^49,50,61−65^ Because the limiting reagent in these experiments is the aryl halide or pseudo-halide, variation of the stoichiometry in **6a**–**b** (n equiv) affects the formal loading in Pd of the first step (*x* mol % [Pd] for the isomerization corresponds to *n*.*x* mol % [Pd] for the Heck reaction). All reactions were conducted in a single flask without isolation of the transiently generated vinyl ethers. Addition of the aryl halide or pseudo-halide, the base, and any other additive was effected once the isomerization was complete. Of note, to maximize operational simplicity, we sought to develop a system using a solvent that would be compatible with both catalytic transformations. As evidenced by control experiments (see the Supporting Information), whereas **C1** is an effective precatalyst for the quantitative isomerization of **6a** into **1a** at room temperature in less than 1 h in ethereal solvents, **C2** is not a competent precursor. Nevertheless, after isomerization of **6a** with **C1**, addition of Cy_2_NMe (1 equiv), phenyl triflate **2a** (1 equiv) and adjustment of the temperature did not lead to the formation of the expected α-arylation product **4aa** (entries 1 and 2). We reasoned that isomerization could be conducted with **C1** and that, subsequently, the monophosphine ligands could be displaced by the addition of the chelating bisphosphine dppp to generate the same active species that is responsible for activity in the α-selective Heck arylation (see entries 8 and 9, Table 1). Gratifyingly, this setting led to the formation of **4aa** in 50% conversion with perfect regioselectivity (*rr*_α/β_ > 20:1; *E*/*Z* = 50:50), thus validating the design of a catalytic assisted tandem reaction, one of our initial objectives (entry 3). The reactivity was improved starting with **6b** but decreased with a lower loading of the added (P,P) ligand (5 vs 10 mol % relative to Pd) (entries 4 and 5). Finally, a 5-fold excess of the allyl ether with respect to **2a** delivered **4ba** as a single regioisomer in 77% yield (*rr*_α/β_ > 20:1; *E*/*Z* = 48:52) (entry 6). Quite unexpectedly, no product was generated when the optimized reaction conditions were applied to the electron-deficient aryl triflate **2b** (entry 7). By comparing this protocol with the optimized experimental conditions for the α-regioselective Heck arylation of **1a** reported in entries 8 and 9 of Table 1, we hypothesized that acetate ions may play an important role in triggering reactivity. Much to our delight, repetition of the previous experiment with added NaOAc (1 equiv) afforded **4bb** in 73% yield (*rr*_α/β_ > 20:1; *E*/*Z* = 62:38) (entry 8). Development of a catalytic assisted tandem protocol to access the product of β-selective arylation **5ab** starting from allyl ether **6a** and using aryl bromide **3b** was more straightforward (entries 9 and 10). We found that **C1** could be used as a precatalyst in TBME for the quantitative isomerization of **6a** into vinyl ether **1a**. The addition of an excess of Et_3_N to convert **C1** into [Pd(P*t*Bu_3_)_2_],^66^ followed by the addition of 4-bromobenzonitrile **3b** and adjustment of the temperature to 50 °C, led to the exclusive formation of regioisomer **5ab** (*rr*_α/β_ > 1:20). When the initial alkene was used in excess relative to the aryl halide, the cross-coupling product was isolated in 70% yield after purification (*E*/*Z* = 47:53). The improved level of regioselectivity in these experiments compared to those of the isolated Heck arylation starting from vinyl ether **1a** is unclear at this stage of our investigations. When the experiments disclosed in entries 16 and 17 of Table 1 were repeated with **C1** as catalyst precursor instead of [Pd(P*t*Bu_3_)_2_], similar catalytic activities were observed, but **5ab** was generated quasi-exclusively with *rr*_α/β_ > 1:20. The low level of stereoselectivity for the [isomerization/β-arylation] assisted tandem process is likely to originate from the absence of stereocontrol by the active [Pd–H] species during the first step as confirmed by independent monitoring experiments of the isomerization reaction (see the Supporting Information).

**Table 2:**
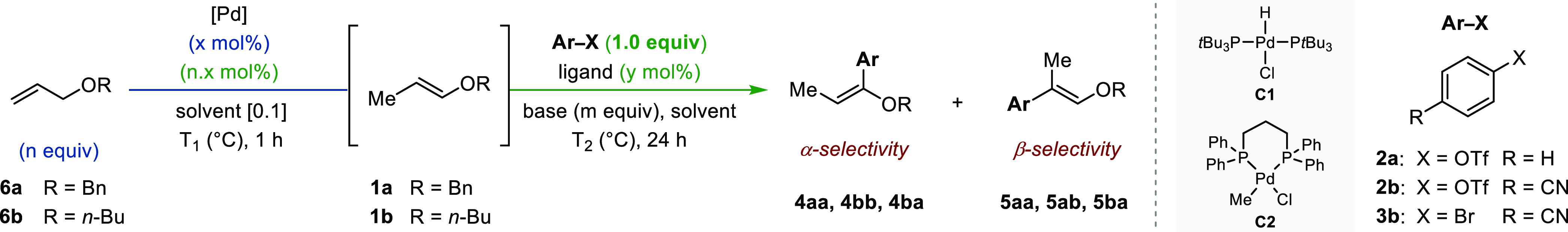
Assisted Tandem [Isomerization/Regiodivergent Heck Arylation] of Alkenyl Ethers: Reaction Optimization^a^

entry	cat. (*x*/*n*.*x*)	**6** (*n*)	solvent	*T*_1_/*T*_2_ (°C)	Ar-X	base (*m*)	ligand (*y*)	conv. (%)^b^	*E*/*Z*^b^	*rr*_α/β_^b^
1	**C1** (4.5/5.0)	**6a** (1.1)	2-MeTHF	25/100	**2a**	Cy_2_NMe (1)		<5	nd	nd
2	**C2** (4.5/5.0)	**6a** (1.1)	2-MeTHF	25/100	**2a**	Cy_2_NMe (1)		<5	nd	nd
3	**C1** (4.5/5.0)	**6a** (1.1)	2-MeTHF	25/100	**2a**	Cy_2_NMe (1)	dppp (10)	50	50:50	>20:1
4	**C1** (4.5/5.0)	**6b** (1.1)	2-MeTHF	25/100	**2a**	Cy_2_NMe (1)	dppp (10)	60	50:50	>20:1
5	**C1** (4.5/5.0)	**6b** (1.1)	2-MeTHF	25/100	**2a**	Cy_2_NMe (1)	dppp (5)	45	50:50	>20:1
6^c^	**C1** (1.0/5.0)	**6b** (5.0)	2-MeTHF	25/100	**2a**	Cy_2_NMe (1)	dppp (10)	70 (77)	48:52	>20:1
7^c^	**C1** (1.0/5.0)	**6b** (5.0)	2-MeTHF	25/100	**2b**	Cy_2_NMe (1)	dppp (10)	<5	nd	nd
8^c^,^d^	**C1** (1.0/5.0)	**6b** (5.0)	2-MeTHF	25/100	**2b**	Cy_2_NMe (1)	dppp (10)	87 (73)	62:38	>20:1
9	**C1** (4.5/5.0)	**6a** (1.1)	TBME	25/50	**3b**	Et_3_N (36)		55 (46)	45:55	>1:20
10^c^	**C1** (1.0/5.0)	**6a** (5.0)	TBME	25/50	**3b**	Et_3_N (36)		76 (70)	47:53	>1:20

a Reactions performed on a 0.1 mmol scale.

b Conversion based on ArX, stereoselectivity, and regioisomeric ratio determined by ^1^H NMR spectroscopy using an internal standard. In parentheses, yield after purification.

c 16 h instead of 1 h for the first step (isomerization).

d With 1.0 equiv of NaOAc.

**Figure 2:**
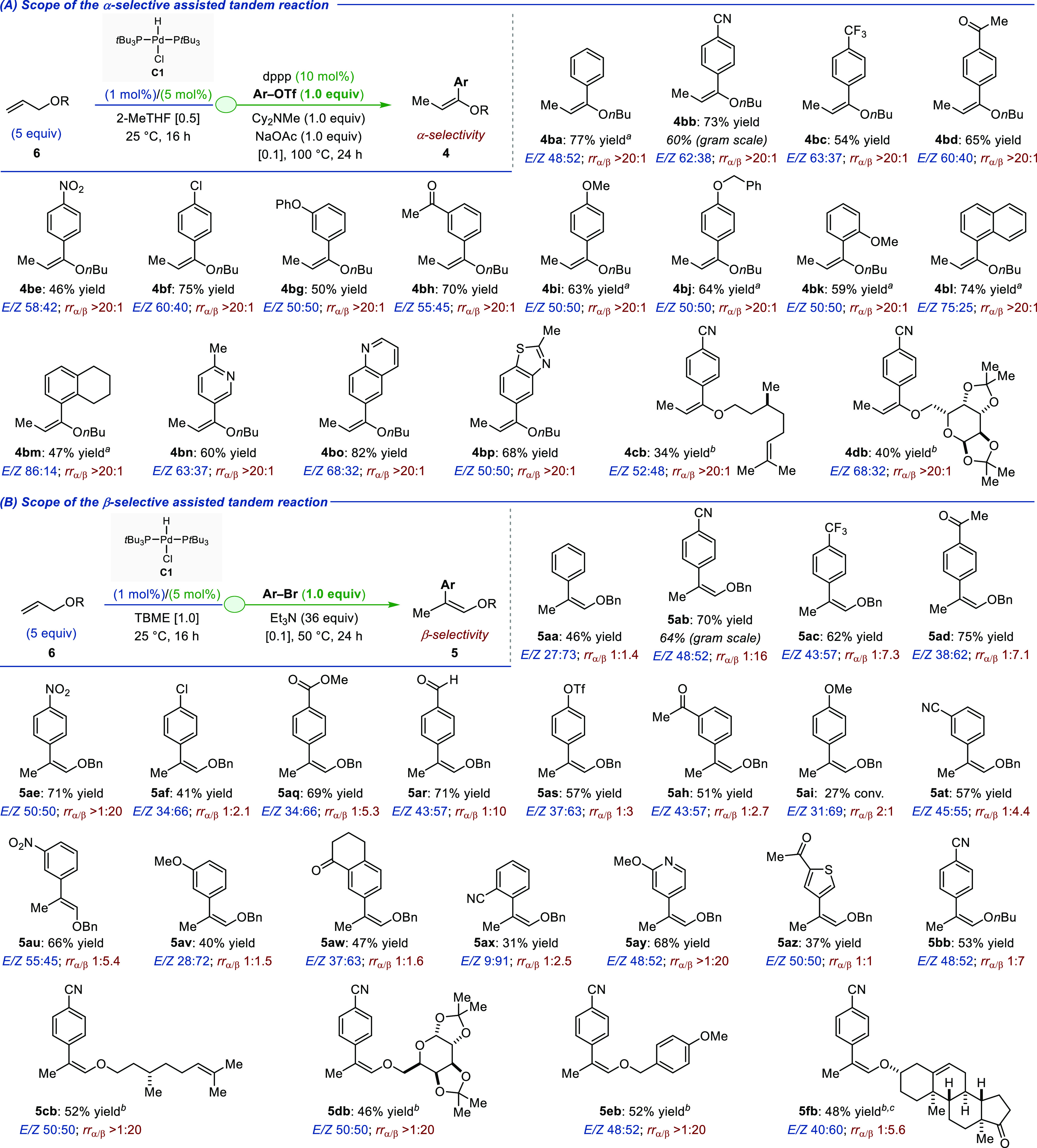
(A) Scope of the assisted tandem isomerization/α-selective Heck arylation of allyl ethers. (B) Scope of the assisted tandem isomerization/β-selective Heck arylation of allyl ethers. Regio- and stereoselectivities were measured on crude reaction mixtures by ^1^H NMR spectroscopy using an internal standard. ^*a*^ Without NaOAc. ^*b*^ Using 1.1 equiv of the appropriate allyl ether **6**. ^*c*^ Isomerization run at 50 °C for 2 h.

The generality of the catalytic α-selective assisted tandem [isomerization/Heck arylation] was investigated using the optimized reaction conditions identified in Table 2 (entries 6 and 8), and sodium acetate was used only for electron-deficient aryl triflates (Figure 2A). In all cases, exclusive formation of the α-regioisomer was observed (*rr*_α/β_ > 20:1) and the cross-coupling product was isolated in 61% average yield (18 examples; 34–82%). The *E*/*Z* stereoselectivity was moderate in most cases and varied from 48:52 to 86:14. Electron-deficient, electron-neutral, and electron-rich aryl triflates were cross-coupled with similar efficiency. All potentially sensitive substituents such as cyano (**2b**), trifluoromethyl (**2c**), (enolizable) keto (**2d**, **2h**), nitro (**2e**), chloro (**2f**), alkoxo (**2i**, **2k**), phenoxo (**2g**), and benzyloxo (**2j**) proved compatible with the catalytic process, independently of their position (*ortho*, *meta*, *para*). Heteroaryl triflates with accessible coordinating nitrogen and sulfur atoms (**2n**–**p**) as well as stereochemically complex scaffolds (**4cb**, **4db**) were also tolerated. We showed that the model [isomerization/Heck cross-coupling] tandem reaction using **6b** and **2b** could be performed on a gram scale (60% yield). Moreover, the loading in palladium could be decreased to 1 mol % with a 1:1 stoichiometry between the reactants without significantly affecting the overall catalytic performance (see the Supporting Information).

The scope of the catalytic β-selective assisted tandem [isomerization/Heck arylation] was delineated next (Figure 2B). Among the 23 combinations of cross-coupling partners evaluated, the yields varied between 31 and 75% and reflected directly the regioisomeric ratio *rr*_α/β_ (which itself varied from 2:1 to >1:20). The lack of stereocontrol in the isomerization reaction is likely responsible for the moderate *E*/*Z* ratio measured for the overall process, except when 2-bromobenzonitrile **3x** was employed (**5ax**: *E*/*Z* = 9:91).^67−69^ While electron-deficient aryl bromides (**3b**–**e**, **3q**–**s**, **3t**–**u**) and heteroaryl bromides (**3y**–**z**) appeared particularly well suited for the transformation, reduced performances were obtained with electron-neutral derivatives (**3a**, **3f**). The α-regioisomer was even produced preferentially when using the electron-rich 4-bromoanisole **3i** (**5ai**: *rr*_α/β_ 2:1). The variety of functional groups tolerated is remarkably broad and underscores the mildness and generality of the catalytic method. Indeed, bromoarenes containing a cyano (**3b**, **3t**, **3x**), a ketone (**3d**, **3h**, **3w**, **3z**), a nitro (**3e**, **3u**), an aldehyde (**3r**), and an ester (**3q**) were compatible coupling partners. Worthy of note, excellent chemoselectivity was observed with 1-bromo-4-chlorobenzene **3f** and 4-bromophenyl trifluoromethanesulfonate **3s**, thus offering the possibility to perform orthogonal cross-coupling reactions under Pd catalysis.^70−73^ Satisfactorily, when structurally more complex allylic ethers **6c**–**f** were subjected to the assisted tandem protocol, the β-regioisomer was formed preferentially and the cross-coupling product isolated in practical yield (despite the 1:1 stoichiometry used for these experiments). Finally, the standard reaction for the β-regioselective process using **6a** and **3b** was run on a gram scale without any significant reduction of the catalytic performance affording **5ab** in 64% (*rr*_α/β_ 1:16; *E*/*Z* = 48:52).

**Figure 3:**
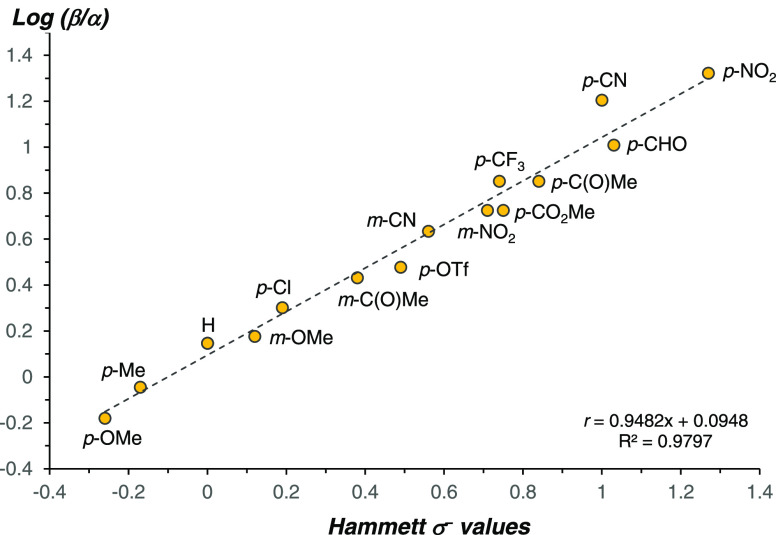
Plot of the log of regioselectivity (β/α) against Hammett σ^–^ values built from the scope of Figure 2B.

The strong dependence of the regioselectivity of insertion to the electronics of the aryl bromide for the β-selective assisted tandem reaction can be quantified by plotting a linear free energy relationship using Hammett σ^–^ values for both *meta*- and *para*-substituted aryl bromides (Figure 3). An excellent correlation, which spans across an unusually wide range of Hammett values (Δ_σ_ = 1.53), was obtained using the examples reported in Figure 2B. The Hammett plot has a positive ρ-value of 0.95, implying that the regioselectivity of insertion is sensitive to the electronic nature of the transient [Pd–Ar] intermediates, with the site selectivity increasing with more electron-poor aryl bromides (i.e., when the Pd–aryl becomes more electrophilic). Moreover, the correlation was optimal using the (σ^–^) scale, indicating that the regioselective outcome is dominated by the stabilization of a negative charge by resonance effects (vide infra, computational analysis).

**Figure 4:**
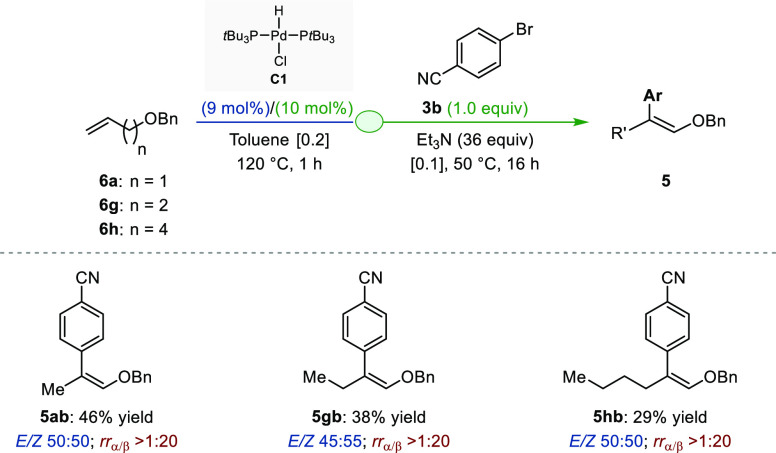
Proof of concept for the assisted tandem [remote isomerization/Heck β-arylation].

We next sought to develop an assisted tandem protocol that would combine the remote isomerization of alkenyl ethers with a regiodivergent Heck arylation (Figure 4). Preliminary investigations revealed that **C1** has only a limited ability to sustain isomerization over more than one carbon atom in ethereal solvents even at high temperatures. This imposed the need to identify novel reaction conditions to achieve long-distance migration of the alkene unit without impacting the subsequent cross-coupling reaction. Despite reassessment of the stoichiometry and evaluation of several solvents and additives along with temperature variations, we have not been able to devise a system compatible with the α-selective Heck arylation. In contrast, we found that when heated at 120 °C in toluene, **C1** isomerized alkenyl benzyl ethers **6a** (*n* = 1), **6g** (*n* = 2) and **6h** (*n* = 4) to the corresponding vinyl ethers. Subsequent β-selective Heck arylation with **3b** was achieved by in situ base-meditated reductive elimination of the [Pd–H] intermediate with an excess of Et_3_N and without solvent switch to deliver **5ab**, **5gb**, and **5hb** in 46, 38, and 29% yields, respectively. While the regioselectivity remained excellent, no stereocontrol could be achieved. The preliminary results reported in Figure 4 are likely to serve as blueprints for further developments of the β-selective process.

### Computational Studies

We sought to obtain additional information on the origin of the regioselectivity for both Heck arylation processes by a series of density functional theory calculations (DFT) using the ORCA 5.0.3. software package.^74^ (*E*)-1-Methoxypropene was used as a model substrate to simplify the conformational space. The *para*-CN-substituted aryl triflate **2b** and aryl bromide **3b** were selected as electrophiles because they provided perfect α- and β-selectivity, respectively.

#### α-Selective Heck Arylation

**Figure 5:**
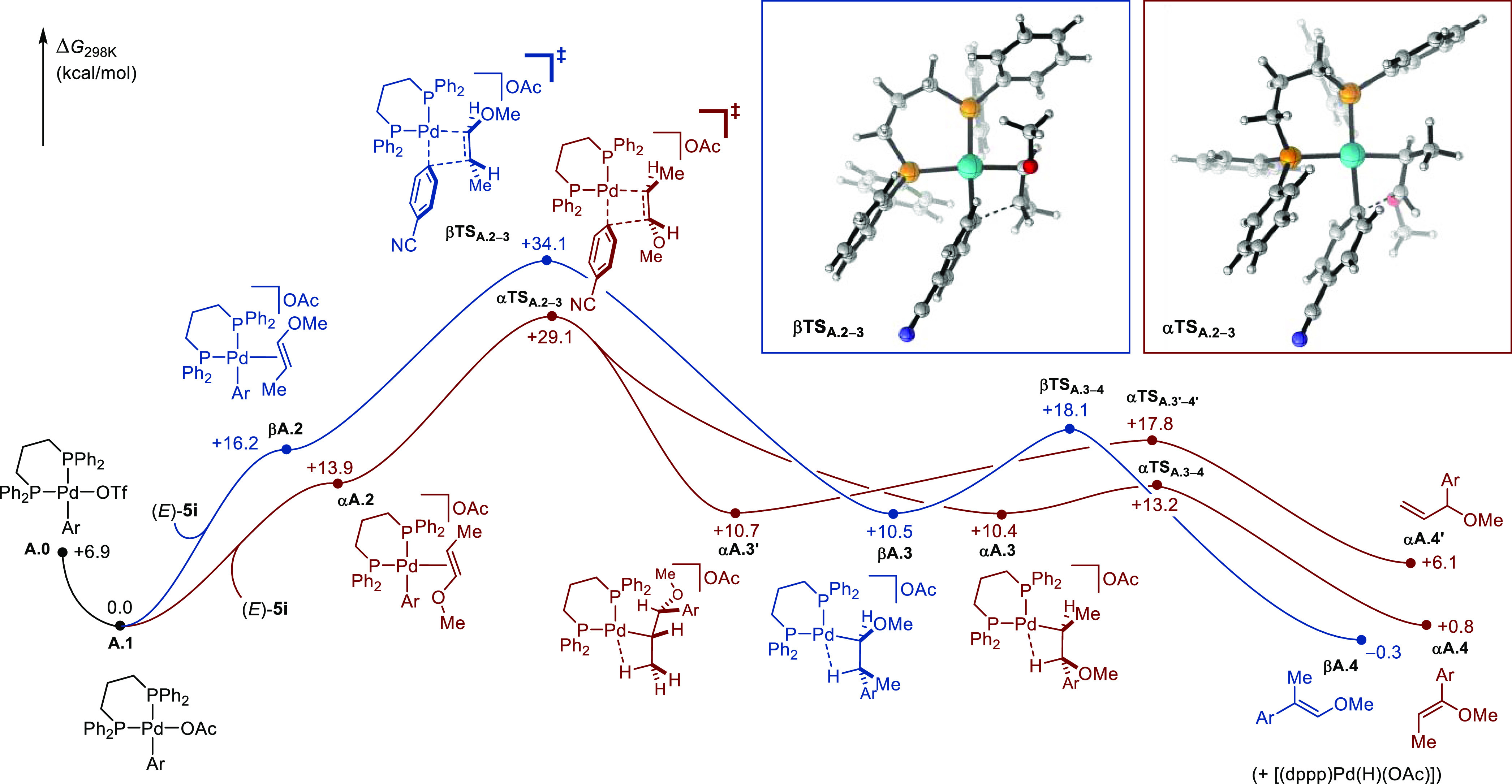
Computed free energy reaction profile (kcal/mol) for the Pd-catalyzed α-selective arylation of substituted (*E*)-**5i** starting from [(dppp)Pd(Ar)(OTf)] (Ar = 4-CN-C_6_H_4_). Level of theory: DLPNO-CCSD(T)/def2-TZVPP//ωB97X-D3/def2-mTZVPP with CPCM(THF).^76−82^ Graphics generated using CYLview (OAc ions omitted for clarity).^83^

Jutand, Amatore, and co-workers showed that weakly coordinating iodide and triflate ions are readily displaced in solution by acetate ions in oxidative addition complexes of general formula [(dppp)Pd(Ar)(X)] (where X = I, OTf).^75^ In preliminary calculations, we indeed found that the [(dppp)Pd(Ar)(OTf)] (Ar = 4-CN-C_6_H_4_) that is expected to initiate catalysis, lies 6.9 kcal/mol higher in energy than the corresponding acetate derivative. Therefore, [(dppp)Pd(Ar)(OAc)] (noted **A.1**) was elected as starting point to investigate the α-selective Heck arylation, and the corresponding computed free energy reaction profile is shown in Figure 5. Due to the relatively low dielectric constant of the solvent (ε_2-MeTHF_ = 7.0), all cationic species were calculated as contact ion pairs with explicit counter-ions. Initial displacement of the acetate ion by an incoming molecule of enol ether (*E*)-**5i** was found to be endergonic by 13.9 kcal/mol to access intermediate **αA.2**, where C_α_ and the *ipso* carbon C(Ar)_ipso_ of the aryl moiety are poised well for subsequent migratory insertion. The latter proceeds via **αTS_2-3_** at +29.1 kcal/mol to form **αA.3** which is characterized by an agostic interaction with H_α_ and lies at +10.4 kcal/mol. Subsequent β-hydride elimination occurs with a barrier at +13.2 kcal/mol (**αTS**_**A3-4**_) and provides access to the α-arylation Heck product **αA.4**.^84^ A second Pd complex featuring an agostic interaction with H_β_ (**αA.3**′) was found to be slightly less stable and β-hydride elimination leading to allyl ether **αA.4**′ appeared less accessible (**αTS**_**A3**′**-4**′_ = +17.8 kcal/mol). From **A.1**, the competing β-arylation pathway is initiated by binding of the enol ether with C_β_ in close proximity to the *cis*-coordinated σ-aryl ligand (**βA.2**) followed by migratory insertion via **βTS**_**A2-3**_ at +34.1 kcal/mol. β-Hydride elimination from the agostic intermediate **βA.3** proceeds with a barrier at +18.1 kcal/mol to give the β-arylation Heck product **βA.4**. The energy difference for the barrier of migratory insertion (ΔΔ*G*_(**αTSA2-3**–**βTSA2-3**)_ = +5.0 kcal/mol) is fully consistent with the level of selectivity measured experimentally (*rr*_α/β_ > 20:1). The reaction profile using (*Z*)-**5i** was also computed and led to similar conclusions. Most notably, the energy difference for the barrier of the selectivity-determining migratory insertion step is of the same order of magnitude (ΔΔ*G* = +4.8 kcal/mol) (see the SI for the full profile).

#### β-Selective Heck Arylation

**Figure 6:**
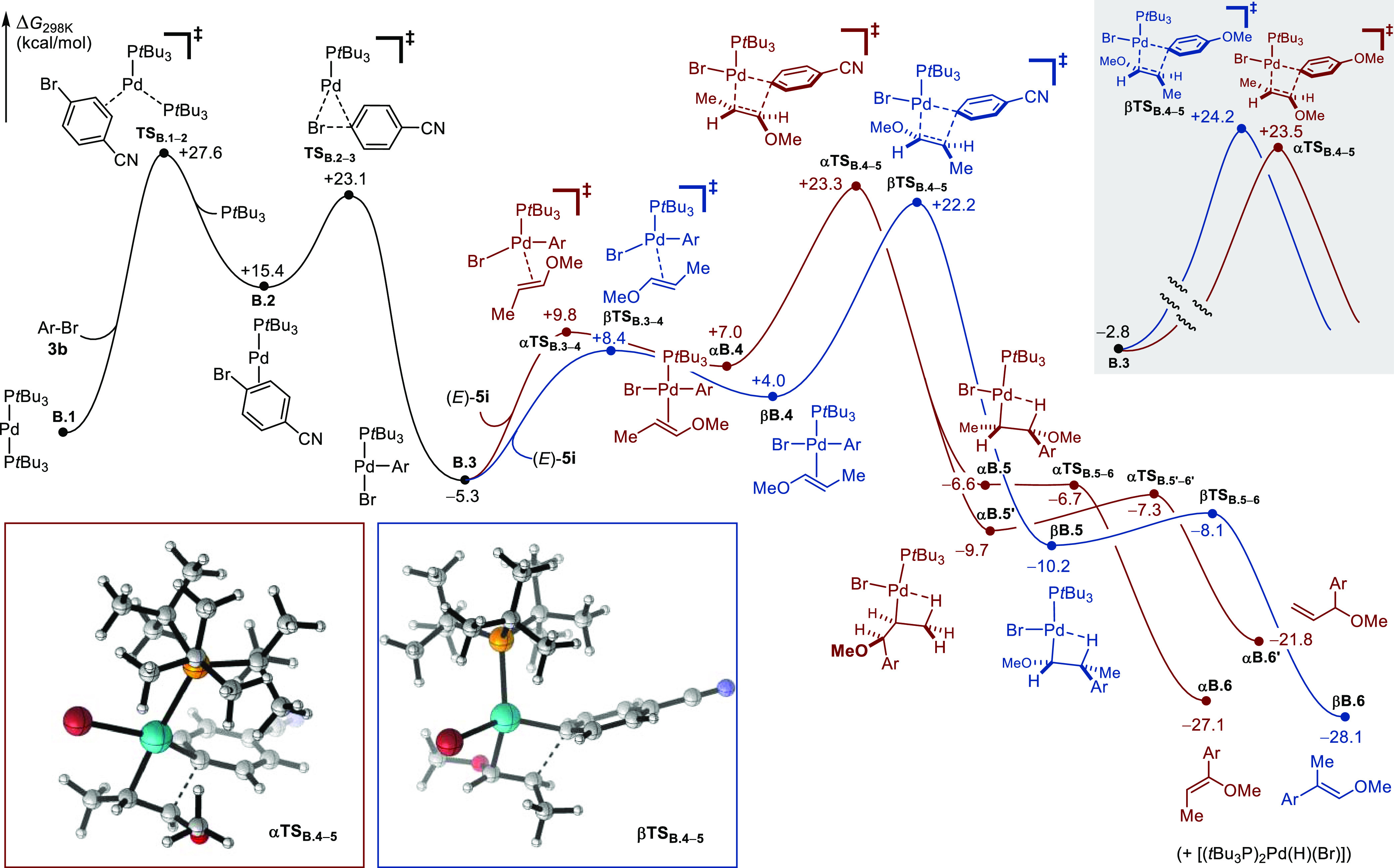
Computed free energy reaction profile (kcal/mol) for the Pd-catalyzed β-selective arylation of substituted (*E*)-**5i** starting from [(*t*Bu_3_P)_2_Pd] (Ar = 4-CN-C_6_H_4_). Inset: selectivity-determining step for Ar = 4-OMe-C_6_H_4_. Level of theory: DLPNO-CCSD(T)/def2-TZVPP//ωB97X-D3/def2-mTZVPP with CPCM(THF).^76−82^ Graphics generated using CYLview.^83^

The computed free energy reaction profile for the β-selective Heck arylation is depicted in Figure 6. Starting from [Pd(P*t*Bu_3_)_2_] (noted **B.1**), ligand dissociation occurs through an associative process by η^2^-coordination of 4-bromobenzonitrile (**3b**) via **TS**_**B1-2**_ at +27.6 kcal/mol leading to **B.2** (+15.4 kcal/mol). This step displays the highest barrier in the profile and is therefore thought to be rate-determining, at least in the early stages of the reaction.^85^ Oxidative addition proceeds via **TS**_**B2-3**_ at +23.1 kcal/mol to afford the T-shaped neutral palladium complex **B.3**.^86,87^ Reversible coordination of (*E*)-**5i** to the vacant site generates two geometrical isomers **αB.4** (+7.0 kcal/mol) and **βB.4** (+4.0 kcal/mol).^88^ Subsequent migratory insertion in the former proceeds via **αTS**_**B4-5**_ at +23.3 kcal/mol, while migratory insertion in the latter is more favorable (**βTS**_**B4-5**_ at +22.2 kcal/mol). The resulting intermediates are all characterized by the presence of an agostic interaction, with **βB.5** being more stable than **αB.5** and **αB.5**′ (−10.2 vs −6.6 and −9.7 kcal/mol, respectively). The ensuing product-forming β-hydride eliminations all occur with low free energy activation barriers.^24^ Overall, migratory insertion is the selectivity-determining step and the β-arylation pathway is favored over the α-arylation pathway with an energy difference (ΔΔ*G*_(**βTSB4-5**–**αTSB4-5**)_ = 1.1 kcal/mol) that is consistent with the selectivity preference obtained experimentally (*rr*_α/β_ = 1:16). When (*Z*)-**5i** was employed as the substrate, a similar energy profile was computed and the selectivity-determining step displayed an energy difference of 1.4 kcal/mol in favor of the β-arylation product, in line with the trend observed experimentally (see the SI for the full profile). Finally, when *p*-MeO-substituted aryl bromide **3i** was used as substrate, the α-arylation pathway was found to be favored with an energy difference of ΔΔ*G*_(**αTSB4-5**–**βTSB4-5**)_ = 0.7 kcal/mol, in agreement with the experimental observations (inset in Figure 6). Despite these small energy differences with respect to the accuracy of the method, the experimental trends appeared well reflected. This prompted us to use these results as a basis to obtain additional computational insights.

#### Energy Decomposition Analysis (EDA)

**Figure 7:**
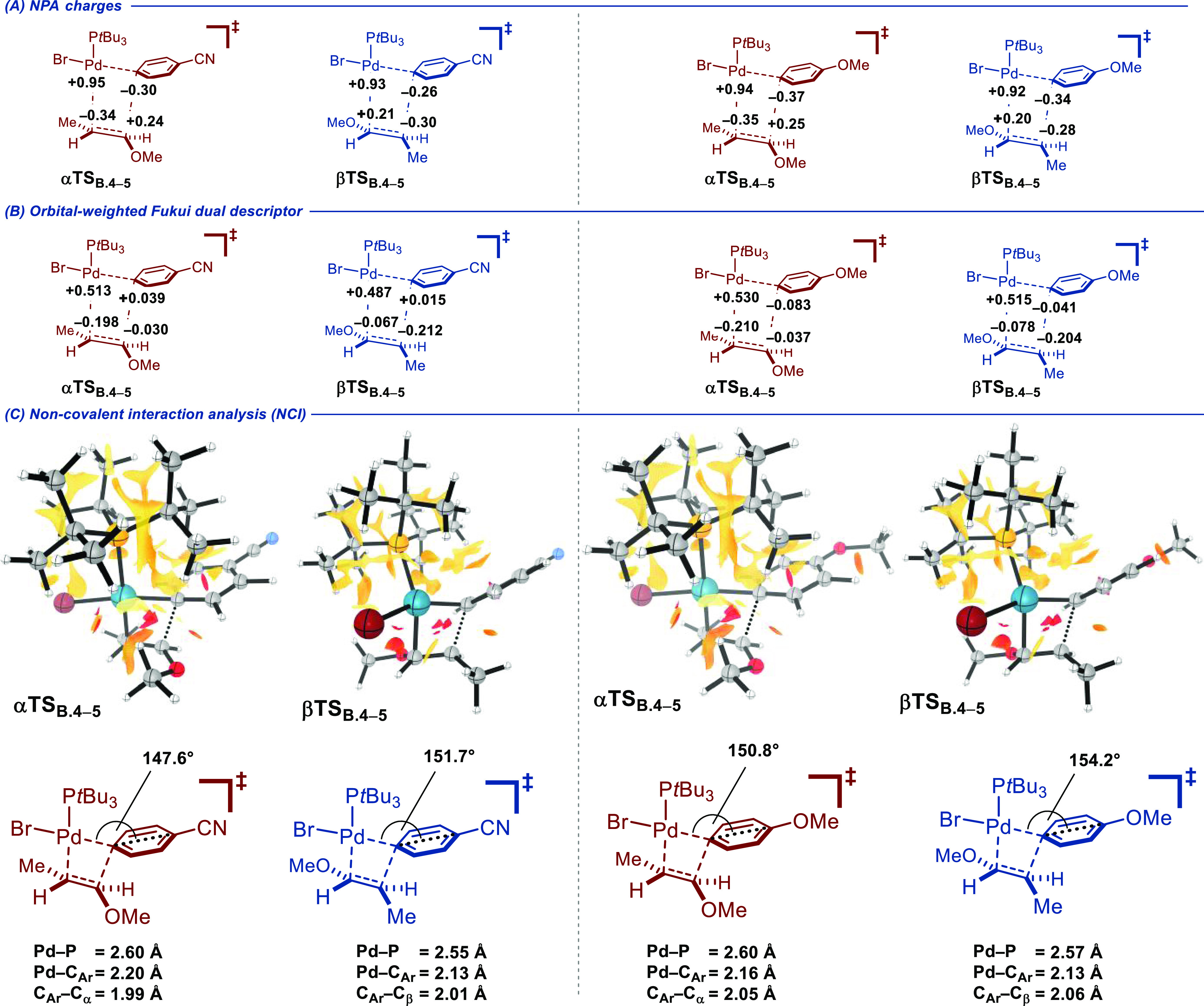
(A) NPA charges calculated with the NBO 6.0 software package,9^2^ on isolated fragments taken in their in-adduct geometries. (B) Orbital-weighted Fukui dual descriptor (OWDD) on isolated fragments taken in their in-adduct geometries. (C) NCI plots drawn with an isosurface for the reduced density gradient (RDG) at 0.35 mapped with sign(λ_2_)ρ as a color scale from 0.00 au (pale yellow) to 0.02 au (red); data pertaining to attractive interactions (sign(λ_2_)ρ < 0) was excluded. Fukui indices and grid data for NCI analysis were computed with Multiwfn 3.8.9^3^.

To rationalize the strong influence of the electronic nature of the aryl bromide on the regioselectivity of the β-selective Heck arylation using [Pd(P*t*Bu_3_)_2_] as precatalyst, we conducted a series of energy decomposition analyses (EDA), based on ORCA’s local energy decomposition (LED) scheme,^89,90^ on the structures of the transition states of the regio-determining migratory insertion step. Because they afford the cross-coupling products with a reversal of regioselectivity, 4-bromobenzonitrile **3b** and 4-bromoanisole **3i** were selected as electrophilic components (**5ab**: *rr*_α/β_ = 1:16; **5ai**: *rr*_α/β_ = 2:1) and (*E*)-1-methoxypropene was employed as an olefinic partner. The equation above Table 3 was used to estimate the different factors that may govern regioselectivity: thermodynamic, steric, electronic, and dispersion, the sum of which amounts to Δ*G*_TS_^‡^. The thermodynamic term includes Δ*G*_thermo_, which corresponds to all thermal and entropy corrections at 298.15 K calculated within the rigid-rotor harmonic oscillator (RRHO) approximation at the ωB97X-D3/def2-mTZVPP level, as well as ΔΔ*G*_solv_ which was computed using the continuum solvation model conductor-like polarizable continuum model (C-PCM) (THF). The remaining contributions stem from a Morokuma-style decomposition scheme.^94^ Δ*E*_distort_ and Δ*E*_Pauli_ were summed into a steric term. Δ*E*_distort_ is the geometrical preparation energy term, related to the energy needed to distort the fragments from their equilibrium isolated geometry to the geometry adopted in the adduct, while Δ*E*_Pauli_ is a repulsive term associated with pure sterics. The terms associated with electrostatic interaction (Δ*E*_elstat_) and with orbital interaction (Δ*E*_orb_) were summed into a single electronic component. The final element Δ*E*_disp_ reflects the dispersion interactions (see the SI for computational details). Table 3 displays the results of the EDA of the four transition states (**αTS**_**B4-5**_ and **βTS**_**B4-5**_ for R = CN and R = OMe). A comparison of the transition states calculated using 4-bromobenzonitrile **3b** shows that the electronic and steric terms are of the greatest magnitude. While the electronic contribution favors the α-regioselectivity by 17.2 kcal/mol, the steric effects favor β-regioselectivity to a lesser extent (15.7 kcal/mol). A closer analysis of the electrostatic contribution using natural population analysis (NPA) charges reveals a coherent orientation of the two fragments with a constructive alignment of the positive and negative partial charges in **αTS**_**B4-5**_ but not in **βTS**_**B4-5**_ (Figure 7A and Table 3, entry Δ*E*_elstat_). A similar conclusion was drawn for the orbital interaction contribution using an orbital-weighted Fukui dual descriptor (OWDD), which shows the favorable positioning of the most nucleophilic site of the alkene with the most electrophilic site of the metal fragment in **αTS**_**B4-5**_ (Figure 7B and Table 3, entry Δ*E*_orb_).^95,96^ A visual representation of steric repulsions resulting from noncovalent interaction analysis (NCI) points to a greater degree of buttressing in **αTS**_**B4-5**_ than in **βTS**_**B4-5**_. Structurally, the more acute Pd–C(Ar)_ipso_–C(Ar)_para_ angle is proposed to result in a greater hindrance in **αTS**_**B4-5**_, which in turn induces elongation of the Pd–C(Ar)_ipso_ and Pd–P bonds (Figure 7C and Table 3, entry Σ(steric)). The eclipsed conformation of the alkene moiety in **αTS**_**B4-5**_ with respect to the C_α_–O bond is likely to further disfavor this pathway. Finally, the contributions of both the dispersion and thermodynamic components, albeit of smaller magnitudes, gear the system toward an overall β-selective outcome.

**Table 3:**
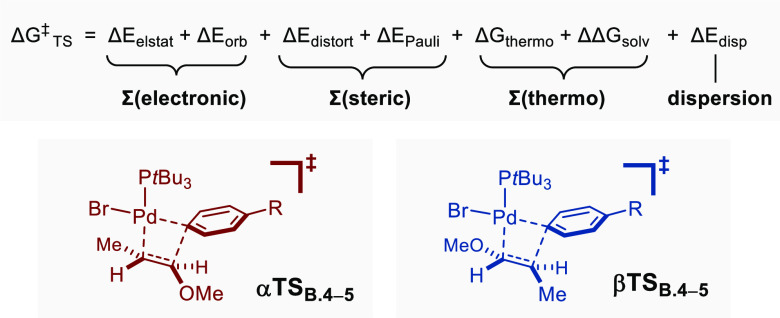
Energy Decomposition Analysis (EDA)^a^

	R = CN		R = OMe	
	αTSB.4-5	βTSB.4-5	αTSB.4-5	βTSB.4-5
Δ*G*_TS_^‡^	28.6	27.5	26.3	27.0
Δ*E*_elstat_	–176.0	–167.6	–170.5	–163.2
Δ*E*_orb_^b^	–143.8	–135.0	–131.1	–123.9
**Σ(electronic)**	**–319.8**	**–302.6**	**–301.6**	**–287.1**
Δ*E*_distort_	64.6	59.0	59.0	54.6
Δ*E*_Pauli_	299.9	289.9	284.3	276.9
**Σ(steric)**	**364.5**	**348.8**	**343.3**	**331.6**
Δ*G*_thermo_	15.6	14.1	15.5	14.4
ΔΔ*G*_solv_	2.0	3.3	2.3	3.7
**Σ(thermo)**	**17.6**	**17.4**	**17.9**	**17.8**
**Δ*E***_**disp**_	**–33.3**	**–35.3**	**–32.0**	**–32.9**

a See the Supporting Information for computational details.

b For further decomposition of Δ*E*_orb_ into its different components see the SI.^91^

The comparative analysis between **αTS**_**B4-5**_ and **βTS**_**B4-5**_ for the reaction employing 4-bromoanisole **3i** (R = OMe) showed that the electronic and steric components are also acting oppositely, with the α-selective pathway being strongly favored overall (Σelectronic = 14.5 kcal/mol; Σsteric = 11.7 kcal/mol). Quite notably, with an electron-richer aryl ring, the NPA indicates a significantly increased accumulation of negative charge at C(Ar)_ipso_ in both **αTS**_**B4-5**_ and **βTS**_**B4-5**_ (Figure 7A). Likewise, increased nucleophilic character is observed at C(Ar)_ipso_ in **βTS**_**B4-5**_ as exhibited by the sign inversion of its Fukui index. This is expected to enhance the repulsive nature of its interaction with C_β_ of the alkene fragment (Figure 7B). Even though more constrained than **βTS**_**B4-5**_, **αTS**_**B4-5**_ is not displaying as much steric buttressing as its analogue calculated for R = CN. This is supported by the relatively open Pd–C(Ar)_ipso_–C(Ar)_para_ angle (150.8°) and shorter Pd–C(Ar)_ipso_ and Pd–P bond lengths (Figure 7C). The weight of the dispersion and thermodynamic components essentially cancel each other out, leading overall to an α-selective pathway. Taken together, these observations suggest a significant influence of the substitution on the aryl moiety primarily on the electronics of the system. Specifically, it affects the nucleophilic character and the accumulation of a negative partial charge at C(Ar)_ipso_, thus accounting for the correlation obtained experimentally between regioselectivity and Hammett σ^–^ values. Consequently, transition states for electron-richer aryl moieties occur earlier along the reaction coordinate, as can be seen by the comparatively longer C(Ar)_ipso_–C(alkene) bond distances with R = OMe (Figure 7C), which in turn induce less distortion, a more open Pd–C(Ar)_ipso_–C(Ar)_para_ angle, and overall alleviate the steric effects that were conversely favoring β-selectivity.

### Addressing the Stereoselectivity Issue by Ligand Control or Postcatalytic Stereo-Correction

**Figure 8:**
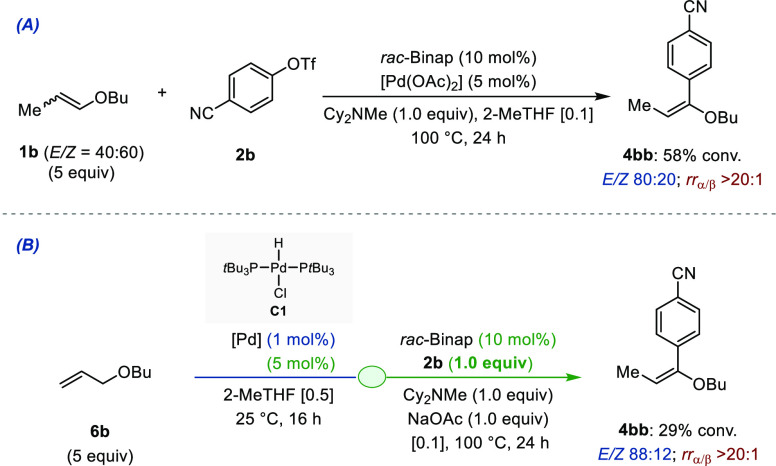
(A) Ligand-controlled stereoselective Heck α-arylation of enol ether **1b**. (B) Ligand-controlled stereoselective [isomerization/Heck α-arylation] assisted tandem process.

At the outset of our investigations, in addition to controlling the site selectivity of insertion of the [Pd–Ar] intermediate across the C=C bond of the transiently generated enol ether, we anticipated that exerting high levels of *E*/*Z* stereocontrol would probably constitute a most acute challenge. Upon reevaluating the Heck reaction between **1b** (*E*/*Z* = 40:60) and aryl triflate **2b** under the optimized reaction conditions using *rac*-Binap, we found that the cross-coupling product **4bb** could be generated in 58% conversion with excellent α-regioselectivity (*rr*_α/β_ > 20:1) and a much improved level of stereocontrol compared to dppp (*E*/*Z* = 80:20), while the recovered alkene still consisted in an *E*/*Z* ∼40/60 stereoisomeric mixture (Figure 8A).

When the corresponding tandem reaction was performed starting from allyl ether **6b**, the α-arylation product **4bb** was obtained with an excellent 88:12 *E*/*Z* ratio. Despite the reduced conversion (29%), this result serves as a proof of principle to demonstrate that ligand control can be achieved in the [isomerization/α-arylation] assisted tandem process, independently of the stereochemistry of the transient enol ether (Figure 8B).

**Figure 9:**
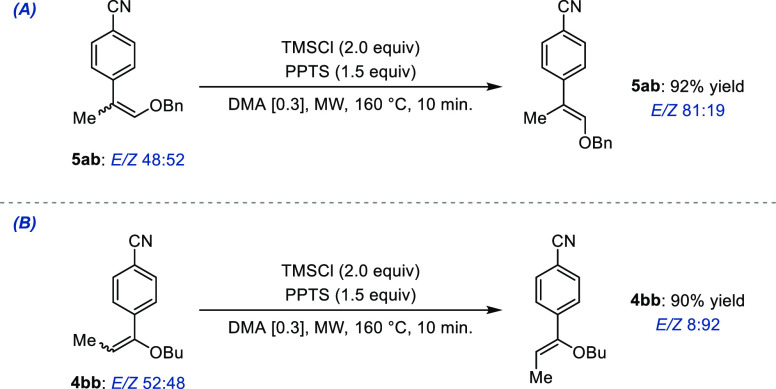
Postcatalytic stereo-correction using Lewis-assisted protic activation protocol.

By contrast, because the Pd-catalyzed isomerization is not stereoselective and the experimental conditions developed for the β-arylation are stereospecific, we sought to identify a postcatalytic protocol that would enable to improve the low level of stereoselectivity obtained after the [isomerization/Heck arylation]. Inspired by a recent report from the Dixon group, we reasoned that the formation of an oxocarbenium ion by Lewis-acid-assisted protic activation of the trisubstituted enol ethers may lead to amplification of the stereoselectivity obtained by assisted tandem Pd catalysis.^97^ Gratifyingly, we found that when a 48:52 *E*/*Z* mixture of **5ab** was reacted with trimethylsilyl chloride (TMSCl) and pyridinium *p*-toluenesulfonate (PPTS), the stereoselectivity could be significantly improved to *E*/*Z* 81:19 within only 10 min in DMA under microwave irradiation (Figure 9A).

Using the same protocol, starting from a stereoisomeric mixture of the α-arylated product **4bb** (*E*/*Z* = 52:48), the *Z* stereoisomer could be obtained almost exclusively (*E*/*Z* = 8:92) in nearly quantitative yield (Figure 9B). The latter experiment complements the results disclosed in Figure 8 as it affords preferentially the stereoisomer of opposite configuration.

## Conclusions

In summary, we have developed two complementary assisted tandem protocols for the regiodivergent Heck arylation of transiently generated acyclic enol ethers, starting from alkenyl ethers. Using a readily available precatalyst, the approach combines an alkene isomerization with a cross-coupling reaction by intercepting the putative [Pd–H] that is common to both catalytic manifolds. In the first system, the catalyst is modified by the addition of a chelating bisphosphine ligand (dppp), an organic base (Cy_2_NMe), sodium acetate, and aryl triflates were used as electrophilic coupling partners. This method is highly regioselective and provides access to a wide array of stable trisubstituted α-aryl enol ethers in practical yield but moderate stereoselectivity. In the second system, the catalyst is simply modified by the addition of an excess of an organic base (Et_3_N) after completion of the isomerization reaction. While excellent levels of β-selectivity are obtained with electron-deficient aryl bromides, electron-rich electrophiles lead to lower levels of regiocontrol. We showed that alkenyl ethers are competent substrates for a [remote isomerization/Heck β-arylation]. Because the cross-coupling step is stereospecific, the low *E*/*Z* selectivity obtained overall is proposed to directly reflect the absence of stereocontrol in the [Pd–H]-catalyzed isomerization.

Computational analyses suggest that migratory insertion is regio-determining for both the Pd-catalyzed α-selective and β-selective arylations. For the latter, an energy decomposition analysis revealed that while electronic factors favor the α-regioselectivity, steric effects favor β-regioselectivity. Although of lower magnitude, the contributions of the thermodynamic and dispersion components are sufficient to favor β-selectivity in the case of electron-deficient aryl halides. Finally, we demonstrated that achieving ligand control in the stereoselective assisted tandem [isomerization/Heck α-arylation] might not be illusive (*E*/*Z* up to 88:12). The complementary *Z* isomer can be obtained using a postcatalytic Lewis-acid-assisted protic activation (*E*/*Z* 8:92). This operationally simple protocol also permits significant amplification of the stereoselectivity for trisubstituted β-aryl enol ethers (*E*/*Z* 81:19). Studies aimed at developing further assisted tandem catalytic systems based on alkene isomerization are underway in our laboratories.

## Methods

### General Procedure for the Assisted Tandem [Isomerization/Heck α-Arylation]

In a 5 mL J-Young tube, **C1** (8.2 mg, 0.015 mmol, 4.5 mol % to **6b**—5 mol % to **2**) was dissolved in 2-MeTHF (3 mL, **2** will be 0.1 M). After 5 min at 25 °C, allyl ether **6b** (5.0 equiv) was added to the yellow solution and the reaction mixture was stirred for 1 h at 25 °C. Next, dppp (12.4 mg, 0.03 mmol, 10 mol % to **2**), NaOAc (24.6 mg, 0.3 mmol, 1 equiv), the organic base Cy_2_NMe (58.6 mg, 0.3 mmol, 1 equiv), and aryl triflate **2** (75.3 mg, 0.3 mmol 1 equiv) were added in sequence. The resulting yellow solution was stirred for 24 h at 100 °C. Once the temperature had reached 25 °C, the solution was diluted with Et_2_O (10 mL) and washed with a 10% wt % NH_4_Cl_aq_ (3 × 10 mL) and with brine (20 mL). The organic phase was dried over Na_2_SO_4_, filtered, and the solvent removed under reduced pressure. The regioisomeric and stereoisomeric ratios were assessed by ^1^H NMR analysis of the crude reaction mixture. The residue was purified by column chromatography to afford product **4**.

### General Procedure for the Assisted Tandem [Isomerization/Heck β-Arylation]

In a 5 mL J-Young tube, **C1** (8.2 mg, 0.015 mmol, 1 mol % to **6a**—5 mol % to **3**) was dissolved in TBME (1.5 mL, 3 will be 0.1 M). After 5 min at 25 °C, allyl benzyl ether **6a** (222 mg, 1.5 mmol, 5 equiv) was added to the yellow solution and the reaction mixture was stirred for 16 h at 25 °C. Next, triethylamine (1.5 mL, 36 equiv) and aryl bromide **3** (0.3 mmol, 1 equiv) were added in sequence. The resulting yellow solution was stirred at 50 °C for 24 h. Once the temperature had reached 25 °C, the resulting heterogeneous solution was filtered over a pad of Celite and washed with Et_2_O (15 mL). The solvent was removed under reduced pressure, and the regioisomeric and stereoisomeric ratios were assessed by ^1^H NMR analysis of the crude reaction mixture. The residue was purified by two consecutive column chromatography ((a) SiO_2_ containing 10 wt % AgNO_3_ using pentane/Et_2_O as eluent; (b) SiO_2_, using pentane/Et_2_O as eluent) to afford product **5**.

### Conformational Sampling

Conformational searches were run for all species with a significant degree of conformational freedom, using the Grimme group’s CREST software with the GFN2-xTB method.^98,99^ Weak constraints (*k* = 0.05 Hartree/Bohr^2^) were applied to maintain the coordination geometry around the metal center on a case-by-case basis. For transition states, the relevant coordinate (e.g., bond distance) was constrained during the conformational sampling. The ensembles obtained were further sorted using the implemented principle component analysis (PCA) and *k*-means sorting clustering algorithm to generate a representative set of 10 geometries. These geometries were further optimized at the DFT level (described thereafter), and the lowest-energy conformer was kept for the energy profile.

### Geometry Optimizations

All DFT calculations were carried out using the ORCA 5.0.3 package.^74^ Geometries were optimized using Grimme’s dispersion-corrected ωB97X-D3 functional,^80,81^ in conjunction with the def2-mTZVPP basis set on all atoms and the associated def2-ECP effective core potential on Pd.^82^ The RIJCOSX approximation was used to reduce the computational cost of calculations using the def2/J auxiliary basis set and integration grids set to DefGrid3.^79^ The conductor-like polarizable continuum model (C-PCM) was used to account for solvent effects in THF.^100^ All stationary points were verified to be minima (zero imaginary frequency) or transition states (one imaginary frequency) by frequency analysis at the same level of theory.

### Thermodynamics Corrections

Thermodynamics were computed within the rigid-rotor harmonic oscillator (RRHO) approximation at 298.15 K. These computed free energies at a 1 atm standard state were corrected to a 1 M solution standard state by a constant Δ*G*_SS_ = *RT* ln(24.46) = 1.89 kcal/mol, where 24.46 L/mol is the molar volume at 1 atm and 298.15 K.

### Single Point Energy Corrections

Single point energy calculations were carried out at the DLPNO-CCSD(T) level of theory with TightPNO settings,^76^ in conjunction with the def2-TZVPP basis set associated with def2-ECP effective core potential on Pd and the def2-TZVPP/C auxiliary basis set,^77,78,100^ in THF using the default C-PCM implementation in ORCA 5.0.3 and using DefGrid3.

### Atomic Properties

Natural population analysis (NPA) charges were calculated with the NBO 6.0 software package.^92^ Fukui orbital-weighted dual descriptor indices were calculated using the Multiwfn 3.8 software.^93^ Fukui indices (orbital weight parameter Δ = 0.1 au) and NPA charges were obtained on both fragments defined in the decomposition scheme (vide infra), in their in-adduct geometry and using the Hartree–Fock reference wavefunction obtained during the DLPNO-CCSD(T) single point calculations.

## Supporting Information Available

Experimental procedures, characterization of all new compounds, spectroscopic data, and computational procedures (PDF)
Crystallographic data (ZIP)
Molecular coordinates of computed structures (XYZ)

## Supplementary Material

au2c00645_si_002.zip

au2c00645_si_003.xyz
