# Supplementary material for: Assisted Tandem Pd Catalysis Enables Regiodivergent Heck Arylation of Transiently Generated Substituted Enol Ethers
Source: JACS Au. 2023 Jan 12;3(1):261–74. doi: 10.1021/jacsau.2c00645 (PMC9875267; doi:10.1021/jacsau.2c00645)
Supplement: Supplementary file 1 — au2c00645_si_001.pdf [file au2c00645_si_001.pdf]

Assisted Tandem Pd Catalysis Enables Regiodivergent Heck Arylation of Transiently-Generated Substituted Enol Ethers

Thomas Duhamel,‡ Simone Scaringi,‡ Baptiste Leforestier,‡ Amalia I. Poblador-Bahamonde and Clément Mazet*

Department of Organic Chemistry, University of Geneva

30, Quai Ernest Ansermet, 1211 Geneva 4, Switzerland

clement.mazet@unige.ch

Table of Contents

**1.**	**General information**	**S3**
**2.**	**General Procedures (GPs)**	**S4**
**3.**	**Preparation of aryl triflates 2**	**S10**
**4.**	**Preparation of vinyl ethers 1 and alkenyl ethers 6**	**S16**
	**4.1** Synthesis of vinyl ether (*Z*)-**1a**	**S16**
	**4.2** Synthesis of vinyl ethers **1a** and **1b**	**S17**
	**4.3** Synthesis of alkenyl ethers **6c-h**	**S18**
**5.**	**Preparation of C1 and C2**	**S21**
**6.**	**Heck reaction optimization**	**S22**
	**6.1** α-regioselectivity	**S22**
	**6.2** β-regioselectivity	**S23**
**7.**	**Optimization of the isomerization reaction**	**S24**
**8.**	**Assisted tandem (AT) reaction**	**S27**
	**8.1** Optimization – α-regioselectivity	**S27**
	**8.2** Optimization – β-regioselectivity	**S28**
	**8.3** Scope of AT reaction – α-regioselectivity	**S29**
	**8.4** Scope of AT reaction – β-regioselectivity	**S49**
**9.**	**Hammett plot**	**S77**
**10.**	**Computational studies**	**S78**
	**10.1** Basis set convergence – DLPNO-CCSD(T) single points	**S79**
	**10.2** Calculated thermodynamics	**S80**
	**10.3** Energy decomposition analysis – LED scheme	**S87**
	**10.4** ETS-NOCV analysis $\alpha TS_{B.4-5}$ on $\beta TS_{B.4-5}$ and for R = CN	**S90**
	**10.5** HOMO and LUMO for $\alpha TS_{B.4-5}$ and $\beta TS_{B.4-5}$	**S92**
**11.**	**Stereoselectivity: ligand control and post-catalytic stereo-correction**	**S94**
**12.**	**References**	**S96**
**13.**	**NMR spectra**	**S98**

1. General information

Unless otherwise noted, all reactions were carried out under an inert atmosphere of nitrogen using a M.Braun glove-box. Solvents were dried over activated alumina columns and further degassed by three successive "freeze-pump-thaw" cycles. All transition metal precatalysts and ligands were stored and weighted inside a M.Braun glove-box. Commercial precatalysts, ligands, allyl ethers **6a-6b**, aryl bromides **3a-f**, **3h-l**, **3q-r**, **3t-z**, aryl triflates **2a** and **2k** were purchased from Aldrich, Fluka, TCI, Acros or Strem. The commercial or synthetic allyl ethers as well as the aryl triflates were carefully dried over P_2O_5 and kept under inert atmosphere. The organic bases (Cy_2NMe, Et_3N) were distilled, degassed and kept under an inert atmosphere.

NMR spectrometers. NMR spectra were acquired at the University of Geneva NMR platform (https://www.unige.ch/sciences/chiorg/nmr/) using a 500 MHz Avance III Bruker NMR spectrometer equipped with a helium-cooled cryogenic 5-mm DCH ^{13}C-^{1}H/D Bruker probe, a 400 MHz Avance III HD NanoBay spectrometer equipped with a N_2 prodigy cryogenic 5 mm CPP BB(F)-H-D probe or a 300 MHz Avance III, HD NanoBay spectrometer, equipped with a 5 mm PA BBO, BB(F)-H-D probe. All ^{1}H and $^{13}C\{^{1}H\}$ experiments were internally referenced with respect to $CDCl_3$ solvent signals and acquired at 298 K. $^{31}P\{^{1}H\}$ and $^{19}F\{^{1}H\}$ NMR chemical shifts are reported in ppm with reference to the lock.

Infrared spectra were obtained on a Perkin–Elmer spectrum 100 FT-IR spectrometer using neat samples on a diamond ATR Golden Gate sampler.

The mass spectrometric data were obtained at the mass spectrometry facility of the University of Geneva (http://www.unige.ch/sciences/sms/).

Thin layer chromatography (TLC) was performed on plates of silica pre-coated with 0.25 mm Kieselgel 60 F_{254} from *Merck*. Flash chromatography was performed using silica gel SiliaFlash® P60 (230-400 mesh) from *Silicycle* or aluminum oxide (neutral, Brockmann I, 40-300 mesh) from *Thermo Scientific*.

2. General Procedures (GPs)

General Procedure I (GP I)

In a flame-dried Schlenk flask, the appropriate phenol (1.0 equiv) and pyridine (2.0 equiv) were dissolved in CH_2Cl_2 (0.62 M) at 0 °C. Trifluoromethanesulfonic anhydride (1.3 equiv) was added slowly (5 min). The mixture was warmed to 25 °C and stirred overnight. The mixture was diluted with Et_2O (10 mL), quenched with 3M HCl_{aq} (20 mL) and washed successively with a saturated aqueous solution of $NaHCO_3$ (20 mL) and brine (20 mL). The organic phases were dried over Na_2SO_4, filtered and the solvent removed under reduced pressure. The residue was purified by silica gel chromatography using pentane/Et_2O as eluent to afford the corresponding aryl trifluoromethanesulfonate **2**.[1]

General Procedure II (GP II)

In a flame-dried Schlenk flask, the appropriate phenol (1 equiv) and *N*-(5-Chloro-2-pyridyl)bis(trifluoromethanesulfonimide) (1.2 equiv) were dissolved in CH_2Cl_2 (0.5 M). Triethylamine (4 equiv) was added at 0 °C. The mixture was stirred for 16 h at 25 °C. The reaction mixture was quenched with water (10 mL) and extracted with CH_2Cl_2 (10 mL × 3). The organic layers were combined and dried over anhydrous Na_2SO_4. The mixture was filtered and concentrated under vacuum. The residue was purified by silica gel chromatography using pentane/Et_2O as eluent to afford the corresponding aryl trifluoromethanesulfonate **2**.[2]

General Procedure III (GP III)

In a flame-dried Schlenk flask, a suspension of sodium hydride (1.1 equiv) in DMF (20 mL) was cooled to 0 °C prior to addition of the appropriate alcohol (1 equiv). The reaction was stirred 1 h at 0 °C before adding KI (1.1 equiv) and allyl bromide (1.1 equiv). The reaction was stirred 16 h at 80 °C. Once the system had reached 25 °C, it was quenched with water (10 mL) and diluted with Et_2O (50 mL). The organic phase was washed with water (4 × 100 mL) and brine (100 mL). The organic layer was dried over anhydrous Na_2SO_4, filtered and concentrated under vacuum. The residue was purified by silica gel chromatography using pentane/Et_2O as eluent to afford allyl ether **6**.

General Procedure IV (GP IV)

In a 5 mL J-Young tube, the palladium source (x mol%) and ligand (y mol%) were dissolved in 2-MeTHF (1 mL, **2a** is 0.1 M). The mixture was stirred for 5 min at 25 °C. The organic base Cy_2NMe (n equiv), vinyl ether (*Z*)-**1a** (1.1 – 5.0 equiv) and aryl triflate **2a** (0.1 mmol, 1 equiv) were added in sequence. The solution was stirred for 24 h at 100 °C. The reaction mixture was cooled to 25 °C, the resulting heterogeneous solution was filtered over a pad of Celite® and washed with Et_2O (5 mL). The solvent was removed under reduced pressure, the conversion, regioisomeric and stereoisomeric ratio of products **4aa** and **5aa** were assessed by ^{1}H NMR analysis of the crude reaction mixture using an internal standard.

General Procedure V (GP V)

In a 5 mL J-Young tube, the palladium source (x mol%) and ligand (y mol%) were dissolved in the selected solvent (1 mL, **3a** or **3b** is 0.1 M). The mixture was stirred for 5 min at 25 °C. The organic base (n equiv), vinyl ether (*Z*)-**1a** (5 equiv) and aryl bromide **3a** or **3b** (0.1 mmol, 1 equiv) were added in sequence. The solution was stirred for 24 h at the reported temperature. The reaction mixture was cooled to 25 °C, the resulting heterogeneous solution was filtered over a pad of Celite® and washed with Et_2O (5 mL). The solvent was removed under reduced pressure and the conversion, regioisomeric and stereoisomeric ratio of products **4aa** or **4ab** and **5aa** or **5ab** was assessed by ^{1}H NMR analysis of the crude reaction mixture using an internal standard.

General Procedure VI (GP VI)

In a J-Young NMR tube, catalyst **C1** (x mol%) was dissolved in THF-d_8 (1 mL, **6a** is 0.1 M). After 5 min at 25 °C, allyl benzyl ether **6a** (14.8 mg, 0.1 mmol, 1 equiv) was added to the yellow solution. ^{1}H NMR spectra were recorded every hour for the first 8 hours and a final analysis was recorded after 16 h. The conversion and the stereoisomeric ratio were assessed by ^{1}H NMR analysis of the crude reaction mixture using an internal standard.

General Procedure VII (GP VII)

In a J-Young tube, precatalyst **C2** (x mol%) and $NaBAr_F$ (4.9 mg, 0.055 mmol, 5.5 mol%) were dissolved in the selected solvent (1 mL, **6a** or **6h** is 0.1 M). After 5 min at 25 °C, alkenyl benzyl ether **6** (1 equiv) was added to the solution and the reaction mixture was heated at the reported temperature. Next, the reaction mixture was cooled to 25 °C, filtered over a pad of Celite® and washed with Et_2O (5 mL). The solvent was removed under reduced pressure and the conversion and stereoisomeric ratio of product **1a** or **1h** were assessed by ^1H NMR analysis of the crude reaction mixture using an internal standard.

General Procedure VIII (GP VIII)

In a 5 mL J-Young tube, **C1** (1 or 4.5 mol% to **6a** or **6b** and 5 mol% to **2a** or **2b**) was dissolved in 2-MeTHF (1 mL, **2a** or **2b** will be 0.1 M). After 5 min at 25 °C, the corresponding allyl ether **6a** or **6b** (1.1 - 5 equiv) was added to the yellow solution and the reaction mixture was stirred for the reported time at 25 °C. Next, the ligand dppp (5 - 10 mol% to **2a** or **2b**), NaOAc (0 or 1 equiv), Cy_2NMe (1 equiv) and, after 5 minutes, aryl triflate **2a** or **2b** (0.1 mmol, 1 equiv) were added. The resulting yellow solution was stirred for 24 h at 100 °C. Once the temperature had reached 25 °C, the heterogeneous solution was filtered over a pad of Celite® and washed with Et_2O (5 mL). The solvent was removed under reduced pressure, the conversion, regioisomeric and stereoisomeric ratio of products **4** and **5** were assessed by ^1H NMR analysis of the crude reaction mixture in presence of an internal standard.

General Procedure IX (GP IX)

In a 5 mL J-Young tube, **C1** (1 or 4.5 mol% to **6a** and 5 mol% to **3b**) was dissolved in TBME (0.5 mL, **3b** will be 0.1 M). After 5 min at 25 °C, allyl ether **6a** (1.1 - 5 equiv) was added to the yellow solution and the reaction mixture was stirred for the reported time at 25 °C. Next, triethylamine (0.5 mL, 36 equiv) and, after 5 minutes, aryl bromide **3b** (0.1 mmol, 1 equiv) were added. The resulting yellow solution was stirred at 50 °C for 24 h. Once the temperature had reached 25 °C, the heterogeneous solution was filtered over a pad of Celite® and washed with Et_2O (5 mL). The solvent was removed under reduced pressure, the conversion, regioisomeric and stereoisomeric ratio of products **4ab** and **5ab** were assessed by ^1H NMR analysis of the crude reaction mixture in presence of an internal standard.

General Procedure X (GP X)

In a 5 mL J-Young tube, **C1** (8.2 mg, 0.015 mmol, 1 mol% to **6b** - 5 mol% to **2**) was dissolved in 2-MeTHF (3 mL, **2** will be 0.1M). After 5 min at 25 °C, allyl butyl ether **6b** (171 mg, 1.5 mmol, 5 equiv) was added to the yellow solution and the reaction mixture was stirred at 25 °C for 16 h. Next, dppp (12.4 mg, 0.03 mmol, 10 mol% to **2**), NaOAc if needed (24.6 mg, 0.3 mmol, 1 equiv), Cy_2NMe (58.6 mg, 0.3 mmol, 1 equiv) and aryl triflate **2** (0.3 mmol 1 equiv) were added in sequence. The resulting yellow solution was stirred for 24 h at 100 °C.* Once the temperature had reached 25 °C, the resulting solution was diluted with Et_2O (10 mL), washed with a 10% wt% aqueous solution of NH_4Cl (3 × 10 mL) and with brine (20 mL). The organic solution was dried over Na_2SO_4, filtered, and the solvent removed under reduced pressure. The regioisomeric and stereoisomeric ratio were assessed by ^1H NMR analysis of the crude reaction mixture. The residue was purified by column chromatography (neutral alumina) to afford product **4**.

*For **4bn**, **4bo** and **4bp**: Once the temperature had reached 25 °C, the resulting solution was filtered, washed with Et_2O (15 mL) and the solvent removed under reduced pressure. The regioisomeric and stereoisomeric ratio were assessed by ^1H NMR analysis of the crude reaction mixture. The residue was purified by column chromatography (neutral alumina) to afford product **4**.

General Procedure XI (GP XI)

In a 5 mL J-Young tube, **C1** (8.2 mg, 0.015 mmol, 4.5 mol% to **6** - 5 mol% to **2b**) was dissolved in 2-MeTHF (3 mL, **2b** will be 0.1M). After 5 min at 25 °C, allyl ether **6** (1.1 equiv) was added to the yellow solution and the reaction mixture was stirred for 1 h at 25 °C. Next, dppp (12.4 mg, 0.03 mmol, 10 mol% to **2b**), NaOAc (24.6 mg, 0.3 mmol, 1 equiv), the organic base Cy_2NMe (58.6 mg, 0.3 mmol, 1 equiv) and aryl triflate **2b** (75.3 mg, 0.3 mmol 1 equiv) were added in sequence. The resulting yellow solution was stirred for 24 h at 100 °C. Once the temperature had reached 25 °C, the solution was diluted with Et_2O (10 mL), washed with a 10% wt% NH_4Cl_{aq} (3 × 10 mL) and with brine (20 mL). The organic phase was dried over Na_2SO_4, filtered, and the solvent removed under reduced pressure. The regioisomeric and stereoisomeric ratio were assessed by ^1H NMR analysis of the crude reaction mixture. The residue was purified by column chromatography to afford product **4**.

General Procedure XII (GP XII)

In a 5 mL J-Young tube, **C1** (8.2 mg, 0.015 mmol, 1 mol% to **6a** - 5 mol% to **3**) was dissolved in TBME (1.5 mL, **3** will be 0.1M). After 5 min at 25 °C, allyl benzyl ether **6a** (222 mg, 1.5 mmol, 5 equiv) was added to the yellow solution and the reaction mixture was stirred for 16 h at 25 °C. Next, trimethylamine (1.5 mL, 36 equiv) and aryl bromide **3** (0.3 mmol, 1 equiv) were added in sequence. The resulting yellow solution was stirred at 50 °C for 24 h. Once the temperature had reached 25 °C, the resulting heterogeneous solution was filtered over a pad of Celite® and washed with Et_2O (15 mL). The solvent was removed under reduced pressure and the regioisomeric and stereoisomeric ratio were assessed by ^1H NMR analysis of the crude reaction mixture. The residue was purified by two consecutive column chromatography ((a) SiO_2 containing 10 wt% $AgNO_3$[3] using pentane/Et_2O as eluent; (b) SiO_2, using pentane/Et_2O as eluent) to afford product **5**.

General Procedure XIII (GP XIII)

In a 5 mL J-Young tube, **C1** (8.2 mg, 0.015 mmol, 4.5 mol% to **6** - 5 mol% to **3b**) was dissolved in TBME (1.5 mL, **3b** will be 0.1M). After 5 min at 25 °C, allyl ether **6** (1.1 equiv) was added and the reaction mixture was stirred for 1 h at 25 °C. Next, the organic base triethylamine (1.5 mL, 36 equiv) and aryl bromide **3b** (0.3 mmol, 1 equiv) were added in sequence. The resulting yellow solution was stirred at 50 °C for 24 h. Once the temperature had reached 25 °C, the resulting heterogeneous solution was filtered over a pad of Celite® and washed with Et_2O (15 mL). The solvent was removed under reduced pressure and the regioisomeric and stereoisomeric ratio were assessed by ^1H NMR analysis of the crude reaction mixture. The residue was purified by two consecutive column chromatographies (1. SiO_2 containing 10 wt% $AgNO_3$[21] using pentane/Et_2O as eluent, if necessary; 2. SiO_2, using pentane/Et_2O as eluent) to afford the corresponding product **5**.

General Procedure XIV (GP XIV)

In a 5 mL J-Young tube, **C1** (16.4 mg, 0.03 mmol, 9 mol% to **6** - 10 mol% to **3b**) was dissolved in toluene (1.5 mL, **3b** will be 0.1M). After 5 min at 25 °C, alkenyl ether **6** (0.33 mmol, 1.1 equiv) was added and the reaction mixture was stirred at 120 °C for 1 h. Next, triethylamine (1.5 mL, 36 equiv) and aryl bromide **3b** (0.3 mmol, 1 equiv) were added in sequence. The resulting black solution was stirred at 50 °C for 24 h. Once the temperature had reached 25 °C, the resulting heterogeneous solution was filtered over a pad of Celite® and washed with Et_2O (15 mL). The resulting heterogeneous solution was filtered through a pad of Celite® and washed with Et_2O. The solvent was removed under reduced pressure and the regioisomeric and stereoisomeric ratio were assessed by ^1H NMR analysis of the crude reaction mixture. The residue was purified by column chromatography (SiO_2, pentane/Et_2O as eluent) to afford product **5**.

3. Preparation of aryl triflates 2

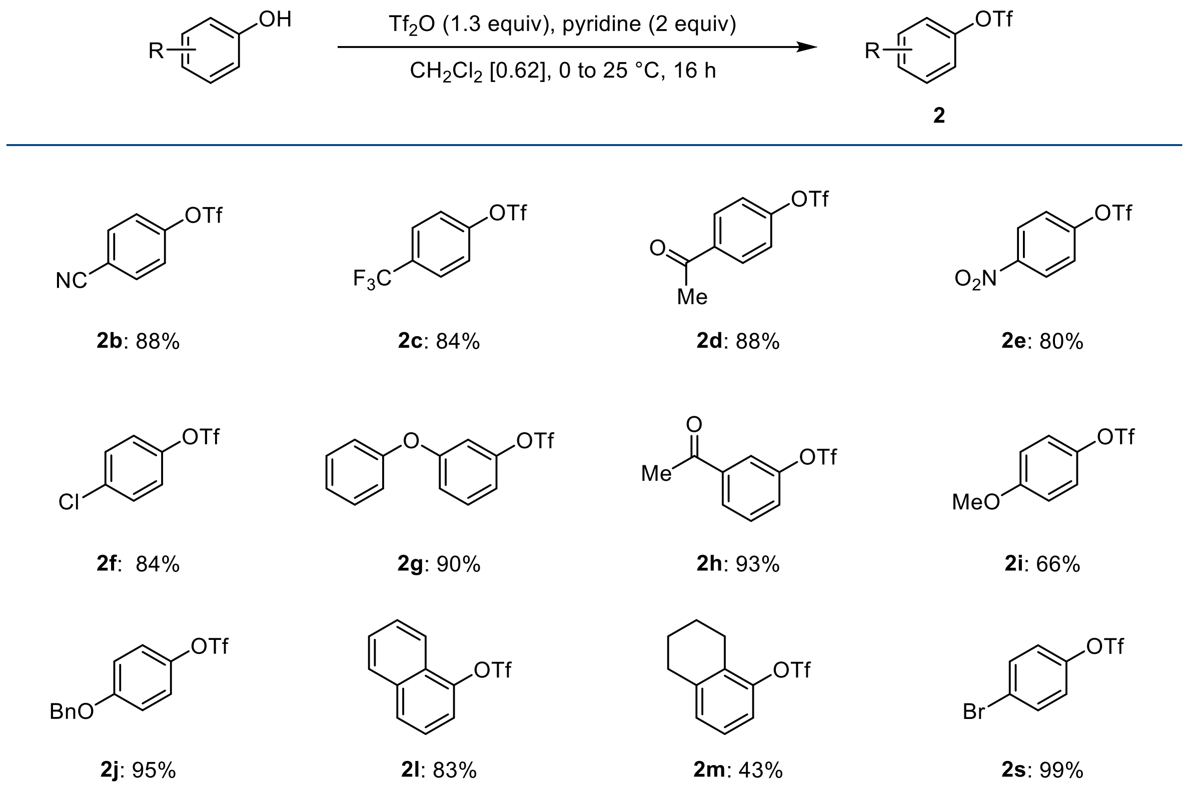

Figure S1. Scope of aryl triflates **2** prepared according to GP I.

4-cyanophenyl trifluoromethanesulfonate (2b)

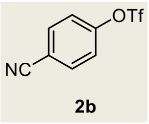

Following GP I using commercially available 4-hydroxybenzonitrile (476 mg, 4 mmol, 1.0), pyridine (625 mg, 8 mmol, 2.0 equiv) and trifluoromethanesulfonic anhydride (1.47 g, 5.2 mmol, 1.3 equiv). Purification by flash chromatography (pentane/Et_2O 90:10) afforded **2b** in pure form as a colorless oil (880 mg, 3.52 mmol, 88% yield). Spectroscopic data were in agreement with those reported in the literature.[4]

4-(trifluoromethyl)phenyl trifluoromethanesulfonate (2c)

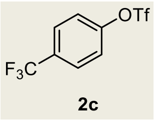

Following GP I using commercially available 4-trifluoromethylphenol (811 mg, 5 mmol, 1.0 equiv), pyridine (791 mg, 10 mmol, 2.0 equiv) and trifluoromethanesulfonic anhydride (1.83 g, 6.5 mmol, 1.3 equiv). Purification by flash chromatography (pentane/Et_2O 95:5) afforded **2c** in pure form as a colorless oil (1.23 g, 4.18 mmol, 84% yield). Spectroscopic data were in agreement with those reported in the literature.[1]

4-acetylphenyl trifluoromethanesulfonate (2d)

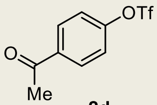

2d

Following GP I using commercially available 4-hydroxyacetophenone (272 mg, 2 mmol, 1.0 equiv), pyridine (313 mg, 4 mmol, 2.0 equiv) and trifluoromethanesulfonic anhydride (734 mg, 2.6 mmol, 1.3 equiv). Purification by flash chromatography (pentane/Et_2O 90:10) afforded **2d** in pure form as a colorless oil (470 mg, 1.76 mmol, 88% yield). Spectroscopic data were in agreement with those reported in the literature.[5]

4-nitrophenyl trifluoromethanesulfonate (2e)

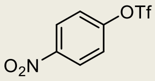

2e

Following GP I using commercially available 4-nitrophenol (279 mg, 2 mmol, 1.0 equiv), pyridine (313 mg, 4 mmol, 2.0 equiv) and trifluoromethanesulfonic anhydride (734 mg, 2.6 mmol, 1.3 equiv). Purification by flash chromatography (pentane/Et_2O 95:5) afforded **2e** in pure form as a white solid (436 mg, 1.6 mmol, 80% yield). Spectroscopic data were in agreement with those reported in the literature.[5]

4-chlorophenyl trifluoromethanesulfonate (2f)

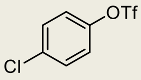

2f

Following GP I using commercially available 4-chlorophenol (257 mg, 2 mmol, 1.0 equiv), pyridine (313 mg, 4 mmol, 2.0 equiv) and trifluoromethanesulfonic anhydride (734 mg, 2.6 mmol, 1.3 equiv). Purification by flash chromatography (pure pentane) afforded **2f** in pure form as a colorless oil (436 mg, 1.68 mmol, 84 % yield). Spectroscopic data were in agreement with those reported in the literature.[4]

3-phenoxyphenyl trifluoromethanesulfonate (2g)

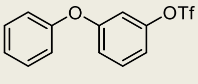

2g

Following GP I using commercially available 3-phenoxyphenol (500 mg, 2.7 mmol, 1.0 equiv), pyridine (419 mg, 5.4 mmol, 2.0 equiv) and trifluoromethanesulfonic anhydride (985 mg, 3.5 mmol, 1.3 equiv). Purification by flash chromatography (pentane/Et_2O 96:4) afforded **2g** in pure form as a colorless oil (772 mg, 2.43 mmol, 90% yield). Spectroscopic data were in agreement with those reported in the literature.[6]

3-acetylphenyl trifluoromethanesulfonate (2h)

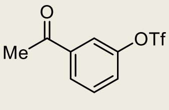

2h

Following GP I using commercially available 3-hydroxyacetophenone (273 mg, 2 mmol, 1.0 equiv), pyridine (313 mg, 4 mmol, 2.0 equiv) and trifluoromethanesulfonic anhydride (734 mg, 2.6 mmol, 1.3 equiv). Purification by flash chromatography (pentane/Et_2O 90:10) afforded **2h** in pure form as a colorless oil (537 mg, 1.86 mmol, 93% yield). Spectroscopic data were in agreement with those reported in the literature.[7]

4-methoxyphenyl trifluoromethanesulfonate (2i)

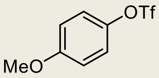

2i

Following GP I using commercially available 4-methoxyphenol (621 mg, 5 mmol, 1.0 equiv), pyridine (791 mg, 10 mmol, 2.0 equiv) and trifluoromethanesulfonic anhydride (1.83 g, 6.5 mmol, 1.3 equiv). Purification by flash chromatography (pentane/Et_2O 90:10) afforded **2i** in pure form as a colorless oil (840 mg, 3.28 mmol, 66% yield). Spectroscopic data were in agreement with those reported in the literature.[8]

4-(benzyloxy)phenyl trifluoromethanesulfonate (2j)

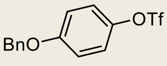

2j

Following GP I using commercially available 4-benzyloxyphenol (401 mg, 2 mmol, 1.0 equiv), pyridine (313 mg, 4 mmol, 2.0 equiv) and trifluoromethanesulfonic anhydride (734 mg, 2.6 mmol, 1.3 equiv). Purification by flash chromatography (pentane/Et_2O 96:4) afforded **2j** in pure form as a colorless oil (635 mg, 1.90 mmol, 95% yield). Spectroscopic data were in agreement with those reported in the literature.[9]

Naphthalen-1-yl trifluoromethanesulfonate (2l)

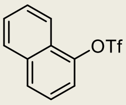

2l

Following GP I using commercially available 1-naphtol (288 mg, 2 mmol, 1.0 equiv), pyridine (313 mg, 4 mmol, 2.0 equiv) and trifluoromethanesulfonic anhydride (734 mg, 2.6 mmol, 1.3 equiv). Purification by flash chromatography (pentane/Et_2O 95:5) afforded **2l** in pure form as a colorless oil (461 mg, 1.66 mmol, 83% yield). Spectroscopic data were in agreement with those reported in the literature.[8]

5,6,7,8-tetrahydronaphthalen-1-yl trifluoromethanesulfonate (2m)

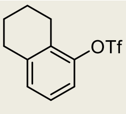

2m

Following GP I using commercially available 5,6,7,8-tetrahydro-1-naphtol (297 mg, 2 mmol, 1.0 equiv), pyridine (313 mg, 4 mmol, 2.0 equiv) and trifluoromethanesulfonic anhydride (734 mg, 2.6 mmol, 1.3 equiv). Purification by flash chromatography (pure pentane) afforded **2m** in pure form as a colorless oil (250 mg, 1.10 mmol, 43% yield). Spectroscopic data were in agreement with those reported in the literature.[10]

4-bromophenyl trifluoromethanesulfonate (2s)

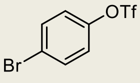

2s

Following GP I using commercially available 4-bromophenol (346 mg, 2 mmol, 1.0 equiv), pyridine (313 mg, 4 mmol, 2.0 equiv) and trifluoromethanesulfonic anhydride (734 mg, 2,6 mmol, 1.3 equiv). Purification by flash chromatography (pentane/Et_2O 95:05) afforded **2s** in pure form as a colorless oil (603 mg, 1.99 mmol, 99% yield). Spectroscopic data were in agreement with those reported in the literature.[11]

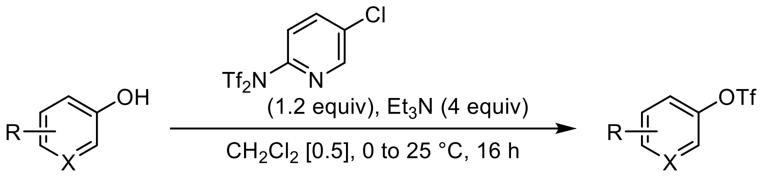

2

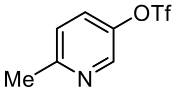

2n: 98%

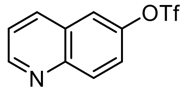

2o: 89%

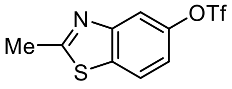

2p: 82%

Figure S2. Scope of aryl triflates **2** prepared according to GP II.

6-methylpyridin-3-yl trifluoromethanesulfonate (2n)

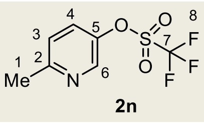

Following GP II using commercially available 5-hydroxy-2-methylpiridine (218 mg, 2 mmol, 1.0 equiv), *N*-(5-Chloro-2-pyridyl)bis(trifluoromethanesulfonimide) (942 mg, 2.4 mmol, 1.2 equiv) and triethylamine (810 mg, 8 mmol, 4.0 equiv). Purification by flash chromatography (pentane/Et_2O 94:6) afforded **2n** in pure form as a colorless oil (473 mg, 1.96 mmol, 98% yield).

TLC: pentane/Et_2O 94:6, R_f = 0.3.

^{1}H NMR (300 MHz, $CDCl_3$) δ (ppm) = 8.49 (d, $^4J_{HH}$ = 2.9 Hz, 1H, H-6), 7.53 (dd, $^3J_{HH}$ = 8.6 Hz, $^4J_{HH}$ = 2.9 Hz, 1H, H-4), 7.27 (d, $^3J_{HH}$ = 8.6 Hz, 1H, H-3), 2.62 (s, 3H, H-1).

^{13}C{^{1}H} NMR (75 MHz, $CDCl_3$) δ (ppm) = 159.2 (C-2), 145.1 (C-5), 142.0 (CH-6), 129.3 (CH-4), 124.4 (CH-3), 118.9 (q, $^1J_{CF}$ = 320 Hz, CF_3-7), 24.1 (CH_3-1).

^{19}F{^{1}H} NMR (282 MHz, $CDCl_3$) δ (ppm) = –72.6 (s, F-8).

HRMS (ESI +): calculated for $C_7H_7F_3NO_3S$ $[M+H]^+$: 242.0099; found: 242.0104.

IR (neat): ν (cm^{-1}) = 1596, 1481, 1424, 1206, 1136, 1020, 877, 707, 609.

Quinolin-6-yl trifluoromethanesulfonate (2o)

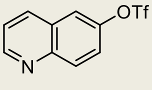

2o

Following GP II using commercially available 6-hydroxyquinoline (290 mg, 2 mmol, 1.0 equiv), *N*-(5-Chloro-2-pyridyl)bis(trifluoromethanesulfonimide) (942 mg, 2.4 mmol, 1.2 equiv) and triethylamine (810 mg, 8 mmol, 4.0 equiv). Purification by flash chromatography (pentane/Et_2O 94:6) afforded **2o** in pure form as a colorless oil (492 mg, 1.78 mmol, 89% yield). Spectroscopic data were in agreement with those reported in the literature.[8]

2-methylbenzo[*d*]thiazol-5-yl trifluoromethanesulfonate (2p)

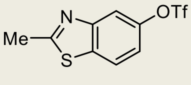

2p

Following GP II using commercially available 2-methylbenzo[d]thiazol-5-ol (330 mg, 2 mmol, 1.0 equiv), *N*-(5-Chloro-2-pyridyl)bis(trifluoromethanesulfonimide) (942 mg, 2.4 mmol, 1.2 equiv) and triethylamine (810 mg, 8 mmol, 4.0 equiv). Purification by flash chromatography (pentane/Et_2O 94:6) afforded **2p** in pure form as a white solid (490 mg, 1.64 mmol, 82% yield). Spectroscopic data were in agreement with those reported in the literature.[12]

4. Preparation of vinyl ethers 1 and alkenyl ethers 6

4.1. Synthesis of vinyl ether (*Z*)-1a

Into a flame-dried flask benzyl allyl ether **6a** (1.48 g, 10 mmol, 1 equiv), *t*-BuOK (1.68 g, 15 mmol, 1.5 equiv) were dissolved in THF (25 mL, 0.4 M). The reaction mixture was heated at 100 °C for 16 h. After reaction completion (monitored by TLC), water (20 mL) was added and the mixture was extracted with Et_2O (20 mL × 3). The organic phases were collected, dried over Na_2SO_4, filtered and concentrated under vacuum. The residue was purified by flash chromatography (pentane/Et_2O 99:1) on silica gel to afford the (*Z*)-((prop-1-en-1-yloxy)methyl)benzene (*Z*)-**1a** (1.33 g, 9.0 mmol, 90% yield). Spectroscopic data were in agreement with those reported in the literature.[13]

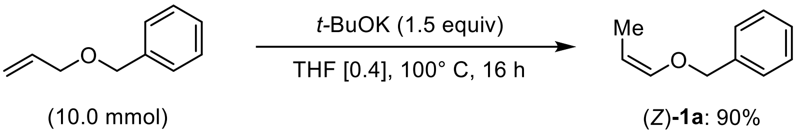

Figure S3. Synthesis of (*Z*)-((prop-1-en-1-yloxy)methyl)benzene (*Z*)-**1a**.

4.2. Synthesis of vinyl ethers 1a and 1b

Entry 1: Adapted from GP VI using catalyst **C1** (91.1 mg, 0.167 mmol, 5 mol%), allyl benzyl ether **6a** (494 mg, 3.3 mmol, 1 equiv) dissolved in THF (33.3 mL, **6a** is 0.1 M) and. The residue was purified by column chromatography (silica gel) using pentane/Et_2O (98/2) as eluent to afford **1a** in pure form as a colorless oil (434 mg, 2.93 mmol, 88% yield, *E/Z* 40:60).

Entry 2: Adapted from GP VI using catalyst **C1** (91.1 mg, 0.167 mmol, 5 mol%) and allyl butyl ether **6b** (380 mg, 3.3 mmol, 1 equiv) in neat conditions. The residue was distilled to afford **1b** in pure form as a colorless oil (281 mg, 2.46 mmol, 74% yield, *E/Z* 40:60).

Table S1. Synthesis of *E/Z* mixture of **1a** and **1b**.

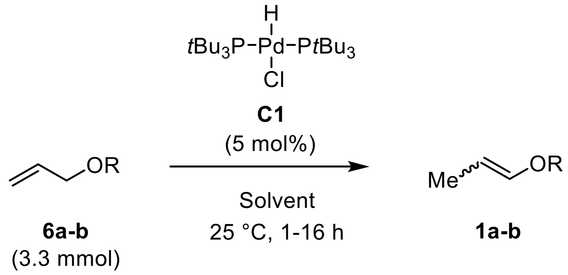

Entry	Solvent	t (h)	Yield **1a-b** (%)	E/Z[a]
1	THF	1	88	40:60
2	neat	16	74	40:60

[a] Stereoisomeric ratio determined by ^{1}H NMR of the crude reaction mixture using an internal standard.

4.3. Synthesis of alkenyl ethers 6c-h

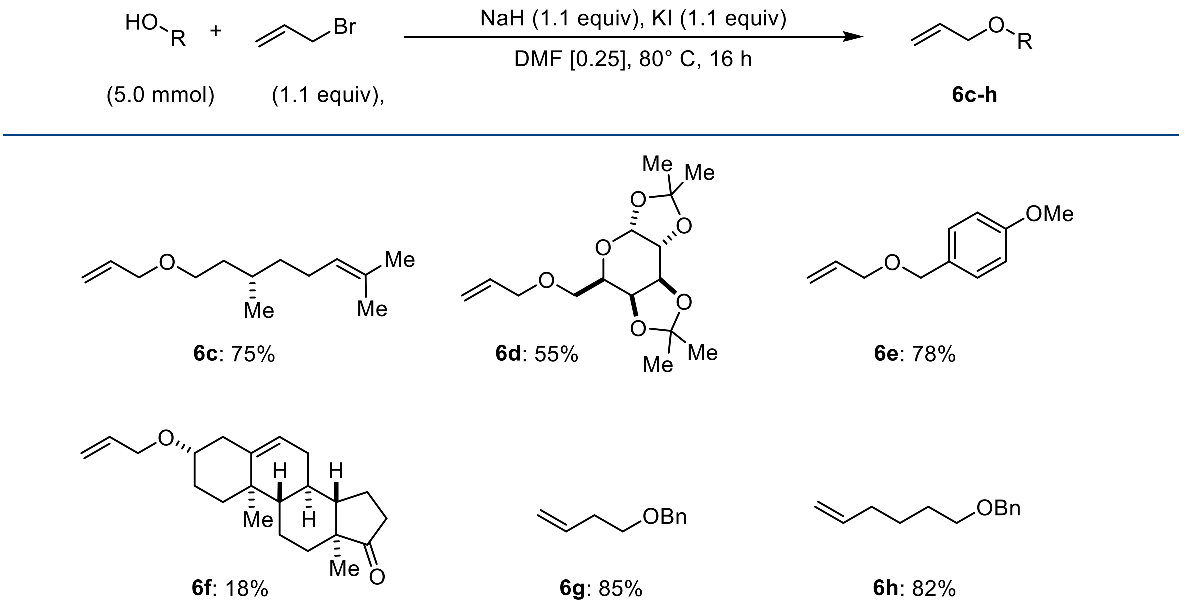

Figure S4. Synthesis of alkenyl ethers **6c-h**.

(*S*)-8-(allyloxy)-2,6-dimethyloct-2-ene (6c)

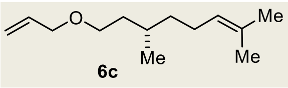

Following GP III using NaH (220 mg, 5.5 mmol, 1.1 equiv), commercially available (*S*)-(−)-β-Citronellol (781 mg, 5 mmol, 1.0 equiv), allyl bromide (665 mg, 5.5 mmol, 1.1 equiv) and KI (913 mg, 5.5 mmol, 1.1 equiv). Purification by flash chromatography (pentane/Et_2O 95:5) afforded **6c** in pure form as a colorless oil (736 mg, 3.75 mmol, 75% yield). Spectroscopic data were in agreement with those reported in the literature.[14]

(3a*R*,5*R*,5a*S*,8a*S*,8b*R*)-5-((allyloxy)methyl)-2,2,7,7-tetramethyltetrahydro-5*H*-bis([1,3]dioxolo)[4,5-*b*:4',5'-*d*]pyran (6d)

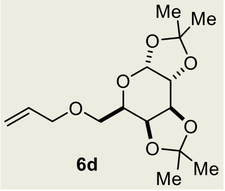

Following GP III using NaH (220 mg, 5.5 mmol, 1.1 equiv), commercially available 1,2:3,4-Di-*O*-isopropylidene-α-D-galactopyranose (1.30 g, 5 mmol, 1.0 equiv), allyl bromide (665 mg, 5.5 mmol, 1.1 equiv) and KI (913 mg, 5.5 mmol, 1.1 equiv). Purification by flash chromatography (pentane/Et_2O 90:10) afforded **6d** in pure form as a viscous colorless oil (826 mg, 2.75 mmol, 55% yield). Spectroscopic data were in agreement with those reported in the literature.[15]

1-((allyloxy)methyl)-4-methoxybenzene (6e)

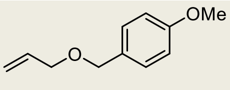

6e

Following GP III using NaH (220 mg, 5.5 mmol, 1.1 equiv), commercially available 4-methoxybenzylacohol (691 mg, 5 mmol, 1.0 equiv), allyl bromide (665 mg, 5.5 mmol, 1.1 equiv) and KI (913 mg, 5.5 mmol, 1.1 equiv). Purification by flash chromatography (pentane/Et_2O 95:5) afforded **6e** in pure form as a colorless oil (695 mg, 3.90 mmol, 78% yield). Spectroscopic data were in agreement with those reported in the literature.[16]

(3*S*,8*R*,9*S*,10*R*,13*S*,14*S*)-3-(allyloxy)-10,13-dimethyl-1,2,3,4,7,8,9,10,11,12,13,14,15,16-tetradecahydro-17*H*-cyclopenta[*a*]phenanthren-17-one (6f)

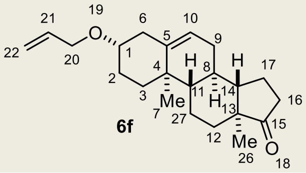

6f

Following GP III using NaH (220 mg, 5.5 mmol, 1.1 equiv), commercially available dehydroepiandrosterone (1.44 g, 5 mmol, 1.0 equiv), allyl bromide (665 mg, 5.5 mmol, 1.1 equiv) and KI (913 mg, 5.5 mmol, 1.1 equiv). Purification by flash chromatography (gradient from pentane/Et_2O 95:5 to 70:30) afforded **6f** in pure form as a white solid (296 mg, 0.9 mmol, 18% yield).

TLC: pentane/Et_2O 80:20, R_f = 0.3.

^{1}H NMR (400 MHz, $CDCl_3$) δ (ppm) = 5.93 (ddt, $^3J_{HH}$ = 17.2, 10.4, 5.6 Hz, 1H, H-21), 5.38 (dt, $^3J_{HH}$ = 5.1, 1.9 Hz, 1H, H-10), 5.27 (dq, $^3J_{HH}$ = 17.2 Hz, $^2J_{HH}$ = 1.7 Hz, 1H, H-22), 5.16 (dq, $^3J_{HH}$ = 10.3 Hz, $^2J_{HH}$ = 1.4 Hz, 1H, H-22), 4.03 (dt, $^3J_{HH}$ = 5.6 Hz, $^2J_{HH}$ = 1.4 Hz, 2H, H-20), 3.21 (tt, $^3J_{HH}$ = 11.3, 4.5 Hz, 1H, H-1), 2.52 – 2.42 (m, 1H, H-16), 2.42 – 2.36 (m, 1H, H-6), 2.30 – 2.18 (m, 1H, H-6), 2.15 – 2.02 (m, 2H, H-9 and H-16), 2.00 – 1.80 (m, 4H, H-2 and H-3 and H-12 and H-17), 1.73 – 1.61 (m, 3H, H-27, H-9 and H-8), 1.56 (s, 2H), 1.54 – 1.41 (m, 3H, H-2 and H-12 and H-27), 1.36 – 1.21 (m, 2H, H-14 and H-17), 1.10 – 0.96 (m, 2H, H-11 and H-3), 1.03 (s, 3H, H-7), 0.89 (s, 3H, H-26).

^{13}C{^{1}H} NMR (101 MHz, $CDCl_3$) δ (ppm) = 221.3 (C-15), 141.4 (C-5), 135.6 (CH-21), 120.9 (CH-10), 116.7 (CH_2-22), 78.5 (CH-1), 69.2 (CH_2-20), 51.9 (CH-14), 50.5 (CH-11), 47.7 (C-13), 39.3 (CH_2-6), 37.3 (C-4), 37.3 (CH_2-3), 36.0 (CH_2-16), 31.7 (CH-8), 31.6 (CH_2-12), 31.0 (CH_2-9), 28.5 (CH_2-2), 22.0 (CH_2-17), 20.5 (CH_2-27), 19.5 (CH_3-7), 13.7 (CH_3-26).

HRMS (ESI +): calculated for $C_{22}H_{32}O_2$ $[M+NH_4]^+$: 346.2741; found: 346.2738.

IR (neat): ν (cm^{-1}) = 2939, 2833, 1731, 1452, 1411, 1375, 1137, 1075, 912, 843.

m.p.: 88-90 °C.

((but-3-en-1-yloxy)methyl)benzene (6g)

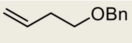

6g

Following GP III using NaH (220 mg, 5.5 mmol, 1.1 equiv), commercially available 3-buten-1-ol (361 mg, 5 mmol, 1.0 equiv), benzyl bromide (665 mg, 5.5 mmol, 1.1 equiv) and KI (913 mg, 5.5 mmol, 1.1 equiv). Purification by flash chromatography (pentane/Et_2O 95:5) afforded **6g** in pure form as a colorless oil (689 mg, 4.25 mmol, 85% yield). Spectroscopic data were in agreement with those reported in the literature.[17]

((hex-5-en-1-yloxy)methyl)benzene (6h)

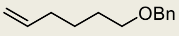

6h

Following GP III using NaH (220 mg, 5.5 mmol, 1.1 equiv), commercially available hex-5-en-1-ol (501 mg, 5 mmol, 1.0 equiv), benzyl bromide (665 mg, 5.5 mmol, 1.1 equiv) and KI (913 mg, 5.5 mmol, 1.1 equiv). Purification by flash chromatography (pentane/Et_2O 95:5) afforded **6h** in pure form as a colorless oil (780 mg, 4.10 mmol, 82% yield). Spectroscopic data were in agreement with those reported in the literature.[18]

5. Preparation of C1 and C2

Into a 50 mL flask, the bis(tri-*t*-butylphosphine)palladium(0) (1.02 g, 2.00 mmol, 1.0 equiv) was dissolved in THF (20 mL, 0.1 M). A 1N solution of HCl in Et_2O (2.2 mL, 2.20 mmol, 1.1 equiv) was added slowly over a 5-minute period and stirred at 25 °C for 1 h. The volatiles were removed under high vacuum and the solid was recovered and washed with pentane (3 × 1 mL) over a sintered glass funnel. The solid was dried overnight under vacuum affording **C1** (822 mg, 1.5 mmol, 75% yield). Spectroscopic data were in agreement with those reported in the literature.[19]

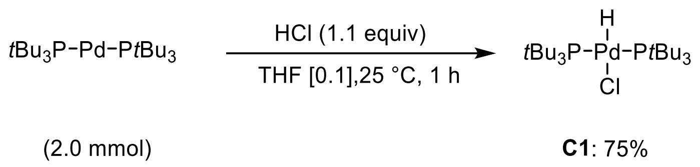

Figure S5. Synthesis of **C1**.

A solution of dppp (206 mg, 0.5 mmol, 1 equiv) in CH_2Cl_2 (7 mL) was added dropwise into a flask containing [(COD)PdMeCl] (133 mg, 0.5 mmol, 1 equiv) in CH_2Cl_2 (8 mL) and stirred at 25 °C for 2 h. Next, the volume was reduced to ca. 1 mL, and layered with pentane (5 mL). The solution was cooled to –20 °C for 2 h. The precipitate thus formed was filtered over a sintered glass funnel and washed with pentane (3 × 5.0 mL). The solid was dried overnight under vacuum affording **C2** (140 mg, 0.245 mmol, 49% yield). Spectroscopic data were in agreement with those reported in the literature.[20]

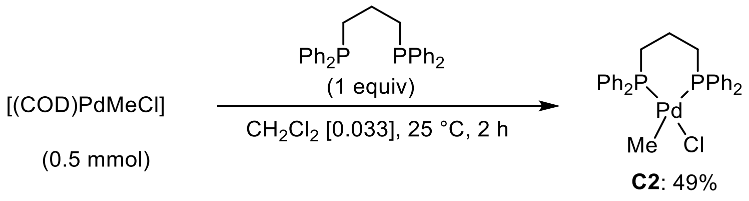

Figure S6. Synthesis of **C2**.

6. Heck reaction optimization

6.1. α-regioselectivity

Following GP IV:

Table S2. Optimization of the Heck reaction – α-selectivity.

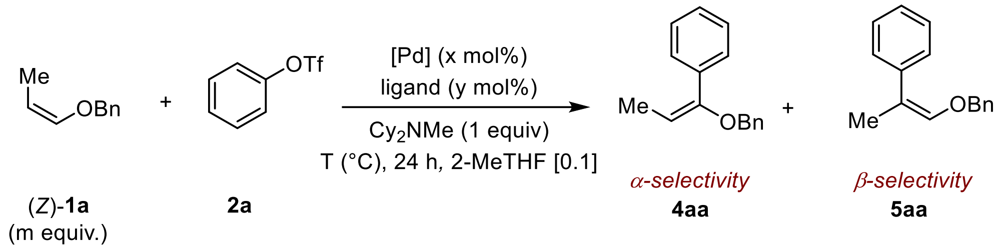

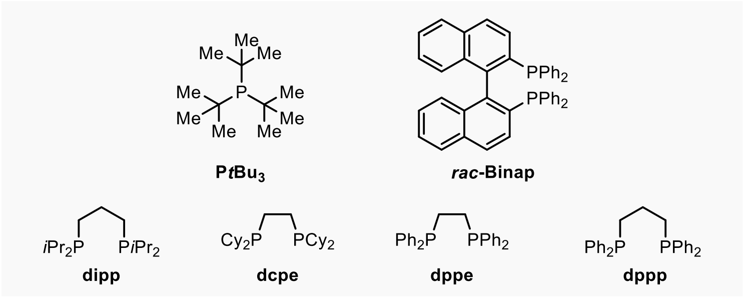

Entry	[Pd] (x)	Ligand (y)	(*Z*)-**1a** (m)	*Conv.* (%)[a]	E/Z[a]	$rr_{\alpha/\beta}$[a]
1	$Pd(OAc)_2$ (5)	-	5	<5	nd	nd
2	$Pd(OAc)_2$ (5)	P*t*Bu₃ (10)	5	<5	nd	nd
3	Pd(P*t*Bu₃)₂ (5)	-	5	<5	nd	nd
4	$Pd(OAc)_2$ (5)	dipp (10)	5	<5	nd	nd
5	$Pd(OAc)_2$ (5)	dcpe (10)	5	<5	nd	nd
6	$Pd(OAc)_2$ (5)	dppe (10)	5	18	74:26	>20:1
7	$Pd(OAc)_2$ (5)	*rac*-Binap (10)	5	29	86:14	>20:1
8	$Pd(OAc)_2$ (5)	dppp (10)	5	90	52:48	>20:1
9	$Pd(OAc)_2$ (5)	dppp (10)	3	80	50:50	>20:1
10	$Pd(OAc)_2$ (5)	dppp (10)	1.1	73	50:50	>20:1
11	$Pd(OAc)_2$ (1)	dppp (2)	1.1	69	40:60	>20:1
12[b]	$Pd(OAc)_2$ (5)	dppp (10)	5	86	55:45	>20:1
13	$Pd(OAc)_2$ (5)	dppp (10)	5[c]	87	48:52	>20:1

Reaction performed on a 0.1 mmol scale. [a] Conversion, stereoisomeric and regioisomeric ratio determined by ^{1}H NMR using an internal standard. [b] Cy_2NMe (19 equiv). [c] **1a** (*E/Z* 40:60).

6.2. β-regioselectivity

Following GP V:

Table S3. Optimization of the Heck reaction – β-selectivity.

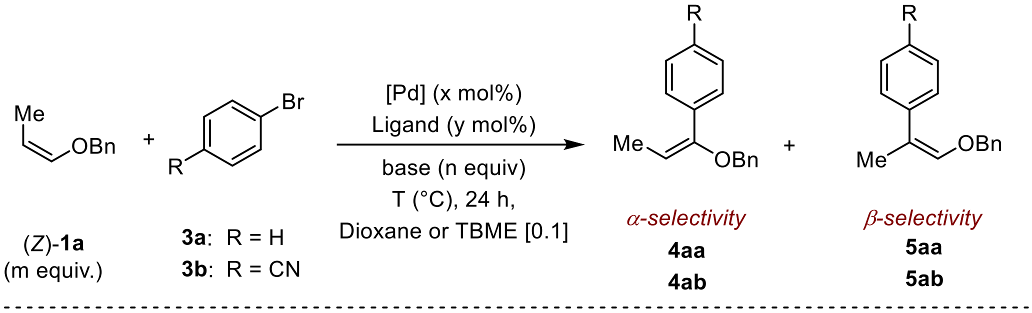

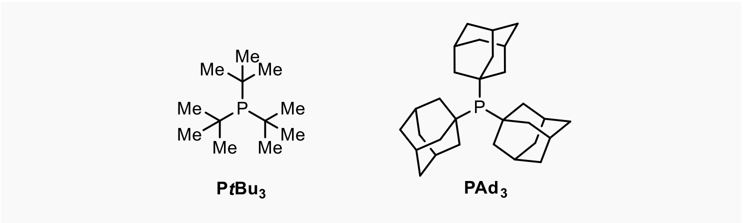

Entry	[Pd] (x)	Ligand (y)	Base (n)	*Conv.* (%)[a]	*E*/*Z*[a]	$rr_{\alpha/\beta}$[a]
1[b]	$Pd_2(dba)_3$ (2.5)	P*t*Bu3 (10)	Cy_2NMe (1)	54	50:50	1:1
2	$Pd_2(dba)_3$ (2.5)	P*t*Bu3 (10)	Cy_2NMe (1)	88	50:50	1:3.2
3	$Pd_2(dba)_3$ (2.5)	PAd_3 (10)	Cy_2NMe (1)	63	50:50	1:3.8
4	$Pd(OAc)_2$ (5)	P*t*Bu3 (10)	Cy_2NMe (1)	<5	nd	nd
5	$Pd(PtBu_3)_2$ (5)	-	Cy_2NMe (1)	83	50:50	1:3.8
6	$Pd(PtBu_3)_2$ (5)	-	Cy_2NMe (7)	75	25:75	1:4.8
7	$Pd(PtBu_3)_2$ (5)	-	Et_3N (7)	74	12:88	1:4.7
8	$Pd(PtBu_3)_2$ (5)	-	Et_3N (36)	91	4:96	1:5.9
9[c]	$Pd(PtBu_3)_2$ (5)	-	Et_3N (36)	<5	nd	nd
10	$Pd(PtBu_3)_2$ (5)	-	Et_3N (36)	91	4:96	1:8.1
11[d]	$Pd(PtBu_3)_2$ (5)	-	Et_3N (36)	91	4:96	1:7.2
12[c]	$Pd(PtBu_3)_2$ (5)	-	Et_3N (36)	26	3:97	1:5.8
13[e]	$Pd(PtBu_3)_2$ (5)	-	Et_3N (36)	73	43:57	1:10
14	$PdHCl(PtBu_3)_2$ (5)	-	Et_3N (36)	82	4:96	1:7.2
15[e]	$PdHCl(PtBu_3)_2$ (5)	-	Et_3N (36)	76	50:50	>1:20
16[e,f]	$Pd(PtBu_3)_2$ (5)	-	Et_3N (36)	82	45:55	1:11.6

Reaction performed on a 0.1 mmol scale with **3b** as substrate. Entries 1-9 using dioxane as solvent, Entries 10-15 using TBME as solvent. [a] Conversion, stereoisomeric and regioisomeric ratio determined by ^{1}H NMR using an internal standard. [b] **3a** was used instead of **3b**. [c] Reaction performed at 25 °C. [d] Reaction performed at 40 °C. [e] **1a** (*E*/*Z* 40:60). [f] Addition of Et_3NHCl (5 mol%).

7. Optimization of the isomerization reaction

Following GP VI:

Table S4. Optimization of the isomerization reaction of **6a** with **C1**.

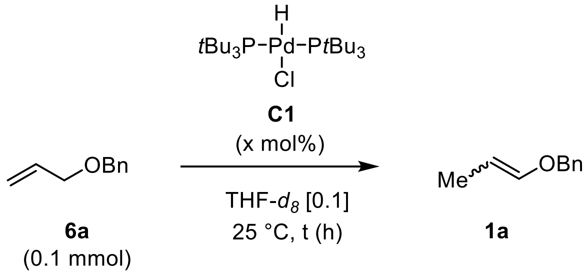

Entry	**C1** (x mol%)	t (h)	*Conv.* **1a** (%)[a]	E/Z[a]
1	5	1	99	40:60
2	4	6	99	40:60
3	1	16	99	40:60

Reaction performed on a 0.1 mmol scale. The time shown in the table corresponds to the minimum reaction time to get 99% conv. into **1a**. [a] Conversion and stereoisomeric ratio were monitored and determined by ^{1}H NMR using an internal standard.

Following GP VII:

Table S5. Optimization of the isomerization reaction of **6a** with **C2**.

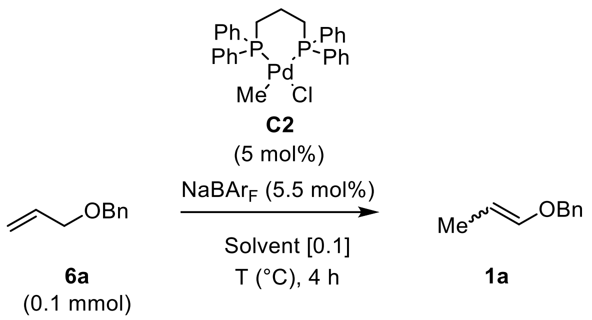

Entry	Solvent	T (°C)	*Conv.* **1a** (%)[a]	E/Z[a]
1	2-MeTHF	25	-	-
2	2-MeTHF	120	<10	nd
3	DME	25	-	-
4	DME	120	<10	nd
5	CH_2Cl_2	25	99	30:70

Reaction performed on a 0.1 mmol scale. [a] Conversion and stereoisomeric ratio determined by ^{1}H NMR using an internal standard.

Following GP VII:

Table S6. Optimization of the isomerization reaction of **6h** with **C2**.

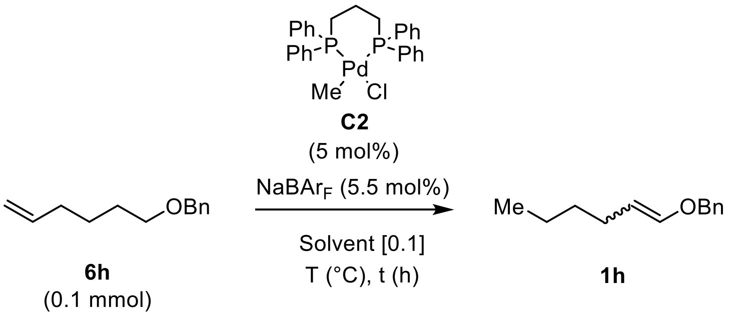

Entry	Solvent	T (°C)	t (h)	*Conv.* **1a** (%)[a]	E/Z[a]
1	2-MeTHF	120	4	<10	nd
2	DME	120	4	<10	nd
3	Toluene	120	4	30	50:50
4	CH_2Cl_2	25	18	68	50:50
5	CH_2Cl_2	60	2	71	50:50

Reaction performed on a 0.1 mmol scale. [a] Conversion and stereoisomeric ratio determined by ^{1}H NMR using an internal standard.

8. Assisted tandem (AT) reaction

8.1. Optimization – α-regioselectivity

Following GP VIII:

Table S7. Optimization of the AT isomerization/Heck reaction – α selectivity.

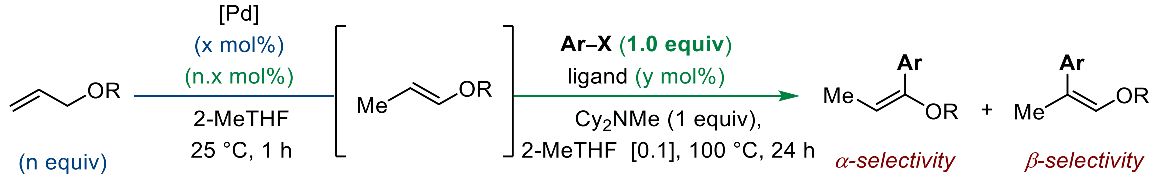

6a R = Bn
6b R = *n*-Bu

1a R = Bn
1b R = *n*-Bu

4aa, 4ab, 4ba **5aa, 5ab, 5ba**

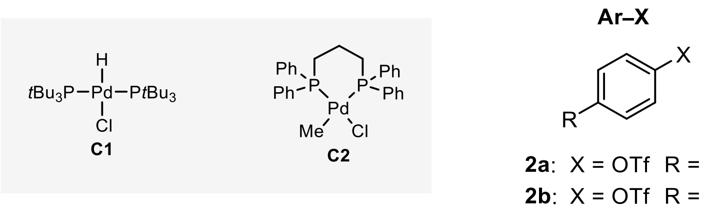

2a: X = OTf R = H
2b: X = OTf R = CN

Entry	**6** (n)	Cat. (x/n.x)	Ar-X	Ligand (y)	Conv. (%)[a]	E/Z[a]	$rr_{\alpha/\beta}$[a]
1	**6a** (1.1)	**C1** (4.5/5.0)	**2a**	-	<5	nd	nd
2	**6a** (1.1)	**C2** (4.5/5.0)[b]	**2a**	-	<5	nd	nd
3	**6a** (1.1)	**C1** (4.5/5.0)	**2a**	dppp (10)	50	50:50	>20:1
4	**6b** (1.1)	**C1** (4.5/5.0)	**2a**	dppp (10)	60	50:50	>20:1
5	**6b** (1.1)	**C1** (4.5/5.0)	**2a**	dppp (5)	45	50:50	>20:1
6	**6b**[c] (5.0)	**C1** (1.0/5.0)	**2a**	dppp (10)	70 (77)	48:52	>20:1
7	**6b**[c] (5.0)	**C1** (1.0/5.0)	**2b**	dppp (10)	<5	nd	nd
8	**6b**[c] (5.0)	**C1** (1.0/5.0)	**2b**	dppp (10)[d]	87 (73)	62:38	>20:1
9	**6b**[c] (5.0)	**C1** (1.0/5.0)	**2b**	dppp (10)[e]	<5	nd	nd

Reaction performed on a 0.1 mmol scale. [a] Conversion, stereoisomeric and regioisomeric ratio determined by ^{1}H NMR using an internal standard. In parenthesis, yield after purification. [b] Precatalyst activation with $NaBAr_F$ (5.5 mol%). [c] The isomerization step required 16 h. [d] Addition of NaOAc (1 equiv). [e] Addition of nBu_4NBF_4 (1 equiv).

8.2. Optimization – β-regioselectivity

Following GP IX:

Table S8. Optimization of the AT isomerization/Heck reaction – β selectivity.

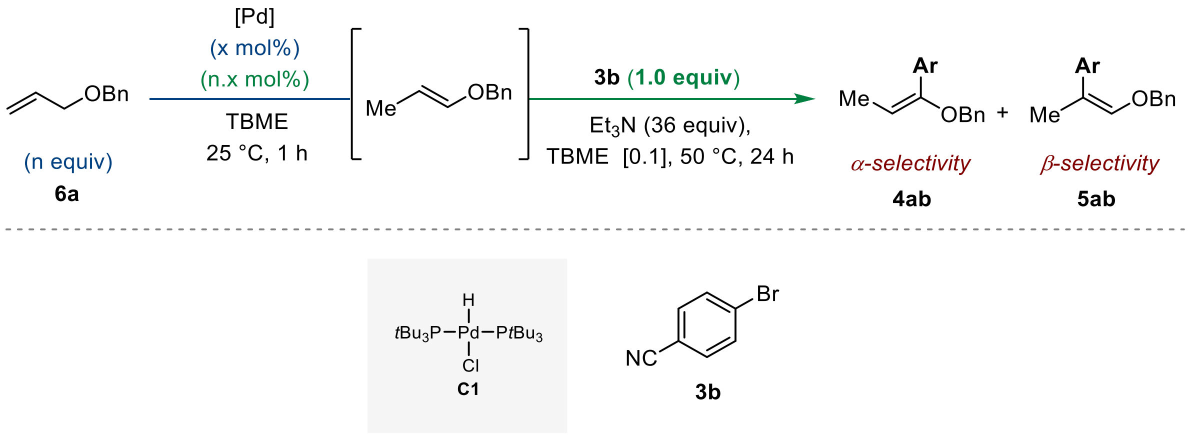

Entry	Cat. (x/n.x)	**6** (n)	Conv. (%)[a]	E/Z[a]	*rr*$_{\alpha/\beta}$[a]
1	**C1** (4.5/5.0)	**6a** (1.1)	55 (46)	45:55	>1:20
2	**C1** (1.0/5.0)	**6a** (5.0)[b]	76 (70)	47:53	>1:20

Reaction performed on a 0.1 mmol scale. [a] Conversion, stereoisomeric and regioisomeric ratio determined by ^{1}H NMR using an internal standard. In parenthesis, yield after purification. [b] The isomerization step required 16 h.

8.3. Scope of AT reaction – α-regioselectivity

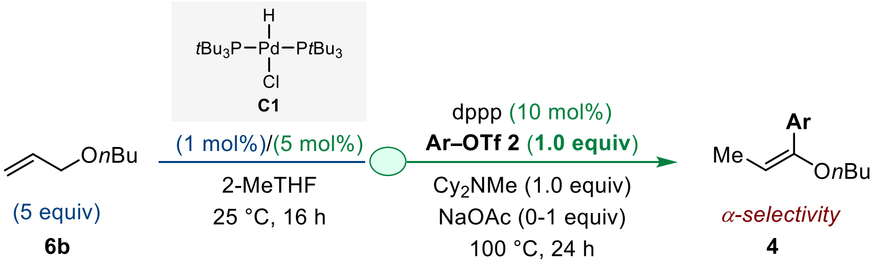

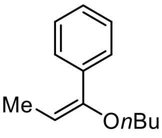

4ba: 77% yield
E/Z 48:52; $rr_{\alpha/\beta}$ >20:1

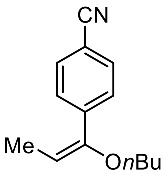

4bb: 73% yield
60% (*gram scale*)
E/Z 62:38; $rr_{\alpha/\beta}$ >20:1

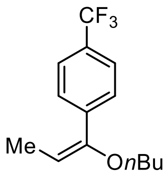

4bc: 54% yield
E/Z 63:37; $rr_{\alpha/\beta}$ >20:1

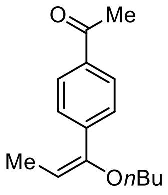

4bd: 65% yield
E/Z 60:40; $rr_{\alpha/\beta}$ >20:1

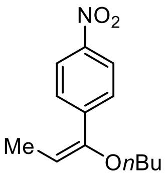

4be: 46% yield
E/Z 58:42; $rr_{\alpha/\beta}$ >20:1

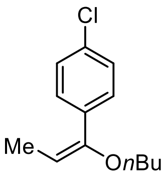

4bf: 75% yield
E/Z 60:40; $rr_{\alpha/\beta}$ >20:1

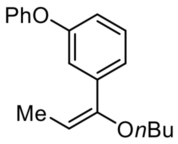

4bg: 50% yield
E/Z 50:50; $rr_{\alpha/\beta}$ >20:1

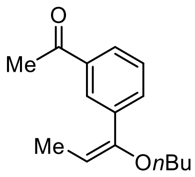

4bh: 70% yield
E/Z 55:45; $rr_{\alpha/\beta}$ >20:1

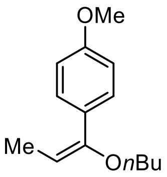

4bi: 63% yield
E/Z 50:50; $rr_{\alpha/\beta}$ >20:1

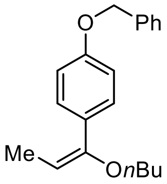

4bj: 64% yield
E/Z 50:50; $rr_{\alpha/\beta}$ >20:1

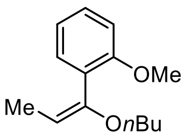

4bk: 59% yield
E/Z 50:50; $rr_{\alpha/\beta}$ >20:1

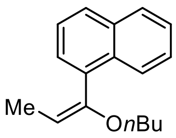

4bl: 74% yield
E/Z 75:25; $rr_{\alpha/\beta}$ >20:1

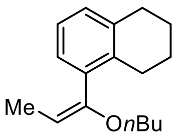

4bm: 47% yield
E/Z 86:14; $rr_{\alpha/\beta}$ >20:1

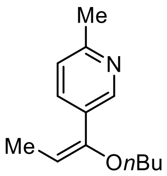

4bn: 60% yield
E/Z 63:37; $rr_{\alpha/\beta}$ >20:1

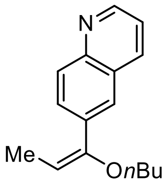

4bo: 82% yield
E/Z 68:32; $rr_{\alpha/\beta}$ >20:1

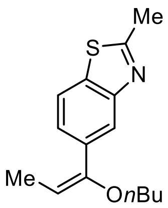

4bp: 68% yield
E/Z 50:50; $rr_{\alpha/\beta}$ >20:1

Figure S7. Scope of aryl triflates **2**.

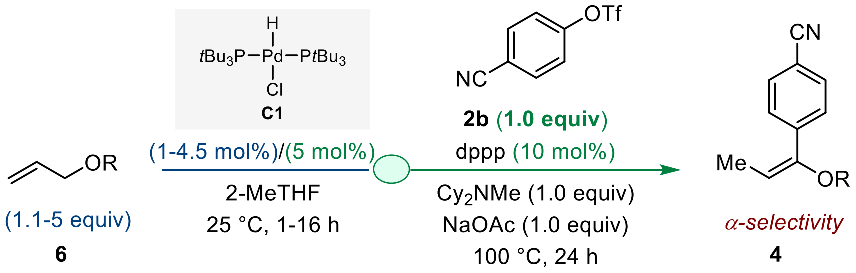

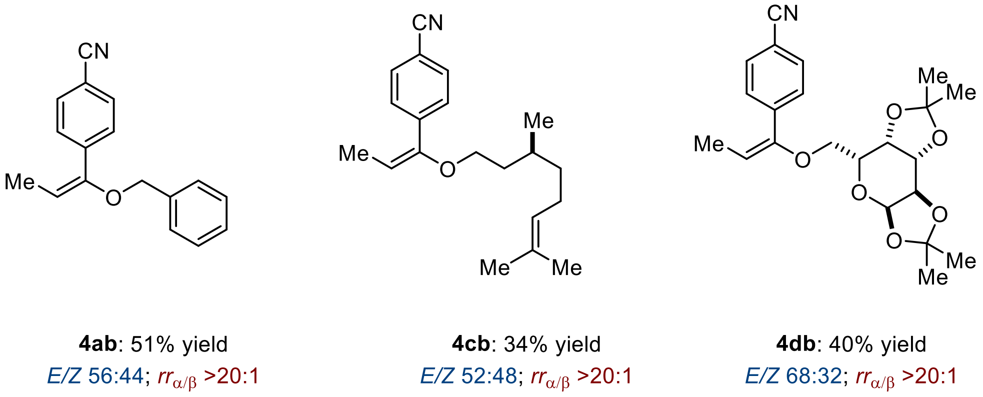

Figure S8. Scope of allyl ethers **6**.

(1-butoxyprop-1-en-1-yl)benzene (4ba)

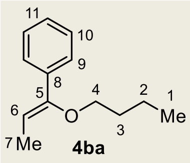

Following GP X using **C1** (8.2 mg, 0.015 mmol, 1 mol% to **6b** - 5 mol% to **2a**), allyl butyl ether **6b** (171 mg, 1.5 mmol, 5 equiv), dppp (12.4 mg, 0.03 mmol, 10 mol% to **2a**), Cy_2NMe (58.6 mg, 0.3 mmol, 1 equiv) and phenyl trifluoromethanesulfonate **2a** (67.8 mg, 0.3 mmol, 1 equiv). Purification by column chromatography (neutral alumina, pentane with 1% Et_3N) afforded product **4ba** in pure form as a colorless oil (44.0 mg, 0.231 mmol, 77% yield, *E/Z* 48:52, $rr_{\alpha/\beta}$ >20:1).

TLC: pentane, R_f = 0.2.

(*Z*): ^{1}H NMR (400 MHz, $CDCl_3$) δ (ppm) = 7.45 – 7.24 (m, 5H, H-9 and H-10 and H-11), 5.36 (q, $^3J_{HH}$ = 6.9 Hz, 1H, H-6), 3.63 (t, $^3J_{HH}$ = 6.6 Hz, 2H, H-4), 1.80 (d, $^3J_{HH}$ = 6.9 Hz, 3H, H-7), 1.73 – 1.64 (m, 2H, H-3), 1.51 – 1.42 (m, 2H, H-2), 0.97 – 0.93 (m, 3H, H-1). **(*E*): ^{1}H NMR** (400 MHz, $CDCl_3$) δ (ppm) = 7.45 – 7.24 (m, 5H, H-9 and H-10 and H-11), 4.83 (q, $^3J_{HH}$ = 7.1 Hz, 1H, H-6), 3.74 (t, $^3J_{HH}$ = 6.5 Hz, 2H, H-4), 1.69 (d, $^3J_{HH}$ = 6.9 Hz, 3H, H-7), 1.73 – 1.64 (m, 2H, H-3), 1.51 – 1.42 (m, 2H, H-2), 0.97 – 0.93 (m, 3H, H-1).

(*Z*): ^{13}C{^{1}H} NMR (101 MHz, $CDCl_3$) δ (ppm) = 154.5 (C-5), 136.9 (C-8), 128.4 (CH-9 or CH-10), 127.6 (CH-11), 125.9 (CH-9 or CH-10), 109.3 (CH-6), 70.4 (CH_2-4), 32.3 (CH_2-3), 19.5 (CH_2-2), 14.1 (CH_3-1), 11.2 (CH_3-7). **(*E*): ^{13}C{^{1}H} NMR** (101 MHz, $CDCl_3$) δ (ppm) = 155.0 (C-5), 136.6 (C-8), 128.9 (CH-9 or CH-10), 128.0 (CH-9 or CH-10), 127.9 (CH-11), 95.5 (CH-6), 67.4 (CH_2-4), 31.6 (CH_2-3), 19.6 (CH_2-2), 14.0 (CH_3-1), 13.1 (CH_3-7).

HRMS (ESI +): calculated for $C_{13}H_{19}O$ $[M+H]^+$: 191.1431; found: 191.1441.

IR (neat) ν (cm^{-1}): 2936, 2871, 1654, 1619, 1322, 1164, 1123, 1065, 845.

4-(1-butoxyprop-1-en-1-yl)benzonitrile (4bb)

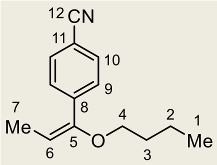

4bb

Following GP X using **C1** (8.2 mg, 0.015 mmol, 1 mol% to **6b** - 5 mol% to **2b**), allyl butyl ether **6b** (171 mg, 1.5 mmol, 5 equiv), dppp (12.4 mg, 0.03 mmol, 10 mol% to **2b**), NaOAc (24.6 mg, 0.3 mmol, 1 equiv), Cy_2NMe (58.6 mg, 0.3 mmol, 1 equiv) and **2b** (75.3 mg, 0.3 mmol, 1 equiv). Purification by column chromatography (neutral alumina, pentane/Et_2O 98:2 with 1% Et_3N) afforded product **4bb** in pure form as a pale yellow oil (47.4 mg, 0.219 mmol, 73% yield, *E/Z* 62:38, $rr_{\alpha/\beta}$ >20:1).

Large-scale experiment: Adapted from GP X using **C1** (110 mg, 0.2 mmol, 1 mol% to **6b** - 5 mol% to **2b**), allyl butyl ether **6b** (2.28 g, 20.0 mmol, 5 equiv), dppp (165 mg, 0.4 mmol, 10 mol% to **2b**), NaOAc (328 mg, 4.0 mmol, 1 equiv), Cy_2NMe (781 mg, 4.0 mmol, 1 equiv) and **2b** (1.0 g, 4.0 mmol, 1 equiv). Purification by column chromatography (neutral alumina, column in pentane/Et_2O 98:2 with 1% Et_3N) afforded product **4bb** in pure form as a pale yellow oil (515 mg, 2.4 mmol, 60% yield, *E/Z* 60:40, $rr_{\alpha/\beta}$ >20:1).

*With 1.1 equiv of **6b** and 1 mol% of [Pd]*: Adapted from GP XI using $[Pd(H)Cl(PtBu_3)_2]$ **C1** (1.64 mg, 0.003 mmol, 0.9 mol% to **6b** - 1 mol% to **2b**), allyl butyl ether **6b** (37.7 mg, 0.33 mmol, 1.1 equiv), dppp (2.47 mg, 0.006 mmol, 10 mol% to **2b**), NaOAc (24.6 mg, 0.3 mmol, 1 equiv), Cy_2NMe (58.6 mg, 0.3 mmol, 1 equiv) and **2b** (75.3 mg, 0.3 mmol, 1 equiv). Reaction time for the isomerization: 16 h. Purification by column chromatography (neutral alumina, column in pentane/Et_2O 98:2 with 1% Et_3N) afforded product **4bb** in pure form as a pale yellow oil (35.1 mg, 0.162 mmol, 54% yield, *E/Z* 60:40, $rr_{\alpha/\beta}$ >20:1).

TLC: pentane/Et_2O 98:2, R_f = 0.2.

(*E*): ^{1}H NMR (400 MHz, $CDCl_3$) δ (ppm) = 7.65 – 7.62 (m, 2H, H-10), 7.54 – 7.50 (m, 2H, H-9), 4.9 (q, $^3J_{HH}$ = 7.2 Hz, 1H, H-6), 3.7 (t, $^3J_{HH}$ = 6.4 Hz, 2H, H-4), 1.7 (d, $^3J_{HH}$ = 7.2 Hz, 3H, H-7), 1.71 – 1.65 (m, 2H, H-3), 1.51 – 1.41 (m, 2H, H-2), 0.97 – 0.93 (m, 3H, H-1). **(*Z*): ^{1}H NMR** (400 MHz, $CDCl_3$) δ (ppm) = 7.61 – 7.59 (m, 2H, H-10), 7.54 – 7.50 (m, 2H, H-9), 5.6 (q, $^3J_{HH}$ = 6.9 Hz, 1H, H-6), 3.6 (t, $^3J_{HH}$ = 6.6 Hz, 2H, H-4), 1.8 (d, $^3J_{HH}$ = 7.0 Hz, 3H, H-7), 1.71 – 1.65 (m, 2H, H-3), 1.51 – 1.41 (m, 2H, H-2), 0.97 – 0.93 (m, 3H, H-1).

(*E*): $^{13}C\{^1H\}$ NMR (101 MHz, $CDCl_3$) δ (ppm) = 153.2 (C-5), 141.2 (C-8), 131.9 (CH-10), 129.5 (CH-9), 119.0 (CN), 111.5 (C-11), 97.4 (CH-6), 67.7 (CH_2-4), 31.4 (CH_2-3), 19.5 (CH_2-2), 14.0 (CH_3-1), 13.1 (CH_3-7). **(*Z*): $^{13}C\{^1H\}$ NMR** (101 MHz, $CDCl_3$) δ (ppm) = 153.1 (C-5), 141.4 (C-8), 132.4 (CH-10), 126.1 (CH-9), 119.1 (CN), 113.3 (CH-6), 111.0 (C-11), 70.9 (CH_2-4), 32.2 (CH_2-3), 19.4 (CH_2-2), 14.0 (CH_3-1), 11.5 (CH_3-7).

HRMS (ESI +): calculated for $C_{14}H_{18}NO$ $[M+H]^+$: 216.1388; found: 216.1379.

IR (neat) ν (cm^{-1}): 2933, 2228, 1650, 1607, 1226, 1129, 1068, 847, 537.

1-(1-butoxyprop-1-en-1-yl)-4-(trifluoromethyl)benzene (4bc)

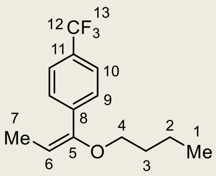

4bc

Following GP X using **C1** (8.2 mg, 0.015 mmol, 1 mol% to **6b** - 5 mol% to **2c**), allyl butyl ether **6b** (171 mg, 1.5 mmol, 5 equiv), dppp (12.4 mg, 0.03 mmol, 10 mol% to **2c**), NaOAc (24.6 mg, 0.3 mmol, 1 equiv), Cy_2NMe (58.6 mg, 0.3 mmol, 1 equiv) and **2c** (88.2 mg, 0.3 mmol, 1 equiv). Purification by column chromatography (neutral alumina, pentane with 1% Et_3N) afforded product **4bc** in pure form as a colorless oil (41.9 mg, 0.162 mmol, 54% yield, *E/Z* 63:37, $rr_{\alpha/\beta}$ >20:1).

TLC: pentane, R_f = 0.5.

(*E*): ^{1}H NMR (400 MHz, $CDCl_3$) δ (ppm) = 7.62 – 7.51 (m, 4H, H-9 and H-10), 4.9 (q, $^3J_{HH}$ = 7.1 Hz, 1H, H-6), 3.7 (t, $^3J_{HH}$ = 6.4 Hz, 2H, H-4), 1.7 (d, $^3J_{HH}$ = 7.1 Hz, 3H, H-7), 1.74 – 1.65 (m, 2H, H-3), 1.50 – 1.42 (m, 2H, H-2), 0.97 – 0.94 (m, 3H, H-1). **(*Z*): ^{1}H NMR** (400 MHz, $CDCl_3$) δ (ppm) = 7.62 – 7.51 (m, 4H, H-9 and H-10), 7.54 – 7.50 (m, 2H, H-9), 5.5 (q, $^3J_{HH}$ = 6.9 Hz, 1H, H-6), 3.6 (t, $^3J_{HH}$ = 6.6 Hz, 2H, H-4), 1.83 (d, $^3J_{HH}$ = 6.9 Hz, 3H, H-7), 1.74 – 1.65 (m, 2H, H-3), 1.50 – 1.42 (m, 2H, H-2), 0.97 – 0.94 (m, 3H, H-1).

(*E*): ^{13}C{^{1}H} NMR (101 MHz, $CDCl_3$) δ (ppm) = 153.7 (C-5), 140.2 (C-8), 129.9 (q, $^2J_{CF}$ = 33.2 Hz, C-11), 129.2 (CH-9), 125.0 (q, $^3J_{CF}$ = 3.8 Hz, C-10), 124.3 (q, $^1J_{CF}$ = 273 Hz, C-12), 96.7 (CH-6), 67.6 (CH_2-4), 31.5 (CH_2-3), 19.6 (CH_2-2), 14.0 (CH_3-1), 13.0 (CH_3-7). **(*Z*): ^{13}C{^{1}H} NMR** (101 MHz, $CDCl_3$) δ (ppm) =. 153.4 (C-5), 140.4 (C-8), 129.9 (q, $^2J_{CF}$ = 33.2 Hz, C-11), 125.9 (CH-9) , 125.5 (q, $^3J_{CF}$ = 3.9 Hz, CH-10), 124.3 (q, $^1J_{CF}$ = 273 Hz, C-12), 111.8 (CH-6), 70.7 (CH_2-4), 32.3 (CH_2-3), 19.4 (CH_2-2), 14.1 (CH_3-1), 11.4 (CH_3-7).

(*E*): ^{19}F{^{1}H} NMR (282 MHz, $CDCl_3$) δ (ppm) = –62.5 (s, F-13). **(*Z*): ^{19}F{^{1}H} NMR** (282 MHz, $CDCl_3$) δ (ppm) = –62.5 (s, F-13).

HRMS (ESI +): calculated for $C_{14}H_{18}F_3O$ $[M+H]^+$: 259.1310; found: 259.1328.

IR (neat) ν (cm^{-1}): 2936, 2871, 1654, 1619, 1322, 1164, 1123, 1065, 845.

1-(4-(1-butoxyprop-1-en-1-yl)phenyl)ethan-1-one (4bd)

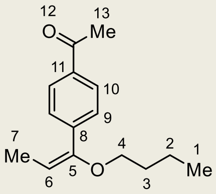

4bd

Following GP X using **C1** (8.2 mg, 0.015 mmol, 1 mol% to **6b** - 5 mol% to **2d**), allyl butyl ether **6b** (171 mg, 1.5 mmol, 5 equiv), dppp (12.4 mg, 0.03 mmol, 10 mol% to **2d**), NaOAc (24.6 mg, 0.3 mmol, 1 equiv), Cy_2NMe (58.6 mg, 0.3 mmol, 1 equiv) and **2d** (80.4 mg, 0.3 mmol, 1 equiv). Purification by column chromatography (neutral alumina, pentane/Et_2O 90:10 with 1% Et_3N) afforded product **4bd** in pure form as a pale yellow oil (45.1 mg, 0.195 mmol, 65% yield, *E/Z* 60:40, $rr_{\alpha/\beta}$ >20:1).

TLC: pentane/Et_2O 90:10 R_f = 0.1.

(*E*): ^{1}H NMR (400 MHz, $CDCl_3$) δ (ppm) = 7.95 (d, $^3J_{HH}$ = 8.6 Hz, 2H, H-10), 7.54 – 7.49 (m, 2H, H-9), 4.91 (q, $^3J_{HH}$ = 7.1 Hz, 1H, H-6), 3.74 (t, $^3J_{HH}$ = 6.4 Hz, 2H, H-4), 2.61 (s, 3H, H-13), 1.71 (d, $^3J_{HH}$ = 7.2 Hz, 3H, H-7), 1.74 – 1.65 (m, 2H, H-3), 1.51 – 1.42 (m, 2H, H-2), 0.97 – 0.94 (m, 3H, H-1). **(*Z*): ^{1}H NMR** (400 MHz, $CDCl_3$) δ (ppm) = 7.92 (d, $^3J_{HH}$ = 8.5 Hz, 2H, H-10), 7.54 – 7.49 (m, 2H, H-9), 5.55 (q, $^3J_{HH}$ = 6.9 Hz, 1H, H-6), 3.63 (t, $^3J_{HH}$ = 6.6 Hz, 2H, H-4), 2.60 (s, 3H, H-13), 1.83 (d, $^3J_{HH}$ = 6.9 Hz, 3H, H-7), 1.74 – 1.65 (m, 2H, H-3), 1.51 – 1.42 (m, 2H, H-2), 0.97 – 0.94 (m, 3H, H-1).

(*E*): ^{13}C{^{1}H} NMR (101 MHz, $CDCl_3$) δ (ppm) = 197.9 (C-13), 153.9 (C-5), 141.3 (C-8), 136.4 (C-11), 129.0 (CH-9), 128.1 (CH-10), 97.1 (CH-6), 67.6 (CH_2-4), 31.5 (CH_2-3), 26.8 (CH_3-13), 19.6 (CH_2-2), 14.1 (CH_3-1), 13.1 (CH_3-7). **(*Z*): ^{13}C{^{1}H} NMR** (101 MHz, $CDCl_3$) δ (ppm) = 197.7 (C-13), 153.7 (C-5), 141.5 (C-8), 136.2 (C-11), 128.7 (CH-10), 125.7 (CH-9), 112.3 (CH-6), 70.8 (CH_2-4), 32.3 (CH_2-3), 26.7 (CH_3-13), 19.4 (CH_2-2), 14.0 (CH_3-1), 11.4 (CH_3-7)

HRMS (ESI +): calculated for $C_{15}H_{21}O_2$ $[M+H]^+$: 233.1542; found: 233.1561.

IR (neat) ν (cm^{-1}): 2932, 2869, 1682, 1604, 1357, 1264, 1131, 847.

1-(1-butoxyprop-1-en-1-yl)-4-nitrobenzene (4be)

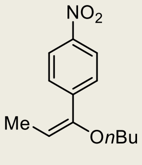

4be

Following GP X using **C1** (8.2 mg, 0.015 mmol, 1 mol% to **6b** - 5 mol% to **2e**), allyl butyl ether **6b** (171 mg, 1.5 mmol, 5 equiv), dppp (12.4 mg, 0.03 mmol, 10 mol% to **2e**), NaOAc (24.6 mg, 0.3 mmol, 1 equiv), Cy_2NMe (58.6 mg, 0.3 mmol, 1 equiv) and **2e** (81.3 mg, 0.3 mmol, 1 equiv). Purification by column chromatography (neutral alumina, cyclohexane/EtOAc 99:1 with 1% Et_3N) afforded product **4be** in pure form as a yellow oil (32.7 mg, 0.138 mmol, 46% yield, *E/Z* 58:42, $rr_{\alpha/\beta}$ >20:1). The spectroscopic data are in agreement with the literature.[21]

1-(1-butoxyprop-1-en-1-yl)-4-chlorobenzene (4bf)

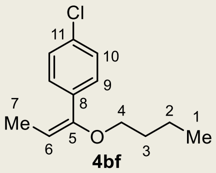

4bf

Following GP X using **C1** (8.2 mg, 0.015 mmol, 1 mol% to **6b** - 5 mol% to **2f**), allyl butyl ether **6b** (171 mg, 1.5 mmol, 5 equiv), dppp (12.4 mg, 0.03 mmol, 10 mol% to **2f**), NaOAc (24.6 mg, 0.3 mmol, 1 equiv), Cy_2NMe (58.6 mg, 0.3 mmol, 1 equiv) and **2f** (78.1 mg, 0.3 mmol, 1 equiv). Purification by column chromatography (neutral alumina, pentane with 1% Et_3N) afforded product **4bf** in pure form as a colorless oil (50.4 mg, 0.225 mmol, 75% yield, *E/Z* 60:40, $rr_{\alpha/\beta}$ >20:1).

TLC: pentane, R_f = 0.2.

(*E*): ^{1}H NMR (400 MHz, $CDCl_3$) δ (ppm) = 7.65 – 7.30 (m, 4H, H-9 and H-10), 4.83 (q, $^3J_{HH}$ = 7.1 Hz, 1H, H-6), 3.72 (t, $^3J_{HH}$ = 6.4 Hz, 2H, H-4), 1.72 – 1.63 (m, 2H, H-3), 1.68 (d, $^3J_{HH}$ = 7.0 Hz, 3H, H-7), 1.50 – 1.41 (m, 2H, H-2), 0.97 – 0.92 (m, 3H, H-1). **(*Z*): ^{1}H NMR** (400 MHz, $CDCl_3$) δ (ppm) = 7.38 – 7.35 (m, 2H, H-9), 7.30 – 7.28 (m, 2H, H-10), 5.36 (q, $^3J_{HH}$ = 6.9 Hz, 1H, H-6), 3.60 (t, $^3J_{HH}$ = 6.6 Hz, 2H, H-4), 1.79 (d, $^3J_{HH}$ = 6.9 Hz, 3H, H-7), 1.72 – 1.63 (m, 2H, H-3), 1.50 – 1.41 (m, 2H, H-2), 0.97 – 0.92 (m, 3H, H-1).

(*E*): ^{13}C{^{1}H} NMR (101 MHz, $CDCl_3$) δ (ppm) = 153.9 (C-5), 135.0 (C-8), 133.7 (C-11), 130.2 (CH-9 or CH-10), 128.2 (CH-9 or CH-10), 95.9 (CH-6), 67.5 (CH_2-4), 31.5 (CH_2-3), 19.6 (CH_2-2), 14.0 (CH_3-1), 13.0 (CH_3-7). **(*Z*): ^{13}C{^{1}H} NMR** (101 MHz, $CDCl_3$) δ (ppm) = 153.5 (C-5), 135.4 (C-8), 133.3 (C-11), 128.6 (CH-Ar), 127.1 (CH-Ar), 110.0 (CH-6), 70.5 (CH_2-4), 32.3 (CH_2-3), 19.4 (CH_2-2), 14.1 (CH_3-1), 11.2 (CH_3-7).

HRMS (ESI +): calculated for $C_{13}H_{18}OCl$ $[M+H]^+$: 225.1046; found: 225.1048.

IR (neat) ν (cm^{-1}): 2932, 1654, 1490, 1223, 1129, 1091, 833.

1-(1-butoxyprop-1-en-1-yl)-3-phenoxybenzene (4bg)

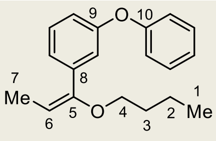

4bg

Following GP X using **C1** (8.2 mg, 0.015 mmol, 1 mol% to **6b** - 5 mol% to **2g**), allyl butyl ether **6b** (171 mg, 1.5 mmol, 5 equiv), dppp (12.4 mg, 0.03 mmol, 10 mol% to **2g**), NaOAc (24.6 mg, 0.3 mmol, 1 equiv), Cy_2NMe (58.6 mg, 0.3 mmol, 1 equiv) and **2g** (95.4 mg, 0.3 mmol, 1 equiv). Purification by column chromatography (neutral alumina, pentane/Et_2O 98:2 with 1% Et_3N) afforded product **4bg** in pure form as a pale yellow oil (42 mg, 0.15 mmol, 50% yield, *E/Z* 50:50, $rr_{\alpha/\beta}$ >20:1).

TLC: pentane/Et_2O 98:2, R_f = 0.3.

(*E*): ^{1}H NMR (400 MHz, $CDCl_3$) δ (ppm) = 7.36 – 7.28 (m, 3H, H-Ar), 7.19 – 7.06 (m, 3H, H-Ar), 7.06 – 7.01 (m, 2H, H-Ar), 6.96 – 6.89 (m, 1H, H-Ar), 4.81 (q, $^3J_{HH}$ = 7.1 Hz, 1H, H-6), 3.71 (t, $^3J_{HH}$ = 6.5 Hz, 2H, H-4), 1.68 (d, $^3J_{HH}$ = 7.1 Hz, 3H, H-7), 1.71 – 1.60 (m, 2H, H-3), 1.46 – 1.38 (m, 2H, H-2), 0.95 – 0.90 (m, 3H, H-1). **(*Z*): ^{1}H NMR** (400 MHz, $CDCl_3$) δ (ppm) = 7.36 – 7.28 (m, 3H, H-Ar), 7.19 – 7.06 (m, 3H, H-Ar), 7.06 – 7.01 (m, 2H, H-Ar), 6.96 – 6.89 (m, 1H, H-Ar), 5.37 (q, $^3J_{HH}$ = 6.9 Hz, 1H, H-6), 3.62 (t, $^3J_{HH}$ = 6.6 Hz, 2H, H-4), 1.78 (d, $^3J_{HH}$ = 7.0 Hz, 3H, H-7), 1.71 – 1.60 (m, 2H, H-3), 1.46 – 1.38 (m, 2H, H-2), 0.95 – 0.90 (m, 3H, H-1).

(*E*): ^{13}C{^{1}H} NMR (101 MHz, $CDCl_3$) δ (ppm) = 157.3 (C-10 or C-9), 157.3 (C-10 or C-9), 153.8 (C-5), 138.3 (C-8), 129.9 (CH-Ar), 129.7 (CH-Ar), 123.8 (CH-Ar), 123.4 (CH-Ar), 119.4 (CH-Ar), 119.1 (CH-Ar), 118.3 (CH-Ar), 95.9 (CH-6), 67.5 (CH_2-4), 31.5 (CH_2-3), 19.5 (CH_2-2), 14.1 (CH_3-1), 13.1 (CH_3-7). **(*Z*): ^{13}C{^{1}H} NMR** (101 MHz, $CDCl_3$) δ (ppm) = 157.6 (C-10 or C-9), 157.0 (C-10 or C-9), 154.3 (C-8), 138.8 (C-8), 129.9 (CH-Ar), 129.3 (CH-Ar), 123.4 (CH-Ar), 120.7 (CH-Ar), 119.1 (CH-Ar), 119.0 (CH-Ar), 118.0 (CH-Ar), 110.0 (CH-6), 70.5 (CH_2-4), 32.3 (CH_2-3), 19.4 (CH_2-2), 14.0 (CH_3-1), 11.2 (CH_3-7).

HRMS (ESI +): calculated for $C_{19}H_{23}O_2$ $[M+H]^+$: 283.1696; found: 283.1700.

IR (neat) ν (cm^{-1}): 1654, 1576, 1486, 1432, 1269, 1219, 1071, 889, 787, 752, 691.

1-(3-(1-butoxyprop-1-en-1-yl)phenyl)ethan-1-one (4bh)

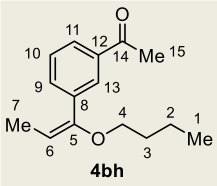

Following GP X using **C1** (8.2 mg, 0.015 mmol, 1 mol% to **6b** - 5 mol% to **2h**), allyl butyl ether **6b** (171 mg, 1.5 mmol, 5 equiv), dppp (12.4 mg, 0.03 mmol, 10 mol% to **2h**), NaOAc (24.6 mg, 0.3 mmol, 1 equiv), Cy_2NMe (58.6 mg, 0.3 mmol, 1 equiv) and **2h** (80.4 mg, 0.3 mmol, 1 equiv). Purification by column chromatography (neutral alumina, pentane/Et_2O 90:10 with 1% Et_3N) afforded product **4bh** in pure form as a pale yellow oil (49.0 mg, 0.21 mmol, 70% yield, *E/Z* 55:45, $rr_{\alpha/\beta}$ >20:1).

TLC: pentane/Et_2O 90:10, R_f = 0.6.

(*E*): ^{1}H NMR (400 MHz, $CDCl_3$) δ (ppm) = 7.99 (t, $^4J_{HH}$ = 1.8 Hz, 1H, H-13), 7.90 (ddd, $^3J_{HH}$ = 7.8 Hz, $^4J_{HH}$ = 1.9, 1.2 Hz, 1H, H-11), 7.60 (dt, $^3J_{HH}$ = 7.7 Hz, $^4J_{HH}$ = 1.4 Hz, 1H, H-9), 7.47 – 7.40 (m, 1H, H-10), 4.87 (q, $^3J_{HH}$ = 7.1 Hz, 1H, H-6), 3.75 (t, $^3J_{HH}$ = 6.5 Hz, 2H, H-4), 2.61 (s, 3H, H-15), 1.74 – 1.65 (m, 2H, H-3), 1.69 (d, $^3J_{HH}$ = 7.1 Hz, 3H, H-7), 1.51 – 1.42 (m, 2H, H-2), 0.97 – 0.93 (m, 3H, H-1). **(*Z*): ^{1}H NMR** (400 MHz, $CDCl_3$) δ (ppm) = 8.00 (t, $^4J_{HH}$ = 1.8 Hz, 1H, H-13), 7.85 (ddd, $^3J_{HH}$ = 7.8 Hz, $^4J_{HH}$ = 1.8, 1.2 Hz, 1H, H-11), 7.63 (ddd, $^3J_{HH}$ = 7.7 Hz, $^4J_{HH}$ = 1.8, 1.2 Hz, 1H, H-9), 7.47 – 7.40 (m, 1H, H-10), 5.46 (q, $^3J_{HH}$ = 6.9 Hz, 1H, H-6), 3.62 (t, $^3J_{HH}$ = 6.6 Hz, 2H, H-4), 2.61 (s, 3H, H-15), 1.82 (d, $^3J_{HH}$ = 6.8 Hz, 3H, H-7), 1.74 – 1.65 (m, 2H, H-3), 1.51 – 1.42 (m, 2H, H-2), 0.97 – 0.93 (m, 3H, H-1).

(*E*): ^{13}C{^{1}H} NMR (101 MHz, $CDCl_3$) δ (ppm) = 198.2 (C-14), 154.1 (C-5), 137.1 (C-12), 137.0 (C-8), 133.6 (CH-9), 128.9 (CH-13), 128.4 (CH-10), 127.7 (CH-11), 96.0 (CH-6), 67.6 (CH_2-4), 31.5 (CH_2-3), 26.8 (CH_3-15), 19.6 (CH_2-2), 14.0 (CH_3-1), 13.0 (CH_3-7). **(*Z*): ^{13}C{^{1}H} NMR** (101 MHz, $CDCl_3$) δ (ppm) = 198.2 (C-14), 153.6 (C-5), 137.5 (C-12), 137.4 (C-8), 130.3 (CH-9), 128.8 (CH-10), 127.5 (CH-11), 125.8 (CH-13), 110.7 (CH-6), 70.6 (CH_2-4), 32.3 (CH_2-3), 26.8 (CH_3-15), 19.5 (CH_2-2), 14.1 (CH_3-1), 11.3 (CH_3-7).

HRMS (ESI +): calculated for $C_{15}H_{21}O_2$ $[M+H]^+$: 233.1542; found: 233.1592.

IR (neat) ν (cm^{-1}): 2933, 1686, 1427, 1357, 1263, 1217, 796, 696, 588.

1-(1-butoxyprop-1-en-1-yl)-4-methoxybenzene (4bi)

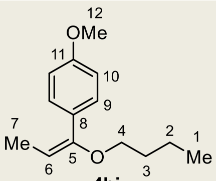

4bi

Following GP X using **C1** (8.2 mg, 0.015 mmol, 1 mol% to **6b** - 5 mol% to **2i**), allyl butyl ether **6b** (171 mg, 1.5 mmol, 5 equiv), dppp (12.4 mg, 0.03 mmol, 10 mol% to **2i**), Cy_2NMe (58.6 mg, 0.3 mmol, 1 equiv) and **2i** (76.8 mg, 0.3 mmol, 1 equiv). Purification by column chromatography (neutral alumina, pentane/Et_2O 98:2 with 1% Et_3N) afforded product **4bi** in pure form as a pale yellow oil (41.9 mg, 0.189 mmol, 63% yield, *E/Z* 50:50, $rr_{\alpha/\beta}$ >20:1).

TLC: pentane/Et_2O 98:2, R_f = 0.2.

(*E*): ^{1}H NMR (400 MHz, $CDCl_3$) δ (ppm) = 7.37 – 7.33 (m, 2H, H-9), 6.91 – 6.85 (m, 2H, H-10), 4.77 (q, $^3J_{HH}$ = 7.1 Hz, 1H, H-6), 3.82 (s, 3H, H-12), 3.71 (t, $^3J_{HH}$ = 6.5 Hz, 2H, H-4), 1.68 (d, $^3J_{HH}$ = 7.0 Hz, 3H, H-7), 1.72 – 1.62 (m, 2H, H-3), 1.50 – 1.41 (m, 2H, H-2), 0.96 – 0.92 (m, 3H, H-1). **(*Z*): ^{1}H NMR** (400 MHz, $CDCl_3$) δ (ppm) = 7.37 – 7.33 (m, 2H, H-9), 6.91 – 6.85 (m, 2H, H-10), 5.23 (q, $^3J_{HH}$ = 6.8 Hz, 1H, H-6), 3.81 (s, 3H, H-12), 3.61 (t, $^3J_{HH}$ = 6.6 Hz, 2H, H-4), 1.77 (d, $^3J_{HH}$ = 7.0 Hz, 3H, H-7), 1.72 – 1.62 (m, 2H, H-3), 1.50 – 1.41 (m, 2H, H-2), 0.96 – 0.92 (m, 3H, H-1).

(*E*): ^{13}C{^{1}H} NMR (101 MHz, $CDCl_3$) δ (ppm) = 159.2 (C-11), 154.7 (C-5), 130.1 (CH-9), 129.1 (C-8), 113.4 (CH-10), 94.8 (CH-6), 67.4 (CH_2-4), 55.4 (CH_3-12), (31.6 (CH_2-3), 19.6 (CH_2-2), 14.1 (CH_3-1), 13.1 (CH_3-7). **(*Z*): ^{13}C{^{1}H} NMR** (101 MHz, $CDCl_3$) δ (ppm) = 159.3 (C-11), 154.2 (C-5), 129.5 (C-8), 127.2 (CH-9), 113.8 (CH-10), 107.5 (CH-6), 70.2 (CH_2-4), 55.4 (CH_3-12), 32.3 (CH_2-3), 19.5 (CH_2-2), 14.1 (CH_3-1), 11.2 (CH_3-7).

HRMS (ESI +): calculated for $C_{14}H_{21}O_2$ $[M+H]^+$: 221.1542; found: 221.1535.

IR (neat) ν (cm^{-1}): 2931, 1609, 1510, 1245, 1035, 834, 473.

1-(benzyloxy)-4-(1-butoxyprop-1-en-1-yl)benzene (4bj)

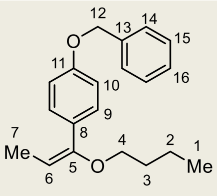

Following GP X using **C1** (8.2 mg, 0.015 mmol, 1 mol% to **6b** - 5 mol% to **2j**), allyl butyl ether **6b** (171 mg, 1.5 mmol, 5 equiv), dppp (12.4 mg, 0.03 mmol, 10 mol% to **2j**), Cy_2NMe (58.6 mg, 0.3 mmol, 1 equiv) and **2j** (99.6 mg, 0.3 mmol, 1 equiv). Purification by column chromatography (neutral alumina, pentane/Et_2O 98:2 with 1% Et_3N) afforded product **4bj** in pure form as a pale brown oil (56.9 mg, 0.192 mmol, 64% yield, *E/Z* 50:50, $rr_{\alpha/\beta}$ >20:1).

TLC: pentane/Et_2O 96:4, R_f = 0.2.

(*E*): ^{1}H NMR (400 MHz, $CDCl_3$) δ (ppm) = 7.45 – 7.43 (m, 2H, H-14), 7.41 – 7.37 (m, 3H, H-15 and H-16), 7.35 – 7.33 (m, 2H, H-9), 7.0 – 6.92 (m, 2H, H-10), 5.08 (s, 2H, H-12), 4.78 (q, $^3J_{HH}$ = 7.1 Hz, 1H, H-6), 3.72 (t, $^3J_{HH}$ = 6.4 Hz, 2H, H-4), 1.69 (d, $^3J_{HH}$ = 7.0 Hz, 3H, H-7), 1.72 – 1.63 (m, 2H, H-3), 1.50 – 1.42 (m, 2H, H-2), 0.97 – 0.92 (m, 3H, H-1). **(*Z*): ^{1}H NMR** (400 MHz, $CDCl_3$) δ (ppm) = 7.45 – 7.43 (m, 2H, H-14), 7.41 – 7.37 (m, 3H, H-15 and H-16), 7.35 – 7.33 (m, 2H, H-9), 7.0 – 6.92 (m, 2H, H-10), 5.24 (q, $^3J_{HH}$ = 6.8 Hz, 1H, H-6), 5.08 (s, 2H, H-12), 3.61 (t, $^3J_{HH}$ = 6.6 Hz, 2H, H-4), 1.78 (d, $^3J_{HH}$ = 6.8 Hz, 3H, H-7), 1.72 – 1.63 (m, 2H, H-3), 1.50 – 1.42 (m, 2H, H-2), 0.97 – 0.92 (m, 3H, H-1).

(*E*): ^{13}C{^{1}H} NMR (101 MHz, $CDCl_3$) δ (ppm) = 158.5 (C-11), 154.1 (C-5), 137.1 (C-13), 129.4 (C-8), 128.7 (CH-9),128.1 (CH-16 or CH-15 or CH-14), 127.6 (CH-16 or CH-15 or CH-14), 127.2 (CH-16 or CH-15 or CH-14), 114.7 (CH-10), 94.8 (CH-6), 70.3 (CH_2-12), 67.4 (CH_2-4), 31.6 (CH_2-3), 19.6 (CH_2-2), 14.1 (CH_3-1), 13.1 (CH_3-7). **(*Z*): ^{13}C{^{1}H} NMR** (101 MHz, $CDCl_3$) δ (ppm) = 158.5 (C-11), 154.7 (C-5), 137.1 (C-13), 130.1 (CH-9), 129.6 (C-8), 128.7 (CH-16 or CH-15 or CH-14), 128.1 (CH-16 or CH-15 or CH-14), 127.6 (CH-16 or CH-15 or CH-14), 114.3 (CH-10), 107.7 (CH-6), 70.2 (CH_2-12), 70.1 (CH_2-4), 32.3 (CH_2-3), 19.5 (CH_2-2), 14.1 (CH_3-1), 11.2 (CH_3-7).

HRMS (ESI +): calculated for $C_{20}H_{25}O$ $[M+H]^+$: 297.1855; found: 297.1856.

IR (neat) ν (cm^{-1}): 2932, 1652, 1607, 1509, 1455, 1222, 1173, 1074, 1022, 834, 734, 696.

1-(1-butoxyprop-1-en-1-yl)-2-methoxybenzene (4bk)

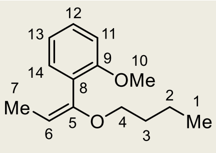

4bk

Following GP X using **C1** (8.2 mg, 0.015 mmol, 1 mol% to **6b** - 5 mol% to **2k**), allyl butyl ether **6b** (171 mg, 1.5 mmol, 5 equiv), dppp (12.4 mg, 0.03 mmol, 10 mol% to **2k**), Cy_2NMe (58.6 mg, 0.3 mmol, 1 equiv) and **2k** (76.8 mg, 0.3 mmol, 1 equiv). Purification by column chromatography (neutral alumina, pentane/Et_2O 98:2 with 1% Et_3N) afforded product **4bk** in pure form as a pale yellow oil (39.2 mg, 0.177 mmol, 59% yield, *E/Z* 50:50, $rr_{\alpha/\beta}$ >20:1).

TLC: pentane/Et_2O 98:2, R_f = 0.1.

(*E*): ^{1}H NMR (400 MHz, $CDCl_3$) δ (ppm) = 7.32 – 7.21 (m, 2H, H-14 and H-12), 6.98 – 6.87 (m, 2H, H-13 and H-11), 4.89 (q, $^3J_{HH}$ = 6.9 Hz, 1H, H-6), 3.83 (s, 3H, H-10), 3.74 (t, $^3J_{HH}$ = 6.5 Hz, 2H, H-4), 1.46 (d, $^3J_{HH}$ = 6.8 Hz, 3H, H-7), 1.70 – 1.63 (m, 2H, H-3), 1.46 – 1.38 (m, 2H, H-2), 0.94 – 0.88 (m, 3H, H-1). **(*Z*): ^{1}H NMR** (400 MHz, $CDCl_3$) δ (ppm) = 7.32 – 7.21 (m, 2H, H-14 and H-12), 6.98 – 6.87 (m, 2H, H-13 and H-11), 5.07 (q, $^3J_{HH}$ = 6.8 Hz, 1H, H-6), 3.85 (s, 3H, H-10), 3.51 (t, $^3J_{HH}$ = 6.5 Hz, 2H, H-4), 1.79 (d, $^3J_{HH}$ = 6.8 Hz, 3H, H-7), 1.63 – 1.55 (m, 2H, H-3), 1.46 – 1.38 (m, 2H, H-2), 0.94 – 0.88 (m, 3H, H-1).

(*E*): ^{13}C{^{1}H} NMR (101 MHz, $CDCl_3$) δ (ppm) = 157.2 (C-9), 152.5 (C-5), 131.4 (CH-14 or CH-12), 129.6 (CH-14 or CH-12), 125.9 (C-8), 120.5 (CH-13 or CH-11), 111.3 (CH-13 or CH-11), 96.7 (CH-6), 68.9 (CH_2-4), 55.6 (CH_3-10), 31.5 (CH_2-3), 19.5 (CH_2-2), 14.0 (CH_3-1), 12.7 (CH_3-7). **(*Z*): ^{13}C{^{1}H} NMR** (101 MHz, $CDCl_3$) δ (ppm) = 157.4 (C-9), 151.9 (C-5), 130.6 (CH-14 or CH-12), 129.2 (CH-14 or CH-12), 125.6 (C-8), 120.5 (CH-13 or CH-11), 110.8 (CH-13 or CH-11), 109.2 (CH-6), 67.5 (CH_2-4), 55.8 (CH_3-10), 32.2 (CH_2-3), 19.4 (CH_2-2), 14.0 (CH_3-1), 11.0 (CH_3-7).

HRMS (ESI +): calculated for $C_{14}H_{21}O_2$ $[M+H]^+$: 221.1542; found: 221.1535.

IR (neat) ν (cm^{-1}): 2932, 1661, 1599, 1491, 1461, 1435, 1243, 1220, 1107, 1027, 800, 750.

1-(1-butoxyprop-1-en-1-yl)naphthalene (4bl)

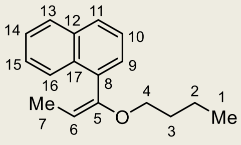
4bl

Following GP X using **C1** (8.2 mg, 0.015 mmol, 1 mol% to **6b** - 5 mol% to **2l**), allyl butyl ether **6b** (171 mg, 1.5 mmol, 5 equiv), dppp (12.4 mg, 0.03 mmol, 10 mol% to **2l**), Cy_2NMe (58.6 mg, 0.3 mmol, 1 equiv) and **2l** (82.8 mg, 0.3 mmol, 1 equiv). Purification by column chromatography (neutral alumina, pentane with 1% Et_3N) afforded product **4bl** in pure form as a colorless oil (53.3 mg, 0.222 mmol, 74% yield, *E/Z* 75:25, $rr_{\alpha/\beta}$ >20:1).

TLC: pentane, R_f = 0.1.

(*E*): ^{1}H NMR (400 MHz, $CDCl_3$) δ (ppm) = 7.97 – 7.94 (m, 1H, H-11), 7.86 – 7.79 (m, 2H, H-14 and H-16), 7.50 – 7.26 (m, 4H, H-9 and H-10 and H-13 and H-15), 5.07 (q, $^3J_{HH}$ = 6.9 Hz, 2H, H-6), 3.83 (t, $^3J_{HH}$ = 6.5 Hz, 2H, H-4), 1.72 – 1.65 (m, 2H, H-3), 1.43 (d, $^3J_{HH}$ = 6.9 Hz, 3H, H-7), 1.47 – 1.37 (m, 2H, H-2), 0.92 (t, $^3J_{HH}$ = 7.4 Hz, 3H, H-1). **(*Z*): ^{1}H NMR** (400 MHz, $CDCl_3$) δ (ppm) = 8.29 – 8.26 (m, 1H, H-11), 7.86 – 7.79 (m, 2H, H-14 and H-16), 7.50 – 7.26 (m, 4H, H-9 and H-10 and H-13 and H-15), 4.99 (q, $^3J_{HH}$ = 6.8 Hz, 2H, H-6), 3.42 (t, $^3J_{HH}$ = 6.5 Hz, 2H, H-4), 1.87 (d, $^3J_{HH}$ = 6.8 Hz, 3H, H-7), 1.56 – 1.49 (m, 2H, H-3), 1.37 – 1.33 (m, 2H, H-2), 0.85 (t, $^3J_{HH}$ = 7.4 Hz, 3H, H-1).

(*E*): ^{13}C{^{1}H} NMR (101 MHz, $CDCl_3$) δ (ppm) = 154.2 (C-5), 134.3 (C-8), 133.8 (C-17), 131.8 (C-12), 128.6 (CH-Ar), 128.3 (CH-Ar), 127.7 (CH-Ar), 126.2 (CH-Ar), 126.1 (CH-Ar), 125.9 (CH-Ar), 125.4 (CH-Ar), 97.0 (CH-6), 67.4 (CH_2-4), 31.5 (CH_2-3), 19.6 (CH_2-2), 14.0 (CH_3-1), 12.8 (CH_3-7). **(*Z*): ^{13}C{^{1}H} NMR** (101 MHz, $CDCl_3$) δ (ppm) = 154.0 (C-5), 135.1 (C-8), 133.5 (C-17), 132.0 (C-12), 128.6 (CH-Ar), 128.2 (CH-Ar), 127.6 (CH-Ar), 126.3 (CH-Ar), 126.1 (CH-Ar), , 126.0 (CH-Ar), 125.3 (CH-Ar), 109.3 (CH-6), 68.7 (CH_2-4), 32.2 (CH_2-3), 19.3 (CH_2-2), 14.0 (CH_3-1), 11.0 (CH_3-7).

HRMS (ESI +): calculated for $C_{17}H_{21}O$ $[M+H]^+$: 241.1592; found: 241.1582.

IR (neat) ν (cm^{-1}): 2930, 1658, 1351, 1230, 1189, 1104, 800, 776, 456.

5-(1-butoxyprop-1-en-1-yl)-1,2,3,4-tetrahydronaphthalene (4bm)

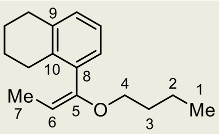

4bm

Following GP X using **C1** (8.2 mg, 0.015 mmol, 1 mol% to **6b** - 5 mol% to **2m**), allyl butyl ether **6b** (171 mg, 1.5 mmol, 5 equiv), dppp (12.4 mg, 0.03 mmol, 10 mol% to **2m**), Cy_2NMe (58.6 mg, 0.3 mmol, 1 equiv) and **2m** (84.0 mg, 0.3 mmol, 1 equiv). Purification by column chromatography (neutral alumina, pentane with 1% Et_3N) afforded product **4bm** in pure form as a colorless oil (34.5 mg, 0.141 mmol, 47% yield, *E/Z* 86:14, $rr_{\alpha/\beta}$ >20:1).

TLC: pentane, R_f = 0.1.

(*E*): ^{1}H NMR (400 MHz, $CDCl_3$) δ (ppm) = 7.10 – 7.03 (m, 2H, H-Ar), 7.03 – 6.98 (m, 1H, H-Ar), 4.79 (q, $^3J_{HH}$ = 7.2 Hz, 1H, H-6), 3.71 (t, $^3J_{HH}$ = 6.6 Hz, 2H, H-4), 2.81 – 2.67 (m, 4H, H-Cy), 1.82 – 1.69 (m, 4H, H-Cy), 1.69 – 1.62 (m, 2H, H-3), 1.46 – 1.37 (m, 2H, H-2), 1.41 (d, $^3J_{HH}$ = 6.9 Hz, 3H, H-7), 0.92 (t, $^3J_{HH}$ = 7.4 Hz, 3H, H-1). **(*Z*): ^{1}H NMR** (400 MHz, $CDCl_3$) δ (ppm) = 7.10 – 7.03 (m, 2H, H-Ar), 7.03 – 6.98 (m, 1H, H-Ar), 4.75 (q, $^3J_{HH}$ = 6.5 Hz, 1H, H-6), 3.39 (t, $^3J_{HH}$ = 6.5 Hz, 2H, H-4), 2.81 – 2.67 (m, 4H, H-Cy), 1.82 – 1.76 (m, 3H, H-7), 1.82 – 1.69 (m, 4H, H-Cy), 1.69 – 1.62 (m, 2H, H-3), 1.46 – 1.37 (m, 2H, H-2), 0.89 (t, $^3J_{HH}$ = 7.3 Hz, 3H, H-1).

(*E*): ^{13}C{^{1}H} NMR (101 MHz, $CDCl_3$) δ (ppm) = 155.6 (C-5), 137.5 (C-9 or C-10), 136.2 (C-9 or C-10), 136.1 (C-8) 129.3 (CH-Ar),127.4 (CH-Ar), 125.1 (CH-Ar), 94.6 (CH-6), 67.0 (CH_2-4), 31.5 (CH_2-3), 30.0 (CH_2-Cy), 26.5 (CH_2-Cy), 23.3 (CH_2-Cy), 23.2 (CH_2-Cy), 19.6 (CH_2-2), 14.0 (CH_3-1), 12.6 (CH_3-7). **(*Z*): ^{13}C{^{1}H} NMR** (101 MHz, $CDCl_3$) δ (ppm) = 154.7 (C-5), 137.3 (C-9 or C-10), 137.1 (C-9 or C-10), 136.9 (C-8), 127.7 (CH-Ar), 125.5 (CH-Ar), 125.0 (CH-Ar), 107.8 (CH-6), 68.3 (CH_2-4), 32.2 (CH_2-3), 29.5 (CH_2-Cy), 27.3 (CH_2-Cy), 23.4 (CH_2-Cy), 23.1 (CH_2-Cy), 19.4 (CH_2-2), 14.0 (CH_3-1), 10.7 (CH_3-7).

HRMS (ESI +): calculated for $C_{17}H_{25}O$ $[M+H]^+$: 245.1905; found: 245.1907.

IR (neat) ν (cm^{-1}): 2929, 1658, 1453, 1234, 1176, 1104, 779, 511.

5-(1-butoxyprop-1-en-1-yl)-2-methylpyridine (4bn)

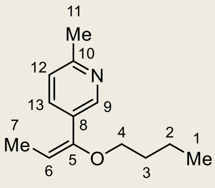

4bn

Following GP X using **C1** (8.2 mg, 0.015 mmol, 1 mol% to **6b** - 5 mol% to **2n**), allyl butyl ether **6b** (171 mg, 1.5 mmol, 5 equiv), dppp (12.4 mg, 0.03 mmol, 10 mol% to **2n**), NaOAc (24.6 mg, 0.3 mmol, 1 equiv), Cy_2NMe (58.6 mg, 0.3 mmol, 1 equiv) and **2n** (72.3 mg, 0.3 mmol, 1 equiv). Purification by column chromatography (neutral alumina, cyclohexane/EtOAc 80:20) afforded product **4bn** in pure form as a pale yellow oil (37.0 mg, 0.180 mmol, 60% yield, *E/Z* 63:37, $rr_{\alpha/\beta}$ >20:1).

TLC: cyclohexane/EtOAc 80:20, R_f = 0.2.

(*E*): ^{1}H NMR (400 MHz, $CDCl_3$) δ (ppm) = 8.53 (dd, $^4J_{HH}$ = 2.3 Hz, $^5J_{HH}$ = 0.9 Hz, 1H, H-9), 7.61 – 7.58 (m, 1H, H-13), 7.14 (d, $^3J_{HH}$ = 8.0 Hz, 1H, H-12), 4.87 (q, $^3J_{HH}$ = 7.1 Hz, 1H, H-6), 3.73 (t, $^3J_{HH}$ = 6.4 Hz, 2H, H-4), 2.56 (s, 3H, H-11),1.69 (d, $^3J_{HH}$ = 7.0 Hz, 3H, H-7), 1.70 – 1.63 (m, 2H, H-3), 1.50 – 1.40 (m, 2H, H-2), 0.96 – 0.92 (m, 3H, H-1). **(*Z*): ^{1}H NMR** (400 MHz, $CDCl_3$) δ (ppm) = 8.57 (dd, $^4J_{HH}$ = 2.4 Hz, $^5J_{HH}$ = 0.8 Hz, 1H, H-9), 7.61 – 7.58 (m, 1H, H-13), 7.10 (d, $^3J_{HH}$ = 8.0 Hz, 1H, H-12), 5.36 (q, $^3J_{HH}$ = 6.9 Hz, 1H, H-6), 3.61 (t, $^3J_{HH}$ = 6.6 Hz, 2H, H-4), 2.54 (s, 3H, H-11), 1.80 (d, $^3J_{HH}$ = 6.9 Hz, 3H, H-7), 1.70 – 1.63 (m, 2H, H-3), 1.50 – 1.40 (m, 2H, H-2), 0.96 – 0.92 (m, 3H, H-1).

(*E*): ^{13}C{^{1}H} NMR (101 MHz, $CDCl_3$) δ (ppm) = 157.7 (C-10), 152.4 (C-5), 149.4 (CH-9), 136.5 (CH-13), 129.4 (C-8), 122.6 (CH-12), 96.4 (CH-6), 67.5 (CH_2-4), 31.5 (CH_2-3), 24.4 (CH_3-11), 19.5 (CH_2-2), 14.0 (CH_3-1), 13.0 (CH_3-7). **(*Z*): ^{13}C{^{1}H} NMR** (101 MHz, $CDCl_3$) δ (ppm) = 157.5 (C-10), 152.0 (C-5), 146.8 (CH-9), 133.5 (CH-13), 129.6 (C-8), 122.9 (CH-12), 110.3 (CH-6), 70.5 (CH_2-4), 32.2 (CH_2-3), 24.3 (CH_3-11), 19.4 (CH_2-2), 14.0 (CH_3-1), 11.2 (CH_3-7).

HRMS (ESI +): calculated for $C_{13}H_{19}NO$ $[M+H]^+$: 206.1545; found: 206.1590.

IR (neat) ν (cm^{-1}): 2932, 1655, 1598, 1488, 1365, 1225, 1134, 1070, 834, 802.

6-(1-butoxyprop-1-en-1-yl)quinoline (4bo)

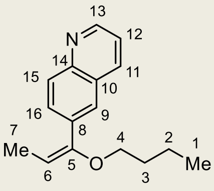

4bo

Following GP X using **C1** (8.2 mg, 0.015 mmol, 1 mol% to **6b** - 5 mol% to **2o**), allyl butyl ether **6b** (171 mg, 1.5 mmol, 5 equiv), dppp (12.4 mg, 0.03 mmol, 10 mol% to **2o**), NaOAc (24.6 mg, 0.3 mmol, 1 equiv), Cy_2NMe (58.6 mg, 0.3 mmol, 1 equiv) and **2o** (83.1 mg, 0.3 mmol, 1 equiv). Purification by column chromatography (neutral alumina, cyclohexane/EtOAc gradient from 80:20 to 70:30) afforded product **4bo** in pure form as a pale yellow oil (59.1 mg, 0.246 mmol, 82% yield, *E/Z* 68:32, $rr_{\alpha/\beta}$ >20:1).

TLC: cyclohexane/EtOAc 80:20, R_f = 0.1.

(*E*): ^{1}H NMR (400 MHz, $CDCl_3$) δ (ppm) = 8.91 (dd, $^3J_{HH}$ = 4.2 Hz, $^4J_{HH}$ = 1.8 Hz, 1H, H-13), 8.17 – 8.13 (m, 1H, H-11), 8.09 (d, $^3J_{HH}$ = 8.7 Hz, 1H, H-15), 7.86 – 7.77 (m, 2H, H-16 and H-9), 7.42 – 7.37 (m, 1H, H-12), 4.95 (q, $^3J_{HH}$ = 7.1 Hz, 1H, H-6), 3.80 (t, $^3J_{HH}$ = 6.5 Hz, 2H, H-4), 1.76 (d, $^3J_{HH}$ = 7.1 Hz, 3H, H-7), 1.77 – 1.69 (m, 2H, H-3), 1.53 – 1.46 (m, 2H, H-2), 0.99 – 0.94 (m, 3H, H-1). **(*Z*): ^{1}H NMR** (400 MHz, $CDCl_3$) δ (ppm) = 8.87 (dd, $^3J_{HH}$ = 4.2 Hz, $^4J_{HH}$ = 1.8 Hz, 1H, H-13), 8.17 – 8.13 (m, 1H, H-11), 8.04 (d, $^3J_{HH}$ = 9.0 Hz, 1H, H-15), 7.86 – 7.77 (m, 2H, H-16 and H-9), 7.42 – 7.37 (m, 1H, H-12), 5.58 (q, $^3J_{HH}$ = 6.9 Hz, 1H, H-6), 3.69 (t, $^3J_{HH}$ = 6.6 Hz, 2H, H-4), 1.87 (d, $^3J_{HH}$ = 7.0 Hz, 3H, H-7), 1.77 – 1.69 (m, 2H, H-3), 1.53 – 1.46 (m, 2H, H-2), 0.99 – 0.94 (m, 3H, H-1).

(*E*): ^{13}C{^{1}H} NMR (101 MHz, $CDCl_3$) δ (ppm) = 154.3 (C-5), 150.7 (CH-13), 148.0 (C-14), 136.4 (CH-11), 134.8 (C-8), 130.5 (CH-9 or CH-16),129.2 (CH-15), 127.9 (C-10), 127.9 (CH-9 or CH-16), 121.4 (CH-12), 96.5 (CH-6), 67.7 (CH_2-4), 31.5 (CH_2-3), 19.6 (CH_2-2), 14.0 (CH_3-1), 13.2 (CH_3-7). **(*Z*): ^{13}C{^{1}H} NMR** (101 MHz, $CDCl_3$) δ (ppm) = 153.9 (C-5), 150.3 (CH-13), 148.1 (C-14), 136.3 (CH-11), 135 (C-8), 129.5 (CH-15), 128.4 (C-10), 127.8 (CH-15 or CH-16), 124.1 (CH-15 or CH-16), 121.5 (CH-12), 111.2 (CH-6), 70.8 (CH_2-4), 32.3 (CH_2-3), 19.5 (CH_2-2), 14.1 (CH_3-1), 11.4 (CH_3-7).

HRMS (ESI +): calculated for $C_{16}H_{20}NO$ $[M+H]^+$: 242.1545; found: 242.1569.

IR (neat) ν (cm^{-1}): 2932, 1651, 1497, 1460, 1359, 1217, 1183, 1110, 1069, 840, 797.

5-(1-butoxyprop-1-en-1-yl)-2-methylbenzo[*d*]thiazole (4bp)

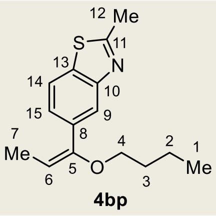

Following GP X using **C1** (8.2 mg, 0.015 mmol, 1 mol% to **6b** - 5 mol% to **2p**), allyl butyl ether **6b** (171 mg, 1.5 mmol, 5 equiv), dppp (12.4 mg, 0.03 mmol, 10 mol% to **2p**), NaOAc (24.6 mg, 0.3 mmol, 1 equiv), Cy_2NMe (58.6 mg, 0.3 mmol, 1 equiv) and **2p** (89.1 mg, 0.3 mmol, 1 equiv). Purification by column chromatography (neutral alumina, cyclohexane/EtOAc 99:1) afforded product **4bp** in pure form as a colorless oil (53.3 mg, 0.204 mmol, 68% yield, *E/Z* 50:50, $rr_{\alpha/\beta}$ >20:1).

TLC: cyclohexane/EtOAc 96:4 R_f = 0.3.

(*Z*): ^{1}H NMR (400 MHz, $CDCl_3$) δ (ppm) = 8.01 (d, $^4J_{HH}$ = 1.7 Hz, 1H, H-9), 7.75 (d, $^3J_{HH}$ = 8.4 Hz, 1H, H-14), 7.45 (dd, $^3J_{HH}$ = 8.4 Hz, $^4J_{HH}$ = 1.7 Hz, 1H, H-15), 5.45 (q, $^3J_{HH}$ = 6.8 Hz, 1H, H-6), 3.66 (t, $^3J_{HH}$ = 6.6 Hz, 2H, H-4), 2.83, (s, 3H, H-12), 1.83 (d, $^3J_{HH}$ = 6.9 Hz, 3H, H-7), 1.74 – 1.66 (m, 2H, H-3), 1.52 – 1.42 (m, 2H, H-2), 0.97 – 0.92 (m, 3H, H-1). **(*E*): ^{1}H NMR** (400 MHz, $CDCl_3$) δ (ppm) = 8.00 (d, $^4J_{HH}$ = 1.6 Hz, 1H, H-9), 7.79 (d, $^3J_{HH}$ = 8.4 Hz, 1H, H-14), 7.42 (dd, $^3J_{HH}$ = 8.2 Hz, $^4J_{HH}$ = 1.6 Hz, 1H, H-15), 4.89 (q, $^3J_{HH}$ = 7.1 Hz, 1H, H-6), 3.77 (t, $^3J_{HH}$ = 6.4 Hz, 2H, H-4), 2.84 (s, 3H, H-12), 1.73 (d, $^3J_{HH}$ = 7.0 Hz, 3H, H-7), 1.74 – 1.66 (m, 2H, H-3), 1.52 – 1.42 (m, 2H, H-2), 0.97 – 0.92 (m, 3H, H-1).

(*Z*): ^{13}C{^{1}H} NMR (101 MHz, $CDCl_3$) δ (ppm) = 167.6 (C-11), 154.1(C-5), 153.9 (C-Ar), 135.3(C-Ar), 134.7 (C-Ar), 122.9 (CH-15), 121.2 (CH-14), 119.5 (CH-9), 109.9 (CH-6), 70.6 (CH_2-4), 32.3 (CH_2-3), 20.3 (CH_3-12), 19.5 (CH_2-2), 14.1 (CH_3-1), 11.4 (CH_3-7). **(*E*): ^{13}C{^{1}H} NMR** (101 MHz, $CDCl_3$) δ (ppm) = 167.6 (C-11), 154.5 (C-5), 153.3 (C-Ar), 135.2 (C-Ar), 134.8 (C-Ar), 125.6 (CH-15), 122.9 (CH-9), 120.9 (CH-14), 96.0 (CH-6), 67.6 (CH_2-4), 31.6 (CH_2-3), 20.3 (CH_3-12), 19.6 (CH_2-2), 14.0 (CH_3-1), 13.2 (CH_3-7).

HRMS (ESI +): calculated for $C_{15}H_{20}NOS$ $[M+H]^+$: 262.1266; found: 262.1284.

IR (neat) ν (cm^{-1}): 2930, 1653, 1526, 1454, 1413, 1316, 1198, 1171, 1112, 1069, 810, 641, 512.

4-(1-(benzyloxy)prop-1-en-1-yl)benzonitrile (4ab)

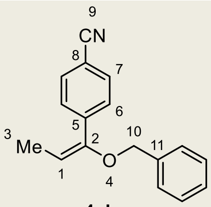

4ab

Following GP X using **C1** (8.2 mg, 0.015 mmol, 1 mol% to **6a** - 5 mol% to **2b**), allyl benzyl ether **6a** (222 mg, 1.5 mmol, 5 equiv), dppp (12.4 mg, 0.03 mmol, 10 mol% to **2b**), NaOAc (24.6 mg, 0.3 mmol, 1 equiv), Cy_2NMe (58.6 mg, 0.3 mmol, 1 equiv) and **2b** (75.3 mg, 0.3 mmol, 1 equiv). Purification by column chromatography (neutral alumina, pentane/EtOAc 98:2 with 1% Et_3N) afforded product **4ab** in pure form as a pale yellow oil (38.0 mg, 0.153 mmol, 51% yield, *E/Z* 56:44, $rr_{\alpha/\beta}$ >20:1).

TLC: pentane/EtOAc 98:2, R_f = 0.2.

(*E*): ^{1}H NMR (400 MHz, $CDCl_3$) δ (ppm) = 7.66 – 7.62 (m, 2H, H-7), 7.58 – 7.54 (m, 2H, H-6), 7.38 – 7.30 (m, 5H, H-Ar), 5.06 (q, $^3J_{HH}$ = 7.2 Hz, 1H, H-1), 4.84 (s, 2H, H-10), 1.73 (d, $^3J_{HH}$ = 7.2 Hz, 3H, H-3). **(*Z*): ^{1}H NMR** (400 MHz, $CDCl_3$) δ (ppm) = 7.66 – 7.62 (m, 2H, H-7), 7.58 – 7.54 (m, 2H, H-6), 7.38 – 7.30 (m, 5H, H-Ar), 5.61 (q, $^3J_{HH}$ = 7.0 Hz, 1H, H-1), 4.66 (s, 2H, H-10), 1.76 (d, $^3J_{HH}$ = 7.0 Hz, 3H, H-3).

(*E*): ^{13}C{^{1}H} NMR (101 MHz, $CDCl_3$) δ (ppm) = 152.9 (C-2), 140.8 (CH-5), 137.2 (CH-11), 132.0 (CH-7), 129.6 (CH-6), 128.7 (CH-Ar), 128.3 (CH-Ar), 127.6 (CH-Ar), 119.0 (CN), 111.6 (C-8), 98.9 (CH-1), 70.1 (CH_2-10), 13.1 (CH_3-1). **(*Z*): ^{13}C{^{1}H} NMR** (101 MHz, $CDCl_3$) δ (ppm) = 152.6 (C-2), 141.1 (CH-5), 137.2 (CH-11), 132.5 (CH-7), 128.6 (CH-Ar), 128.3 (CH-Ar), 128.0 (CH-Ar), 126.2 (CH-6), 119.0 (CN), 114.3 (CH-1), 111.2 (C-8), 72.9 (CH_2-10), 11.6 (CH_3-1).

HRMS (ESI +): calculated for $C_{17}H_{16}NO$ $[M+H]^+$: 250.1232; found: 250.1230.

IR (neat) ν (cm^{-1}): 2914, 2227, 1650, 1606, 1499, 1454, 1364, 1314, 1228, 1124, 1102, 1047, 1018, 847, 735, 697.

(*S*,*E*)-4-(1-((3,7-dimethyloct-6-en-1-yl)oxy)prop-1-en-1-yl)benzonitrile (4cb)

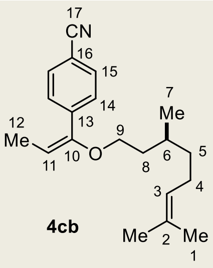

4cb

Following GP XI using **C1** (8.2 mg, 0.015 mmol, 4.5 mol% to **6c** - 5 mol% to **2b**), allyl ether **6c** (64.7 mg, 0.33 mmol, 1.1 equiv), dppp (12.4 mg, 0.03 mmol, 10 mol% to **2b**), NaOAc (24.6 mg, 0.3 mmol, 1 equiv), Cy_2NMe (58.6 mg, 0.3 mmol, 1 equiv) and **2b** (75.3 mg, 0.3 mmol, 1 equiv). Purification by column chromatography (neutral alumina, pentane/Et_2O 96:4 with 1% Et_3N) afforded product **4cb** in pure form as pale brown oil (30.0 mg, 0.102 mmol, 34% yield, *E/Z* 52:48, $rr_{\alpha/\beta}$ >20:1).

TLC: pentane/Et_2O 96:4, R_f = 0.1.

(*E*): ^{1}H NMR (400 MHz, $CDCl_3$) δ (ppm) = 7.64 (d, $^3J_{HH}$ = 8.2 Hz, 2H, H-15), 7.54 – 7.50 (m, 2H, H-14), 5.11 – 5.07 (m, 1H, H-3), 4.93 (q, $^3J_{HH}$ = 7.2 Hz, 1H, H-11), 3.79 – 3.74 (m, 2H, H-9), 2.07 – 1.92 (m, 2H, H-4), 1.79 – 1.72 (m, 1H, H-8), 1.71 (d, $^3J_{HH}$ = 7.2 Hz, 3H, H-12), 1.66 – 1.61 (m, 1H, H-6), 1.68 (s, 6H, H-1), 1.57 – 1.48 (m, 1H, H-8'), 1.40 – 1.31 (m, 1H, H-5), 1.23 – 1.15 (m, 1H, H-5'), 0.94 – 0.90 (m, 3H, H-7). **(*Z*): ^{1}H NMR** (400 MHz, $CDCl_3$) δ (ppm) = 7.61 (d, $^3J_{HH}$ = 8.6 Hz, 2H, H-15), 7.54 – 7.50 (m, 2H, H-14), 5.57 (q, $^3J_{HH}$ = 6.9 Hz, 1H, H-11), 5.11 – 5.07 (m, 1H, H-3), 3.66 – 3.62 (m, 2H, H-9), 2.07 – 1.92 (m, 2H, H-4), 1.83 (d, $^3J_{HH}$ = 7.0 Hz, 3H, H-12), 1.79 – 1.72 (m, 1H, H-8), 1.60 (s, 6H, H-1), 1.66 – 1.61 (m, 1H, H-6), 1.57 – 1.48 (m, 1H, H-8'), 1.40 – 1.31 (m, 1H, H-5), 1.23 – 1.15 (m, 1H, H-5'), 0.94 – 0.90 (m, 3H, H-7).

(*E*): ^{13}C{^{1}H} NMR (101 MHz, $CDCl_3$) δ (ppm) = 153.2 (C-10), 141.4 (C-13), 131.9 (CH-15), 131.5 (C-2), 126.1 (CH-14), 124.8 (CH-3), 119.0 (CN), 111.5 (C-16), 97.5 (CH-11), 66.2 (CH_2-9), 37.2 (CH_2-5), 37.2 (CH_2-8), 29.7 (CH-6), 25.9 (CH_2-4), 25.6 (CH_3-1), 19.7 (CH_3-7), 13.1 (CH_3-12). **(*Z*): ^{13}C{^{1}H} NMR** (101 MHz, $CDCl_3$) δ (ppm) = 153.1 (C-10), 141.1 (C-13), 132.4 (CH-15), 131.5 (C-2), 129.5 (CH-14), 124.7 (CH-3), 119.1 (CN), 113.3 (CH-11), 111.0 (C-16), 69.5 (CH_2-9), 37.2 (CH_2-5), 36.2 (CH_2-8), 29.5 (CH-6), 25.9 (CH_2-4), 17.8 (CH_3-1), 19.7 (CH_3-7), 11.5 (CH_3-12).

HRMS (ESI +): calculated for $C_{20}H_{28}NO$ $[M+H]^+$: 298.2171; found: 298.2163.

IR (neat) ν (cm^{-1}): 2923, 2228, 1650, 1606, 1450, 1377, 1226, 1129, 1067, 848.

4-((*E*)-1-(((3a*R*,5*R*,5a*S*,8a*S*,8b*R*)-2,2,7,7-tetramethyltetrahydro-5*H*-bis([1,3]dioxolo)[4,5-*b*:4',5'-d]pyran-5-yl)methoxy)prop-1-en-1-yl)benzonitrile (4db)

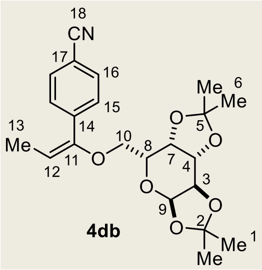

Following GP XI using **C1** (8.21 mg, 0.015 mmol, 4.5 mol% to **6d** - 5 mol% to **2b**), allyl ether **6d** (99.1 mg, 0.33 mmol, 1.1 equiv), dppp (12.4 mg, 0.03 mmol, 10 mol% to **2b**), NaOAc (24.6 mg, 0.3 mmol, 1 equiv), Cy_2NMe (58.6 mg, 0.3 mmol, 1 equiv) and **2b** (75.3 mg, 0.3 mmol, 1 equiv). Purification by column chromatography (neutral alumina, pentane/EtOAc 95:5) afforded product **4db** in pure form as pale brown oil (48.1 mg, 0.120 mmol, 40% yield, *E/Z* 68:32, $rr_{\alpha/\beta}$ >20:1).

Signs of decomposition of the product were observed by ^{1}H and ^{13}C{^{1}H } NMR spectroscopy within 1 h after isolation.

TLC: pentane/EtOAc 95:5, R_f = 0.2.

^{1}H NMR ($CDCl_3$, 400 MHz) δ (ppm) = **(*E*): ^{1}H NMR** (400 MHz, $CDCl_3$) δ (ppm) = 7.66 – 7.52 (m, 4H, H-15 and H-16), 5.58 – 5.55 (m, 1H, H-9), 5.01 (q, $^3J_{HH}$ = 7.2 Hz, 1H, H-12), 4.34 (dd, $^3J_{HH}$ = 5.0, 2.4 Hz, 1H, H-3), 4.63 (dd, $^3J_{HH}$ = 7.9, 2.4 Hz, 1H, H-4), 4.30 (dd, $^3J_{HH}$ = 7.9, 1.9 Hz, 1H, H-7), 4.13 (td, $^3J_{HH}$ = 6.1, 2.0 Hz, 1H, H-8) 3.99 – 3.90 (m, 2H, H-10), 1.72 (d, $^3J_{HH}$ = 7.2 Hz, 3H, H-13), 1.51 (s, 3H, H-1 or H-6), 1.46 (s, 3H, H-1 or H-6), 1.36 – 1.32 (m, 6H, H-1' or H-6'). **(*Z*): ^{1}H NMR** (400 MHz, $CDCl_3$) δ (ppm) = 7.66 – 7.52 (m, 4H, H-15 and H-16), 5.58 – 5.55 (m, 1H, H-9), 5.58 – 5.55 (m, 1H, H-12), 4.27 (dd, $^3J_{HH}$ = 7.2, 2.0 Hz, 1H, H-7), 4.34 (dd, $^3J_{HH}$ = 5.0, 2.4 Hz, 1H, H-3), 4.63 – 4.61 (m, 1H, H-4), 4.27 (dd, $^3J_{HH}$ = 7.8, 1.9 Hz, 1H, H-7), 4.10 – 4.06 (m, 1H, H-8), 3.88 – 3.77 (m, 2H, H-10), 1.86 (d, $^3J_{HH}$ = 7.0 Hz, 3H, H-13), 1.55 (s, 3H, H-1 or H-6), 1.41 (s, 3H, H-1 or H-6), 1.36 – 1.32 (m, 6H, H-1' or H-6').

^{13}C{^{1}H} NMR ($CDCl_3$, 101 MHz) δ (ppm) = **(*E*): ^{13}C{^{1}H} NMR** (101 MHz, $CDCl_3$) δ (ppm) = 152.6 (C-11), 140.7 (C-14), 131.9 (CH-15 or CH-16), 129.6 (CH-15 or CH-16), 119.0 (CN), 111.5 (C-17), 109.6 (C-5), 108.9 (C-2), 99.0 (CH-12), 96.5 (CH-9), 71.3 (CH-7), 70.8 (CH-3), 70.8 (CH-4) , 66.9 (CH_2-10), 66.3 (CH-8), 26.2 (CH_3-1 or CH_3-6), 26.1 (CH_3-1 or CH_3-6), 25.1 (CH_3-1 or CH_3-6), 24.6 (CH_3-1 or CH_3-6), 13.0 (CH_3-13). **(*Z*): ^{13}C{^{1}H} NMR** (101 MHz, $CDCl_3$) δ (ppm) = 152.9 (C-11), 140.7 (C-9), 133.3 (CH-15 or CH-16), 126.3 (CH-15 or CH-16), 119.1 (CN), 113.5 (CH-12), 111.0 (C-17), 109.6 (C-5), 108.8 (C-2), 96.5 (CH-9), 71.3 (CH-7), 70.8 (CH-3), 70.6 (CH-4), 70.0 (CH_2-10), 66.9 (CH-8), 26.2 (CH_3-1 or CH_3-6), 26.1 (CH_3-1 or CH_3-6), 25.1 (CH_3-1 or CH_3-6), 24.6 (CH_3-1 or CH_3-6), 11.7 (CH_3-13).

HRMS (ESI +): calculated for $C_{22}H_{28}NO_6$ $[M+H]^+$: 402.1917; found: 402.1887.

IR (neat) ν (cm^{-1}): 2933, 2228, 1652, 1607, 1381, 1254, 1211, 1168, 1114, 1067, 1002, 849, 731.

8.4. Scope of AT reaction – β-regioselectivity

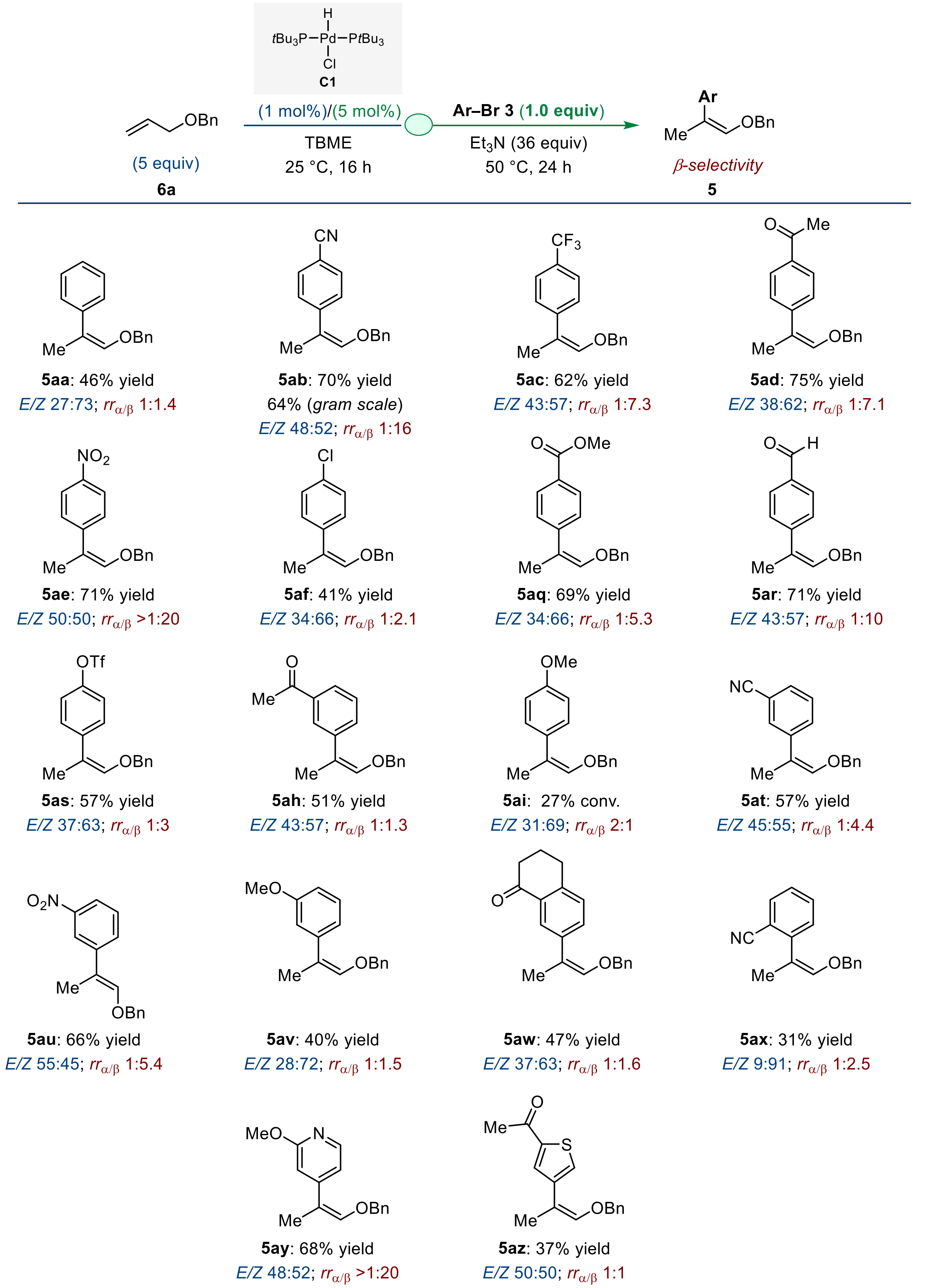

Figure S9. Scope of aryl bromides **3**.

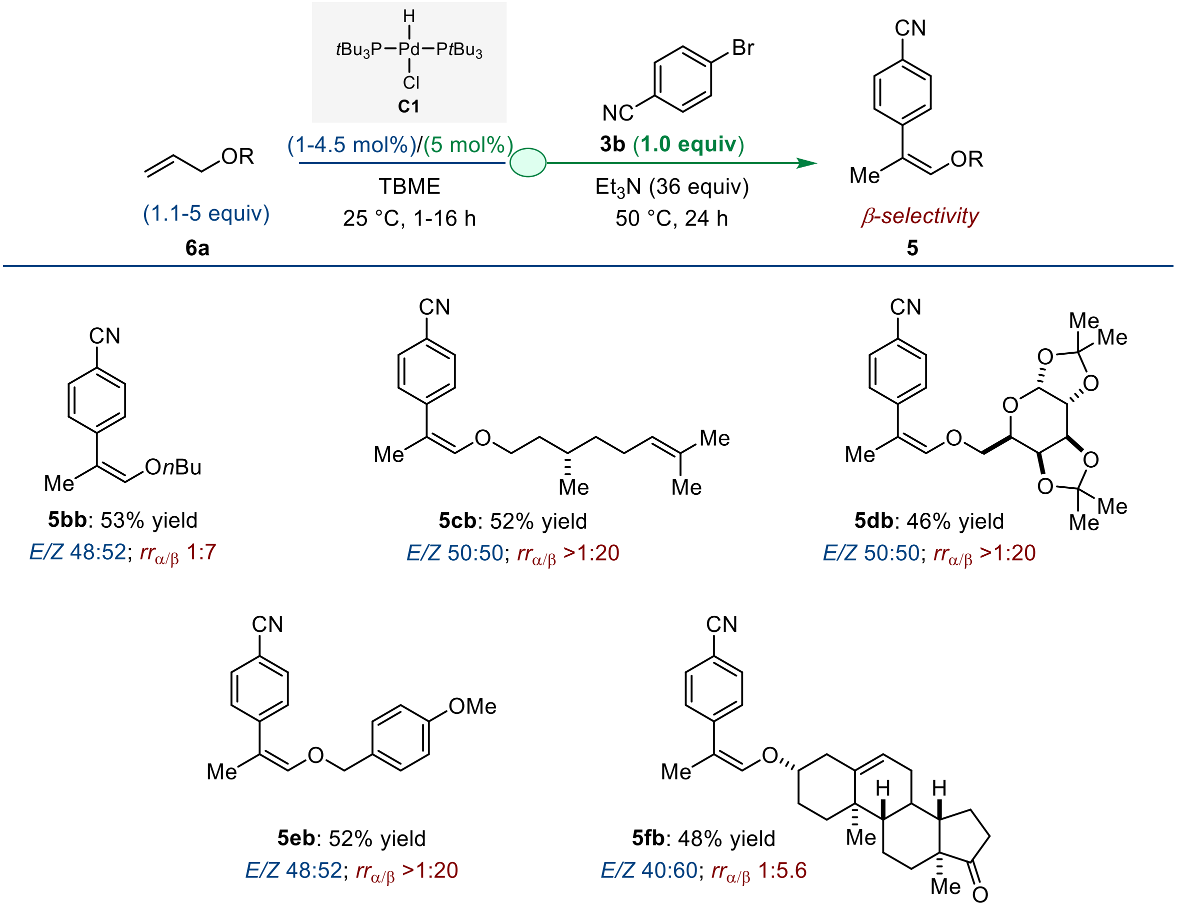

Figure S10. Scope of allyl ethers **6**.

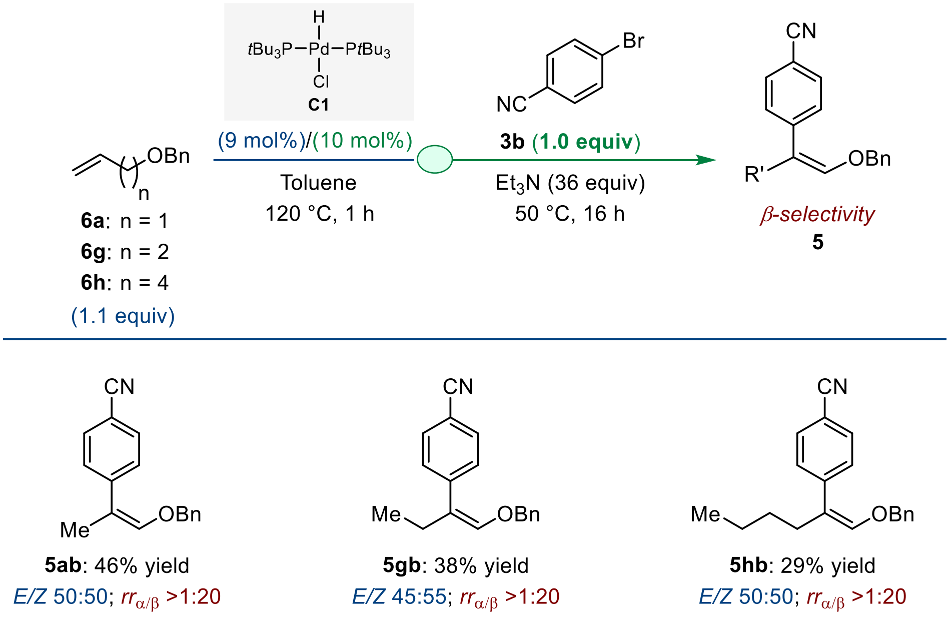

Figure S11. Scope of alkenyl ethers **6a**, **6g** and **6h**.

(1-(benzyloxy)prop-1-en-2-yl)benzene (5aa)

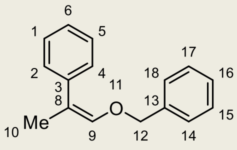

5aa

Following GP XII using **C1** (8.2 mg, 0.015 mmol, 1 mol% to **6a** - 5 mol% to **3a**), benzyl allyl ether **6a** (222 mg, 1.5 mmol, 5 equiv), bromobenzene **3a** (47.1 mg, 0.3 mmol, 1 equiv) and triethylamine (1.5 mL, 36 equiv). Purification by two consecutive column chromatography (1. SiO_2 containing 10% $AgNO_3$, pentane/Et_2O 98:2; 2. SiO_2, pentane/Et_2O 98:2) afforded product **5aa** in pure form as colorless oil (30.8 mg, 0.14 mmol, 46% yield, *E/Z* 27:73, $rr_{\alpha/\beta}$ 1:1.4).

TLC: pentane/Et_2O 97/3, R_f = 0.5.

(*Z*): ^{1}H NMR (400 MHz, $CDCl_3$) δ (ppm) = 7.70 – 7.65 (m, 2H, H-2 and H-4), 7.40 – 7.27 (m, 7H, H-1 and H-5 and H-(14-18)), 7.23 – 7.14 (m, 1H, H-6), 6.26 (q, $^4J_{HH}$ = 1.4 Hz, 1H, H-9), 4.90 (s, 2H, H-12), 1.92 (d, $^4J_{HH}$ = 1.4 Hz, 3H, H-10). **(*E*): ^{1}H NMR** (400 MHz, $CDCl_3$) δ (ppm) = 7.40 – 7.27 (m, 9H, H-(1-5) and H-(14-18)), 7.23 – 7.14 (m, 1H, H-6), 6.57 (q, $^4J_{HH}$ = 1.4 Hz, 1H, H-9), 4.93 (s, 2H, H-12), 2.06 (d, $^4J_{HH}$ = 1.4 Hz, 3H, H-10).

(*Z*): ^{13}C{^{1}H} NMR (101 MHz, $CDCl_3$) δ (ppm) = 143.7 (CH-9), 138.4 (C-3), 137.7 (C-13), 128.7 (CH-15 or CH-16), 128.1 (CH-15 or CH-16), 128.0 (CH-1), 127.7 (CH-2), 127.4 (CH-14), 126.2 (CH-6), 111.5 (C-8), 74.5 (CH_2-12), 18.5 (CH_3-10). **(*E*): ^{13}C{^{1}H} NMR** (101 MHz, $CDCl_3$) δ (ppm) = 143.1 (CH-9), 140.7 (C-3), 137.7 (C-13), 128.7 (CH-15 or CH-16 or CH-2 or CH-1), 128.4 (CH-15 or CH-16 or CH-2 or CH-1), 128.0 (CH-15 or CH-16 or CH-2 or CH-1), 127.5 (CH-14), 126.1 (CH-6), 125.2 (CH-1 or CH-2), 115.4 (C-8), 74.2 (CH_2-12), 12.9 (CH_3-10).

HRMS (ESI +): calculated for $C_{16}H_{16}O$ $[M+H]^+$: 225.1274; found: 225.1288.

IR (neat): ν (cm^{-1}) = 2931, 1648, 1496, 1453, 1443, 1366, 1281, 1138, 1073, 1003, 909, 834.

4-(1-(benzyloxy)prop-1-en-2-yl)benzonitrile (5ab)

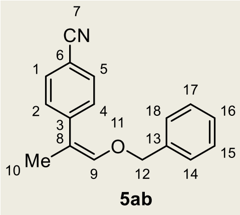

Following GP XII using **C1** (8.2 mg, 0.015 mmol, 1 mol% to **6a** - 5 mol% to **3b**), benzyl allyl ether **6a** (222 mg, 1.5 mmol, 5 equiv), 4-bromobenzonitrile **3b** (54.6 mg, 0.3 mmol, 1 equiv) and triethylamine (1.5 mL, 36 equiv). Purification by column chromatography (SiO_2, pentane/Et_2O 90:10) afforded product **5ab** in pure form as white solid (52.7 mg, 0.21 mmol, 70% yield, *E/Z* 48:52, $rr_{\alpha/\beta}$ 1:16).

Large-scale experiment: Adapted from GP XII using [Pd(H)Cl(PtBu$_3$)$_2$] **C1** (150 mg, 0.28 mmol, 1 mol% to **6a** - 5 mol% to **3b**), benzyl allyl ether **6a** (4.07 g, 27.5 mmol, 5 equiv), 4-bromobenzonitrile **3b** (1.0 g, 5.5 mmol, 1 equiv) and triethylamine (27.5 mL, 36 equiv). Removal of the excess of **6a** was performed by distillation (88% could be recovered). Next, the colorless oil was dissolved in a minimum amount of CH_2Cl_2. Pentane was added and a white precipitate was formed, filtrated and washed with pentane (3 × 20 mL). Product **5ab** was obtained in pure form as a white solid (872 mg, 3.51 mmol, 64% yield, *E/Z* 48:52, $rr_{\alpha/\beta}$ >1:20).

*With 1.1 equiv of **6a***: Adapted from GP XIII using **C1** (150 mg, 0.28 mmol, 4.5 mol% to **6a** - 5 mol% to **3b**), benzyl allyl ether **6a** (48.9 mg, 0.33 mmol, 1.1 equiv), 4-bromobenzonitrile **3b** (54.6 mg, 0.3 mmol, 1 equiv) and triethylamine (1.5 mL, 36 equiv). Purification by column chromatography (SiO_2, pentane/Et_2O 90:10) afforded product **5ab** in pure form as white solid (34.1 mg, 0.14 mmol, 46% yield, *E/Z* 48:52, $rr_{\alpha/\beta}$ >1:20).

*With 1.1 equiv of **6a** and 1 mol% of [Pd]*: Adapted from GP XIII using **C1** (1.6 mg, 0.003 mmol, 0.9 mol% to **6a** - 1 mol% to **3b**), benzyl allyl ether **6a** (48.9 mg, 0.33 mmol, 1.1 equiv), 4-bromobenzonitrile **3b** (54.6 mg, 0.3 mmol, 1 equiv) and triethylamine (1.5 mL, 36 equiv). Purification by column chromatography (SiO_2, pentane/Et_2O 90:10) afforded product **5ab** in pure form as white solid (29.9 mg, 0.12 mmol, 40% yield, *E/Z* 39:61, $rr_{\alpha/\beta}$ >1:20).

With toluene instead of TBME: Following GP XIV using **C1** (16.4 mg, 0.03 mmol, 9 mol% to **6a** - 10 mol% to **3b**), benzyl allyl ether **6a** (48.9 mg, 0.33 mmol, 1.1 equiv), 4-bromobenzonitrile **3b** (54.6 mg, 0.3 mmol, 1 equiv) and triethylamine (1.5 mL, 36 equiv). Purification by column chromatography (SiO_2, pentane/Et_2O 90:10) afforded product 5ab in pure form as white solid (34.0 mg, 0.14 mmol, 46% yield, *E/Z* 50:50, $rr_{\alpha/\beta}$ >1:20).

*With 4-chlorobenzonitrile instead of **3b***: Adapted from GP XII using **C1** (8.2 mg, 0.015 mmol, 1 mol% to **6a** - 5 mol% to **3b**), benzyl allyl ether **6a** (222 mg, 1.5 mmol, 5 equiv), 4-chlorobenzonitrile (41.3 mg, 0.3 mmol, 1 equiv) and triethylamine (1.5 mL, 36 equiv). Purification by column chromatography (SiO_2, pentane/Et_2O 90:10) afforded product **5ab** in pure form as white solid (52.0 mg, 0.21 mmol, 70% yield, *E/Z* 47:53, $rr_{\alpha/\beta}$ >1:20).

TLC: pentane/Et_2O 80:20, R_f = 0.4.

(*Z*): ^{1}H NMR (400 MHz, $CDCl_3$) δ (ppm) = 7.81 – 7.71 (m, 2H, H-2 and H-4), 7.61 – 7.56 (m, 2H, H-1 and H-5), 7.42 – 7.30 (m, 5H, H-(14-18)), 6.40 (q, $^4J_{HH}$ = 1.4 Hz, 1H, H-9), 4.94 (s, 2H, H-12), 1.92 (d, $^4J_{HH}$ = 1.3 Hz, 3H, H-10). **(*E*): ^{1}H NMR** (400 MHz, $CDCl_3$) δ (ppm) = 7.55 – 7.50 (m, 2H, H-1 and H-5), 7.42 – 7.30 (m, 7H, H-2 and H-4 and H-(14-18)), 6.72 (q, $^4J_{HH}$ = 1.3 Hz, 1H, H-9), 4.98 (s, 2H, H-12), 2.03 (d, $^4J_{HH}$ = 1.3 Hz, 3H, H-10).

(*Z*): ^{13}C{^{1}H} NMR (101 MHz, $CDCl_3$) δ (ppm) = 145.9 (CH-9), 143.1 (C-3), 137.0 (C-13), 131.8 (CH-2), 128.8 (CH-15 or CH-16), 128.4 (CH-15 or CH-16), 128.0 (CH-1), 127.5 (CH-14), 119.5 (C-7), 109.7 (C-8), 109.0 (C-6), 75.1 (CH_2-12), 18.0 (CH_3-10). **(*E*): ^{13}C{^{1}H} NMR** (101 MHz, $CDCl_3$) δ (ppm) = 146.3 (CH-9), 145.5 (C-3), 137.1 (C-13), 132.3 (CH-1), 128.8 (CH-15 or CH-16), 128.4 (CH-15 or CH-16), 127.5 (CH-14), 125.1 (CH-2), 119.6 (C-7), 113.9 (C-8), 109.0 (C-6), 74.7 (CH_2-12), 12.4 (CH_3-10).

HRMS (ESI +): calculated for $C_{17}H_{15}NO$ $[M+H]^+$: 250.1227; found: 250.1225.

IR (neat): ν (cm^{-1}) = 2919, 2222, 1645, 1599, 1504, 1454, 1274, 1208, 1143, 1113, 988, 853.

m.p.: 49-52 °C.

1-(1-(benzyloxy)prop-1-en-2-yl)-4-(trifluoromethyl)benzene (5ac)

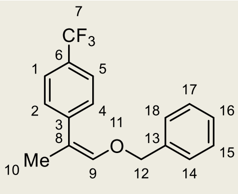

5ac

Following GP XII using **C1** (8.2 mg, 0.015 mmol, 1 mol% to **6a** - 5 mol% to **3c**), benzyl allyl ether **6a** (222 mg, 1.5 mmol, 5 equiv), 1-bromo-4-(trifluoromethyl)benzene **3c** (67.5 mg, 0.3 mmol, 1 equiv) and triethylamine (1.5 mL, 36 equiv). Purification by two consecutive column chromatography (1. SiO_2 containing 10% $AgNO_3$, pentane/Et_2O 99:1; 2. SiO_2, pentane/Et_2O 99:1) afforded product **5ac** in pure form as colorless oil (54.2 mg, 0.19 mmol, 62% yield, *E/Z* 43:57, $rr_{\alpha/\beta}$ 1:7.3).

TLC: pentane/Et_2O 98:2, R_f = 0.4.

(*Z*): ^{1}H NMR (400 MHz, $CDCl_3$) δ (ppm) = 7.81 – 7.74 (m, 2H, H-2 and H-4), 7.60 – 7.54 (m, 2H, H-1 and H-5), 7.44 – 7.32 (m, 5H, H-(14-18)), 6.37 (q, $^4J_{HH}$ = 1.4 Hz, 1H, H-9), 4.94 (s, 2H, H-12), 1.94 (d, $^4J_{HH}$ = 1.4 Hz, 3H, H-10). **(*E*): ^{1}H NMR** (400 MHz, $CDCl_3$) δ (ppm) = 7.56 – 7.49 (m, 2H, H-1 and H-5), 7.44 – 7.32 (m, 7H, H-2 and H-4 and H-(14-18)), 6.67 (q, $^4J_{HH}$ = 1.3 Hz, 1H, H-9), 4.98 (s, 2H, H-12), 2.07 (d, $^4J_{HH}$ = 1.2 Hz, 3H, H-10).

(*Z*): ^{13}C{^{1}H} NMR (101 MHz, $CDCl_3$) δ (ppm) = 145.2 (CH-9), 142.0 (q, $^5J_{CF}$ = 1.4 Hz, C-3), 137.2 (C-13), 128.8 (CH-15 or CH-16), 128.3 (CH-15 or CH-16), 127.8 (q, $^2J_{CF}$ = 32.3 Hz, C-6), 127.7 (CH-2), 127.5 (CH-14), 124.9 (q, $^3J_{CF}$ = 3.8 Hz, CH-1), 124.6 (q, $^1J_{CF}$ = 271.7 Hz, CF_3-7), 110.2 (C-8), 74.9 (CH_2-12), 18.3 (CH_3-10). **(*E*): ^{13}C{^{1}H} NMR** (101 MHz, $CDCl_3$) δ (ppm) = 144.8 (CH-9), 144.4 (q, $^5J_{CF}$ = 1.2 Hz, C-3), 137.4 (C-13), 128.8 (CH-15 or CH-16), 128.3 (CH-15 or CH-16), 127.8 (q, $^2J_{CF}$ = 32.3 Hz, C-6), 127.5 (CH-14), 125.4 (q, $^3J_{CF}$ = 3.8 Hz, CH-1), 125.0 (CH-2), 124.6 (q, $^1J_{CF}$ = 271.7 Hz, CF_3-7), 114.2 (C-8), 74.5 (CH_2-12), 12.7 (CH_3-10).

(*Z*): ^{19}F{^{1}H} NMR (282 MHz, $CDCl_3$) δ (ppm) = -62.4. **(*E*): ^{19}F{^{1}H} NMR** (282 MHz, $CDCl_3$) δ (ppm) = -62.3.

HRMS (ESI +): calculated for $C_{17}H_{15}F_3O$ $[M+NH_4]^+$: 310.1414; found: 310.1397.

IR (neat): ν (cm^{-1}) = 2932, 1648, 1613, 1322, 1161, 1109, 1076, 851, 831.

1-(4-(1-(benzyloxy)prop-1-en-2-yl)phenyl)ethan-1-one (5ad)

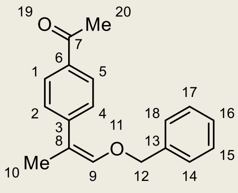

5ad

Following GP XII using **C1** (8.2 mg, 0.015 mmol, 1 mol% to **6a** - 5 mol% to **3d**), benzyl allyl ether **6a** (222 mg, 1.5 mmol, 5 equiv), 4-bromoacetophenone **3d** (59.7 mg, 0.3 mmol, 1 equiv) and triethylamine (1.5 mL, 36 equiv). Purification by two consecutive column chromatography (1. SiO_2 containing 10% $AgNO_3$, pentane/Et_2O 90:10; 2. SiO_2, pentane/Et_2O 90:10) afforded product **5ad** in pure form as white solid (60.0 mg, 0.23 mmol, 75% yield, *E/Z* 38:62, $rr_{\alpha/\beta}$ 1:7.1).

TLC: pentane/Et_2O 80:20, R_f = 0.5.

(*Z*): ^{1}H NMR (400 MHz, $CDCl_3$) δ (ppm) = 7.95 – 7.89 (m, 2H, H-1 and H-2), 7.80 – 7.72 (m, 2H, H-2 and H-4), 7.43 – 7.29 (m, 5H, H-(14-18)), 6.37 (q, $^4J_{HH}$ = 1.4 Hz, 1H, H-9), 4.94 (s, 2H, H-12), 2.59 (s, 3H, H-20), 1.94 (d, $^4J_{HH}$ = 1.3 Hz, 3H, H-10). **(*E*): ^{1}H NMR** (400 MHz, $CDCl_3$) δ (ppm) = 7.89 – 7.83 (m, 2H, H-1 and H-5), 7.43 – 7.29 (m, 7H, H-2 and H-4 and H-(14-18)), 6.74 (q, $^4J_{HH}$ = 1.3 Hz, 1H, H-9), 4.98 (s, 2H, H-12), 2.57 (s, 3H, H-20), 2.06 (d, $^4J_{HH}$ = 1.3 Hz, 3H, H-10).

(*Z*): ^{13}C{^{1}H} NMR (101 MHz, $CDCl_3$) δ (ppm) = 197.9 (C-7), 145.3 (CH-9), 143.4 (C-3), 137.2 (C-13), 134.6 (C-6), 128.8 (CH-15 or CH-16), 128.2 (CH-15 or CH-16), 128.2 (CH-1), 127.6 (CH-14), 127.5 (CH-2), 110.5 (C-8), 74.9 (CH_2-12), 26.7 (CH_3-20), 18.2 (CH_3-10). **(*E*): ^{13}C{^{1}H} NMR** (101 MHz, $CDCl_3$) δ (ppm) = 197.7 (C-7), 145.7 (C-3), 145.7 (CH-9), 137.3 (C-13), 134.7 (C-6), 128.7 (CH-1), 128.3 (CH-15 or CH-16), 128.2 (CH-15 or CH-16), 127.5 (CH-14), 124.7 (CH-2), 114.4 (C-8), 74.5 (CH_2-12), 26.6 (CH_3-20), 12.5 (CH_3-10).

HRMS (ESI +): calculated for $C_{18}H_{18}O_2$ $[M+H]^+$: 267.1380; found: 267.1399.

IR (neat): ν (cm^{-1}) = 2972, 1664, 1637, 1596, 1354, 1272, 1143, 1126, 956, 857.

m.p.: 38-40 °C.

1-(1-(benzyloxy)prop-1-en-2-yl)-4-nitrobenzene (5ae)

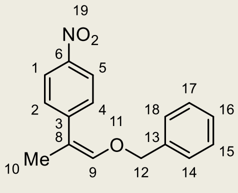
5ae

Following GP XII using **C1** (8.2 mg, 0.015 mmol, 1 mol% to **6a** - 5 mol% to **3e**), benzyl allyl ether **6a** (222 mg, 1.5 mmol, 5 equiv), 4-nitrobromobenzene **3e** (60.6 mg, 0.3 mmol, 1 equiv) and triethylamine (1.5 mL, 36 equiv). Purification by column chromatography (SiO_2, pentane/Et_2O 90:10) afforded product **5ae** in pure form as a yellow solid (57.2 mg, 0.21 mmol, 71% yield, *E/Z* 50:50, $rr_{\alpha/\beta}$ >1:20).

TLC: pentane/Et_2O 80:20, R_f = 0.4.

(*Z*): ^{1}H NMR (400 MHz, $CDCl_3$) δ (ppm) = 8.19 – 8.15 (m, 2H, H-1 and H-5), 7.86 – 7.79 (m, 2H, H-2 and H-4), 7.42 – 7.33 (m, 5H, H-(14-18)), 6.45 (q, $^4J_{HH}$ = 1.4 Hz, 1H, H-9), 4.97 (s, 2H, H-12), 1.95 (d, $^4J_{HH}$ = 1.3 Hz, 3H, H-10). **(*E*): ^{1}H NMR** (400 MHz, $CDCl_3$) δ (ppm) = 8.14 – 8.09 (m, 2H, H-1 and H-5), 7.42 – 7.33 (m, 7H, H-2 and H-4 and H-(14-18)), 6.81 (q, $^4J_{HH}$ = 1.3 Hz, 1H, H-9), 5.01 (s, 2H, H-12), 2.06 (d, $^4J_{HH}$ = 1.3 Hz, 3H, H-10).

(*Z*): ^{13}C{^{1}H} NMR (101 MHz, $CDCl_3$) δ (ppm) = 146.6 (CH-9), 145.5 (C-3), 145.2 (C-6), 136.9 (C-13), 128.8 (CH-15 or CH-16), 128.5 (CH-15 or CH-16), 128.0 (CH-2), 127.5 (CH-14), 123.4 (CH-1), 109.5 (C-8), 75.3 (CH_2-12), 18.2 (CH_3-10). **(*E*): ^{13}C{^{1}H} NMR** (101 MHz, $CDCl_3$) δ (ppm) = 147.7 (C-6), 147.0 (CH-9), 145.8 (C-3), 137.0 (C-13), 128.9 (CH-15 or CH-16), 128.4 (CH-15 or CH-16), 127.5 (CH-14), 124.9 (CH-2), 123.9 (CH-1), 113.7 (C-8), 74.8 (CH_2-12), 12.5 (CH_3-10).

HRMS (ESI +): calculated for $C_{16}H_{15}NO_3$ $[M+H]^+$: 270.1125; found: 270.1107.

IR (neat): ν (cm^{-1}) = 2918, 1633, 1583, 1497, 1333, 1141, 1105, 986, 849.

m.p.: 52-54 °C.

1-(1-(benzyloxy)prop-1-en-2-yl)-4-chlorobenzene (5af)

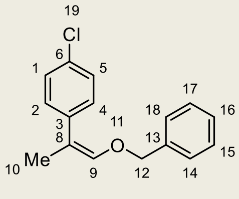

5af

Following GP XII using **C1** (8.2 mg, 0.015 mmol, 1 mol% to **6a** - 5 mol% to **3f**), benzyl allyl ether **6a** (222 mg, 1.5 mmol, 5 equiv), 4-chlorobromobenzene **3f** (57.4 mg, 0.3 mmol, 1 equiv) and triethylamine (1.5 mL, 36 equiv). Purification by two consecutive column chromatography (1. SiO_2 containing 10% $AgNO_3$, pentane/Et_2O 98:2; 2. SiO_2, pentane/Et_2O 98:2) afforded product **5af** in pure form as colorless oil (31.6 mg, 0.12 mmol, 41% yield, *E/Z* 34:66, $rr_{\alpha/\beta}$ 1:2.1).

TLC: pentane/Et_2O 98:2, R_f = 0.5.

(*Z*): ^{1}H NMR (400 MHz, $CDCl_3$) δ (ppm) = 7.65 – 7.57 (m, 2H, H-2 and H-4), 7.40 – 7.31 (m, 5H, H-(14-18)), 7.31 – 7.27 (m, 2H, H-1 and H-5), 6.27 (q, $^4J_{HH}$ = 1.4 Hz, 1H, H-9), 4.90 (s, 2H, H-12), 1.89 (d, $^4J_{HH}$ = 1.4 Hz, 2H, H-10). **(*E*): ^{1}H NMR** (400 MHz, $CDCl_3$) δ (ppm) = 7.40 – 7.31 (m, 5H, H-(14-18)), 7.25 – 7.22 (m, 2H, H-2 and H-4), 7.22 – 7.18 (m, 1H, H-1 and H-5), 6.55 (q, $^4J_{HH}$ = 1.3 Hz, 1H, H-9), 4.93 (s, 2H, H-12), 2.02 (d, $^4J_{HH}$ = 1.4 Hz, 1H, H-10).

(*Z*): ^{13}C{^{1}H} NMR (101 MHz, $CDCl_3$) δ (ppm) = 143.6 (CH-9), 137.4 (C-13), 136.8 (C-3), 131.6 (C-6), 129.0 (CH-2), 128.7 (CH-15 and CH-16), 128.1 (CH-1), 127.4 (CH-14), 110.4 (C-8), 74.7 (CH_2-12), 18.4 (CH_3-10). **(*E*): ^{13}C{^{1}H} NMR** (101 MHz, $CDCl_3$) δ (ppm) = 144.0 (CH-9), 139.2 (C-3), 137.5 (C-13), 131.6 (C-6), 128.5 (CH-2), 128.2 (CH-15 or CH-16), 128.1 (CH-15 or CH-16), 127.5 (CH-14), 126.3 (CH-1), 114.4 (C-8), 74.3 (CH_2-12), 12.9 (CH_3-10).

HRMS (ESI +): calculated for $C_{16}H_{15}ClO$ $[M+NH_4]^+$: 276.1150; found: 276.1153.

IR (neat): ν (cm^{-1}) = 2928, 1646, 1491, 1454, 1142, 1096, 1010, 840, 820.

1-(3-(1-(benzyloxy)prop-1-en-2-yl)phenyl)ethan-1-one (5ah)

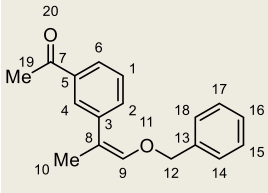
5ah

Following GP XII using **C1** (8.2 mg, 0.015 mmol, 1 mol% to **6a** - 5 mol% to **3h**), benzyl allyl ether **6a** (222 mg, 1.5 mmol, 5 equiv), 3-bromoacetophenone **3h** (59.7 mg, 0.3 mmol, 1 equiv) and triethylamine (1.5 mL, 36 equiv). Purification by two consecutive column chromatography (1. SiO_2 containing 10% $AgNO_3$, pentane/Et_2O 80:20; 2. SiO_2, pentane/Et_2O 80:20) afforded product **5ah** in pure form as colorless oil (40.7 mg, 0.15 mmol, 51% yield, *E/Z* 43:57, $rr_{\alpha/\beta}$ 1:2.7).

TLC: pentane/Et_2O 80:20, R_f = 0.2.

(*Z*): ^{1}H NMR (400 MHz, $CDCl_3$) δ (ppm) = 8.29 (dd, $^4J_{HH}$ = 1.8 Hz, 1H, H-4), 7.85 (ddd, $^3J_{HH}$ = 7.8 Hz, $^4J_{HH}$ = 1.9, 1.1 Hz, 1H, H-2), 7.78 (ddd, $^3J_{HH}$ = 7.8 Hz, $^4J_{HH}$ = 1.8, 1.1 Hz, 1H, H-6), 7.44 – 7.29 (m, 6H, H-1 and H-(14-18)), 6.64 (q, $^4J_{HH}$ = 1.4 Hz, 1H, H-9), 4.92 (s, 2H, H-12), 2.55 (s, 1H, H-12), 1.96 (d, $^4J_{HH}$ = 1.4 Hz, 3H, H-10). **(*E*): ^{1}H NMR** (400 MHz, $CDCl_3$) δ (ppm) = 7.88 (dd, $^4J_{HH}$ = 1.9, 0.5 Hz, 1H, H-4), 7.74 (ddd, $^3J_{HH}$ = 7.7 Hz, $^4J_{HH}$ = 1.8, 1.2 Hz, 1H, H-6), 7.48 (ddd, $^3J_{HH}$ = 7.8 Hz, $^4J_{HH}$ = 2.0, 1.2 Hz, 1H, H-2), 7.44 – 7.29 (m, 6H, H-1 and H-(14-18)), 6.35 (q, $^4J_{HH}$ = 1.4 Hz, 1H, H-9), 4.96 (s, 2H, H-12), 2.60 (s, 3H, H-12), 2.08 (d, $^4J_{HH}$ = 1.4 Hz, 3H, H-10).

(*Z*): ^{13}C{^{1}H} NMR (101 MHz, $CDCl_3$) δ (ppm) = 198.8 (C-7), 143.9 (CH-9), 138.9 (C-3), 137.3 (C-13), 137.0 (C-5), 132.3 (CH-2), 128.7 (CH-1 or CH-15 or CH-16), 128.3 (CH-1 or CH-15 or CH-16), 128.2 (CH-1 or CH-15 or CH-16), 127.8 (CH-4), 127.5 (CH-14), 125.9 (CH-6), 110.7 (C-8), 74.8 (CH_2-12), 26.8 (CH_3-19), 18.3 (CH_3-10). **(*E*): ^{13}C{^{1}H} NMR** (101 MHz, $CDCl_3$) δ (ppm) = 198.5 (C-7), 144.5 (CH-9), 141.3 (C-3), 137.5 (C-5), 137.3 (C-13), 129.7 (CH-2), 128.7 (CH-1 or CH-15 or CH-16), 128.6 (CH-1 or CH-15 or CH-16), 128.2 (CH-1 or CH-15 or CH-16), 127.5 (CH-14), 126.1 (CH-6), 124.7 (CH-4), 114.5 (C-8), 74.4 (CH_2-12), 26.9 (CH_3-19), 12.9 (CH_3-10).

HRMS (ESI +): calculated for $C_{18}H_{18}O_2$ $[M+H]^+$: 267.1380; found: 267.1399.

IR (neat): ν (cm^{-1}) = 2924, 1681, 1646, 1595, 1355, 1272, 1236, 1141, 996, 842.

1-(1-(benzyloxy)prop-1-en-2-yl)-4-methoxybenzene (5ai)

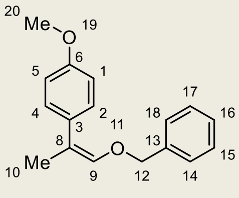

5ai

Following GP XII using **C1** (8.2 mg, 0.015 mmol, 1 mol% to **6a** - 5 mol% to **3i**), benzyl allyl ether **6a** (222 mg, 1.5 mmol, 5 equiv), 4-bromoanisole **3i** (59.7 mg, 0.3 mmol, 1 equiv) and triethylamine (1.5 mL, 36 equiv). The reaction afforded **5ai** in 27% conversion (*E*/*Z* 31:69, $rr_{\alpha/\beta}$ 2:1) assessed by ^{1}H NMR analysis of the crude reaction mixture using an internal standard.

Below are the most relevant signals assigned to **5ai**:

(*Z*): ^{1}H NMR (400 MHz, $CDCl_3$) δ (ppm) = 6.11 (q, $^{4}J_{HH}$ = 1.3 Hz, 1H, H-9), 1.81 (d, $^{4}J_{HH}$ = 1.4 Hz, 3H, H-10). **(*E*): ^{1}H NMR** (400 MHz, $CDCl_3$) δ (ppm) = 6.39 (q, $^{4}J_{HH}$ = 1.4 Hz, 1H, H-9), 1.95 (d, $^{4}J_{HH}$ = 1.4 Hz, 3H, H-10).

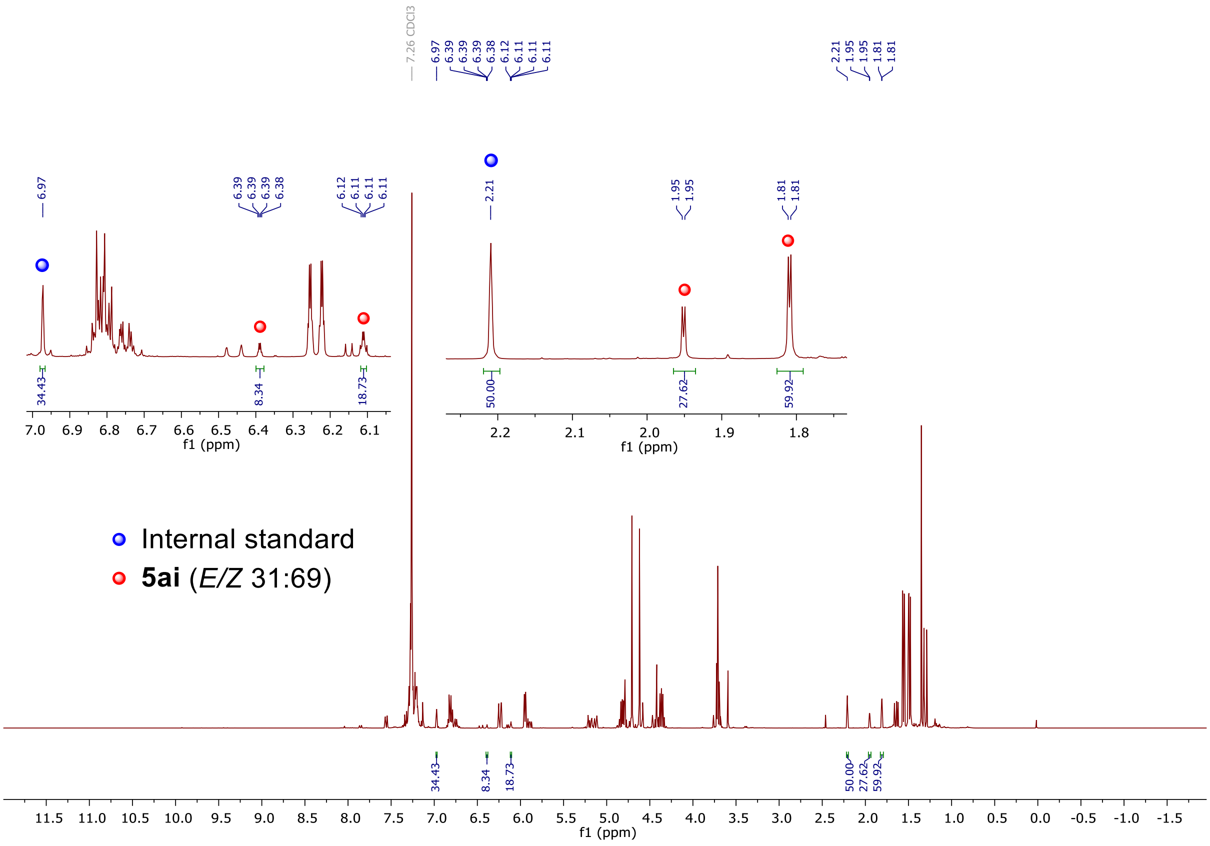

Figure S12. ^{1}H NMR spectrum of the crude reaction mixture.

Methyl 4-(1-(benzyloxy)prop-1-en-2-yl)benzoate (5aq)

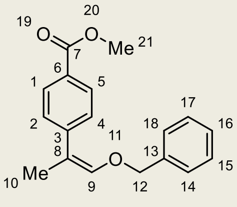

5aq

Following GP XII using **C1** (8.2 mg, 0.015 mmol, 1 mol% to **6a** - 5 mol% to **3q**), benzyl allyl ether **6a** (222 mg, 1.5 mmol, 5 equiv), methyl 4-bromobenzoate **3q** (64.5 mg, 0.3 mmol, 1 equiv) and triethylamine (1.5 mL, 36 equiv). Purification by two consecutive column chromatography (1. SiO_2 containing 10% $AgNO_3$, pentane/Et_2O 90:10; 2. SiO_2, pentane/Et_2O 90:10) afforded product **5aq** in pure form as white solid (58.1 mg, 0.21 mmol, 69% yield, *E/Z* 34:66, $rr_{\alpha/\beta}$ 1:5.3).

TLC: pentane/Et_2O 80:20, R_f = 0.4.

(*Z*): ^{1}H NMR (400 MHz, $CDCl_3$) δ (ppm) = 8.01 – 7.96 (m, 2H, H-1 and H-5), 7.77 – 7.71 (m, 2H, H-2 and H-4), 7.42 – 7.30 (m, 5H, H-(14-18)), 6.36 (q, $^4J_{HH}$ = 1.4 Hz, 1H, H-9), 4.94 (s, 2H, H-12), 3.91 (s, 3H, H-21), 1.93 (d, $^4J_{HH}$ = 1.4 Hz, 3H, H-10). **(*E*): ^{1}H NMR** (400 MHz, $CDCl_3$) δ (ppm) = 7.95 – 7.90 (m, 2H, H-1 and H-5), 7.42 – 7.30 (m, 7H, H-2 and H-4 and H-(14-18)), 6.72 (q, $^4J_{HH}$ = 1.3 Hz, 1H, H-9), 4.97 (s, 2H, H-12), 3.90 (s, 3H, H-21), 2.06 (d, $^4J_{HH}$ = 1.3 Hz, 3H, H-10).

(*Z*): ^{13}C{^{1}H} NMR (101 MHz, $CDCl_3$) δ (ppm) = 167.3 (C-7), 145.0 (CH-9), 143.2 (C-3), 137.3 (C-13), 129.3 (CH-1), 128.8 (CH-15 or CH-16), 128.2 (CH-15 or CH-16), 127.4 (CH-14), 127.4 (CH-2), 127.4 (C-6), 110.6 (C-8), 74.9 (CH_2-12), 52.1 (CH_3-21), 18.3 (CH_3-10). **(*E*): ^{13}C{^{1}H} NMR** (101 MHz, $CDCl_3$) δ (ppm) = 167.2 (C-7), 145.5 (CH-9), 145.5 (C-3), 137.4 (C-13), 129.8 (CH-1), 128.2 (CH-15 or CH-16), 127.5 (CH-14, CH-15 or CH-16), 127.5 (CH-14, CH-15 or CH-16), 127.4 (C-6), 124.6 (CH-2), 114.5 (C-8), 74.5 (CH_2-12), 52.1 (CH_3-21), 12.6 (CH_3-10).

HRMS (ESI +): calculated for $C_{18}H_{18}O_3$ $[M+H]^+$: 283.1329; found: 283.1351.

IR (neat): ν (cm^{-1}) = 2948, 1700, 1637, 1599, 1453, 1431, 1275, 1189, 1153, 1107, 1069, 1004, 866.

m.p.: 39-41 °C.

4-(1-(benzyloxy)prop-1-en-2-yl)benzaldehyde (5ar)

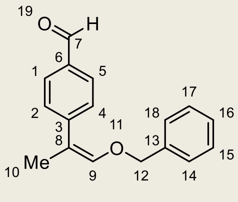

Following GP XII using **C1** (8.2 mg, 0.015 mmol, 1 mol% to **6a** - 5 mol% to **3r**), benzyl allyl ether **6a** (222 mg, 1.5 mmol, 5 equiv), 4-bromobenzaldehyde **3r** (55.5 mg, 0.3 mmol, 1 equiv) and triethylamine (1.5 mL, 36 equiv). Purification by two consecutive column chromatography (1. SiO_2 containing 10% $AgNO_3$, pentane/Et_2O 90:10; 2. SiO_2, pentane/Et_2O 90:10) afforded product **5ar** in pure form as pale yellow oil (53.7 mg, 0.21 mmol, 71% yield, *E/Z* 43:57, $rr_{\alpha/\beta}$ 1:10).

TLC: pentane/Et_2O 80:20, R_f = 0.3.

(*Z*): ^{1}H NMR (400 MHz, $CDCl_3$) δ (ppm) = 9.97 (s, 1H, H-7), 7.86 – 7.80 (m, 5H, H-2 and H-4 and H-1 and H-5), 7.41 – 7.31 (m, 5H, H-(14-18)), 6.41 (q, $^4J_{HH}$ = 1.4 Hz, 1H, H-9), 4.96 (s, 2H, H-12), 1.95 (d, $^4J_{HH}$ = 1.4 Hz, 3H, H-10). **(*E*): ^{1}H NMR** (400 MHz, $CDCl_3$) δ (ppm) = 9.94 (s, 1H, H-7), 7.79 – 7.75 (m, 2H, H-1 and H-5), 7.45 – 7.41 (m, 2H, H-2 and H-4), 7.41 – 7.31 (m, 5H, H-(14-18)), 6.79 (q, $^4J_{HH}$ = 1.3 Hz, 1H, H-9), 4.99 (s, 2H, H-12), 2.07 (d, $^4J_{HH}$ = 1.3 Hz, 3H, H-10).

(*Z*): ^{13}C{^{1}H} NMR (101 MHz, $CDCl_3$) δ (ppm) = 192.1 (CH-7), 145.8 (CH-9), 144.9 (C-3), 137.1 (C-13), 134.0 (C-6), 129.6 (CH-1), 128.8 (CH-15 and CH-16), 128.0 (CH-2), 127.5 (CH-14), 110.4 (C-8), 75.0 (CH_2-12), 18.2 (CH_3-10), **(*E*): ^{13}C{^{1}H} NMR** (101 MHz, $CDCl_3$) δ (ppm) = 191.8 (CH-7), 147.2 (C-3), 146.3 (CH-9), 137.2 (C-13), 134.1 (C-6), 130.1 (CH-1), 128.3 (CH-15 and CH-16), 127.5 (CH-14), 125.0 (CH-2), 114.4 (C-4), 74.6 (CH_2-12), 12.5 (CH_3-10).

HRMS (ESI +): calculated for $C_{17}H_{16}O_2$ $[M+H]^+$: 253.1223; found: 253.1243.

IR (neat): ν (cm^{-1}) = 2922, 1694, 1641, 1595, 1560, 1454, 1217, 1143, 1111, 1067, 823.

4-(1-(benzyloxy)prop-1-en-2-yl)phenyl trifluoromethanesulfonate (5as)

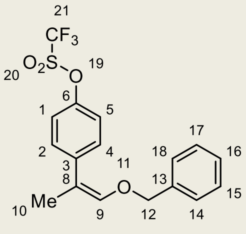
5as

Following GP XII using **C1** (8.2 mg, 0.015 mmol, 1 mol% to **6a** - 5 mol% to **3s**), benzyl allyl ether **6a** (222 mg, 1.5 mmol, 5 equiv), 4-bromophenyl trifluoromethanesulfonate **3s** (91.5 mg, 0.3 mmol, 1 equiv) and triethylamine (1.5 mL, 36 equiv). Purification by two consecutive column chromatography (1. SiO_2 containing 10% $AgNO_3$, pentane/Et_2O 98:2; 2. SiO_2, pentane/Et_2O 98:2) afforded product **5as** in pure form as colorless oil (63.5 mg, 0.17 mmol, 57% yield, *E/Z* 37:63, $rr_{\alpha/\beta}$ 1:3).

TLC: pentane/Et_2O 98:2, R_f = 0.5.

(*Z*): ^{1}H NMR (400 MHz, $CDCl_3$) δ (ppm) = 7.79 – 7.71 (m, 2H, H-2 and H-4), 7.42 – 7.33 (m, 5H, H-(14-18)), 7.25 – 7.17 (m, 2H, H-1 and H-5), 6.33 (q, $^4J_{HH}$ = 1.4 Hz, 1H, H-9), 4.92 (s, 2H, H-12), 1.91 (d, $^4J_{HH}$ = 1.4 Hz, 3H, H-10). **(*E*): ^{1}H NMR** (400 MHz, $CDCl_3$) δ (ppm) = 7.42 – 7.33 (m, 5H, H-(14-18)), 7.33 – 7.30 (m, 2H, H-2 and H-4), 7.20 – 7.12 (m, 2H, H-1 and H-5), 6.58 (q, $^4J_{HH}$ = 1.4 Hz, 1H, H-9), 4.95 (s, 2H, H-12), 2.03 (d, $^4J_{HH}$ = 1.3 Hz, 3H, H-10).

(*Z*): ^{13}C{^{1}H} NMR (101 MHz, $CDCl_3$) δ (ppm) = 147.5 (C-6), 144.4 (C-9), 138.8 (C-3), 137.2 (C-13), 129.3 (CH-2), 128.8 (CH-15 or CH-16), 128.3 (CH-15 or CH-16), 127.5 (CH-14), 120.8 (CH-1), 118.9 (q, $^1J_{CF}$ = 320.8 Hz, CF_3-21), 109.6 (C-8), 74.9 (CH_2-12), 18.3 (CH_3-10). **(*E*): ^{13}C{^{1}H} NMR** (101 MHz, $CDCl_3$) δ (ppm) = 147.8 (C-6), 144.8 (C-9), 141.3 (C-3), 137.4 (C-13), 128.8 (CH-15 or CH-16), 128.3 (CH-15 or CH-16), 127.5 (CH-14), 126.5 (CH-2), 121.2 (CH-1), 118.9 (q, $^1J_{CF}$ = 320.8 Hz, CF_3-21), 113.8 (C-8), 74.4 (CH_2-12), 12.9 (CH_3-10).

(*Z*): ^{19}F{^{1}H} NMR (282 MHz, $CDCl_3$) δ (ppm) = -72.9. **(*E*): ^{19}F{^{1}H} NMR** (282 MHz, $CDCl_3$) δ (ppm) = -72.8.

HRMS (ESI +): calculated for $C_{17}H_{15}F_3O_4S$ $[M+NH_4]^+$: 390.0987; found: 390.0991.

IR (neat): ν (cm^{-1}) = 2938, 1639, 1499, 1418, 1207, 1133, 1110, 883, 832.

3-(1-(benzyloxy)prop-1-en-2-yl)benzonitrile (5at)

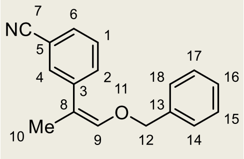
5at

Following GP XII using **C1** (8.2 mg, 0.015 mmol, 1 mol% to **6a** - 5 mol% to **3t**), benzyl allyl ether **6a** (222 mg, 1.5 mmol, 5 equiv), 3-bromobenzonitrile **3t** (54.6 mg, 0.3 mmol, 1 equiv) and triethylamine (1.5 mL, 36 equiv). Purification by two consecutive column chromatography (1. SiO_2 containing 10% $AgNO_3$, pentane/Et_2O 90:10; 2. SiO_2, pentane/Et_2O 90:10) afforded product **5at** in pure form as colorless oil (42.4 mg, 0.17 mmol, 57% yield, *E/Z* 45:55, $rr_{\alpha/\beta}$ 1:4.4).

TLC: pentane/Et_2O 80:20, R_f = 0.2.

(*Z*): ^{1}H NMR (400 MHz, $CDCl_3$) δ (ppm) = 7.98 (dd, $^4J_{HH}$ = 1.5, 1.5 Hz, 1H, H-4), 7.87 (ddd, $^3J_{HH}$ = 7.8 Hz, $^4J_{HH}$ = 1.6, 1.6 Hz, 1H, H-2), 7.46 (dd, $^4J_{HH}$ = 1.5, 1.5 Hz, 1H, H-6), 7.43 – 7.31 (m, 6H, H-1 and H-(14-18)), 6.35 (q, $^4J_{HH}$ = 1.4 Hz, 1H, H-9), 4.93 (s, 2H, H-12), 1.90 (d, $^4J_{HH}$ = 1.4 Hz, 3H, H-10). **(*E*): ^{1}H NMR** (400 MHz, $CDCl_3$) δ (ppm) = 7.54 (dd, $^4J_{HH}$ = 1.5, 1.5 Hz, 1H, H-4), 7.49 (ddd, $^3J_{HH}$ = 7.9 Hz, $^4J_{HH}$ = 2.0, 1.3 Hz, 1H, H-2), 7.43 – 7.31 (m, 7H, H-1 and H-6 and H-(14-18)), 6.62 (q, $^4J_{HH}$ = 1.3 Hz, 1H, H-9), 4.96 (s, 2H, H-12), 2.03 (d, $^4J_{HH}$ = 1.4 Hz, 3H, H-10).

(*Z*): ^{13}C{^{1}H} NMR (101 MHz, $CDCl_3$) δ (ppm) = 144.9 (CH-9), 139.6 (C-3), 137.1 (C-13), 131.8 (CH-2), 131.4 (CH-4), 129.3 (CH-6), 129.2 (CH-6 or CH-15 or CH-16), 128.8 (CH-6 or CH-15 or CH-16), 128.4 (CH-6 or CH-15 or CH-16), 127.5 (CH-14), 119.6 (C-20), 112.1 (C-5), 109.3 (C-8), 74.9 (CH_2-12), 18.0 (CH_3-10). **(*E*): ^{13}C{^{1}H} NMR** (101 MHz, $CDCl_3$) δ (ppm) = 145.2 (CH-9), 142.0 (C-3), 137.2 (C-13), 129.3 (CH-2), 129.2 (CH-1 or CH-6 or CH-15 or CH-16), 128.8 (CH-1 or CH-6 or CH-15 or CH-16), 128.5 (CH-4), 128.4 (CH-1 or CH-6 or CH-15 or CH-16), 128.3 (CH-1 or CH-6 or CH-15 or CH-16), 127.5 (CH-14), 119.3 (C-20), 113.5 (C-8), 112.6 (C-5), 74.5 (CH_2-12), 12.6 (CH_3-10).

HRMS (ESI +): calculated for $C_{17}H_{15}NO$ $[M+NH_4]^+$: 267.1492; found: 267.1500.

IR (neat): ν (cm^{-1}) = 2925, 2228, 1645, 1594, 1454, 1273, 1142, 1083, 991, 858.

1-(1-(benzyloxy)prop-1-en-2-yl)-3-nitrobenzene (5au)

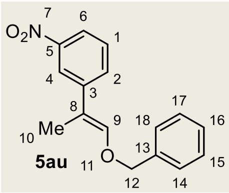

Following GP XII using **C1** (8.2 mg, 0.015 mmol, 1 mol% to **6a** - 5 mol% to **3u**), benzyl allyl ether **6a** (222 mg, 1.5 mmol, 5 equiv), 3-nitrobromobenzene **3u** (60.6 mg, 0.3 mmol, 1 equiv) and triethylamine (1.5 mL, 36 equiv). Purification by two consecutive column chromatography (1. SiO_2 containing 10% $AgNO_3$, pentane/Et_2O 99:1; 2. SiO_2, pentane/Et_2O 99:1) afforded product **5au** in pure form as yellow oil (53.1 mg, 0.20 mmol, 66% yield, *E/Z* 55:45, $rr_{\alpha/\beta}$ 1:5.4).

TLC: pentane/Et_2O 90:10, R_f = 0.2.

(*E*): ^{1}H NMR (400 MHz, $CDCl_3$) δ (ppm) = 8.13 (dd, $^4J_{HH}$ = 2.1, 2.1 Hz, 1H, H-4), 8.04 – 7.94 (m, 1H, H-6), 7.58 (ddd, $^3J_{HH}$ = 7.8 Hz, $^4J_{HH}$ = 1.8, 1.0 Hz, 1H, H-2), 7.49 – 7.31 (m, 6H, H-1 and H-(14-18)), 6.72 (q, $^4J_{HH}$ = 1.4 Hz, 1H, H-9), 4.99 (s, 2H, H-12), 2.08 (d, $^4J_{HH}$ = 1.3 Hz, 3H, H-10). **(*Z*): ^{1}H NMR** (400 MHz, $CDCl_3$) δ (ppm) = 8.59 (dd, $^4J_{HH}$ = 2.1, 2.1 Hz, 1H, H-4), 8.04 – 7.94 (m, 2H, H-2 and H-6), 7.49 – 7.31 (m, 6H, H-1 and H-(14-18)), 6.40 (q, $^4J_{HH}$ = 1.4 Hz, 1H, H-9), 4.96 (s, 2H, H-12), 1.96 (d, $^4J_{HH}$ = 1.3 Hz, 3H, H-10).

(*E*): ^{13}C{^{1}H} NMR (101 MHz, $CDCl_3$) δ (ppm) = 148.6 (C-5), 145.6 (CH-9), 142.5 (C-3), 137.2 (C-13), 130.7 (CH-2), 129.2 (CH-15 or CH-16), 128.8 (CH-1), 128.3 (CH-15 or CH-16), 127.5 (CH-14), 120.6 (CH-6), 119.6 (CH-4), 113.4 (C-8), 74.6 (CH_2-12), 12.7 (CH_3-10). **(*Z*): ^{13}C{^{1}H} NMR** (101 MHz, $CDCl_3$) δ (ppm) = 148.3 (C-5), 145.2 (CH-9), 140.0 (C-3), 137.0 (C-13), 133.4 (CH-2), 128.8 (CH-1), 128.8 (CH-15 or CH-16), 128.3 (CH-15 or CH-16), 127.5 (CH-14), 122.5 (CH-4), 120.7 (CH-6), 109.3 (C-8), 75.0 (CH_2-12), 18.1 (CH_3-10).

HRMS (ESI+): calculated for $C_{16}H_{15}NO_3$ $[M+NH_4]^+$: 287.1391; found: 287.1389.

IR (neat): ν (cm^{-1}) = 2922, 1644, 1523, 1454, 1347, 1143, 1106, 1077, 997, 883.

1-(1-(benzyloxy)prop-1-en-2-yl)-3-methoxybenzene (5av)

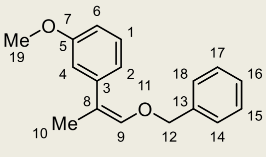

5av

Following GP XII using **C1** (8.2 mg, 0.015 mmol, 1 mol% to **6a** - 5 mol% to **3v**), benzyl allyl ether **6a** (222 mg, 1.5 mmol, 5 equiv), 3-bromoanisole **3v** (56.1 mg, 0.3 mmol, 1 equiv) and triethylamine (1.5 mL, 36 equiv). Purification by two consecutive column chromatography (1. SiO_2 containing 10% $AgNO_3$, pentane/Et_2O 90:10; 2. SiO_2, pentane/Et_2O 90:10) afforded product **5av** in pure form as colorless oil (30.5 mg, 0.12 mmol, 40% yield, *E/Z* 28:72, $rr_{\alpha/\beta}$ 1:1.5).

TLC: pentane/Et_2O 80:20, R_f = 0.2.

(*Z*): ^{1}H NMR (400 MHz, $CDCl_3$) δ (ppm) = 7.39 – 7.28 (m, 6H, H-4 and H-(14-18)), 7.26 – 7.22 (m, 1H, H-2 and H-1), 6.79 – 6.69 (m, 1H, H-6), 6.27 (q, $^4J_{HH}$ = 1.4 Hz, 1H, H-9), 4.90 (s, 2H, H-12), 3.77 (s, 3H, H-19), 1.91 (d, $^4J_{HH}$ = 1.4 Hz, 3H, H-10). **(*E*): ^{1}H NMR** (400 MHz, $CDCl_3$) δ (ppm) = 7.39 – 7.28 (m, 5H, H-(14-18)), 7.19 (dd, $^4J_{HH}$ = 8.0, 8.0 Hz, 1H, H-1), 6.89 (ddd, $^3J_{HH}$ = 7.8 Hz, $^4J_{HH}$ = 1.7, 0.9 Hz, 1H, H-2), 6.83 (dd, $^4J_{HH}$ = 2.5, 1.7 Hz, 1H, H-4), 6.79 – 6.69 (m, 1H, H-6), 6.58 (q, $^4J_{HH}$ = 1.4 Hz, 1H, H-9), 4.93 (s, 2H, H-12), 3.80 (s, 3H, H-19), 2.04 (d, $^4J_{HH}$ = 1.3 Hz, 3H, H-10).

(*Z*): ^{13}C{^{1}H} NMR (101 MHz, $CDCl_3$) δ (ppm) = 159.4 (C-5), 143.4 (CH-9), 139.8 (C-3), 137.6 (C-13), 128.9 (CH-15 or CH-16), 128.7 (CH-1), 128.0 (CH-15 or CH-16), 127.4 (CH-14), 120.2 (CH-2), 113.4 (CH-4), 111.9 (CH-6), 111.3 (C-8), 74.6 (CH_2-10), 55.2 (CH_3-19), 18.6 (CH_3-10). **(*E*): ^{13}C{^{1}H} NMR** (101 MHz, $CDCl_3$) δ (ppm) = 159.8 (C-5), 143.9 (CH-9), 142.3 (C-3), 137.7 (C-13), 129.3 (CH-1), 128.7 (CH-15 or CH-16), 128.1 (CH-15 or CH-16), 127.5 (CH-14), 117.8 (CH-2), 115.2 (C-8), 111.3 (CH-4), 111.1 (CH-6), 74.2 (CH_2-10), 55.3 (CH_3-19), 13.0 (CH_3-10).

HRMS (ESI +): calculated for $C_{17}H_{18}O_2$ $[M+H]^+$: 255.1380; found: 255.1398.

IR (neat): ν (cm^{-1}) = 2933, 1645, 1597, 1574, 1487, 1453, 1427, 1286, 1216, 1138, 1047, 874.

7-(1-(benzyloxy)prop-1-en-2-yl)-3,4-dihydronaphthalen-1(2*H*)-one (5aw)

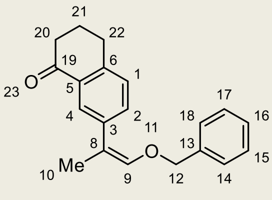

5aw

Following GP XII using **C1** (8.2 mg, 0.015 mmol, 1 mol% to **6a** - 5 mol% to **3w**), benzyl allyl ether **6a** (222 mg, 1.5 mmol, 5 equiv), 7-bromo-3,4-dihydronaphthalen-1(2H)-one **3w** (67.5 mg, 0.3 mmol, 1 equiv) and triethylamine (1.5 mL, 36 equiv). Purification by two consecutive column chromatography (1. SiO_2 containing 10% $AgNO_3$, pentane/Et_2O 80:20; 2. SiO_2, pentane/Et_2O 80:20) afforded product **5aw** in pure form as pale yellow oil (41.6 mg, 0.14 mmol, 47% yield, *E/Z* 37:63, $rr_{\alpha/\beta}$ 1:1.6).

TLC: pentane/Et_2O 80:20, R_f = 0.2.

(*Z*): ^{1}H NMR (400 MHz, $CDCl_3$) δ (ppm) = 8.20 (d, $^4J_{HH}$ = 2.1 Hz, 1H, H-4), 7.92 (dd, $^3J_{HH}$ = 8.1 Hz, $^4J_{HH}$ = 2.1 Hz, 1H, H-2), 7.38 – 7.29 (m, 5H, H-(14-18)), 7.20 (ddd, $^3J_{HH}$ = 8.1 Hz, $^4J_{HH}$ = 0.8, 0.8 Hz, 1H, H-1), 6.27 (q, $^4J_{HH}$ = 1.4 Hz, 1H, H-9), 4.90 (s, 2H, H-12), 2.97 – 2.90 (m, 2H, H-22), 2.69 – 2.60 (m, 2H, H-20), 2.19 – 2.05 (m, 2H, H-21), 1.93 (d, $^4J_{HH}$ = 1.4 Hz, 3H, H-10). **(*E*): ^{1}H NMR** (400 MHz, $CDCl_3$) δ (ppm) = 7.96 (d, $^4J_{HH}$ = 2.0 Hz, 1H, H-4), 7.92 (dd, *J* = 8.1, 2.1 Hz, 1H), 7.40 (dd, $^3J_{HH}$ = 8.0 Hz, $^4J_{HH}$ = 2.2 Hz, 1H, H-2), 7.44 – 7.34 (m, 5H, H-(14-18)), 7.38 – 7.29 (m, 1H), 7.16 (dq, $^3J_{HH}$ = 8.0 Hz, $^4J_{HH}$ = 0.8 Hz, 1H, H-1), 6.63 (q, $^4J_{HH}$ = 1.3 Hz, 1H, H-9), 4.94 (s, 2H, H-12), 2.97 – 2.90 (m, 2H, H-22), 2.69 – 2.60 (m, 2H, H-20), 2.19 – 2.05 (m, 2H, H-21), 2.05 (d, $^4J_{HH}$ = 1.3 Hz, 2H, H-10).

(*Z*): ^{13}C{^{1}H} NMR (101 MHz, $CDCl_3$) δ (ppm) = 198.8 (C-19), 143.4 (CH-9), 142.3 (C-6), 137.5 (C-13), 137.1 (C-3), 133.3 (CH-2), 132.3 (C-5), 128.7 (CH-15 or CH-16), 128.5 (CH-1), 128.1 (CH-15 or CH-16), 127.5 (CH-14), 125.6 (CH-4), 110.8 (C-8), 74.6 (CH_2-12), 39.4 (CH_2-20), 29.6 (CH_2-20), 23.5 (CH_2-21), 18.4 (CH_3-10). **(*E*): ^{13}C{^{1}H} NMR** (101 MHz, $CDCl_3$) δ (ppm) = 198.8 (C-19), 144.2 (CH-9), 142.1 (C-6), 139.3 (C-3), 137.6 (C-13), 132.6 (C-5), 130.1 (CH-2), 128.9 (CH-1), 128.7 (CH-15 or CH-16), 128.1 (CH-15 or CH-16), 127.5 (CH-14), 123.2 (CH-4), 114.4 (C-8), 74.3 (CH_2-12), 39.4 (CH_2-22), 29.5 (CH_2-20), 23.5 (CH_2-21), 12.8 (CH_3-10).

HRMS (ESI +): calculated for $C_{20}H_{20}O_2$ $[M+H]^+$: 293.1536; found: 293.1536.

IR (neat): ν (cm^{-1}) = 2929, 1646, 1668, 1605, 1498, 1453, 1252, 1201, 1129, 996, 814.

(*Z*)-2-(1-(benzyloxy)prop-1-en-2-yl)benzonitrile (5ax)

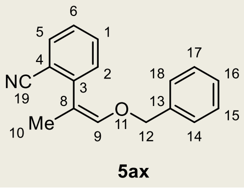

Following GP XII using **C1** (8.2 mg, 0.015 mmol, 1 mol% to **6a** - 5 mol% to **3x**), benzyl allyl ether **6a** (222 mg, 1.5 mmol, 5 equiv), 2-bromobenzonitrile **3x** (54.6 mg, 0.3 mmol, 1 equiv) and triethylamine (1.5 mL, 36 equiv). Purification by two consecutive column chromatography (1. SiO_2 containing 10% $AgNO_3$, pentane/Et_2O 80:20; 2. SiO_2, pentane/Et_2O 80:20) afforded product **5ax** in pure form as colorless oil (23.5 mg, 0.09 mmol, 31% yield, *E/Z* 9:91, $rr_{\alpha/\beta}$ 1:2.5).

TLC: pentane/Et_2O 80:20, R_f = 0.3.

(*Z*): ^{1}H NMR (400 MHz, $CDCl_3$) δ (ppm) = 7.66 (ddd, $^3J_{HH}$ = 7.7 Hz, $^4J_{HH}$ = 1.4, 0.6 Hz, 1H, H-5), 7.53 (ddd, $^3J_{HH}$ = 7.7 Hz, $^4J_{HH}$ = 1.4 Hz, 1H, H-1), 7.37 – 7.27 (m, 7H, H-6 and H-2 and H-(14-18)), 6.30 (q, $^4J_{HH}$ = 1.5 Hz, 1H, H-9), 4.87 (s, 2H, H-12), 1.94 (d, $^4J_{HH}$ = 1.4 Hz, 3H, H-10). **(*Z*): ^{13}C{^{1}H} NMR** (101 MHz, $CDCl_3$) δ (ppm) = 144.0 (C-3), 143.4 (CH-9), 137.1 (C-13), 133.1 (CH-5), 132.5 (CH-1), 129.4 (CH-2), 128.6 (CH-15 or CH-16), 128.1 (CH-15 or CH-16), 127.8 (CH-14), 126.9 (CH-6), 119.2 (C-19), 112.6 (C-4), 111.2 (C-8), 73.9 (CH_2-12), 18.9 (CH_3-10).

HRMS (ESI +): calculated for $C_{17}H_{15}NO$ $[M+NH_4]^+$: 267.1492; found: 267.1500.

IR (neat): ν (cm^{-1}) = 2922, 2224, 1662, 1485, 1454, 1360, 1141, 991, 910.

4-(1-(benzyloxy)prop-1-en-2-yl)-2-methoxypyridine (5ay)

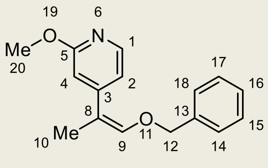

5ay

Following GP XII using **C1** (8.2 mg, 0.015 mmol, 1 mol% to **6a** - 5 mol% to **3y**), benzyl allyl ether **6a** (222 mg, 1.5 mmol, 5 equiv), 4-bromo-2-methoxypyridine **3y** (56.4 mg, 0.3 mmol, 1 equiv) and triethylamine (1.5 mL, 36 equiv). Purification by column chromatography (SiO_2, pentane/Et_2O 80:20) afforded product **5ay** in pure form as pale yellow oil (51.9 mg, 0.20 mmol, 68% yield, *E/Z* 48:52, $rr_{\alpha/\beta}$ >1:20).

TLC: pentane/Et_2O 80:20, R_f = 0.2.

(*Z*): ^{1}H NMR (400 MHz, $CDCl_3$) δ (ppm) = 8.08 (dd, $^3J_{HH}$ = 5.5 Hz, $^5J_{HH}$ = 0.7 Hz, 1H, H-1), 7.41 – 7.30 (m, 5H, H-(14-18)), 7.26 (dd, $^3J_{HH}$ = 5.5 Hz, $^4J_{HH}$ = 1.6 Hz, 1H, H-2), 6.98 (dd, $^4J_{HH}$ = 1.6 Hz, $^5J_{HH}$ = 0.7 Hz, 1H, H-4), 6.38 (q, $^4J_{HH}$ = 1.4 Hz, 1H, H-9), 4.95 (s, 2H, H-12), 3.92 (s, 3H, H-20), 1.87 (d, $^4J_{HH}$ = 1.3 Hz, 3H, H-10). **(*E*): ^{1}H NMR** (400 MHz, $CDCl_3$) δ (ppm) = 7.99 (dd, $^3J_{HH}$ = 5.6 Hz, $^5J_{HH}$ = 0.6 Hz, 1H, H-1), 7.41 – 7.30 (m, 5H, H-(14-18)), 6.81 (q, $^4J_{HH}$ = 1.3 Hz, 1H, H-9), 6.79 (dd, $^3J_{HH}$ = 5.6 Hz, $^4J_{HH}$ = 1.6 Hz, 1H, H-2), 6.60 (dd, $^4J_{HH}$ = 1.6 Hz, $^5J_{HH}$ = 0.7 Hz, 1H, H-4), 4.97 (s, 2H, H-12), 3.93 (s, 3H, H-20), 1.98 (d, $^4J_{HH}$ = 1.3 Hz, 3H, H-10).

(*Z*): ^{13}C{^{1}H} NMR (101 MHz, $CDCl_3$) δ (ppm) = 164.9 (C-5), 148.4 (C-3), 146.8 (CH-9), 146.3 (CH-1), 137.1 (C-13), 128.8 (CH-15 or CH-16), 128.3 (CH-15 or CH-16), 127.5 (CH-14), 116.1 (CH-2), 108.9 (C-8), 108.5 (CH-4), 75.1 (CH_2-12), 53.4 (CH_3-20), 17.6 (CH_3-10). **(*E*): ^{13}C{^{1}H} NMR** (101 MHz, $CDCl_3$) δ (ppm) = 164.8 (C-5), 151.0 (C-3), 146.4 (CH-9), 146.4 (CH-1), 137.1 (C-13), 128.8 (CH-15 or CH-16), 128.3 (CH-15 or CH-16), 127.5 (CH-14), 113.1 (C-8), 112.8 (CH-2), 106.0 (CH-4), 74.6 (CH_2-12), 53.4 (CH_3-20), 11.8 (CH_3-10).

HRMS (ESI +): calculated for $C_{16}H_{17}NO_2$ $[M+H]^+$: 256.1333; found: 256.1342.

IR (neat): ν (cm^{-1}) = 2943, 1646, 1600, 1539, 1483, 1446, 1396, 1378, 1215, 1144, 1040, 988, 819.

Methyl 4-(1-(benzyloxy)prop-1-en-2-yl)thiophene-2-carboxylate (5az)

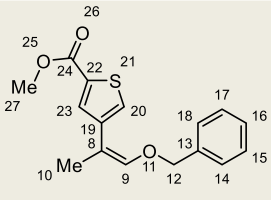

5az

Following GP XII using **C1** (8.2 mg, 0.015 mmol, 1 mol% to **6a** - 5 mol% to **3z**), benzyl allyl ether **6a** (222 mg, 1.5 mmol, 5 equiv), methyl 4-bromothiophene-2-carboxylate **3z** (66.3 mg, 0.3 mmol, 1 equiv) and triethylamine (1.5 mL, 36 equiv). Purification by two consecutive column chromatography (1. SiO_2 containing 10% $AgNO_3$, pentane/Et_2O 90:10; 2. SiO_2, pentane/Et_2O 90:10) afforded product **5az** in pure form as colorless oil (31.6 mg, 0.11 mmol, 37% yield, *E/Z* 50:50, $rr_{\alpha/\beta}$ 1:1).

TLC: pentane/Et_2O 80:20, R_f = 0.4.

(*Z*): ^{1}H NMR (400 MHz, $CDCl_3$) δ (ppm) = 8.16 (d, $^4J_{HH}$ = 1.6 Hz, 1H, H-20), 7.71 (d, $^4J_{HH}$ = 1.6 Hz, 1H, H-23), 7.41 – 7.29 (m, 5H, H-(14-18)), 6.21 (q, $^4J_{HH}$ = 1.4 Hz, 1H, H-9), 4.96 (s, 2H, H-12), 3.88 (s, 3H, H-27), 1.90 (d, $^4J_{HH}$ = 1.4 Hz, 1H, H-10). **(*E*): ^{1}H NMR** (400 MHz, $CDCl_3$) δ (ppm) = 7.78 (d, $^4J_{HH}$ = 1.7 Hz, 1H, H-20), 7.41 – 7.29 (m, 5H, H-(14-18)), 7.15 (d, $^4J_{HH}$ = 1.6 Hz, 1H, H-23), 6.70 (q, $^4J_{HH}$ = 1.4 Hz, 1H, H-9), 4.92 (s, 2H, H-12), 3.87 (s, 3H, H-27), 1.99 (d, $^4J_{HH}$ = 1.4 Hz, 1H, H-10).

(*Z*): ^{13}C{^{1}H} NMR (101 MHz, $CDCl_3$) δ (ppm) = 163.2 (C-24), 143.4 (CH-9), 140.0 (C-19), 137.4 (C-13), 133.8 (CH-20), 132.1 (C-22), 128.2 (CH-15 or CH-16), 128.2 (CH-15 or CH-16), 128.1 (CH-23), 127.4 (CH-14), 110.6 (C-8), 74.7 (CH_2-12), 52.3 (CH_3-27), 17.9 (CH_3-10). **(*E*): ^{13}C{^{1}H} NMR** (101 MHz, $CDCl_3$) δ (ppm) = 162.9 (C-24), 144.2 (CH-9), 142.5 (C-19), 137.4 (C-13), 133.3 (C-22), 130.4 (CH-20), 128.8 (CH-15 or CH-16), 128.7 (CH-15 or CH-16), 127.5 (CH-14), 123.8 (CH-23), 106.8 (C-8), 74.4 (CH_2-12), 52.3 (CH_3-27), 12.7 (CH_3-10).

HRMS (ESI +): calculated for $C_{16}H_{16}O_3S$ $[M+H]^+$: 289.0893; found: 289.0905.

IR (neat): ν (cm^{-1}) = 2921, 1708, 1652, 1444, 1376, 1287, 1250, 1196, 1136, 1096, 1065, 1005, 864.

4-(1-butoxyprop-1-en-2-yl)benzonitrile (5bb)

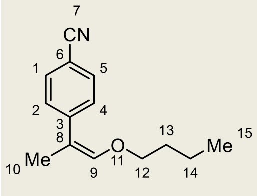

5bb

Following GP XII using **C1** (8.2 mg, 0.015 mmol, 1 mol% to **6b** - 5 mol% to **3b**), allyl butyl ether **6b** (171 mg, 1.5 mmol, 5 equiv), 4-bromobenzonitrile **3b** (54.6 mg, 0.3 mmol, 1 equiv) and triethylamine (1.5 mL, 36 equiv). Purification by two consecutive column chromatography (1. SiO_2 containing 10% $AgNO_3$, pentane/Et_2O 98:2; 2. SiO_2, pentane/Et_2O 98:2) afforded product **5bb** in pure form as colorless oil (34.0 mg, 0.15 mmol, 53% yield, *E/Z* 48:52, $rr_{\alpha/\beta}$ 1:7).

TLC: pentane/Et_2O 90:10, R_f = 0.6.

(*Z*): ^{1}H NMR (400 MHz, $CDCl_3$) δ (ppm) = 7.77 – 7.72 (m, 2H, H-2 and H-4), 7.60 – 7.56 (m, 2H, H-1 and H-5), 6.32 (q, $^4J_{HH}$ = 1.3 Hz, 1H, H-9), 3.89 (t, $^3J_{HH}$ = 6.6 Hz, 2H, H-12), 1.92 (d, $^4J_{HH}$ = 1.3 Hz, 3H, H-10), 1.74 – 1.62 (m, 2H, H-13), 1.51 – 1.36 (m, 2H, H-14), 0.95 (t, $^3J_{HH}$ = 7.4 Hz, 3H, H-15). **(*E*): ^{1}H NMR** (400 MHz, $CDCl_3$) δ (ppm) = 7.56 – 7.52 (m, 2H, H-1 and H-5), 7.39 – 7.34 (m, 2H, H-2 and H-4), 6.65 (q, $^4J_{HH}$ = 1.3 Hz, 1H, H-9), 3.92 (t, $^3J_{HH}$ = 6.5 Hz, 2H, H-12), 1.98 (d, $^4J_{HH}$ = 1.3 Hz, 3H, H-10), 1.74 – 1.62 (m, 2H, H-13), 1.51 – 1.36 (m, 2H, H-14), 0.96 (t, $^3J_{HH}$ = 7.4 Hz, 3H, H-15).

(*Z*): ^{13}C{^{1}H} NMR (101 MHz, $CDCl_3$) δ (ppm) = 146.9 (CH-9), 143.2 (C-3), 131.7 (CH-1), 127.8 (CH-2), 119.7 (C-7), 108.7 (C-6), 108.4 (C-8), 73.7 (CH_2-12), 32.1 (CH_2-13), 19.2 (CH_2-14), 17.9 (CH_3-10), 13.9 (CH_3-15). **(*E*): ^{13}C{^{1}H} NMR** (101 MHz, $CDCl_3$) δ (ppm) = 147.0 (CH-9), 145.8 (C-3), 132.2 (CH-1), 124.9 (CH-2), 119.5 (C-7), 112.7 (C-8), 108.7 (C-6), 73.2 (CH_2-14), 32.0 (CH_2-13), 19.2 (CH_2-14), 13.9 (CH_3-15), 12.1 (CH_3-10).

HRMS (ESI +): calculated for $C_{14}H_{17}NO$ $[M+H]^+$: 216.1383; found: 216.1404.

IR (neat): ν (cm^{-1}) = 2959, 2223, 1642, 1600, 1506, 1464, 1261, 1168, 1113, 1072, 1017, 831.

(*S*)-4-(1-((3,7-dimethyloct-6-en-1-yl)oxy)prop-1-en-2-yl)benzonitrile (5cb)

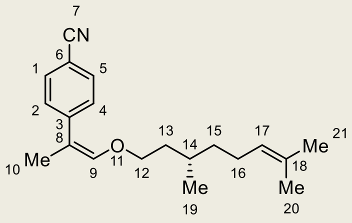

5cb

Following GP XIII using **C1** (8.2 mg, 0.015 mmol, 4.5 mol% to **6c** - 5 mol% to **3b**), allyl ether **6c** (64.8 mg, 0.33 mmol, 1.1 equiv), 4-bromobenzonitrile **3b** (54.6 mg, 0.3 mmol, 1 equiv) and triethylamine (1.5 mL, 36 equiv). Purification by column chromatography (SiO_2, pentane/Et_2O 98:2) afforded product **5cb** in pure form as colorless oil (46.4 mg, 0.15 mmol, 52% yield, *E/Z* 50:50, $rr_{\alpha/\beta}$ >1:20).

TLC: pentane/Et_2O 90:10, R_f = 0.5.

(*Z*): ^{1}H NMR (400 MHz, $CDCl_3$) δ (ppm) = 7.77 – 7.71 (m, 2H, H-2 and H-4), 7.60 – 7.55 (m, 2H, H-1 and H-5), 6.32 (q, $^4J_{HH}$ = 1.3 Hz, 1H, H-9), 5.14 – 5.04 (m, 1H, H-17), 4.03 – 3.85 (m, 2H, H-12), 2.05 – 1.90 (m, 2H, H-16), 1.92 (d, $^4J_{HH}$ = 1.3 Hz, 3H, H-10), 1.80 – 1.70 (m, 1H, H-13), 1.60 (s, 6H, H-20 and H-21), 1.66 – 1.57 (m, 1H, H-14), 1.56 – 1.45 (m, 1H, H-13), 1.42 – 1.31 (m, 1H, H-15), 1.24 – 1.14 (m, 1H, H-15), 0.93 (d, $^3J_{HH}$ = 6.5 Hz, 3H, H-19). **(*E*): ^{1}H NMR** (400 MHz, $CDCl_3$) δ (ppm) = 7.55 – 7.51 (m, 2H, H-1 and H-5), 7.39 – 7.33 (m, 2H, H-2 and H-4), 6.65 (q, $^3J_{HH}$ = 1.3 Hz, 1H, H-9), 5.14 – 5.04 (m, 1H, H-17), 4.03 – 3.85 (m, 2H, H-12), 2.05 – 1.90 (m, 2H, H-16), 1.98 (d, $^4J_{HH}$ = 1.3 Hz, 3H, H-10), 1.80 – 1.70 (m, 1H, H-13), 1.68 (s, 6H, H-20 and H-21), 1.56 – 1.45 (m, 2H, H-13 and H-14), 1.42 – 1.31 (m, 1H, H-15), 1.24 – 1.14 (m, 1H, H-15), 0.94 (d, $^3J_{HH}$ = 6.5 Hz, 3H, H-19).

(*Z*): ^{13}C{^{1}H} NMR (101 MHz, $CDCl_3$) δ (ppm) = 146.9 (CH-9), 143.2 (C-3), 131.7 (CH-1), 131.6 (C-18), 127.8 (CH-2), 124.6 (CH-17), 119.7 (C-7), 108.5 (C-6 and C-8), 72.3 (CH_2-12), 37.1 (CH_2-15), 36.8 (CH_2-13), 29.5 (CH-14), 25.6 (CH_2-16), 19.7 (CH_3-19), 17.9 (CH_3-10), 17.8 (CH_3-20 and CH_3-21). **(*E*): ^{13}C{^{1}H} NMR** (101 MHz, $CDCl_3$) δ (ppm) = 147.0 (CH-9), 145.8 (C-3), 132.3 (CH-1), 131.5 (C-18), 124.9 (CH-2), 124.7 (CH-17), 119.6 (C-7), 112.8 (C-8), 108.7 (C-6), 71.8 (CH_2-12), 37.2 (CH_2-15), 36.9 (CH_2-13), 29.5 (CH-14), 25.9 (CH_3-20 and CH_3-21), 25.6 (CH_2-16), 19.7 (CH_3-19), 12.2 (CH_3-10).

HRMS (ESI +): calculated for $C_{20}H_{27}NO$ $[M+H]^+$: 298.2166; found: 298.2158.

IR (neat): ν (cm^{-1}) = 2920, 2223, 1643, 1601, 1506, 1377, 1164, 1114, 1072, 854, 831.

4-(1-(((3a*R*,5*R*,5a*S*,8a*S*,8b*R*)-2,2,7,7-tetramethyltetrahydro-5*H*-bis([1,3]dioxolo)[4,5-*b*:4',5'-d]pyran-5-yl)methoxy)prop-1-en-2-yl)benzonitrile (5db)

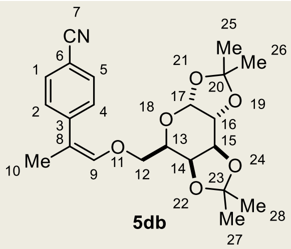

Following GP XIII using **C1** (8.2 mg, 0.015 mmol, 4.5 mol% to **6d** - 5 mol% to **3b**), allyl ether **6d** (99.1 mg, 0.33 mmol, 1.1 equiv), 4-bromobenzonitrile **3b** (54.6 mg, 0.3 mmol, 1 equiv) and triethylamine (1.5 mL, 36 equiv). Purification by column chromatography (SiO_2, pentane/Et_2O 75:25) afforded product **5db** in pure form as colorless oil (46.4 mg, 0.15 mmol, 46% yield, *E/Z* 50:50, $rr_{\alpha/\beta}$ >1:20). Rapid decomposition was observed in less than 10 h at 25 °C.

TLC: pentane/Et_2O 75:25, R_f = 0.3.

(*Z*): ^{1}H NMR (400 MHz, $CDCl_3$) δ (ppm) = 7.78 – 7.73 (m, 2H, H-2 and H-4), 7.59 – 7.55 (m, 2H, H-1 and H-5), 6.34 (q, $^4J_{HH}$ = 1.3 Hz, 1H, H-9), 5.54 (d, $^3J_{HH}$ = 5.0 Hz, 1H, H-17), 4.62 (dd, $^3J_{HH}$ = 7.9, 2.5 Hz, 1H, H-15), 4.33 (dd, $^3J_{HH}$ = 5.0, 2.5 Hz, 1H, H-16), 4.27 (dd, $^3J_{HH}$ = 7.9, 1.5 Hz, 1H, H-14), 4.15 – 3.98 (m, 3H, H-12 and H-13), 1.91 (d, $^4J_{HH}$ = 1.3 Hz, 3H, H-10), 1.51 (s, 3H, H-25 or H-26 or H-27 or H-28), 1.47 (s, 3H, H-25 or H-26 or H-27 or H-28), 1.37 – 1.31 (m, 6H, H-25 or H-26 or H-27 or H-28). **(*E*): ^{1}H NMR** (400 MHz, $CDCl_3$) δ (ppm) = 7.55 – 7.52 (m, 2H, H-1 and H-5), 7.39 – 7.34 (m, 2H, H-2 and H-4), 6.70 (q, $^4J_{HH}$ = 1.3 Hz, 1H, H-9), 5.55 (d, $^3J_{HH}$ = 5.0 Hz, 1H, H-17), 4.64 (dd, $^3J_{HH}$ = 7.9, 2.5 Hz, 1H, H-15), 4.34 (dd, $^3J_{HH}$ = 5.0, 2.5 Hz, 1H, H-16), 4.23 (dd, $^3J_{HH}$ = 7.9, 1.5 Hz, 1H, H-14), 4.15 – 3.98 (m, 3H, H-12 and H-13), 1.98 (d, $^4J_{HH}$ = 1.3 Hz, 3H, H-10), 1.51 (s, 3H, H-25 or H-26 or H-27 or H-28), 1.47 (s, 3H, H-25 or H-26 or H-27 or H-28), 1.37 – 1.31 (m, 6H, H-25 or H-26 or H-27 or H-28).

(*Z*): ^{13}C{^{1}H} NMR (101 MHz, $CDCl_3$) δ (ppm) = 146.3 (CH-9), 143.2 (C-3), 131.7 (CH-1), 128.1 (CH-2), 119.7 (C-7), 109.7 (C-8), 109.7 (C-20 and C-23), 108.9 (C-6), 96.4 (CH-17), 71.8 (CH-16), 71.1 (CH-13), 70.8 (CH-15), 70.7 (CH-16), 67.1 (CH_2-12), 26.2 (CH_3-25 or CH_3-26 or CH_3-27 or CH_3-28), 26.1 (CH_3-25 or CH_3-26 or CH_3-27 or CH_3-28), 25.1 (CH_3-25 or CH_3-26 or CH_3-27 or CH_3-28), 24.5 (CH_3-25 or CH_3-26 or CH_3-27 or CH_3-28), 17.8 (CH_3-10). **(*E*): ^{13}C{^{1}H} NMR** (101 MHz, $CDCl_3$) δ (ppm) = 146.9 (CH-9), 145.6 (C-3), 132.3 (CH-1), 125.1 (CH-2), 119.5 (C-7), 113.7 (C-8), 109.7 (C-20 and C-23), 108.9 (C-6), 96.4 (CH-17), 72.3 (CH-13), 71.1 (CH-14), 70.8 (CH-15), 70.7 (CH-16), 67.1 (CH_2-12), 26.2 (CH_3-25 or CH_3-26 or CH_3-27 or CH_3-28), 26.1 (CH_3-25 or CH_3-26 or CH_3-27 or CH_3-28), 25.1 (CH_3-25 or CH_3-26 or CH_3-27 or CH_3-28), 24.6 (CH_3-25 or CH_3-26 or CH_3-27 or CH_3-28), 12.2 (CH_3-10).

HRMS (ESI +): Not found because of rapid decomposition.

IR (neat): ν (cm^{-1}) = Not measured because of rapid decomposition.

4-(1-((4-methoxybenzyl)oxy)prop-1-en-2-yl)benzonitrile (5eb)

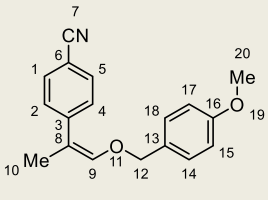

5eb

Following GP XIII using **C1** (8.2 mg, 0.015 mmol, 4.5 mol% to **6e** - 5 mol% to **3b**), allyl ether **6e** (58.8 mg, 0.33 mmol, 1.1 equiv), 4-bromobenzonitrile **3b** (54.6 mg, 0.3 mmol, 1 equiv) and triethylamine (1.5 mL, 36 equiv). Purification by column chromatography (SiO_2, pentane/Et_2O 80:20) afforded product **5eb** in pure form as white solid (43.6 mg, 0.15 mmol, 52% yield, *E/Z* 48:52, $rr_{\alpha/\beta}$ >1:20).

TLC: pentane/Et_2O 80:20, R_f = 0.2.

(*Z*): ^{1}H NMR (400 MHz, $CDCl_3$) δ (ppm) = 7.78 – 7.70 (m, 2H, H-2 and H-4), 7.61 – 7.53 (m, 2H, H-1 and H-5), 7.32 – 7.26 (m, 2H, H-14 and H-18), 6.91 (m, 2H, H-15 and H-17), 6.39 (q, $^4J_{HH}$ = 1.3 Hz, 1H, H-9), 4.87 (s, 2H, H-12), 3.82 (s, 3H, H-20), 1.90 (d, $^4J_{HH}$ = 1.3 Hz, 1H, H-10). **(*E*): ^{1}H NMR** (400 MHz, $CDCl_3$) δ (ppm) = 7.57 – 7.49 (m, 2H, H-1 and H-5), 7.37 – 7.32 (m, 2H, H-2 and H-4), 7.32 – 7.26 (m, 2H, H-14 and H-18), 6.91 (m, 2H, H-15 and H-17), 6.72 (q, $^4J_{HH}$ = 1.4 Hz, 1H, H-9), 4.90 (s, 2H, H-12), 3.82 (s, 3H, H-20), 2.00 (d, $^4J_{HH}$ = 1.4 Hz, 1H, H-10).

(*Z*): ^{13}C{^{1}H} NMR (101 MHz, $CDCl_3$) δ (ppm) = 159.8 (CH-9), 145.9 (C-13), 143.1 (C-3), 131.8 (CH-1), 129.3 (CH-14), 129.0 (C-16), 128.0 (CH-2), 119.5 (C-7), 114.2 (CH-15), 109.5 (C-8), 108.9 (C-6), 74.9 (CH_2-12), 55.4 (CH_3-20), 18.0 (CH_3-10). **(*E*): ^{13}C{^{1}H} NMR** (101 MHz, $CDCl_3$) δ (ppm) = 159.8 (CH-9), 146.2 (C-13), 145.6 (C-3), 132.2 (CH-1), 129.3 (CH-15), 129.2 (C-16), 125.1 (CH-2), 119.6 (C-7), 113.7 (C-8), 108.9 (C-6), 74.5 (CH_2-12), 55.4 (CH_3-20), 12.4 (CH_3-10).

HRMS (ESI +): calculated for $C_{18}H_{17}NO_2$ $[M+H]^+$: 280.1333; found: 280.1344.

IR (neat): ν (cm^{-1}) = 2939, 2221, 1643, 1601, 1514, 1366, 1253, 1147, 1113, 1029, 970, 852, 823.

m.p.: 43-45 °C.

4-(1-(((3*S*,9*S*,10*R*,13*S*,14*S*)-10,13-dimethyl-17-oxo-2,3,4,7,8,9,10,11,12,13,14,15,16,17-tetradecahydro-1*H*-cyclopenta[*a*]phenanthren-3-yl)oxy)prop-1-en-2-yl)benzonitrile (5fb)

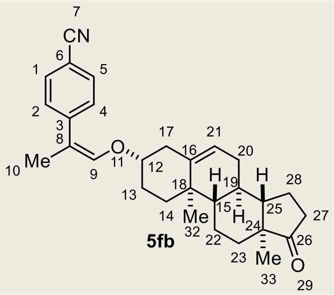

Adapted from GP XIII using **C1** (8.2 mg, 0.015 mmol, 4.5 mol% to **6f** - 5 mol% to **3b**), allyl ether **6f** (108 mg, 0.33 mmol, 1.1 equiv), 4-bromobenzonitrile **3b** (54.6 mg, 0.3 mmol, 1 equiv) and triethylamine (1.5 mL, 36 equiv). Isomerization step performed at 50 °C for 2 h. Purification by column chromatography (SiO_2, gradient from pentane/Et_2O 98:2 to 66:33) afforded product **5fb** in pure form as colorless oil (46.4 mg, 0.15 mmol, 48% yield, *E/Z* 40:60, $rr_{\alpha/\beta}$ 1:5.6). Rapid decomposition was observed in less than 10 h at 25 °C.

TLC: pentane/Et_2O 75:25, R_f = 0.4.

(*Z*): ^{1}H NMR (400 MHz, $CDCl_3$) δ (ppm) = 7.79 – 7.74 (m, 2H, H-2 and H-4), 7.61 – 7.56 (m, 2H, H-1 and H-5), 6.40 (q, $^4J_{HH}$ = 1.3 Hz, 1H, H-9), 5.47 – 5.39 (m, 1H, H-21), 3.74 – 3.58 (m, 1H, H-12), 2.56 – 2.36 (m, 3H, H-17 and H-27), 2.20 – 2.03 (m, 3H, H-20 and H-27), 2.02 – 1.82 (m, 4H, H-28 and H-14 and H-13 and H-23), 1.92 (d, $^4J_{HH}$ = 1.3 Hz, 3H, H-10), 1.74 – 1.61 (m, 3H, H-13 and H-19 and H-22), 1.60 – 1.44 (m, 2H, H-22 and H-28), 1.35 – 1.23 (m, 2H, H-23 and H-25), 1.07 (s, 3H, H-32), 1.15 – 0.95 (m, 2H, H-15 and H-14), 0.89 (s, 3H, H-33). **(*E*): ^{1}H NMR** (400 MHz, $CDCl_3$) δ (ppm) = 7.56 – 7.51 (m, 2H, H-1 and H-5), 7.39 – 7.34 (m, 2H, H-2 and H-4), 6.73 (q, $^4J_{HH}$ = 1.3 Hz, 1H, H-9), 5.47 – 5.39 (m, 1H, H-21), 3.74 – 3.58 (m, 1H, H-12), 2.56 – 2.36 (m, 3H, H-17 and H-27), 2.20 – 2.03 (m, 3H, H-20 and H-27), 1.98 (d, $^4J_{HH}$ = 1.3 Hz, 3H, H-10), 2.02 – 1.82 (m, 4H, H-28 and H-14 and H-13 and H-23), 1.74 – 1.61 (m, 3H, H-13 and H-19 and H-22), 1.60 – 1.44 (m, 2H, H-22 and H-28), 1.35 – 1.23 (m, 2H, H-23 and H-25), 1.07 (s, 3H, H-32), 1.15 – 0.95 (m, 2H, H-15 and H-14), 0.89 (s, 3H, H-33).

(*Z*): ^{13}C{^{1}H} NMR (101 MHz, $CDCl_3$) δ (ppm) = 221.1 (C-26), 145.4 (CH-9), 143.3 (C-3), 140.3 (C-16), 131.8 (CH-1), 127.8 (CH-2), 121.9 (CH-21), 119.7 (C-7), 108.5 (C-6), 108.5 (C-8), 82.8 (CH-12), 51.9 (CH-25), 50.4 (CH-15), 47.7 (C-24), 39.5 (CH_2-17), 37.1 (C-18), 37.1 (CH_2-14), 36.0 (CH_2-27), 31.6 (CH_2-23), 31.6 (CH-19), 30.9 (CH-20), 28.9 (CH_2-13), 22.0 (CH_2-28), 20.5 (CH_2-22), 19.6 (CH_3-32), 18.0 (CH_3-10), 13.7 (CH_3-33). **(*E*): ^{13}C{^{1}H} NMR** (101 MHz, $CDCl_3$) δ (ppm) = 221.1 (C-26), 145.8 (C-3), 145.4 (CH-9), 140.3 (C-16), 132.3 (CH-1), 124.9 (CH-2), 121.9 (CH-21), 119.5 (C-7), 113.1 (C-8), 108.6 (C-6), 82.1 (CH-12), 51.9 (CH-25), 50.4 (CH-15), 47.7 (C-24), 39.5 (CH_2-17), 37.1 (C-18), 37.0 (CH_2-14), 36.0 (CH_2-27), 31.6 (CH_2-23), 31.6 (CH-19), 30.9 (CH-20), 28.9 (CH_2-13), 22.0 (CH_2-28), 20.5 (CH_2-22), 19.6 (CH_3-32), 13.7 (CH_3-33), 12.3 (CH_3-10).

HRMS (ESI +): Not found because of rapid decomposition.

IR (neat): ν (cm^{-1}) = Not measured because of rapid decomposition.

4-(1-(benzyloxy)but-1-en-2-yl)benzonitrile (5gb)

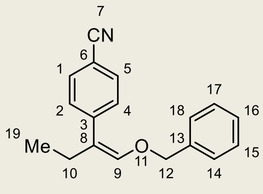

5gb

Following GP XIV using **C1** (16.4 mg, 0.03 mmol, 9 mol% to **6g** - 10 mol% to **3b**), alkenyl ether **6g** (64.8 mg, 0.33 mmol, 1.1 equiv), 4-bromobenzonitrile **3b** (53.5 mg, 0.3 mmol, 1 equiv) and triethylamine (1.5 mL, 36 equiv). Purification by column chromatography (SiO_2, pentane/Et_2O 90:10) afforded product **5gb** in pure form as colorless oil (46.4 mg, 0.15 mmol, 38% yield, *E/Z* 45:55, $rr_{\alpha/\beta}$ >1:20).

<5 % impurity were observed by ^{1}H and ^{13}C{^{1}H } NMR spectroscopy, further purification proved challenging due to coelution of olefinic impurities.

TLC: pentane/Et_2O 80:20, R_f = 0.4.

(*Z*): ^{1}H NMR (400 MHz, $CDCl_3$) δ (ppm) = 7.68 – 7.63 (m, 2H, H-2 and H-4), 7.61 – 7.56 (m, 2H, H-1 and H-5), 7.44 – 7.24 (m, 5H, H-(14-18)), 6.34 (t, $^4J_{HH}$ = 1.2 Hz, 1H, H-9), 4.90 (s, 2H, H-12), 2.34 (qd, $^3J_{HH}$ = 7.4 Hz, $^4J_{HH}$ = 1.2 Hz, 2H, H-10), 0.99 (t, $^3J_{HH}$ = 7.4 Hz, 3H, H-19). **(*E*): ^{1}H NMR** (400 MHz, $CDCl_3$) δ (ppm) = 7.56 – 7.51 (m, 2H, H-1 and H-5), 7.44 – 7.24 (m, 7H, H-2 and H-4 and H-(14-18)), 6.58 (s, 1H, H-9), 4.96 (s, 2H, H-12), 2.58 (q, $^3J_{HH}$ = 7.5 Hz, 2H, H-10), 1.02 (t, $^3J_{HH}$ = 7.4 Hz, 3H, H-19).

(*Z*): ^{13}C{^{1}H} NMR (101 MHz, $CDCl_3$) δ (ppm) = 144.8 (CH-9), 142.4 (C-3), 137.1 (C-13), 131.9 (CH-1), 128.8 (CH-15 or CH-16), 128.7 (CH-2), 128.3 (CH-15 or CH-16), 127.5 (CH-14), 119.6 (C-7), 117.1 (C-8), 109.1 (C-6), 74.9 (CH_2-12), 25.3 (CH_2-10), 14.3 (CH_3-19). **(*E*): ^{13}C{^{1}H} NMR** (101 MHz, $CDCl_3$) δ (ppm) = 145.8 (CH-9), 144.7 (C-3), 137.2 (C-13), 132.3 (CH-1), 128.8 (CH-15 or CH-16), 128.3 (CH-15 or CH-16), 127.5 (CH-14), 125.8 (CH-2), 120.8 (C-8), 119.5 (C-7), 109.1 (C-6), 74.6 (CH_2-12), 19.9 (CH_2-10), 13.1 (CH_3-19).

HRMS (ESI +): calculated for $C_{18}H_{17}NO$ $[M+H]^+$: 264.1383; found: 264.1390.

IR (neat): ν (cm^{-1}) = 2964, 2223, 1638, 1600, 1454, 1261, 1147, 1114, 1094, 1017, 833.

4-(1-(benzyloxy)hex-1-en-2-yl)benzonitrile (5hb)

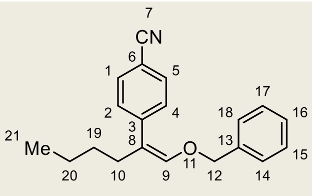

Following GP XIV using **C1** (16.4 mg, 0.03 mmol, 9 mol% to **6h** - 10 mol% to **3b**), allyl ether **6h** (171 mg, 0.33 mmol, 1.1 equiv), 4-bromobenzonitrile **3b** (54.6 mg, 0.3 mmol, 1 equiv) and triethylamine (1.5 mL, 36 equiv). Purification by column chromatography (SiO_2, pentane/Et_2O 90:10) afforded product **5hb** in pure form as colorless oil (46.4 mg, 0.15 mmol, 29% yield, *E/Z* 50:50, $rr_{\alpha/\beta}$ >1:20).

<5 % impurity were observed by ^{1}H and ^{13}C{^{1}H } NMR spectroscopy, further purification proved challenging due to coelution of olefinic impurities.

TLC: pentane/Et_2O 80:20, R_f = 0.5.

(Z): ^{1}H NMR (400 MHz, $CDCl_3$) δ (ppm) = 7.66 – 7.62 (m, 2H, H-2 and H-4), 7.60 – 7.56 (m, 2H, H-1 and H-5), 7.41 – 7.28 (m, 5H, H-(14-18)), 6.32 (t, $^4J_{HH}$ = 1.1 Hz, 1H, H-9), 4.89 (s, 2H, H-12), 2.35 – 2.23 (m, 2H, H-10), 1.41 – 1.19 (m, 4H, H-19 and H-20), 0.88 (t, $^3J_{HH}$ = 7.2 Hz, 3H, H-21). **(*E*): ^{1}H NMR** (400 MHz, $CDCl_3$) δ (ppm) = 7.55 – 7.51 (m, 2H, H-1 and H-5), 7.41 – 7.28 (m, 7H, H-2 and H-4 and H-(14-18)), 6.58 (s, 1H, H-9), 4.95 (s, 2H, H-12), 2.59 – 2.52 (m, 2H, H-10), 1.41 – 1.19 (m, 4H, H-19 and H-20), 0.85 (t, $^4J_{HH}$ = 7.2 Hz, 3H, H-21).

(Z): ^{13}C{^{1}H} NMR (101 MHz, $CDCl_3$) δ (ppm) = 145.1 (CH-9), 142.5 (C-3), 137.1 (C-13), 131.9 (CH-1), 128.8 (CH-15 or CH-16), 128.7 (CH-2), 128.3 (CH-15 or CH-16), 127.5 (CH-14), 119.5 (C-7), 115.6 (C-8), 109.1 (C-6), 74.9 (CH_2-12), 31.8 (CH_2-10), 31.2 (CH_2-19 or CH_2-20), 22.7 (CH_2-19 or CH_2-20), 14.1 (CH_3-21). **(*E*): ^{13}C{^{1}H} NMR** (101 MHz, $CDCl_3$) δ (ppm) = 146.1 (CH-9), 145.0 (C-3), 137.1 (C-13), 132.3 (CH-1), 128.8 (CH-15 or CH-16), 128.3 (CH-15 or CH-16), 127.5 (CH-14), 125.9 (CH-2), 119.6 (C-8), 119.5 (C-7), 109.1 (C-6), 74.6 (CH_2-12), 30.4 (CH_2-19 or CH_2-20), 26.2 (CH_2-10), 22.3 (CH_2-19 or CH_2-20), 14.0 (CH_3-21).

HRMS (ESI +): calculated for $C_{20}H_{21}NO$ $[M+H]^+$: 292.1696; found: 292.1701.

IR (neat): ν (cm^{-1}) = 2929, 2224, 1639, 1601, 1454, 1262, 1146, 1108, 1016, 834.

9. Hammett plot

Table S9. Hammett σ⁻ values and calculation of Log(β/α) for various substituents.

R	σ^-	β/α	log(β/α)
4-NO_2	1.27	21	1.322219295
4-CH(O)	1.03	11.8	1.071882007
4-CN	1.00	16	1.204119983
4-C(O)Me	0.84	7.1	0.851258349
4-C(O)OMe	0.75	5.3	0.72427587
4-CF_3	0.74	7.3	0.86332286
3-NO_2	0.71	5.4	0.73239376
3-CN	0.56	4.4	0.643452676
4-OTf	0.49	3	0.477121255
3-C(O)Me	0.38	2.7	0.431363764
4-Cl	0.19	2.1	0.322219295
3-OMe	0.12	1.5	0.176091259
H	0	1.4	0.146128036
4-Me	-0.17	0.9	-0.045757491
4-OMe	-0.26	0.66	-0.180456064

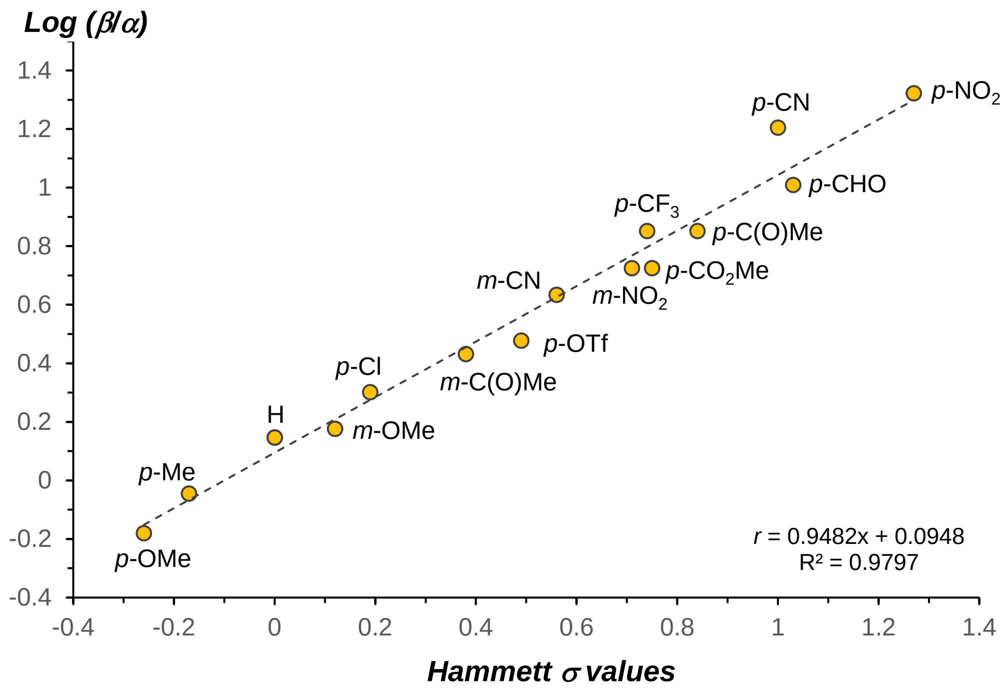

Figure S13. Plot of Log(β/α) against Hammett σ⁻ values.

10. Computational studies

Conformational Sampling. Conformational searches were run for all species with a significant degree of conformational freedom, using the Grimme group's CREST software with the GFN2-xTB method.[22,23] Weak constraints (k = 0.05 Hartree/Bohr^2) were applied to maintain the coordination geometry around the metal center on a case by case basis. For transition states, the relevant coordinate (*e.g*. bond distance) was constrained during the conformational sampling. The ensembles obtained were further sorted using the implemented principle component analysis (PCA) and k-Means sorting clustering algorithm to generate a representative set of 10 geometries. These geometries were further optimized at the DFT level (described thereafter) and the lowest energy conformer was kept for the energy profile.

Geometry optimizations. All DFT calculations were carried out using the ORCA 5.0.3 package.[24] Geometries were optimized using Grimme's dispersion corrected ωB97X-D3 functional,[25,26] in conjunction with the def2-mTZVPP basis set on all atoms and the associated def2-ECP effective core potential on Pd.[27] The RIJCOSX approximation was used to reduce the computational cost of calculations using the def2/J auxiliary basis set and integration grids set to DefGrid3.[28] The Conductor-like Polarizable Continuum Model (C-PCM) was used to account for solvent effects in THF.[29] All stationary points were verified to be minima (zero imaginary frequency) or transition states (one imaginary frequency) by frequency analysis at the same level of theory.

Thermodynamics corrections. Thermodynamics were computed within the Rigid-Rotor-Harmonic-Oscillator (RRHO) approximation at 298.15K. These computed free energies at a 1 atm standard state were corrected to a 1 M solution standard state by a constant ΔG_{SS} = $RT\ln(24.46)$ = 1.89 kcal/mol, where 24.46 L/mol is the molar volume at 1 atm and 298.15 K.

Single point energy corrections. Single point energy calculations were carried out at the DLPNO-CCSD(T) level of theory with TightPNO settings,[30] in conjunction with the def2-TZVPP basis set associated with def2-ECP effective core potential on Pd and the def2-TZVPP/C auxiliary basis set,[29,31,32] in THF using the default C-PCM implementation in ORCA 5.0.3 and using DefGrid3.

Atomic properties. Natural Population Analysis (NPA) charges were calculated with the NBO 6.0 software package.[33] Fukui orbital-weighted dual descriptor indices were calculated using the Multiwfn 3.8 software.[34] Fukui indices (orbital weight parameter Δ = 0.1 a.u.) and NPA charges were obtained on both fragments defined in the decomposition scheme (*vide infra*), in their in-adduct geometry and using the Hartree Fock reference wavefunction obtained during the DLPNO-CCSD(T) single point calculations.

10.1. Basis set convergence – DLPNO-CCSD(T) single points

The basis set convergence at the DLPNO-CCSD(T) level was verified on the barrier associated with the key transition state $\beta TS_{B.4\text{-}5}$. Overall, results were found to be within 1 kcal/mol of the highest basis set trialed (def2-QZVPP), and def2-TZVPP was chosen as a compromise between cost and accuracy for the full energetic profiles.

Table S10. Basis set convergence study.

	basis set			
	def2-TZVP	def2-TZVPP	def2-QZVP	def2-QZVPP
(*E*)-5i	-232.020125	-232.042540	-232.108378	-232.108378
B.3	-3837.134699	-3837.398162	-3837.807261	-3838.066578
$\beta TS_{B.4\text{-}5}$	-4069.132070	-4069.419454	-4069.892418	-4070.151940
Δ*E* (kcal/mol)	14.3	13.3	14.6	14.4

10.2. Calculated thermodynamics

10.2.1. P*t*Bu₃ system – *E*-alkene

Table S11. Calculated thermodynamic data for the Heck coupling of 3b with (*E*)-5i in the conditions for β-regioselectivity.

	$E_{CCSD(T)}$	$(G\text{-}E_{el})_{DFT}$	G(total)
3b	-2896.019295	0.059834	-2895.959461
P*t*Bu$_3$	-813.573103	0.333982	-813.239121
B.1	-1754.942488	0.686687	-1754.255800
TS$_{B.1\text{-}2}$	-4650.941149	0.769842	-4650.171307
B.2	-3837.362760	0.411157	-3836.951603
TS$_{B.2\text{-}3}$	-3837.349971	0.410576	-3836.939394
B.3	-3837.398162	0.413624	-3836.984538
(*E*)-5i	-232.042540	0.086806	-231.955734
αTS$_{B.3\text{-}4}$	-4069.439149	0.522816	-4068.916333
βTS$_{B.3\text{-}4}$	-4069.440749	0.522222	-4068.918527
αB.4	-4069.446013	0.525272	-4068.920741
βB.4	-4069.448845	0.523370	-4068.925475
αTS$_{B.4\text{-}5}$	-4069.420008	0.525258	-4068.894750
βTS$_{B.4\text{-}5}$	-4069.419454	0.522961	-4068.896493
αB.5	-4069.465050	0.522580	-4068.942470
αB.5'	-4069.470461	0.523138	-4068.947322
βB.5	-4069.471330	0.523274	-4068.948056
αTS$_{B.5\text{-}6}$	-4069.462770	0.520277	-4068.942493
αTS$_{B.5'\text{-}6'}$	-4069.464021	0.520523	-4068.943497
βTS$_{B.5\text{-}6}$	-4069.465483	0.520715	-4068.944768
αB.6•[Pd-H]	-4069.467844	0.520631	-4882.186335
αB.6’•[Pd-H]	-4069.465666	0.521078	-4882.183709
βB.6•[Pd-H]	-4069.476774	0.520723	-4882.195173
αTS$_{B.6•[Pd\text{-}H]\text{-}B.6}$	-4069.461229	0.520000	-4882.180350
αTS$_{B.6’•[Pd\text{-}H]\text{-}B.6’}$	-4069.450429	0.518515	-4882.171034
βTS$_{B.6•[Pd\text{-}H]\text{-}B.6}$	-4069.465668	0.518789	-4882.186000
αB.6	-554.767990	0.158920	-554.609070
αB.6'	-554.760151	0.159572	-554.600579
βB.6	-554.769772	0.159031	-554.610741
[(*t*Bu$_3$P)$_2$Pd(H)(Br)]	-4328.303111	0.698020	-4327.605092
3i	-2918.279053	0.093163	-2918.185890
MeO_B.3	-3859.653974	0.447020	-3859.206954
MeO_αTS$_{B.4\text{-}5}$	-4091.679378	0.558573	-4091.120805
MeO_βTS$_{B.4\text{-}5}$	-4091.675976	0.556280	-4091.119696

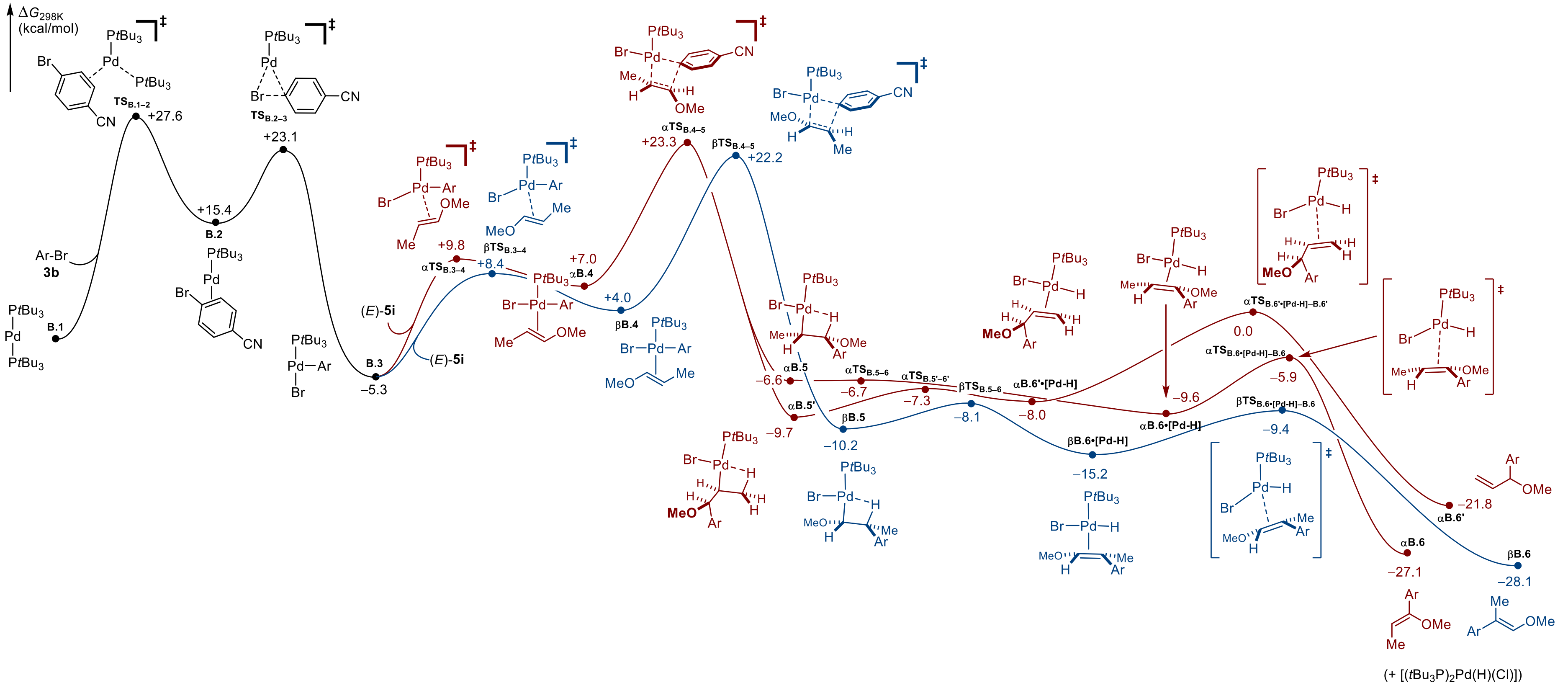

Figure S14. Complete computed free energy reaction profile (kcal/mol) for the Pd-catalyzed β-selective arylation of substituted (*E*)-configured enol ethers starting from $[Pd(PtBu_3)_2]$ (Ar = 4-CN-C_6H_4).

10.2.2. P*t*Bu₃ system – *Z*-alkene

Table S12. Calculated thermodynamic data for the Heck coupling of 3b with (*Z*)-**5i** in the conditions for β-regioselectivity.

	$E_{CCSD(T)}$	$(G\text{-}E_{el})_{DFT}$	G(total)
(*Z*)-5i	-232.043382	0.085867	-231.957515
$TS_{BZ.3\text{-}4}$	-4069.439751	0.521271	-4068.918479
BZ.4	-4069.448666	0.524143	-4068.924523
$\alpha TS_{BZ.4\text{-}5}$	-4069.415119	0.522701	-4068.892419
$\beta TS_{BZ.4\text{-}5}$	-4069.417983	0.523368	-4068.894616
αBZ.5	-4069.464079	0.523211	-4068.940868
αBZ.5'	-4069.472577	0.523939	-4068.948638
βBZ.5	-4069.467987	0.522653	-4068.945335
$\alpha TS_{BZ.5\text{-}6}$	-4069.461073	0.520409	-4068.940664
$\alpha TS_{BZ.5'\text{-}6'}$	-4069.465356	0.522350	-4068.943006
$\beta TS_{BZ.5\text{-}6}$	-4069.463446	0.520467	-4068.942979
αBZ.6	-554.768510	0.160168	-554.608342
αBZ.6'	-554.760151	0.159572	-554.600579
βBZ.6	-554.768752	0.159705	-554.609047
$[(tBu_3P)_2Pd(H)(Br)]$	-4328.303111	0.698020	-4327.605092

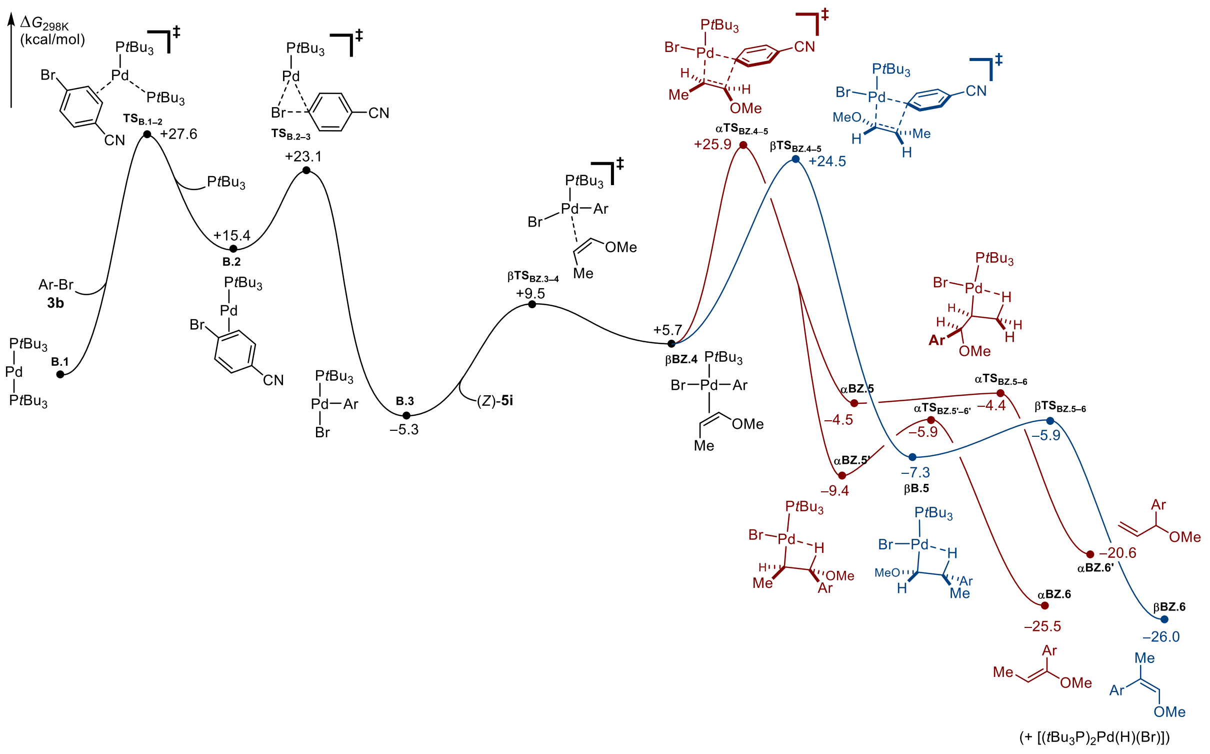

Figure S15. Computed free energy reaction profile (kcal/mol) for the Pd-catalyzed β-selective arylation of substituted (*Z*)-configured enol ethers starting from [Pd(P*t*Bu$_3$)$_2$] (Ar = 4-CN-C$_6$H$_4$).

10.2.3. dppp system – *E*-alkene

Table S13. Calculated thermodynamic data for the Heck coupling of **3b** with (*E*)-**5i** in the conditions for α-regioselectivity.

	$E_{CCSD(T)}$	$(G-E_{el})_{DFT}$	G(total)
$[Na(THF)_3][OTf]$	-1818.793479	0.328307	-1818.465172
$[Na(THF)_3][OAc]$	-1086.541344	0.354469	-1086.186875
A.0	-3135.949021	0.510786	-3135.438235
A.1	-2403.707176	0.536246	-2403.170930
(*E*)-5i	-232.042540	0.086806	-231.955734
αA.2	-2635.748470	0.643905	-2635.104565
βA.2	-2635.746230	0.645365	-2635.100865
$\alpha TS_{A.2-3}$	-2635.725673	0.645344	-2635.080329
$\beta TS_{A.2-3}$	-2635.718460	0.646078	-2635.072381
αA.3	-2635.754878	0.644710	-2635.110169
αA.3'	-2635.755768	0.646116	-2635.109652
βA.3	-2635.755387	0.645387	-2635.110000
$\alpha TS_{A.3-4}$	-2635.748267	0.642625	-2635.105642
$\alpha TS_{A.3'-4'}$	-2635.740431	0.642092	-2635.098340
$\beta TS_{A.3-4}$	-2635.741917	0.644039	-2635.097878
αA.4•[Pd-H]	-2635.749928	0.642844	-4453.572256
αA.4'•[Pd-H]	-2635.741661	0.642588	-4453.564245
βA.4•[Pd-H]	-2635.751800	0.643717	-4453.573255
$\alpha TS_{A.4•[Pd-H]-A.4}$	-2635.730268	0.642662	-4453.552778
$\alpha TS_{A.4'•[Pd-H]-A.4'}$	-2635.738787	0.643449	-4453.560510
$\beta TS_{A.4•[Pd-H]-A.4}$	-2635.747122	0.643821	-4453.568472
αA.4	-554.767990	0.158920	-554.609070
αA.4'	-554.760151	0.159572	-554.600579
βA.4	-554.769772	0.159031	-554.610741
[(dppp)Pd(H)(OAc)]	-2080.975940	0.459578	-2080.516363

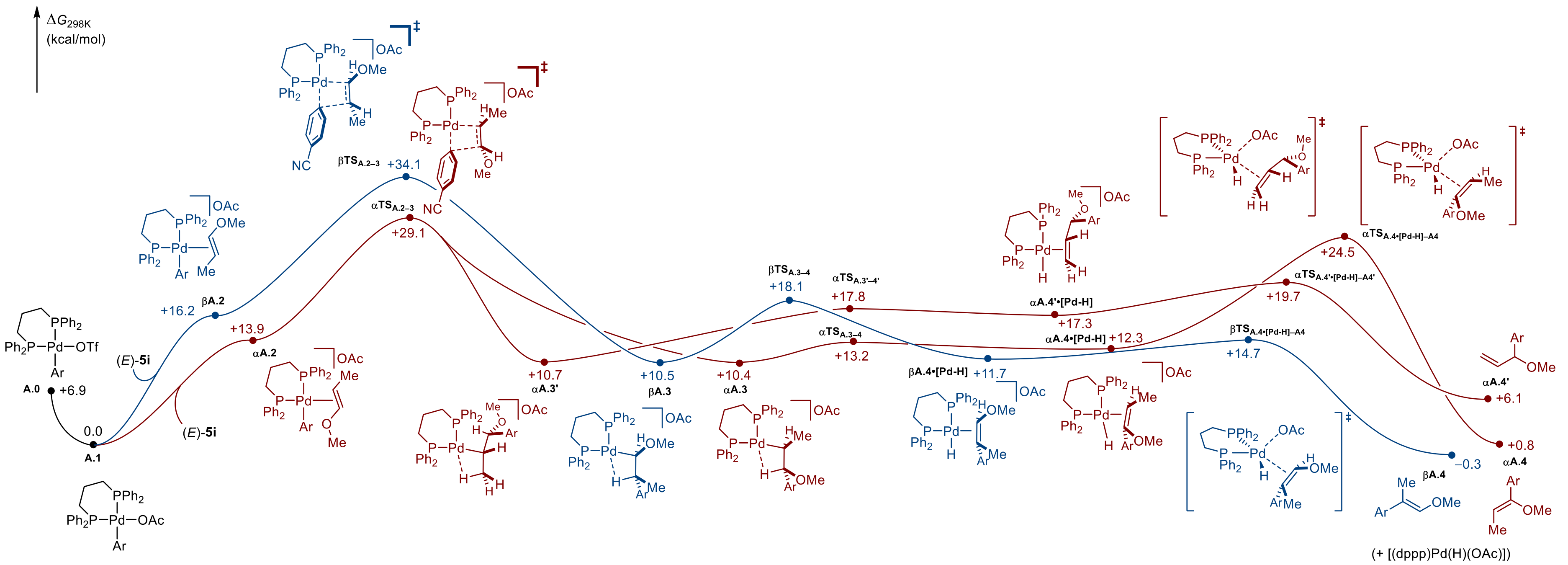

Figure S16. Complete computed free energy reaction profile (kcal/mol) for the Pd-catalyzed β-selective arylation of substituted (*E*)-configured enol ethers starting from [(dppp)Pd(Ar)(OTf)] (Ar = 4-CN-C_6H_4).

10.2.4. dppp system – *Z*-alkene

Table S14. Calculated thermodynamic data for the Heck coupling of **3b** with (*Z*)-**5i** in the conditions for α-regioselectivity.

	$E_{CCSD(T)}$	$(G\text{-}E_{el})_{DFT}$	G(total)
(*Z*)-5i	-232.043382	0.085867	-231.957515
AZ.2	-2635.751119	0.644388	-2635.106731
α$TS_{AZ.2\text{-}3}$	-2635.726085	0.646129	-2635.079956
β$TS_{AZ.2\text{-}3}$	-2635.719211	0.646836	-2635.072375
αAZ.3	-2635.752007	0.644302	-2635.107705
αAZ.3'	-2635.759395	0.645833	-2635.113562
βAZ.3	-2635.751669	0.645251	-2635.106418
α$TS_{AZ.3\text{-}4}$	-2635.743500	0.641738	-2635.101762
α$TS_{AZ.3'\text{-}4'}$	-2635.745939	0.643501	-2635.102437
β$TS_{AZ.3\text{-}4}$	-2635.739477	0.643297	-2635.096180
αAZ.4	-554.768510	0.160168	-554.608342
αAZ.4'	-554.760151	0.159572	-554.600579
βAZ.4	-554.768752	0.159705	-554.609047
[(dppp)Pd(H)(OAc)]	-2080.975940	0.459578	-2080.516363

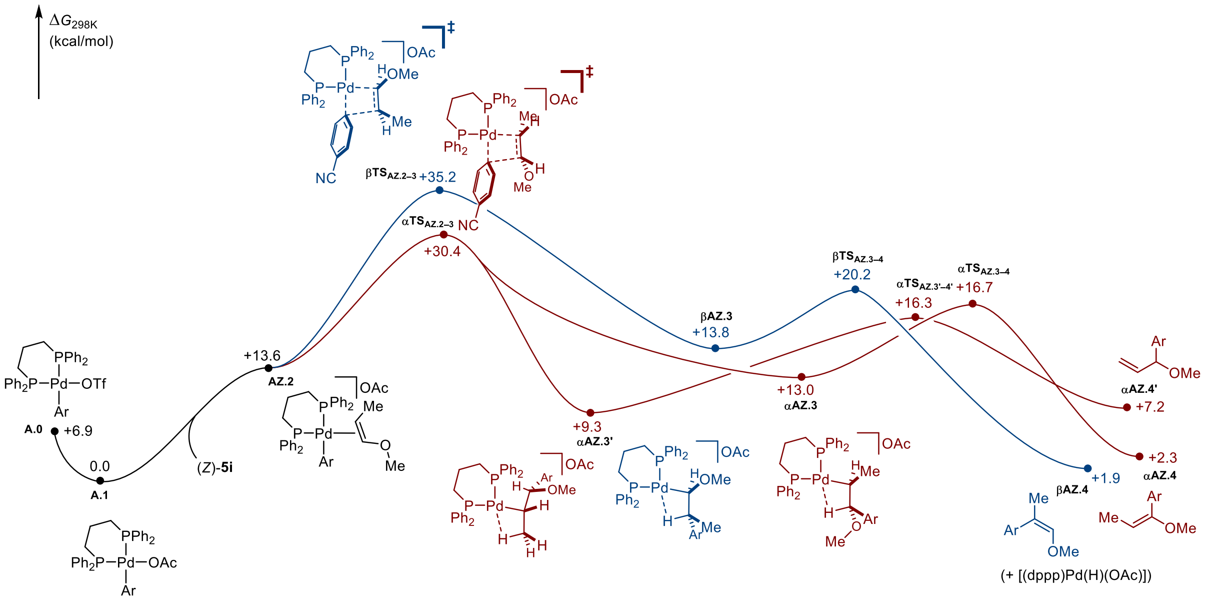

Figure S17. Computed free energy reaction profile (kcal/mol) for the Pd-catalyzed α-selective arylation of substituted (*Z*)-configured enol ethers starting from [(dppp)Pd(Ar)(OTf)] (Ar = 4-CN-C_6H_4).

10.3. Energy decomposition analysis – LED scheme

The binding energy components between the fragment {Pd(PtBu$_3$)(Br)(Ar)} and the alkene moiety at the transition state geometry were decomposed using the Local Energy Decomposition (LED) analysis method, as implemented in ORCA 5.0.3.[35,36] and described in the manual in section 9.42.6.

Within this scheme, the total binding energy can be expressed as:

$$\mathbf{\Delta E_{bind} = \Delta E_{distort} + \Delta E_{int}}$$

where $\mathbf{\Delta E_{distort}}$ represents the energy necessary to distort the fragments from their relaxed equilibrium geometry to the geometry they adopt in the adduct. Thus, $\mathbf{\Delta E_{int}}$ is the interaction energy between the two fragments and can be partitioned into:

$$\mathbf{\Delta E_{int} = \Delta E_{int}^{HF} + \Delta E_{int}^{C}}$$

where $\mathbf{\Delta E_{int}^{HF}}$ corresponds to the Hartree-Fock component of the interaction and $\mathbf{\Delta E_{int}^{C}}$ is the correlation component which contains contributions from the DLPNO-CCSD energy and from the triples correction $\mathbf{\Delta E_{int}^{C\text{-}(T)}}$.

$$\mathbf{\Delta E_{int}^{C} = \Delta E_{int}^{C\text{-}CCSD} + \Delta E_{int}^{C\text{-}(T)}}$$

The correlation component is further decomposed into dispersive $\mathbf{E_{dispersion}}$ and non-dispersive contributions $\mathbf{\Delta E_{residual}}$.

On the other hand, $\mathbf{\Delta \mathit{E}_{int}^{HF}}$ is partitioned as the following:

$$\mathbf{\Delta E_{int}^{HF} = \Delta E_{el\text{-}prep}^{HF} + E_{elstat} + E_{exch}}$$

where $\mathbf{\Delta E_{el\text{-}prep}^{HF}}$ represents the energy required to distort the electronic structure from its ground state in the fragments' isolated equilibrium geometries to the electronic structure they adopt in the adduct, $\mathbf{E_{elstat}}$ is the electrostatic interaction between the distorted electron densities and $\mathbf{E_{exch}}$ is the interfragment exchange interaction.

As described in the literature,[35] the LED components of the reference HF energy were further decomposed into an orbital relaxation term as well as electrostatics and Pauli (sterics) contributions from the frozen state. The latter is defined as the adduct formed of the two fragments and their respective wavefunction in isolated state, *i.e.* before orbital relaxation effects are allowed to occur. Thus, in the frozen state we obtain:

$$\mathbf{\Delta E_{int}^{HF,0} = \Delta E_{el\text{-}prep}^{HF,0} + E_{elstat}^{0} + E_{exch}^{0}}$$

The scheme can subsequently be likened to a Morokuma style EDA scheme by defining $\mathbf{\Delta \mathit{E}_{orb}}$ as the term by term difference between frozen and relaxed states:

$$\mathbf{\Delta E_{orb} = (E_{exch} - E_{exch}^{0}) + (E_{elstat} - E_{elstat}^{0}) + (\Delta E_{el\text{-}prep}^{HF} - \Delta E_{el\text{-}prep}^{HF,0})}$$

and by assimilating the terms

$$\mathbf{\Delta E_{Pauli} \approx \Delta E_{el\text{-}prep}^{HF,0} + E_{exch}^{0}}$$

$$\mathbf{\Delta E_{elstat} \approx E_{elstat}^{0}}$$

The decomposition scheme then becomes:

$$\mathbf{\Delta E_{int} = \Delta E_{elstat} + \Delta E_{Pauli} + \Delta E_{orb} + \Delta E_{disp} + \Delta E_{residual}}$$

ΔE_{orb}, which represents the stabilization upon orbital relaxation, can also be analyzed using the extended transition state method and the natural orbitals for chemical valence theory (ETS-NOCV).[37,38]

To complete the scheme, and for direct comparison to the obtained absolute barriers, thermal corrections and solvation contributions were added such that:

$$\mathbf{\Delta G^{\ddagger}_{TS} = \Delta E_{int} + \Delta E_{distort} + \Delta\Delta G_{solv} + \Delta G_{thermo}}$$

where **$\Delta\Delta G_{solv}$** is defined as the difference between the solvation free energy of the supermolecule and the solvation free energy of the isolated fragments, and **ΔG_{thermo}** represents all the enthalpy and entropy corrections calculated at the optimization level and pertaining to the elementary reaction:

[Pd(PtBu$_3$)(Br)(Ar)] + alkene → [Pd(PtBu$_3$)(Br)(Ar)(alkene)].

The scheme may be summarized as follows:

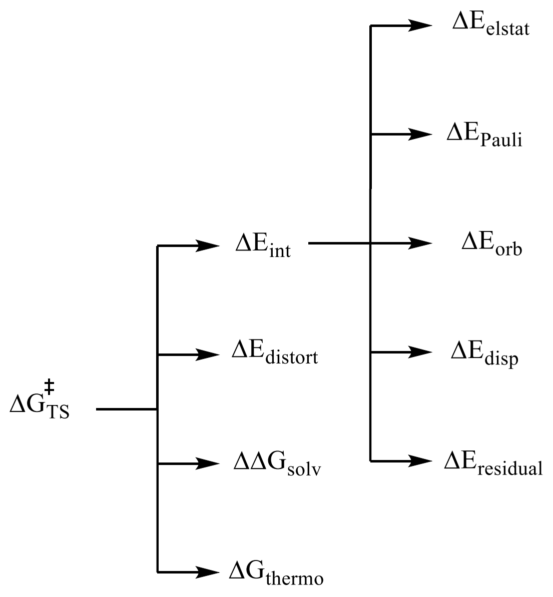

Figure S18. Overview of the energy decomposition scheme.

Overall:

Table S15. Energy decomposition analysis for {Pd(PtBu$_3$)(Br)(Ar)} + alkene ➞ **TS$_{B.4-5}$** (kcal/mol)

$$\Delta G^{\ddagger}_{TS} = \Delta E_{elstat} + \Delta E_{orb} + \Delta E_{distort} + \Delta E_{Pauli} + \Delta G_{thermo} + \Delta\Delta G_{solv} + \Delta E_{disp} + \Delta E_{residual}$$

	R = CN		R = OMe	
	**αTS$_{B.4-5}$**	**βTS$_{B.4-5}$**	**αTS$_{B.4-5}$**	**βTS$_{B.4-5}$**
**ΔG‡$_{TS}$**	**28.6**	**27.5**	**26.3**	**27.0**
ΔE_{elstat}	-176.0	-167.6	-170.5	-163.2
ΔE_{orb}	-143.8	-135.0	-131.1	-123.9
└ $\pi_{alkene}\rightarrow\sigma^*_{Pd-C\{Ar\}}$	-93.2 (64%)	-83,4 (62%)	-87.0 (66%)	-77.9 (63%)
└ $\sigma_{Pd-C\{Ar\}}\rightarrow\pi^*_{alkene}$	-30.1 (21%)	-28.4 (21%)	-25.3 (19%)	-25.1 (20%)
└ residual	-20.5	-23.2	-18.8	-20.9
**Σ(electronic)**	**-319.8**	**-302.6**	**-301.6**	**-287.1**
$\Delta E_{distort}$	64.6	59.0	59.0	54.6
ΔE_{Pauli}	299.9	289.9	284.3	276.9
**Σ(steric)**	**364.5**	**348.8**	**343.3**	**331.6**
ΔG_{thermo}	15.6	14.1	15.5	14.1
$\Delta\Delta G_{solv}$	2.0	3.3	2.3	3.7
**Σ(thermo)**	**17.6**	**17.4**	**17.9**	**17.8**
**ΔE$_{disp}$**	**-33.3**	**-35.3**	**-32.0**	**-32.9**
$\Delta E_{residual}$	-0.4	-0.9	-1.3	-2.4

10.4. ETS-NOCV analysis αTS$_{B.4\text{-}5}$ and βTS$_{B.4\text{-}5}$ for R = CN

Table S16. Selected ETS-NOCV analysis results on the 1st and 2nd contributions which account for 85% of the total orbital interaction in **αTS$_{B.4\text{-}5}$**. Orbital isosurfaces are plotted with a contour value of 0.03. Deformation density isosurfaces are plotted with a contour value of 0.004, charge flow goes from orange to cyan.

**αTS$_{B.4\text{-}5}$**	1st contribution	2nd contribution
Donor orbitals	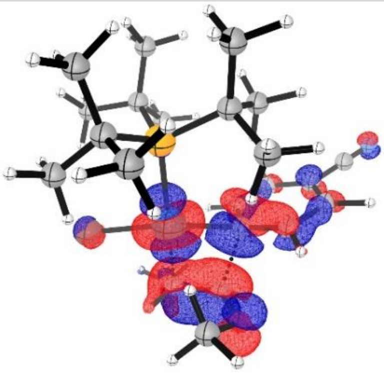	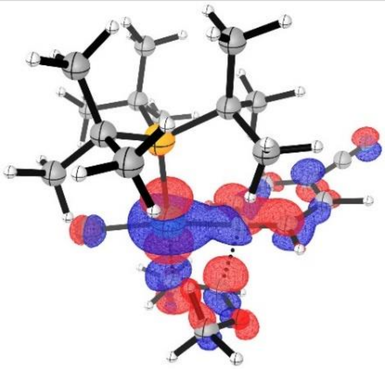
Acceptor orbitals	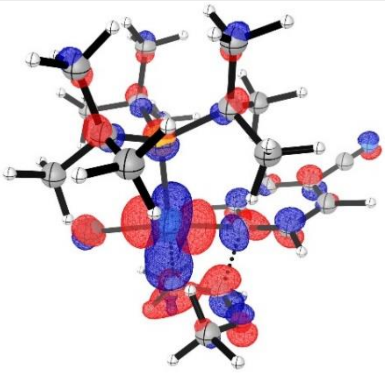	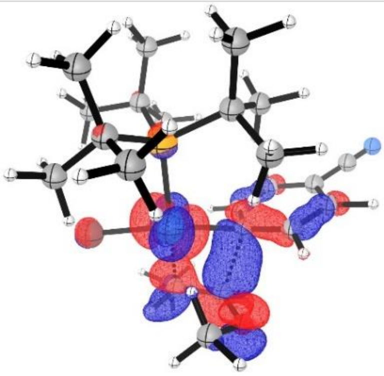
Deformation density	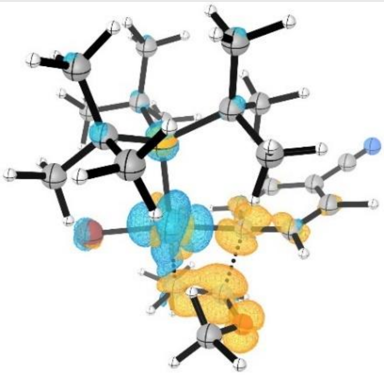	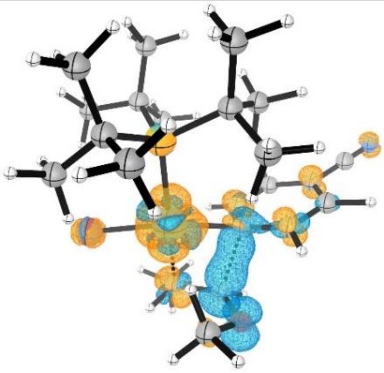
Stabilization energy (kcal/mol) / Eigenvalue (e)	-93.209 / 1.062	-30.132 / 0.662

Table S17. Selected ETS-NOCV analysis results on the 1st and 2nd contributions which account for 83% of the total orbital interaction in **βTS$_{B.4-5}$**. Orbital isosurfaces are plotted with a contour value of 0.03. Deformation density isosurfaces are plotted with a contour value of 0.004, charge flow goes from orange to cyan.

**βTS$_{B.4-5}$**	1st contribution	2nd contribution
Donor orbitals	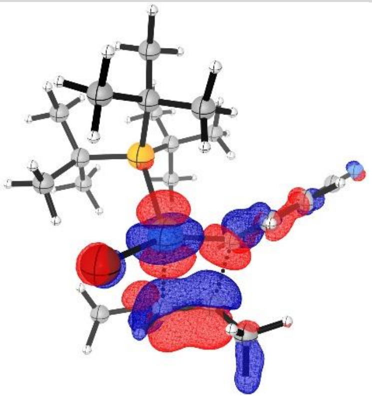	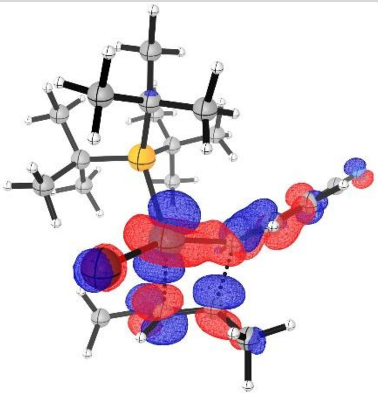
Acceptor orbitals	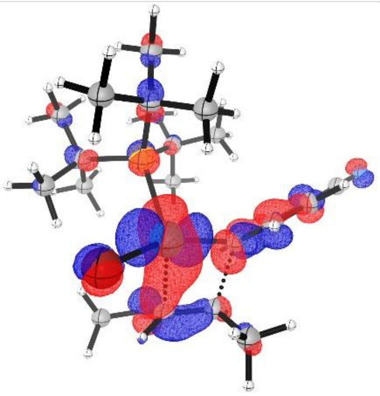	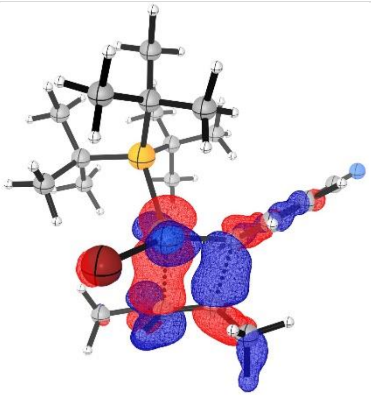
Deformation density	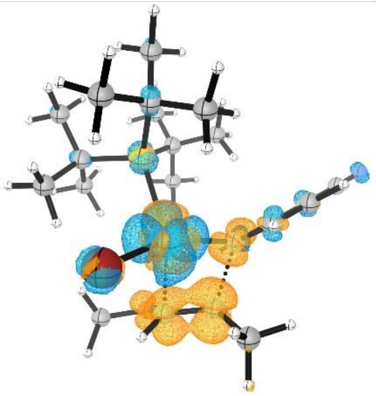	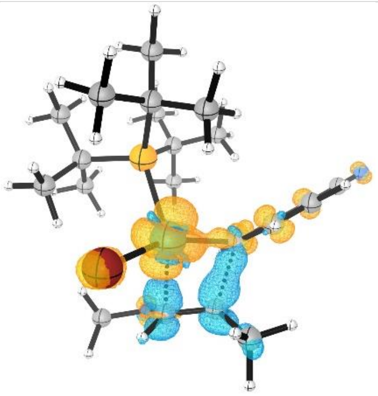
Stabilization energy (kcal/mol) / Eigenvalue (e)	-83.474 / 1.094	-28.401 / 0.611

10.5 HOMO and LUMO for αTS$_{B.4\text{-}5}$ and βTS$_{B.4\text{-}5}$

Table S18. HOMO and LUMO orbitals for **αTS$_{B.4\text{-}5}$** and **βTS$_{B.4\text{-}5}$**, R = CN. Orbital isosurfaces are plotted with a contour value of 0.03.

	HOMO	LUMO
**αTS$_{B.4\text{-}5}$**<br>**R = CN**<br>**{Pd-Ar}**	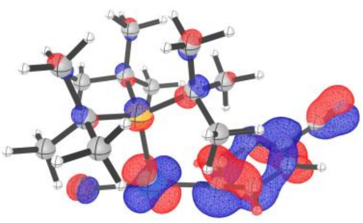<br>–9.0793 eV	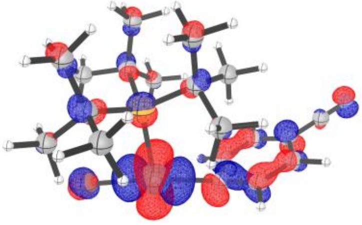<br>–0.1604 eV
**αTS$_{B.4\text{-}5}$**<br>**R = CN**<br>**{alkene}**	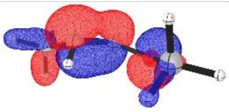<br>–8.3690 eV	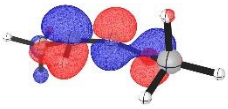<br>+3.2470 eV
**βTS$_{B.4\text{-}5}$**<br>**R = CN**<br>**{Pd-Ar}**	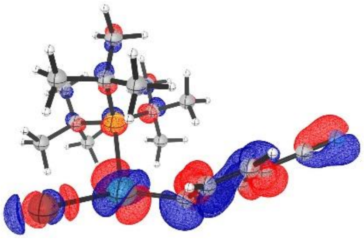<br>–9.0540 eV	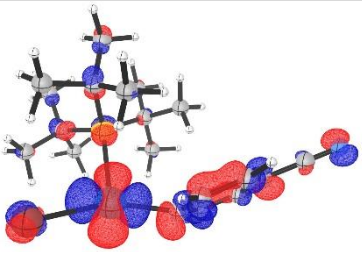<br>–0.1689 eV
**βTS$_{B.4\text{-}5}$**<br>**R = CN**<br>**{alkene}**	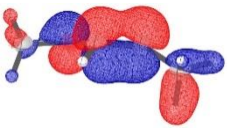<br>–8.2528 eV	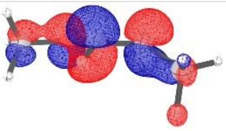<br>+3.2514 eV

Table S19. HOMO and LUMO orbitals for **αTS$_{B.4\text{-}5}$** and **βTS$_{B.4\text{-}5}$**, R = OMe. Orbital isosurfaces are plotted with a contour value of 0.03.

	HOMO	LUMO
**αTS$_{B.4\text{-}5}$**<br>**R = OMe**<br>**{Pd-Ar}**	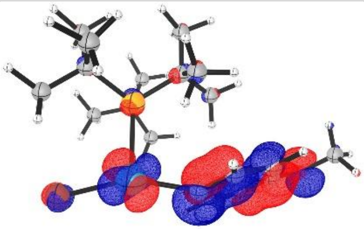<br>–8.1350 eV	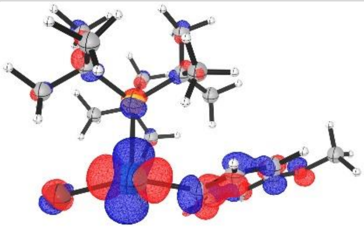<br>+0.3774 eV
**αTS$_{B.4\text{-}5}$**<br>**R = OMe**<br>**{alkene}**	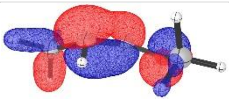<br>–8.3852 eV	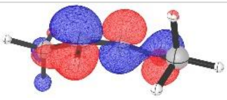<br>+3.3310 eV
**βTS$_{B.4\text{-}5}$**<br>**R = OMe**<br>**{Pd-Ar}**	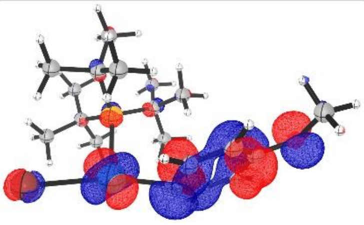<br>–8.2246 eV	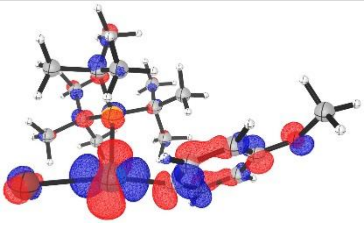<br>+0.3637 eV
**βTS$_{B.4\text{-}5}$**<br>**R = OMe**<br>**{alkene}**	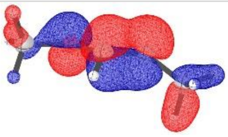<br>–8.3282 eV	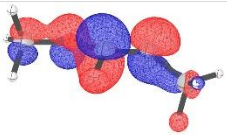<br>+3.3129 eV

11. Stereoselectivity: ligand control and post-catalytic stereo-correction

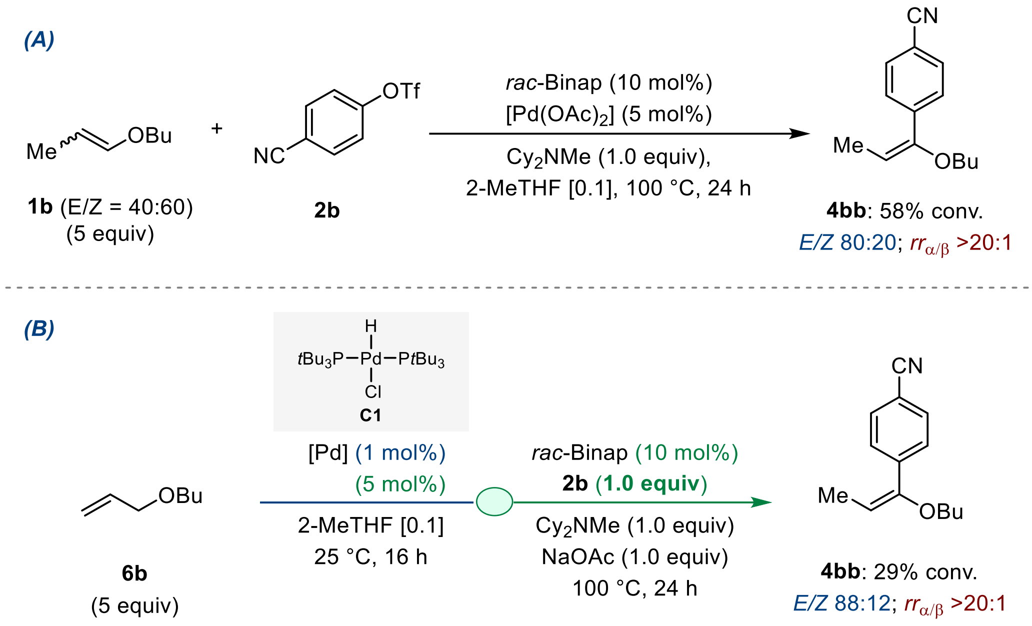

Figure S19. (A) Ligand-controlled stereoselective Heck α-arylation of enol ether **1b**. (B) Ligand-controlled stereoselective [Isomerization/Heck α-arylation] assisted tandem (AT) process.

(A): Adapted from GP IV using [Pd(OAc)$_2$] (1.1 mg, 0.005 mmol, 5 mol% to **2b**), *rac*-Binap (6.2 mg, 0.01 mmol, 10 mol% to **2b**), vinyl ether **1b** (57.1 mg, 0.5 mmol, 5 equiv), Cy$_2$NMe (19.5 mg, 0.1 mmol, 1 equiv) and aryl triflate **2b** (25.1 mg, 0.1 mmol, 1 equiv). The reaction afforded **4bb** in 58% conversion (*E/Z* 80:20, $rr_{\alpha/\beta}$ >20:1) assessed by ^{1}H NMR analysis of the crude reaction mixture using an internal standard.

(B): Adapted from GP X using **C1** (2.7 mg, 0.005 mmol, 1 mol% to **6b** - 5 mol% to **2b**), allyl butyl ether **6b** (74.1 mg, 0.5 mmol, 5 equiv), *rac*-Binap (6.2 mg, 0.01 mmol, 10 mol% to **2b**), NaOAc (8.2 mg, 0.1 mmol, 1 equiv), Cy$_2$NMe (19.5 mg, 0.1 mmol, 1 equiv) and aryl triflate **2b** (25.1 mg, 0.1 mmol, 1 equiv). The reaction afforded **4bb** in 29% conversion (*E/Z* 88:12, $rr_{\alpha/\beta}$ >20:1) assessed by ^{1}H NMR analysis of the crude reaction mixture using an internal standard.

Into a microwave tube, the tri-substituted vinyl ether **4bb** (64.6 mg, 0.3 mmol, 1 equiv) or **5ab** (74.8 mg, 0.3 mmol, 1 equiv), pyridinium *p*-toluenesulfonate (113 mg, 0.45 mmol, 1.5 equiv), TMSCl (65.2 mg, 0.6 mmol, 2 equiv) were dissolved in DMA (1 mL, 0.3M). The tube was sealed and irradiated under microwave at 160 °C for 20 min. Once the system had reached 25 °C, it was quenched with water (5 mL) and diluted with Et_2O (20 mL). The organic phase was washed with a saturated solution of $NaHCO_3$ (4 × 100 mL) and brine (100 mL). The organic layer was dried over anhydrous Na_2SO_4, filtered and concentrated under vacuum. The residue was purified by column chromatography (neutral alumina) using pentane/Et_2O 98:2 as eluent with 1% Et_3N to afford product **4bb** in pure form as a pale yellow oil (58 mg, 0.27 mmol, 90% yield, *E/Z* 8:92) or by silica gel chromatography using pentane/Et_2O 90:10 as eluent to afford product **5ab** in pure form as a white solid (59.4 mg, 0.276 mmol, 92% yield, *E/Z* 81:19).

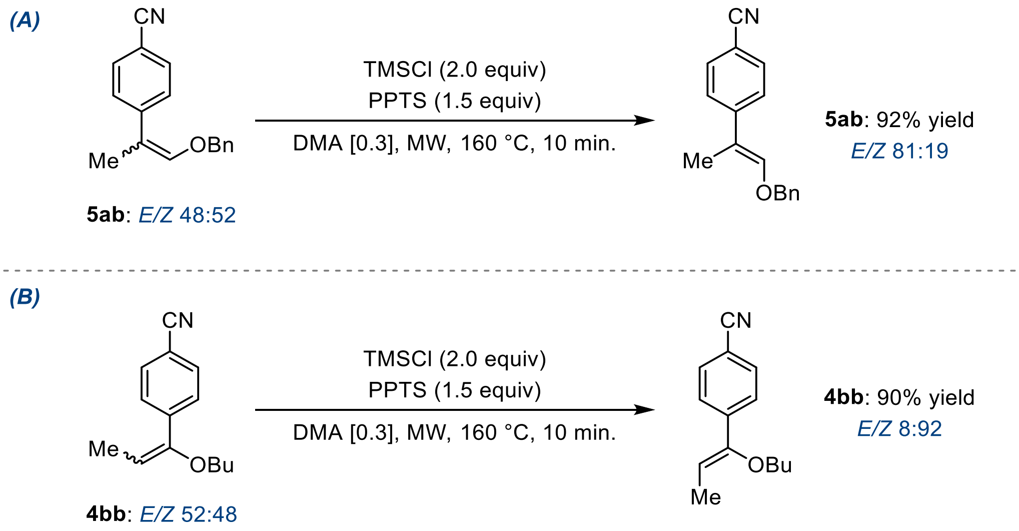

Figure S20. Post-catalytic stereo-correction using Lewis-assisted protic activation protocol.

12. References

(1) Procedure adapted from: Myhill, J. A.; Zhang, L.; Lovinger, G. J.; Morken, J. P. *Angew. Chem. Int. Ed.* **2018**, *57*, 12799–10803.

(2) Procedure adapted from: Vyvyan, J. R.; Dell, J. A.; Ligon, T. J.; Motanic, K. K.; Wall, H. S. *Synthesis* **2010**, *21*, 3637–3644.

(3) To prepare 100 g of silica containing 10 wt% of $AgNO_3$: In a round bottom flask covered by an aluminum foil was charged 10 g of $AgNO_3$ and 20 mL of water. Once all $AgNO_3$ was dissolved, 100 mL of MeOH was added followed by 90 g of silica. The gel was stirred for ca. 5 min. The solvents were removed under vacuum and the silica containing 10 wt% of $AgNO_3$ was dried overnight in an oven at 120 °C.

<u>Caution:</u> To avoid the formation of silver(0), all the procedure must be carried out with the lights off and all the glassware must be covered with an aluminum foil. During chromatography, the walls of the column are covered with an aluminum foil.

(4) Schimler, S. D.; Froese, R. D. J.; Bland, D. C.; Sanford. M. S. *J. Org. Chem.* **2018**, *83*, 11178–11190.

(5) Taeufer, T.; Pospech, J. *J. Org. Chem.* **2020**, *85*, 7097–7111.

(6) Qin, L.; Ren, X.; Lu, Y.; Li, Y.; Zhou, J. *Angew. Chem. Int. Ed.* **2012**, *51*, 5915–5919.

(7) Dürr, A. B.; Yin, G.; Kalvet, I.; Napoly, F.; Schoenebeck, D. *Chem. Sci.* **2016**, *7*, 1076–1081.

(8) Yu, P.; Morandi, B. *Angew. Chem. Int. Ed.* **2017**, *56*, 15693–15697.

(9) Zhu, Z.; Weiqi, Y.; Xue, W.; Gong, H. *Org. Lett.* **2021**, *23*, 2158−2163.

(10) Dogga, B.; Kumar, A.; Joseph, J. T. *Eur. J. Org. Chem.* **2021**, 309–313.

(11) Planas, O.; Peciukenas, V.; Cornella, J. *J. Am. Chem. Soc.* **2020**, *142*, 11382–11387.

(12) Vinogradova, E. V.; Park, N. H.; Fors, B. P.; Buchwald. S. L. *Org. Lett.* **2013**, *15*, 1394–1397.

(13) Procedure adapted from: Lu, C.; Su, X.; Floreancig, P. E. *J. Org. Chem.* **2013**, *78*, 9366–9376.

(14) Kathe, P.; Berkefeld, A.; Fleischer, I. *Synlett* **2021**, *32*, 1629–1632.

(15) Ekholm, F. S.; Pynnönen, H.; Vilkman, A.; Pitkänen, V.; Helin, J.; Saarinene, J.; Satomaa, T. *ChemMedChem* **2016**, *11*, 2501–2505.

(16) Ma, X.; Herzon, S. B. *Chem. Sci.* **2015**, *6*, 6250–6255.

(17) Connolly, T.; Wang, Z.; Walker, M. A.; McDonald, I. M.; Peese, K. M. *Org. Lett.* **2014**, *16*, 4444–4447.

(18) Florence, G. J.; Fraser, A. L.; Gould, E. R.; King, E. F. B.; Menzies, S. K.; Morris, J. C.; Tulloch, L. B.; Smith, T. K. *ChemMedChem* **2014**, *9*, 2548–2556.

(19) Hu, Y.; Shen, Z.; Huan, H. *ACS Catal.* **2016**, *6*, 6785–6789.

(20) Arendse, M. J.; Anderson, G. K.; Rath, N. P. *Polyhedron* **2001**, *20*, 2495–2503.
(21) Anderson, C.-M.; Hallberg, A. *J. Org. Chem.* **1987**, *52*, 3529–3536.
(22) Pracht, P.; Bohle, F.; Grimme, S. *Phys. Chem. Chem. Phys.* **2020**, *22*, 7169–7192.
(23) Bannwarth, C.; Ehlert, S.; Grimme, S. *J. Chem. Theory Comput.* **2019**, *15*, 1652–1671.
(24) Neese, F. Software Update: The ORCA Program System—Version 5.0. *WIREs Comput. Mol. Sci.* **2022**, *12*, e1606.
(25) Chai, J.-D.; Head-Gordon, M. *J. Chem. Phys.* **2008**, *128*, 084106.
(26) Grimme, S.; Antony, J.; Ehrlich, S.; Krieg, H. *J. Chem. Phys.* **2010**, *132*, 154104.
(27) Grimme, S.; Hansen, A.; Ehlert, S.; Mewes, J.-M. *J. Chem. Phys.* **2021**, *154*, 064103.
(28) Weigend, F. *Phys. Chem. Chem. Phys.* **2006**, *8*, 1057–1065.
(29) Barone, V.; Cossi, M. *J. Phys. Chem. A* **1998**, *102*, 1995–2001.
(30) Riplinger, C.; Pinski, P.; Becker, U.; Valeev, E. F.; Neese, F. *J. Chem. Phys.* **2016**, *144*, 024109.
(31) Weigend, F.; Ahlrichs, R. *Phys. Chem. Chem. Phys.* **2005**, *7*, 3297–3305.
(32) Hellweg, A.; Hättig, C.; Höfener, S.; Klopper, W. *Theor. Chem. Acc.* **2007**, *117*, 587–597.
(33) Glendening, E. D.; Landis, C. R.; Weinhold, F. *J. Comput. Chem.* **2013**, *34*, 1429–1437.
(34) Lu, T.; Chen, F. *J. Comput. Chem.* **2012**, *33*, 580–592.
(35) Schneider, W. B.; Bistoni, G.; Sparta, M.; Saitow, M.; Riplinger, C.; Auer, A. A.; Neese, F. *J. Chem. Theory Comput.* **2016**, *12*, 4778–4792.
(36) Altun, A.; Neese, F.; Bistoni, G. *Beilstein J. Org. Chem.* **2018**, *14*, 919–929.
(37) Mitoraj, M. P.; Michalak, A.; Ziegler, T. *J. Chem. Theory Comput.* **2009**, *5*, 962–975.
(38) Altun, A.; Neese, F.; Bistoni, G. *J. Chem. Theory Comput.* **2019**, *15*, 215–228.

13. NMR spectra

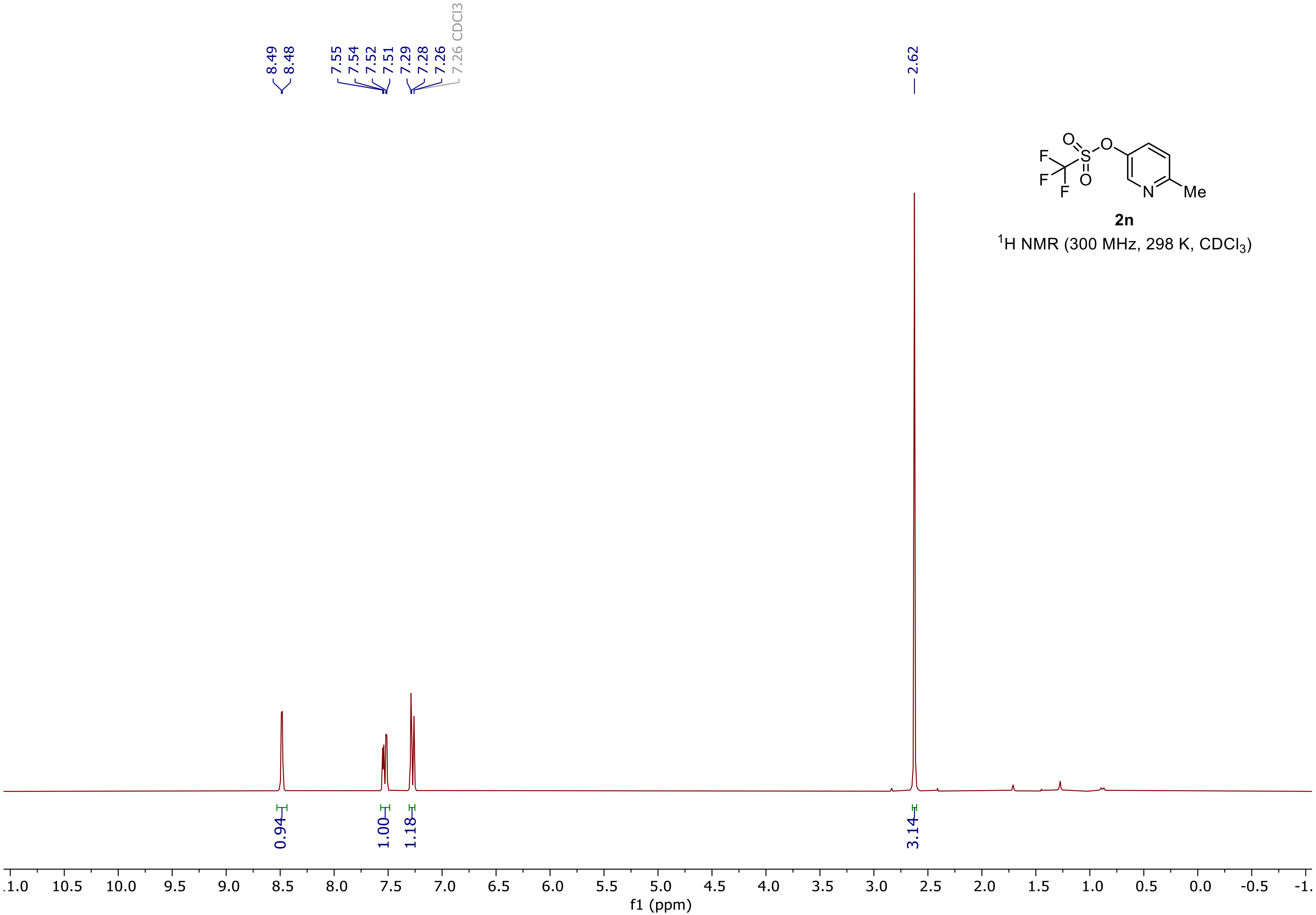

2n

^1H NMR (300 MHz, 298 K, $CDCl_3$)

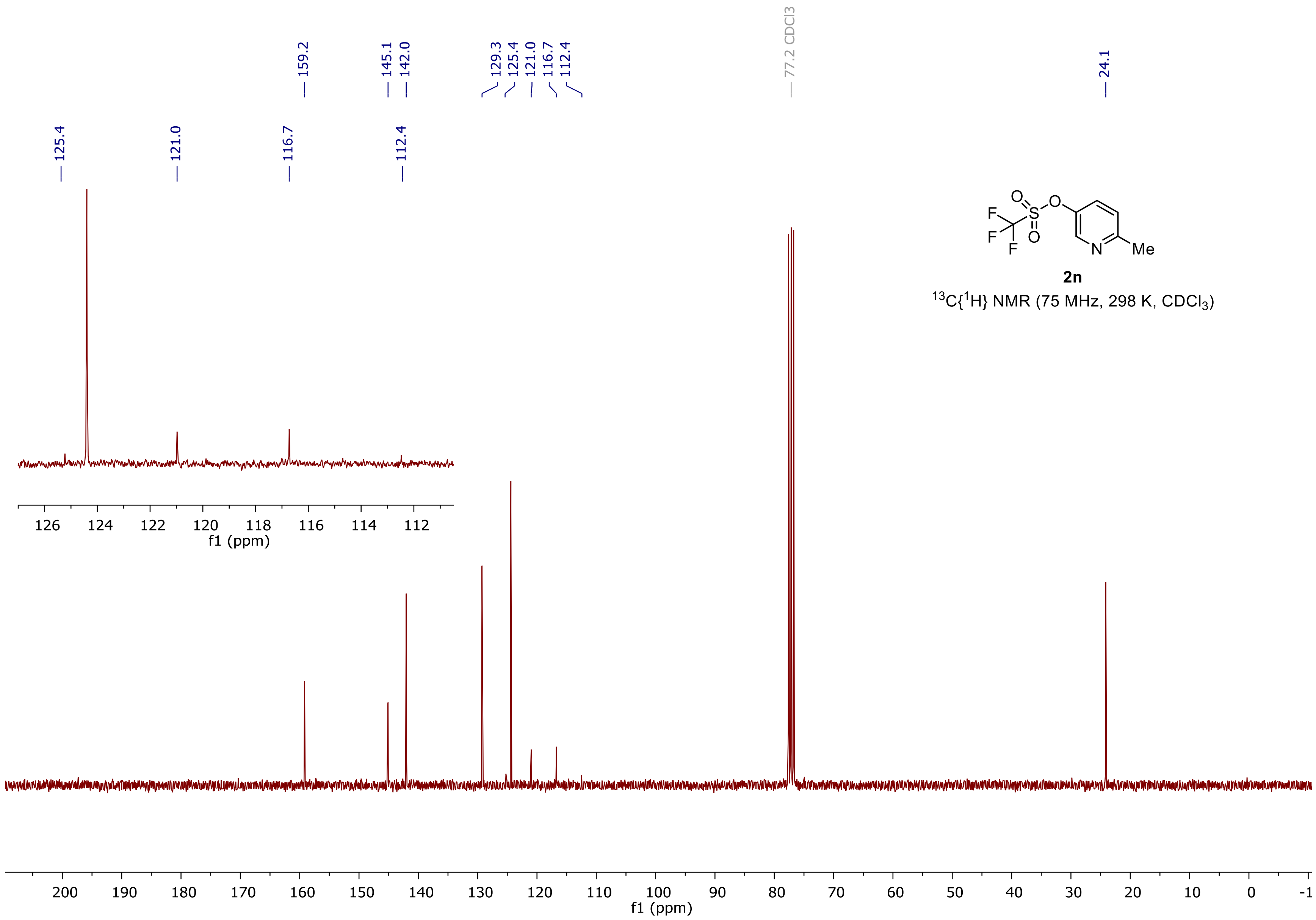

2n

$^{13}C\{^{1}H\}$ NMR (75 MHz, 298 K, $CDCl_3$)

2n
$^{19}F\{^1H\}$ NMR (282 MHz, 298 K, $CDCl_3$)

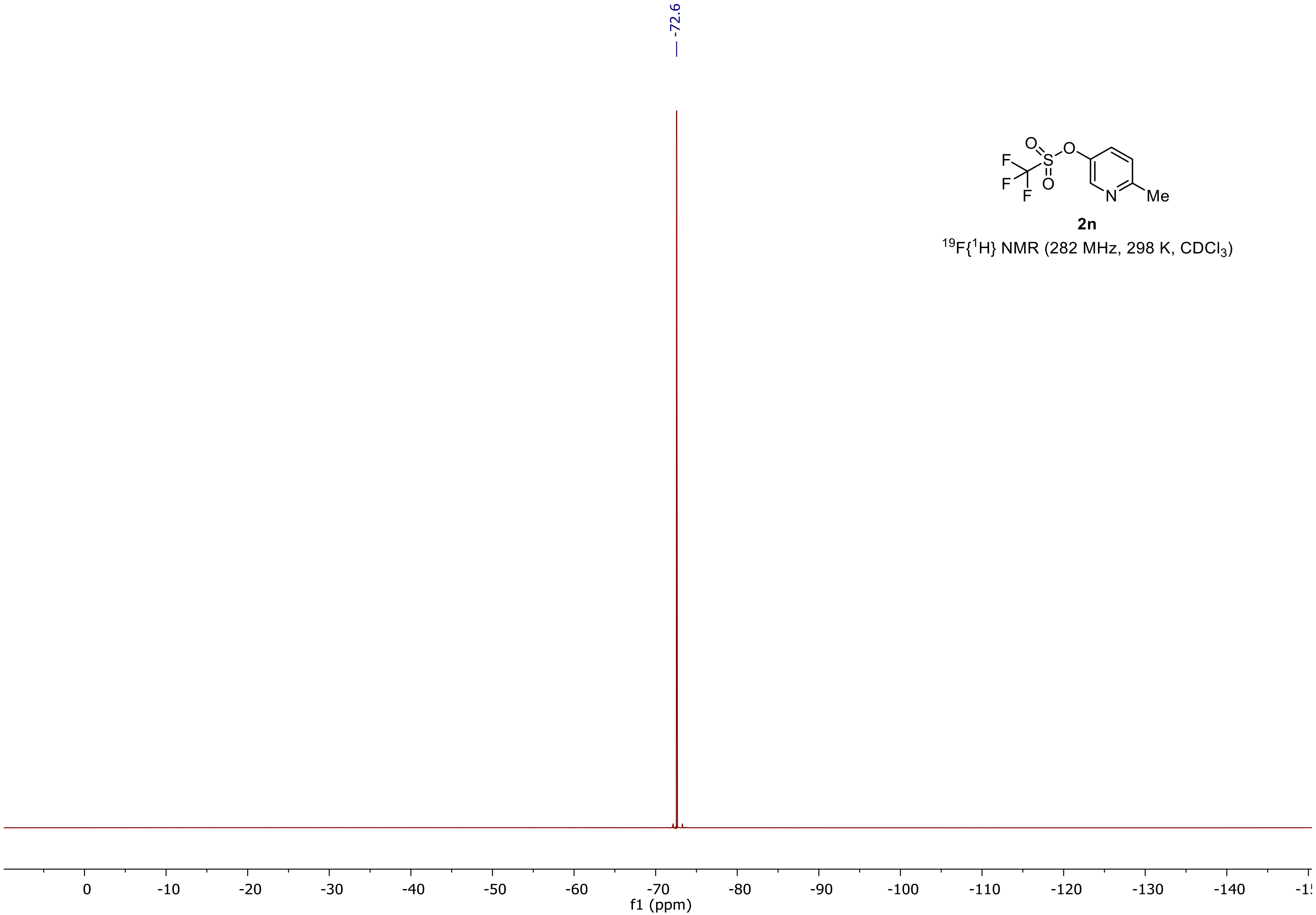

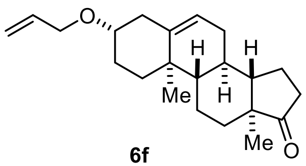

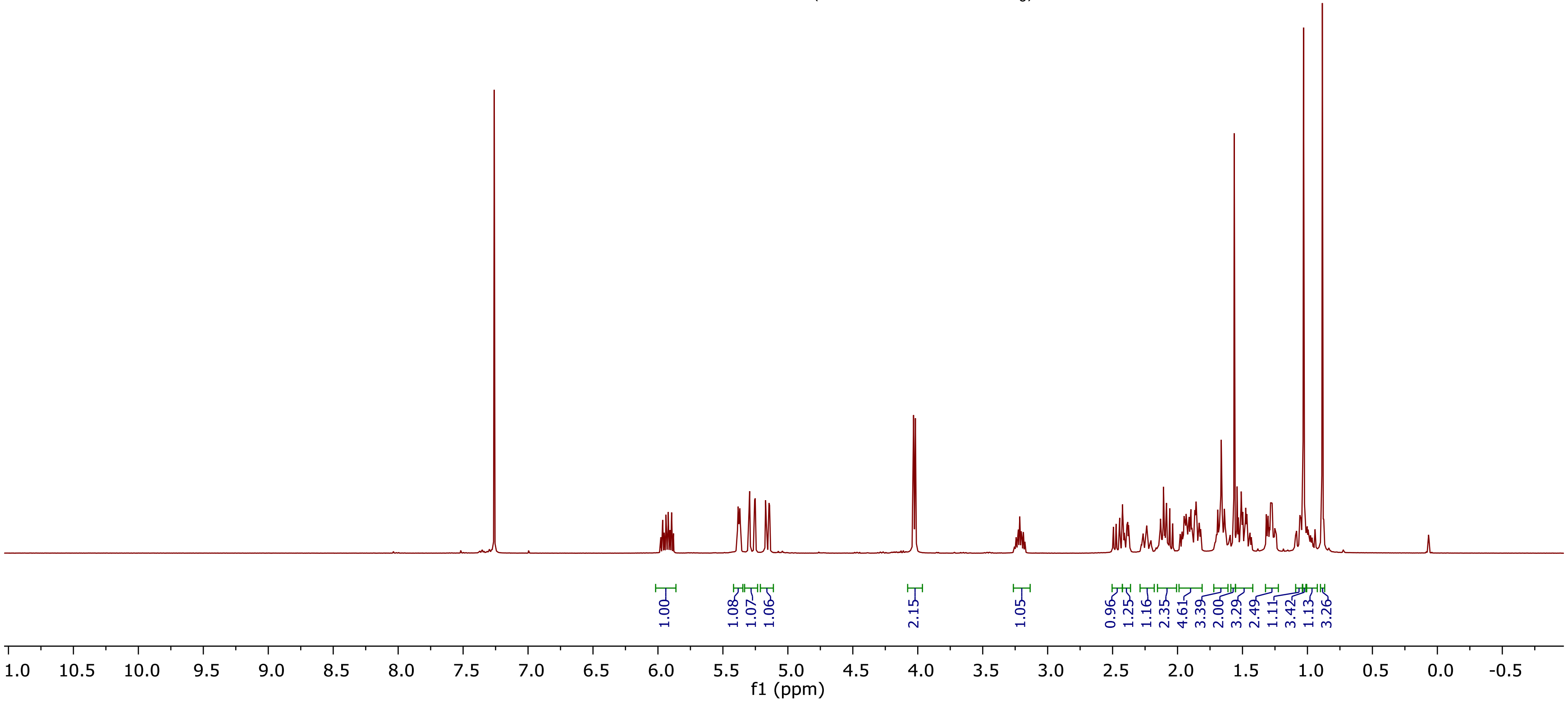

6f

^{1}H NMR (400 MHz, 298 K, $CDCl_3$)

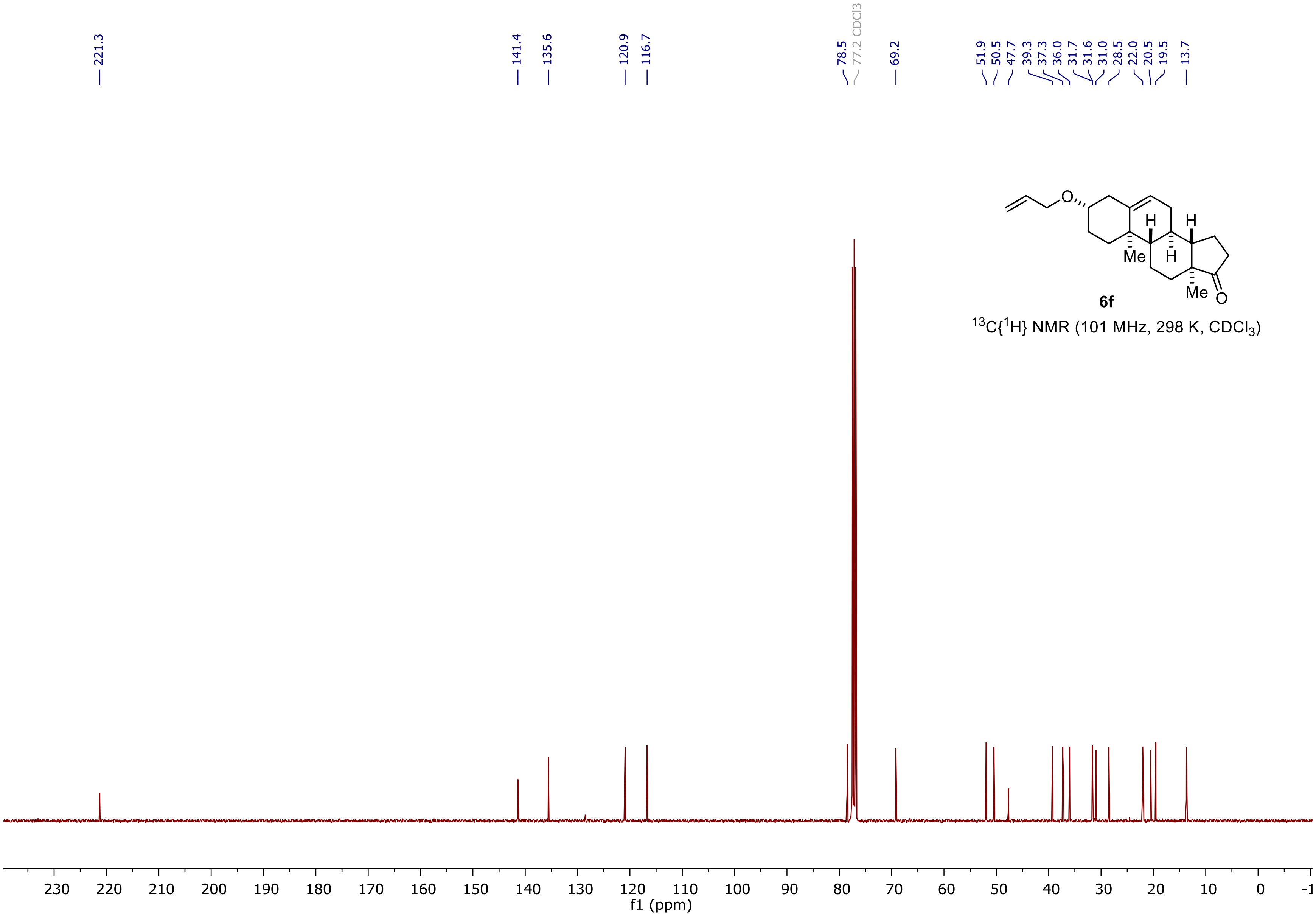
221.3
141.4
135.6
120.9
116.7
78.5
77.2 CDCl3
69.2
51.9
50.5
47.7
39.3
37.3
36.0
31.7
31.6
31.0
28.5
22.0
20.5
19.5
13.7
O
H
H
Me
H
Me
O
6f
$^{13}C\{^{1}H\}$ NMR (101 MHz, 298 K, $CDCl_3$)
230
220
210
200
190
180
170
160
150
140
130
120
110
100
90
80
70
60
50
40
30
20
10
0
-1
f1 (ppm)

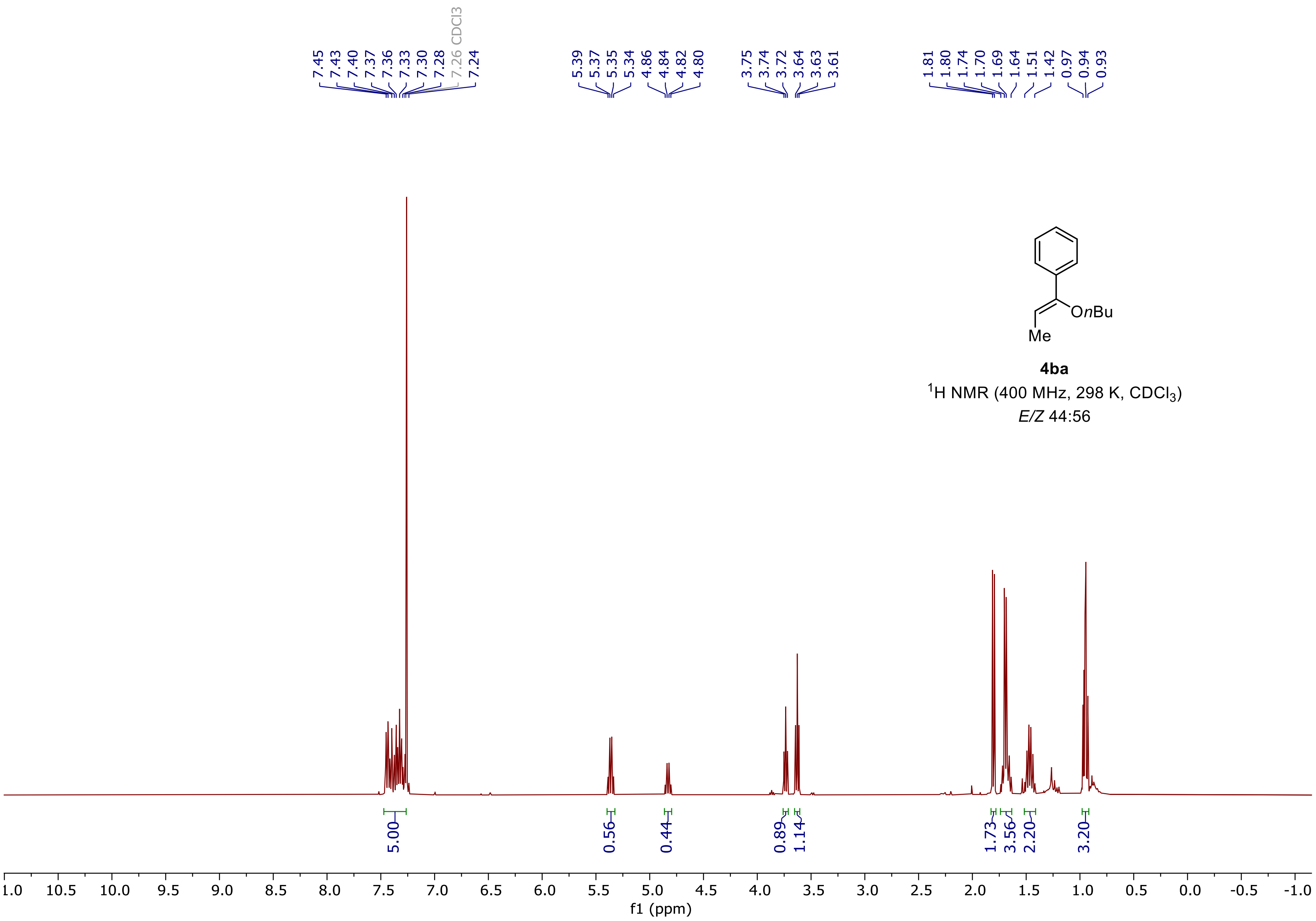

4ba

^{1}H NMR (400 MHz, 298 K, $CDCl_3$)

E/Z 44:56

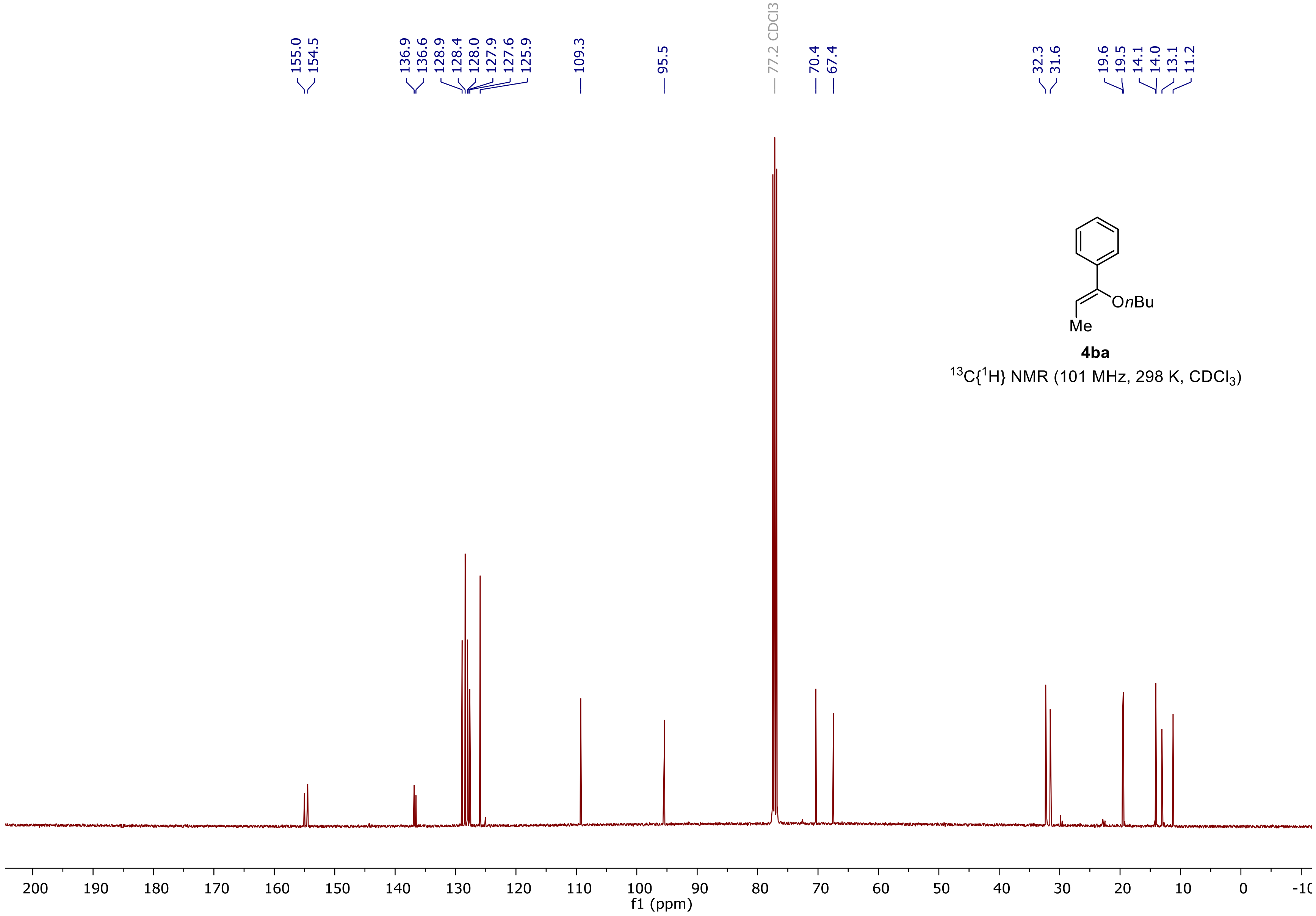

4ba

$^{13}C\{^{1}H\}$ NMR (101 MHz, 298 K, $CDCl_3$)

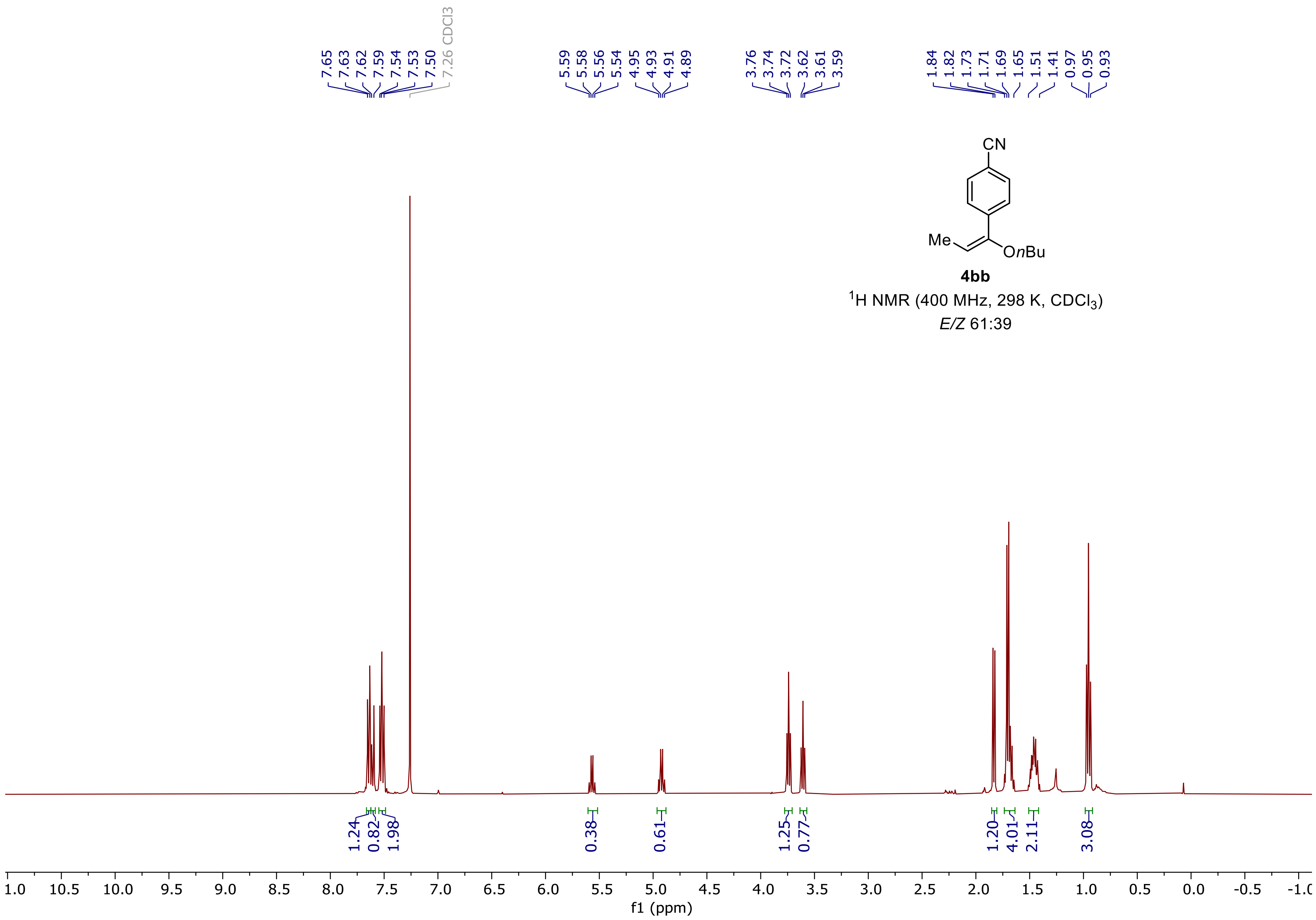

4bb

^{1}H NMR (400 MHz, 298 K, $CDCl_3$)

E/Z 61:39

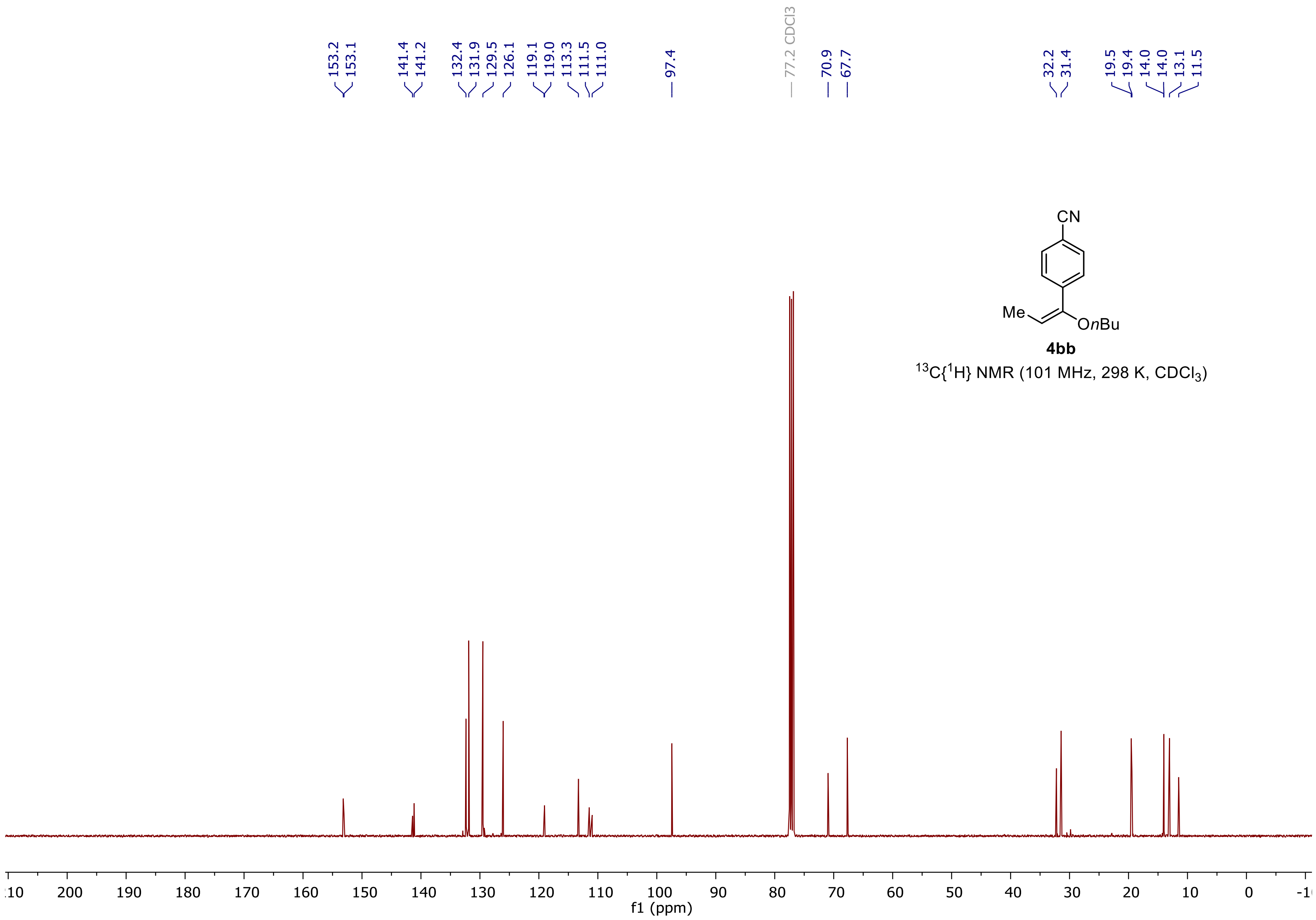

4bb

$^{13}C\{^{1}H\}$ NMR (101 MHz, 298 K, $CDCl_3$)

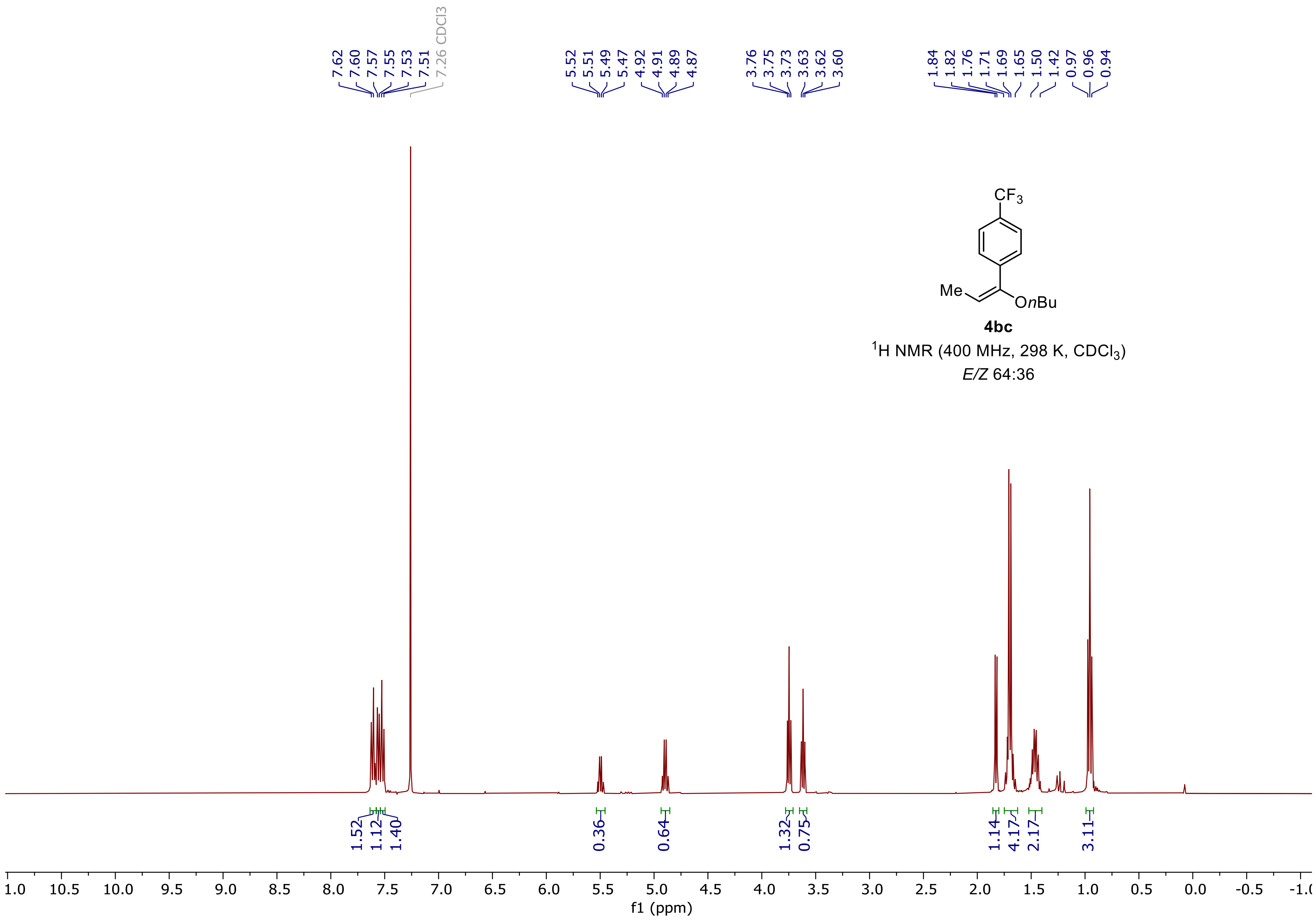

4bc

^{1}H NMR (400 MHz, 298 K, $CDCl_3$)

E/Z 64:36

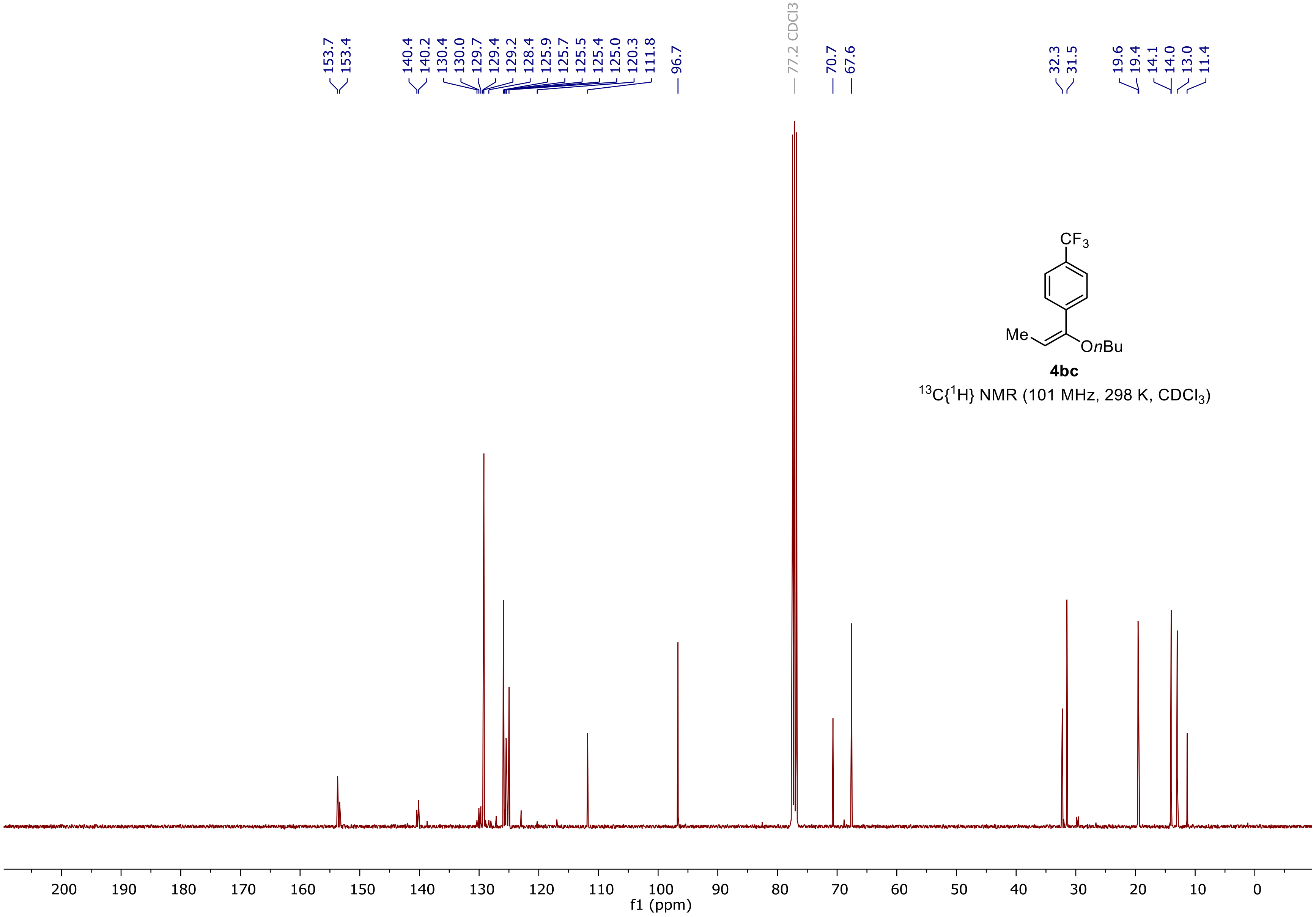
153.7
153.4
140.4
140.2
130.4
130.0
129.7
129.4
129.2
128.4
125.9
125.7
125.5
125.4
125.0
120.3
111.8
96.7
77.2 CDCl3
70.7
67.6
32.3
31.5
19.6
19.4
14.1
14.0
13.0
11.4
CF3
Me
OnBu
4bc
$^{13}C\{^1H\}$ NMR (101 MHz, 298 K, $CDCl_3$)
200 190 180 170 160 150 140 130 120 110 100 90 80 70 60 50 40 30 20 10 0
f1 (ppm)

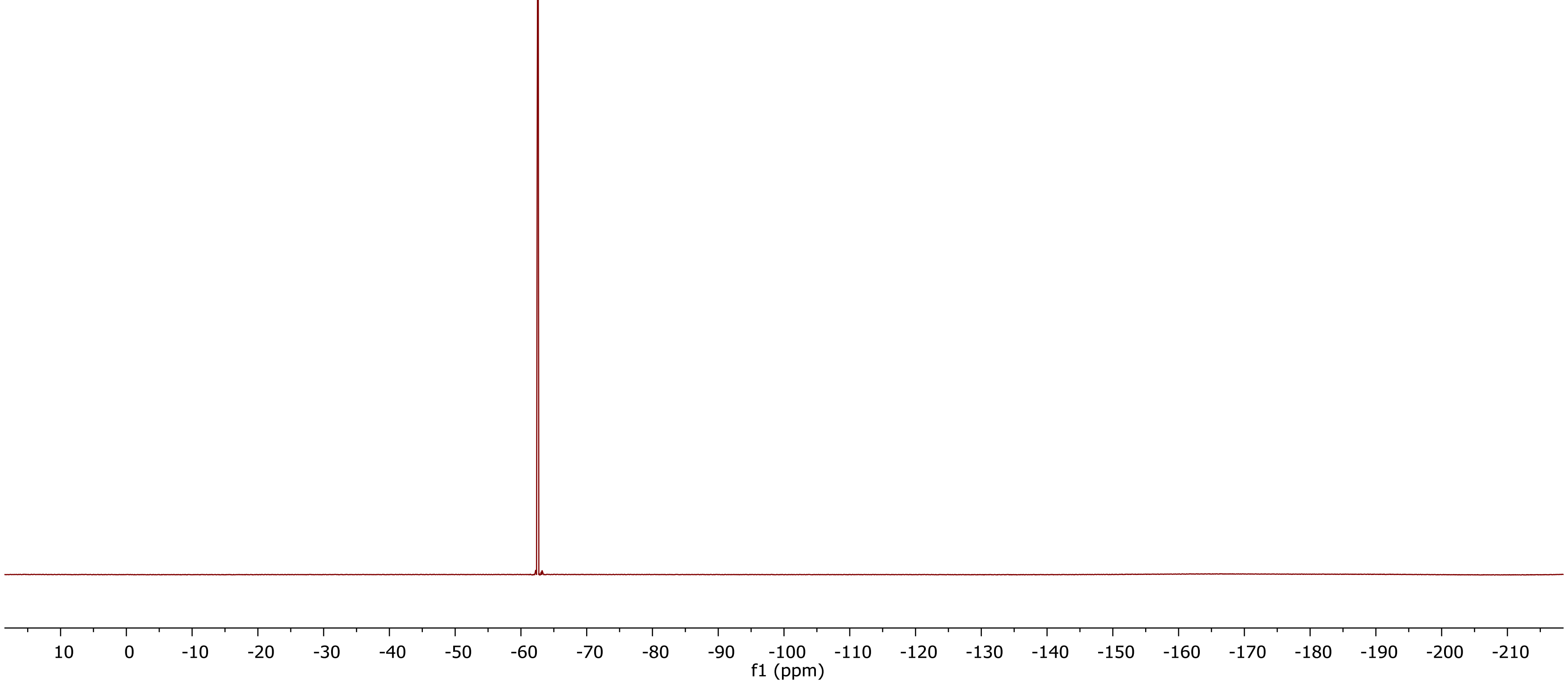

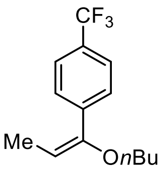

4bc

$^{19}F\{^{1}H\}$ NMR (282 MHz, 298 K, $CDCl_3$)

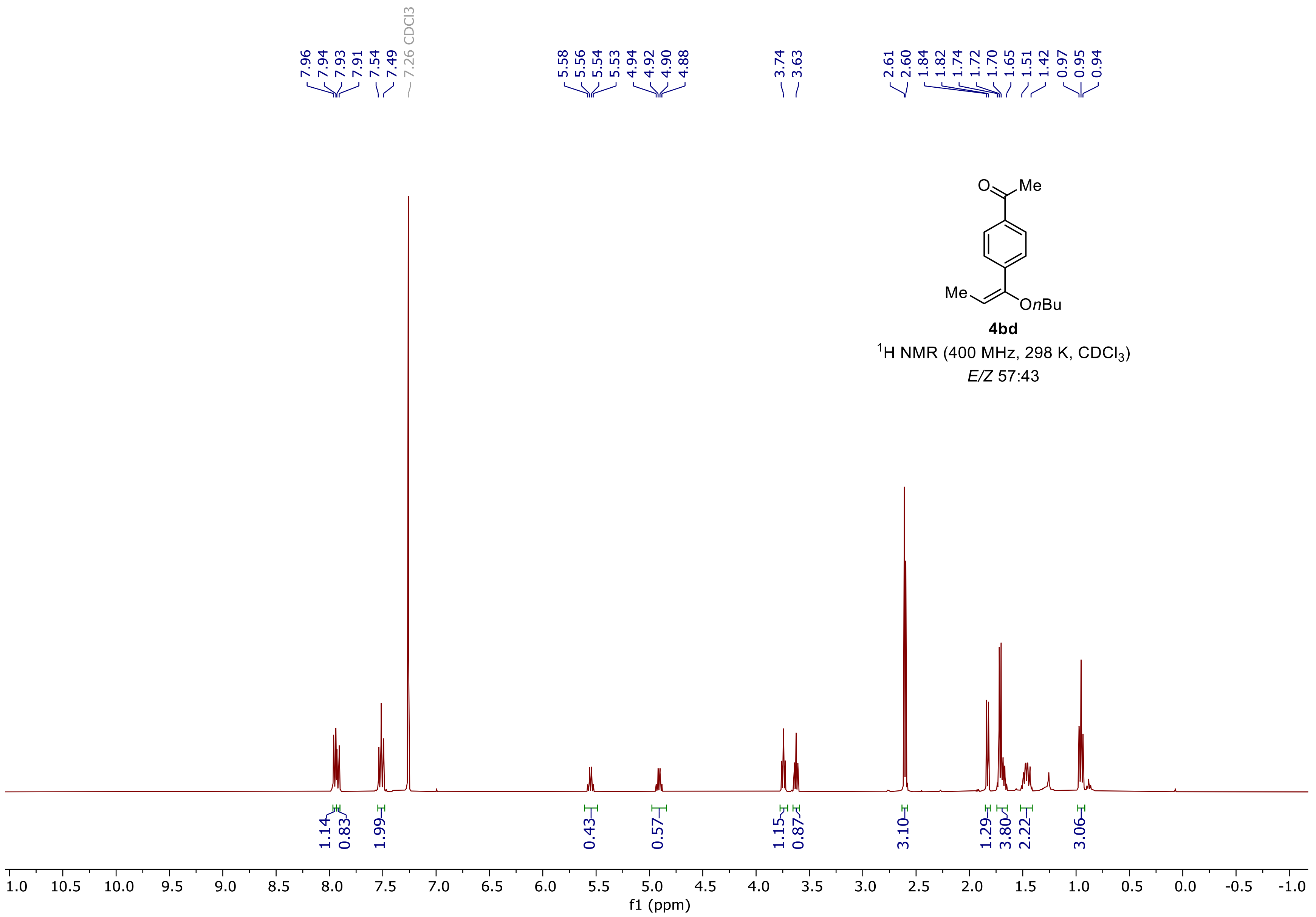
7.96
7.94
7.93
7.91
7.54
7.49
7.26 CDCl3
5.58
5.56
5.54
5.53
4.94
4.92
4.90
4.88
3.74
3.63
2.61
2.60
1.84
1.82
1.74
1.72
1.70
1.65
1.51
1.42
0.97
0.95
0.94
O
Me
Me
OnBu
4bd
1H NMR (400 MHz, 298 K, CDCl3)
E/Z 57:43
1.14
0.83
1.99
0.43
0.57
1.15
0.87
3.10
1.29
3.80
2.22
3.06
1.0
10.5
10.0
9.5
9.0
8.5
8.0
7.5
7.0
6.5
6.0
5.5
5.0
4.5
4.0
3.5
3.0
2.5
2.0
1.5
1.0
0.5
0.0
-0.5
-1.0
f1 (ppm)

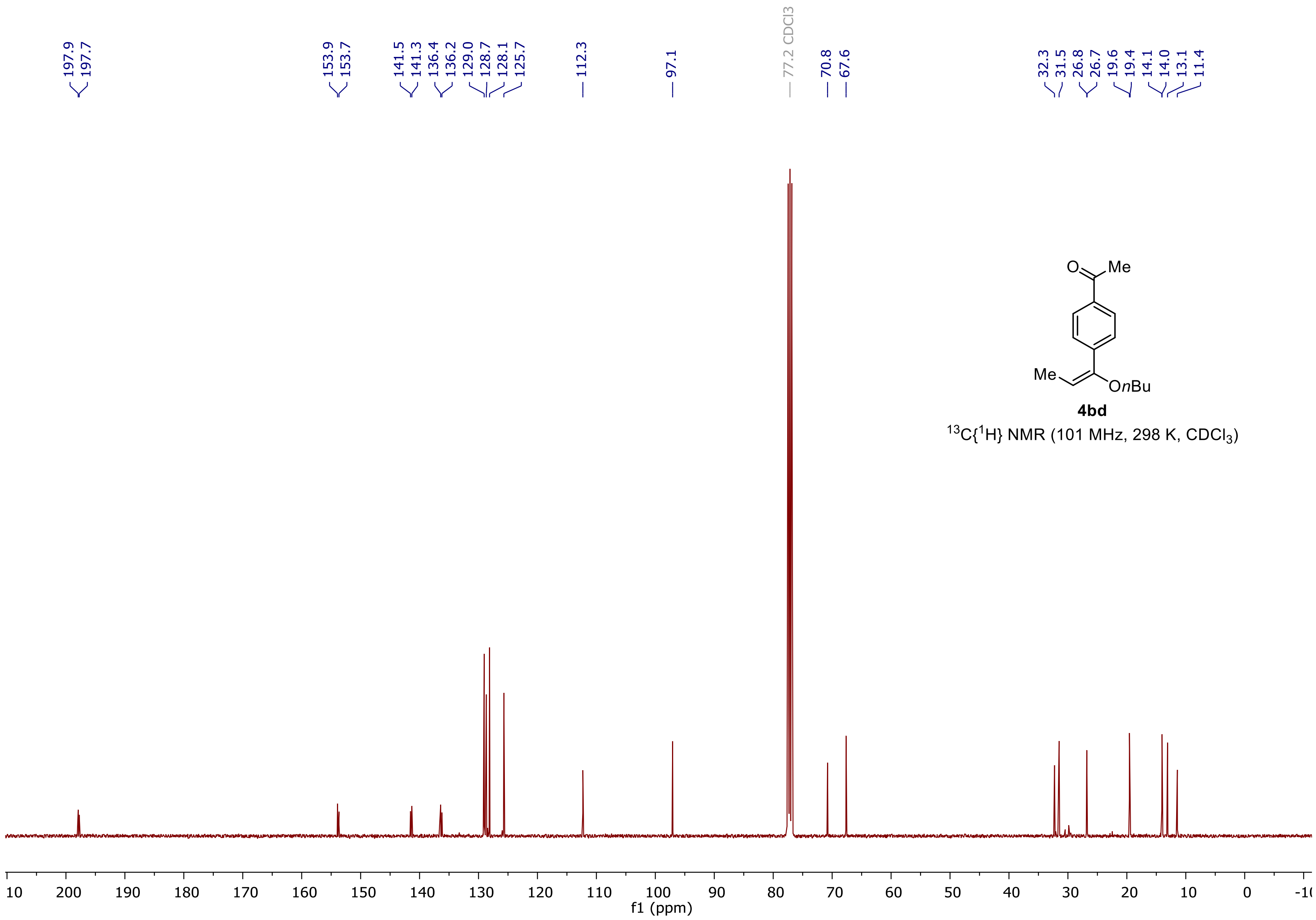

4bd

$^{13}C\{^1H\}$ NMR (101 MHz, 298 K, $CDCl_3$)

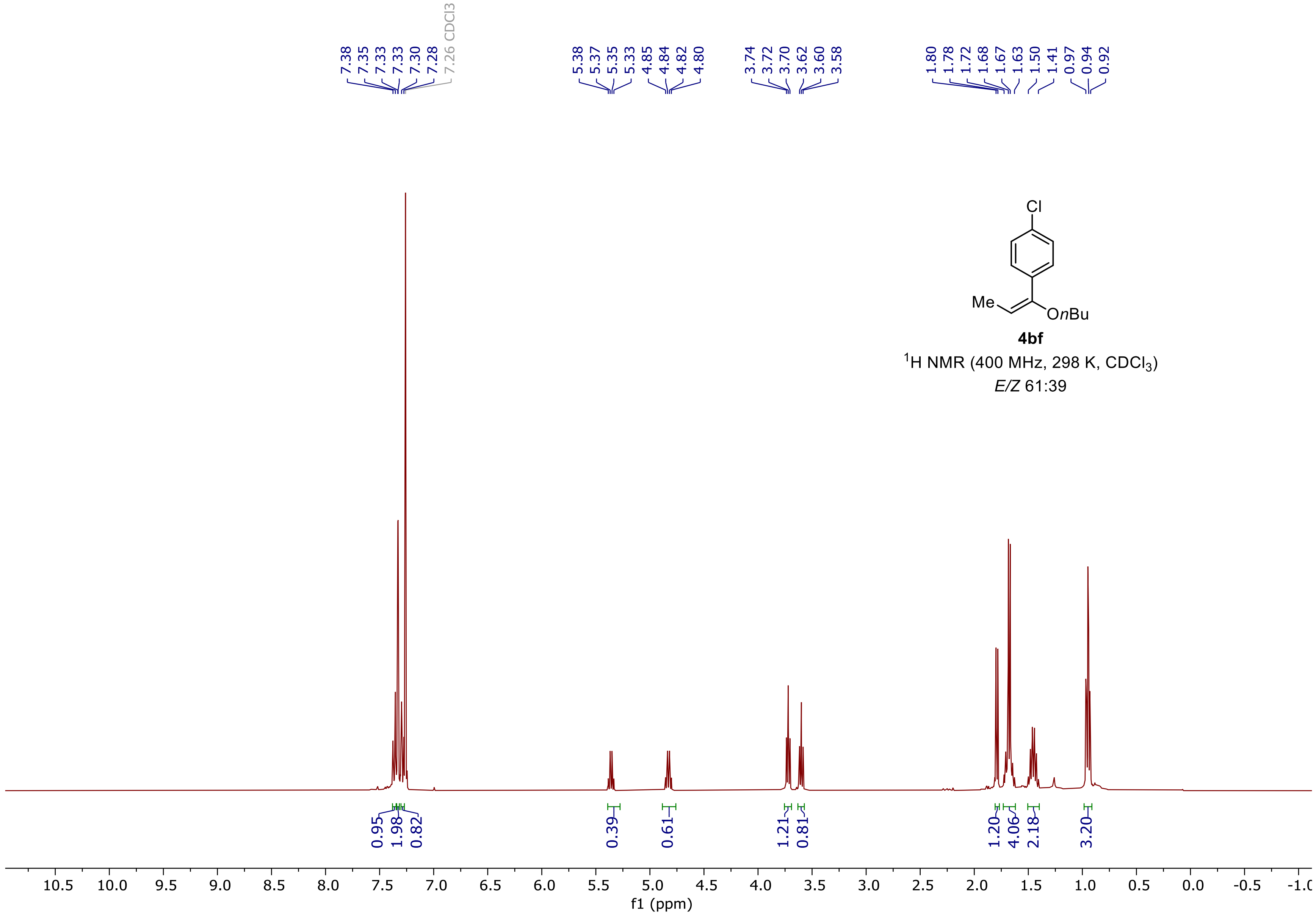
7.38
7.35
7.33
7.33
7.30
7.28
7.26 CDCl3
5.38
5.37
5.35
5.33
4.85
4.84
4.82
4.80
3.74
3.72
3.70
3.62
3.60
3.58
1.80
1.78
1.72
1.68
1.67
1.63
1.50
1.41
0.97
0.94
0.92
Cl
Me
O*n*Bu
4bf
^{1}H NMR (400 MHz, 298 K, $CDCl_3$)
E/Z 61:39
0.95
1.98
0.82
0.39
0.61
1.21
0.81
1.20
4.06
2.18
3.20
f1 (ppm)

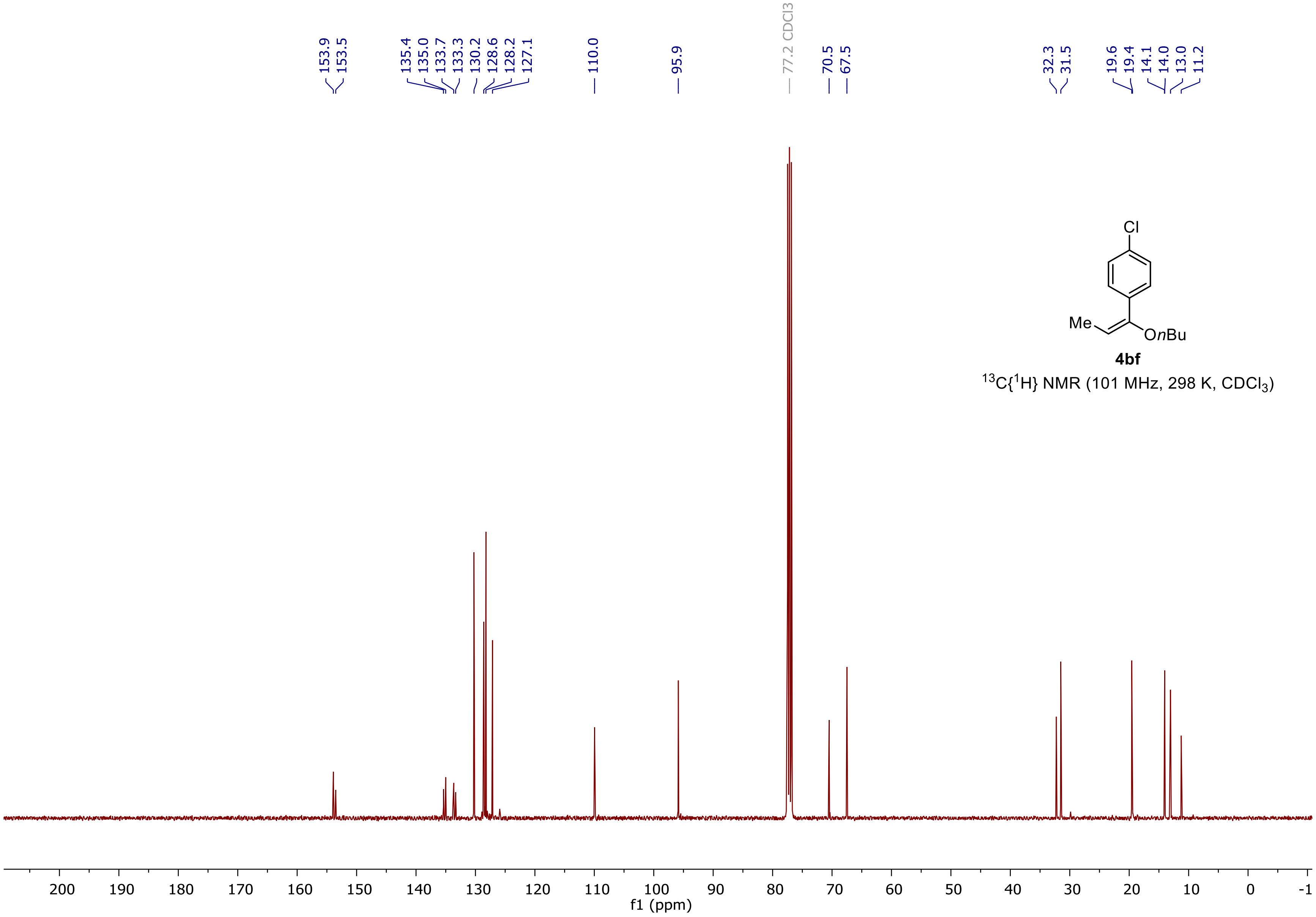

4bf

$^{13}C\{^1H\}$ NMR (101 MHz, 298 K, $CDCl_3$)

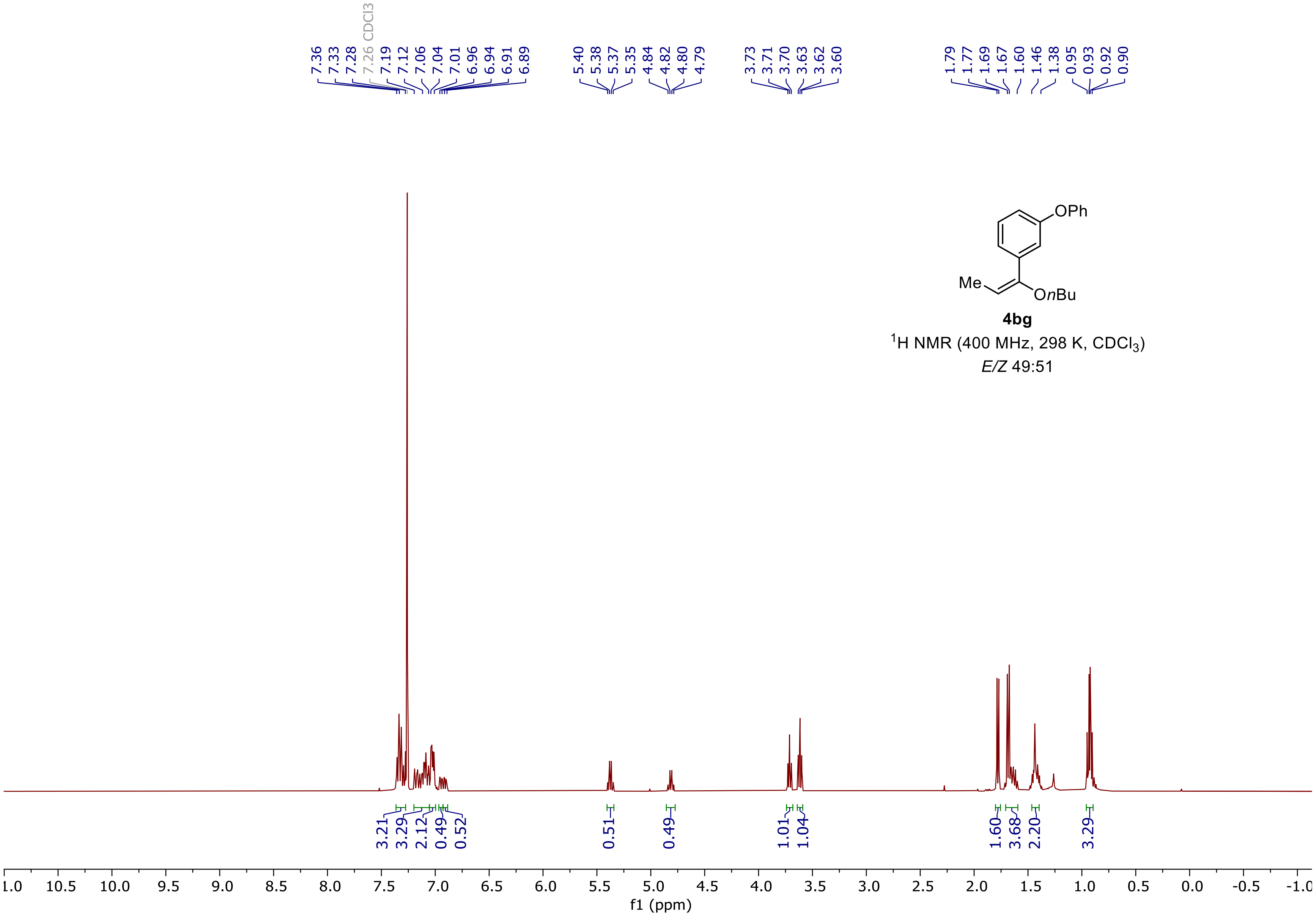
7.36
7.33
7.28
7.26 CDCl3
7.19
7.12
7.06
7.04
7.01
6.96
6.94
6.91
6.89
5.40
5.38
5.37
5.35
4.84
4.82
4.80
4.79
3.73
3.71
3.70
3.63
3.62
3.60
1.79
1.77
1.69
1.67
1.60
1.46
1.38
0.95
0.93
0.92
0.90
OPh
Me
OnBu
4bg
1H NMR (400 MHz, 298 K, CDCl3)
E/Z 49:51
3.21
3.29
2.12
0.49
0.52
0.51
0.49
1.01
1.04
1.60
3.68
2.20
3.29
f1 (ppm)

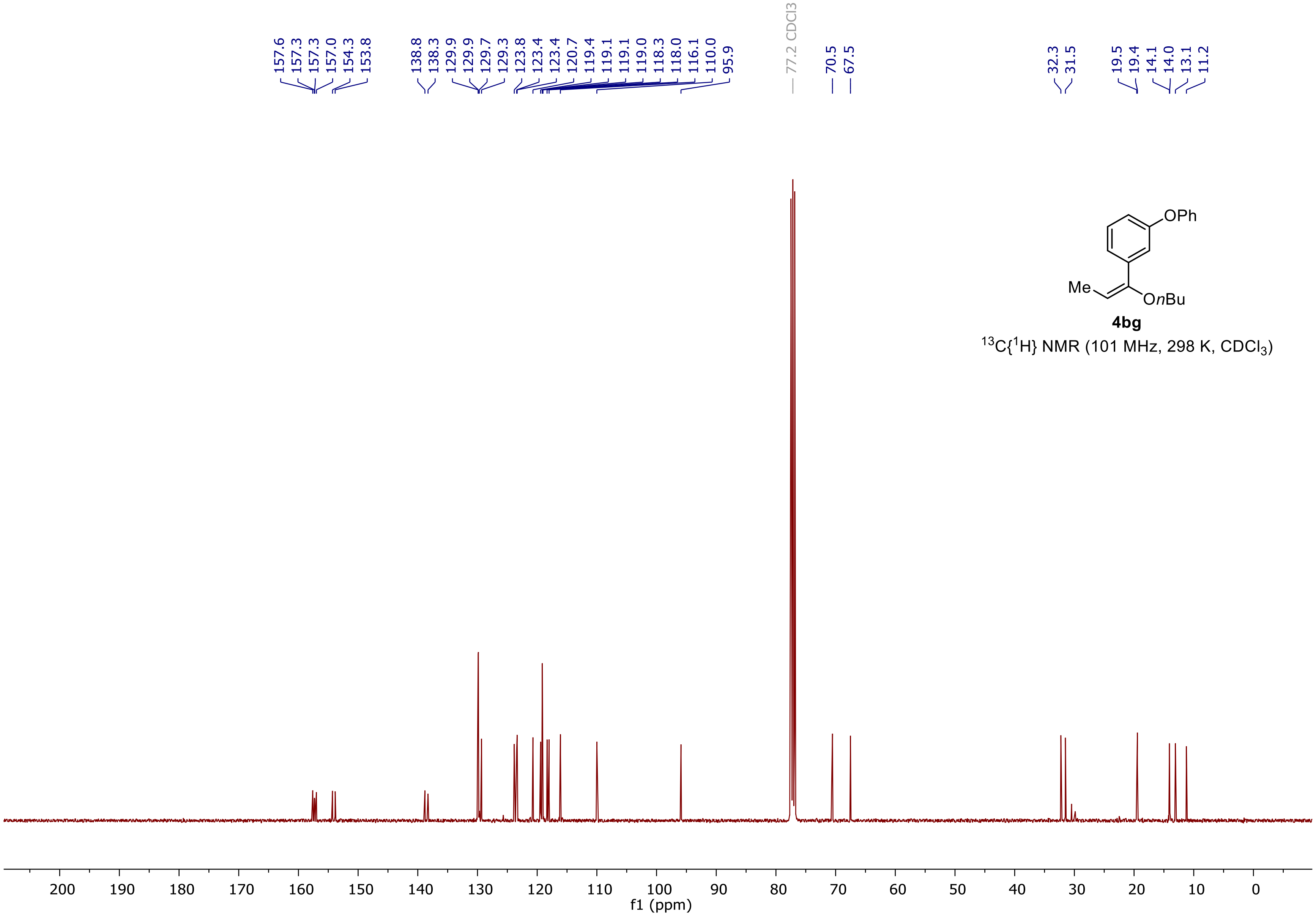

4bg

$^{13}C\{^{1}H\}$ NMR (101 MHz, 298 K, $CDCl_3$)

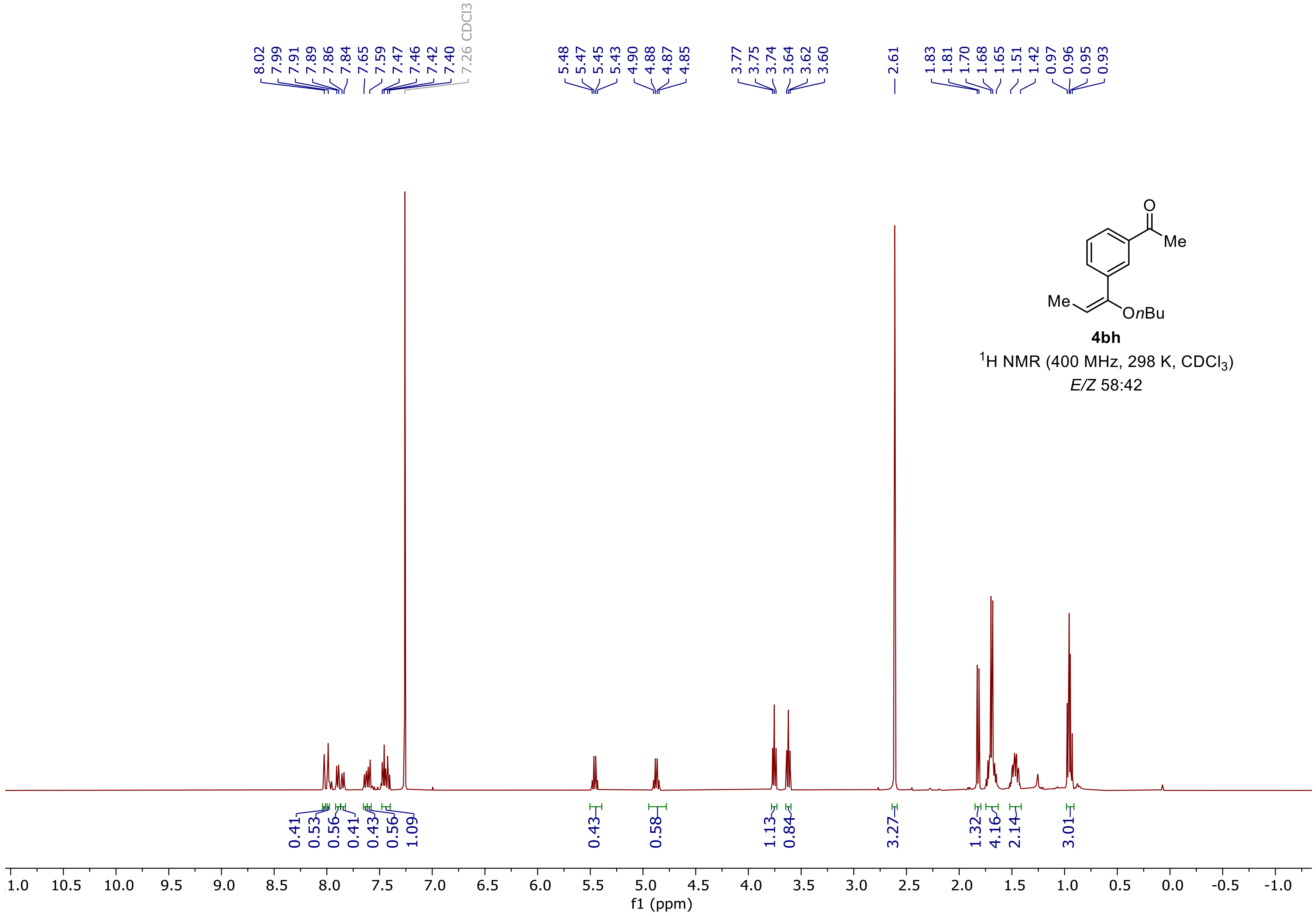

4bh

^{1}H NMR (400 MHz, 298 K, $CDCl_3$)

E/Z 58:42

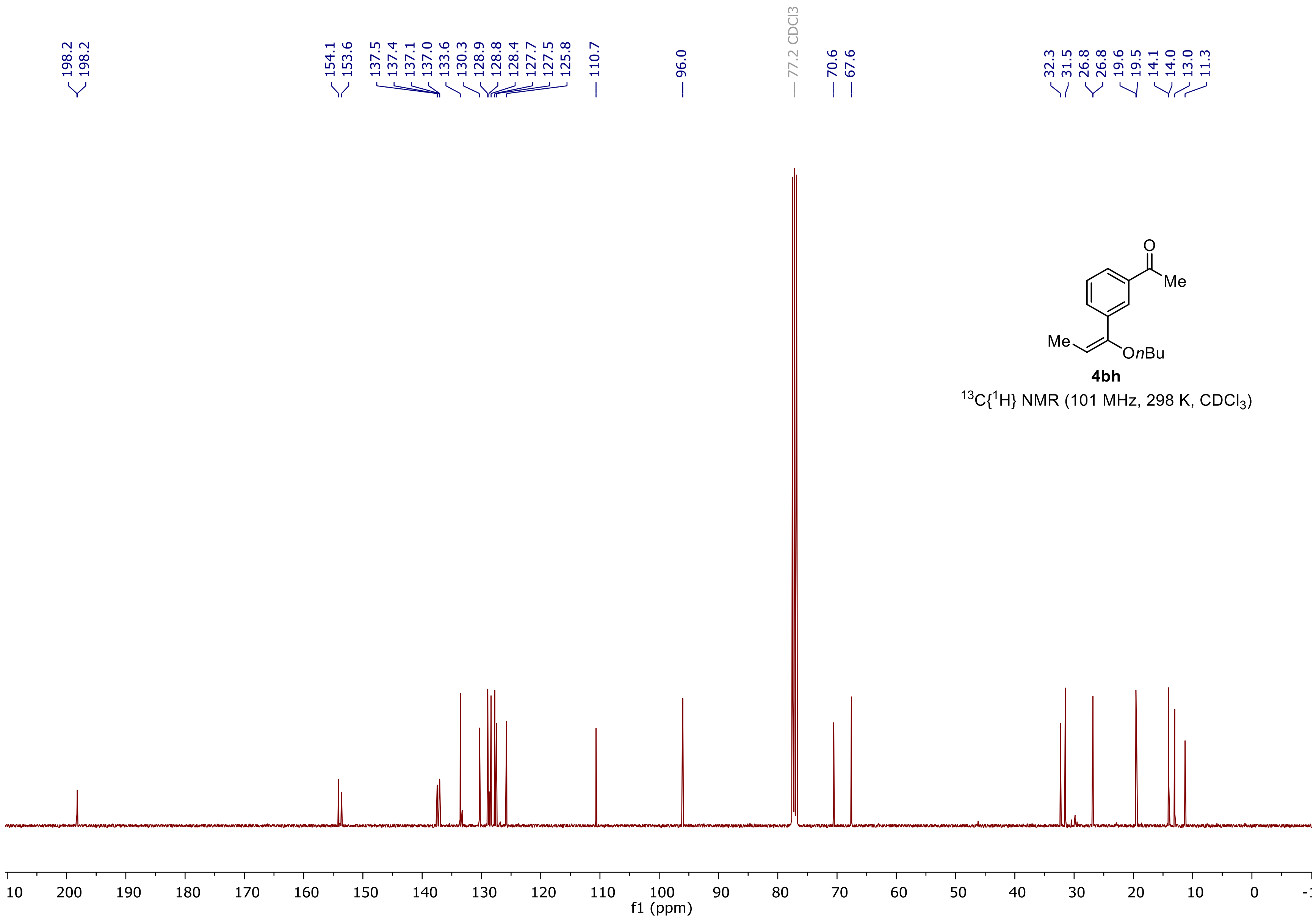

4bh

$^{13}C\{^{1}H\}$ NMR (101 MHz, 298 K, $CDCl_3$)

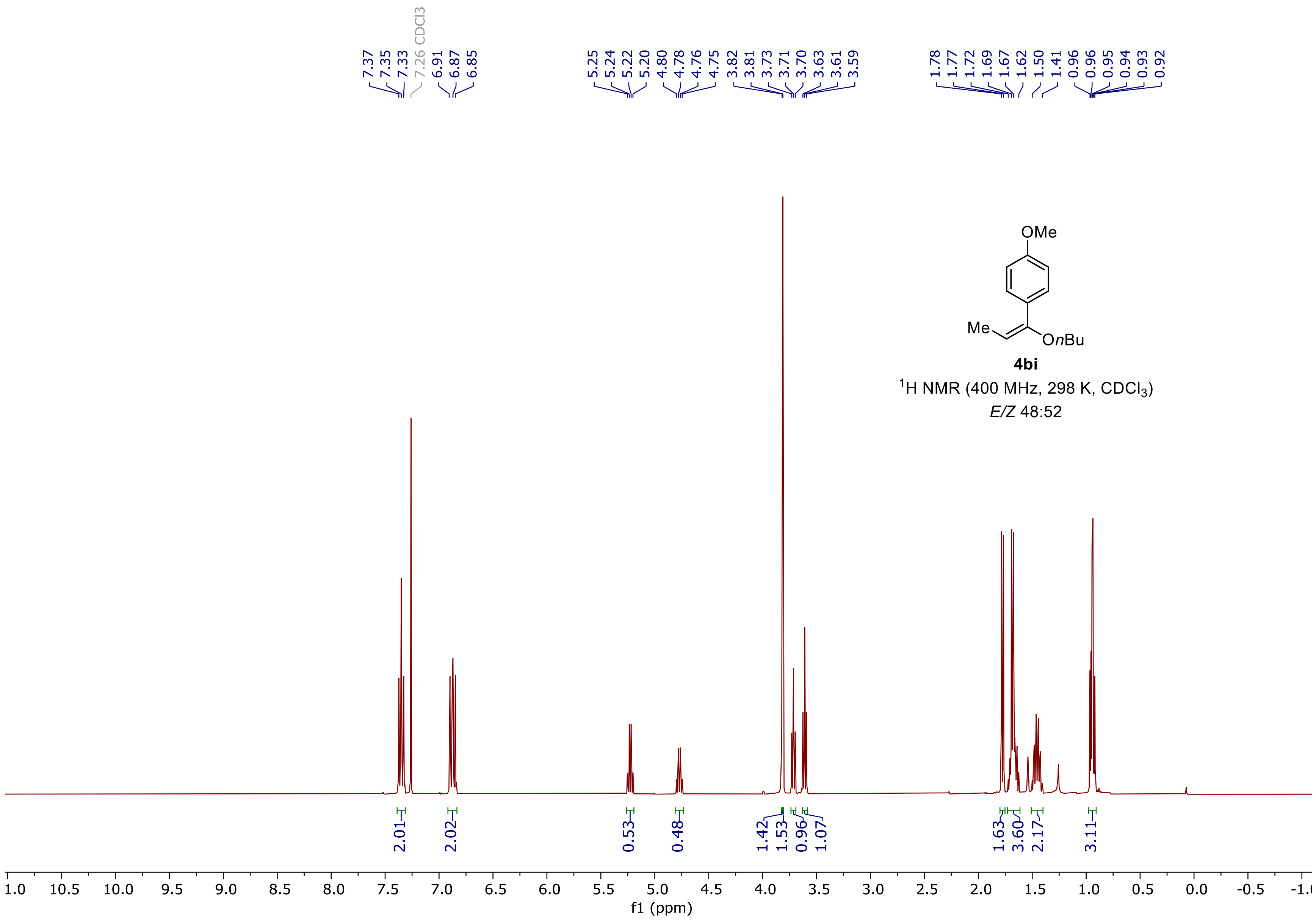

4bi

^{1}H NMR (400 MHz, 298 K, $CDCl_3$)

E/Z 48:52

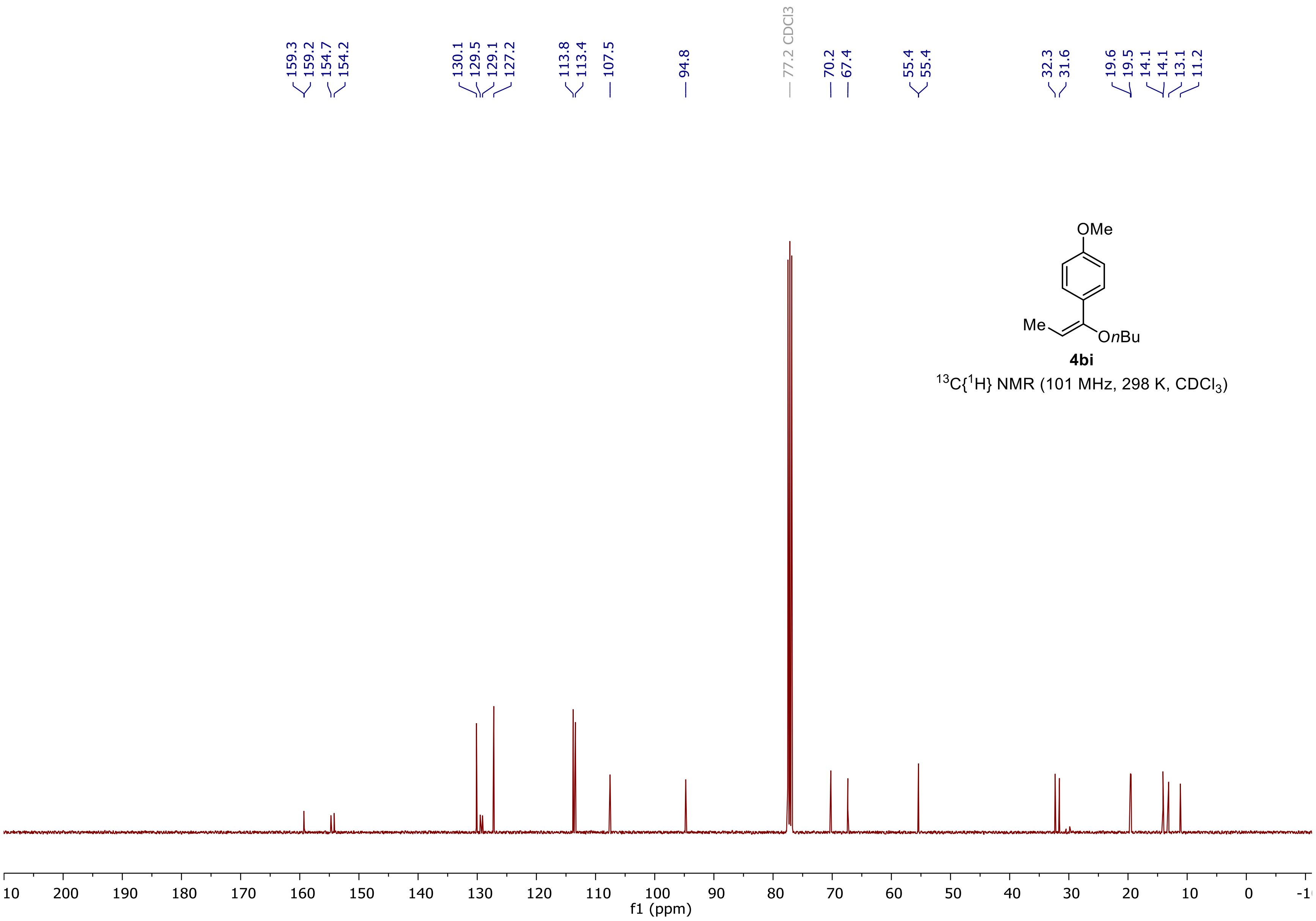
159.3
159.2
154.7
154.2
130.1
129.5
129.1
127.2
113.8
113.4
107.5
94.8
77.2 CDCl3
70.2
67.4
55.4
55.4
32.3
31.6
19.6
19.5
14.1
14.1
13.1
11.2
OMe
Me
OnBu
4bi
13C{1H} NMR (101 MHz, 298 K, CDCl3)
f1 (ppm)

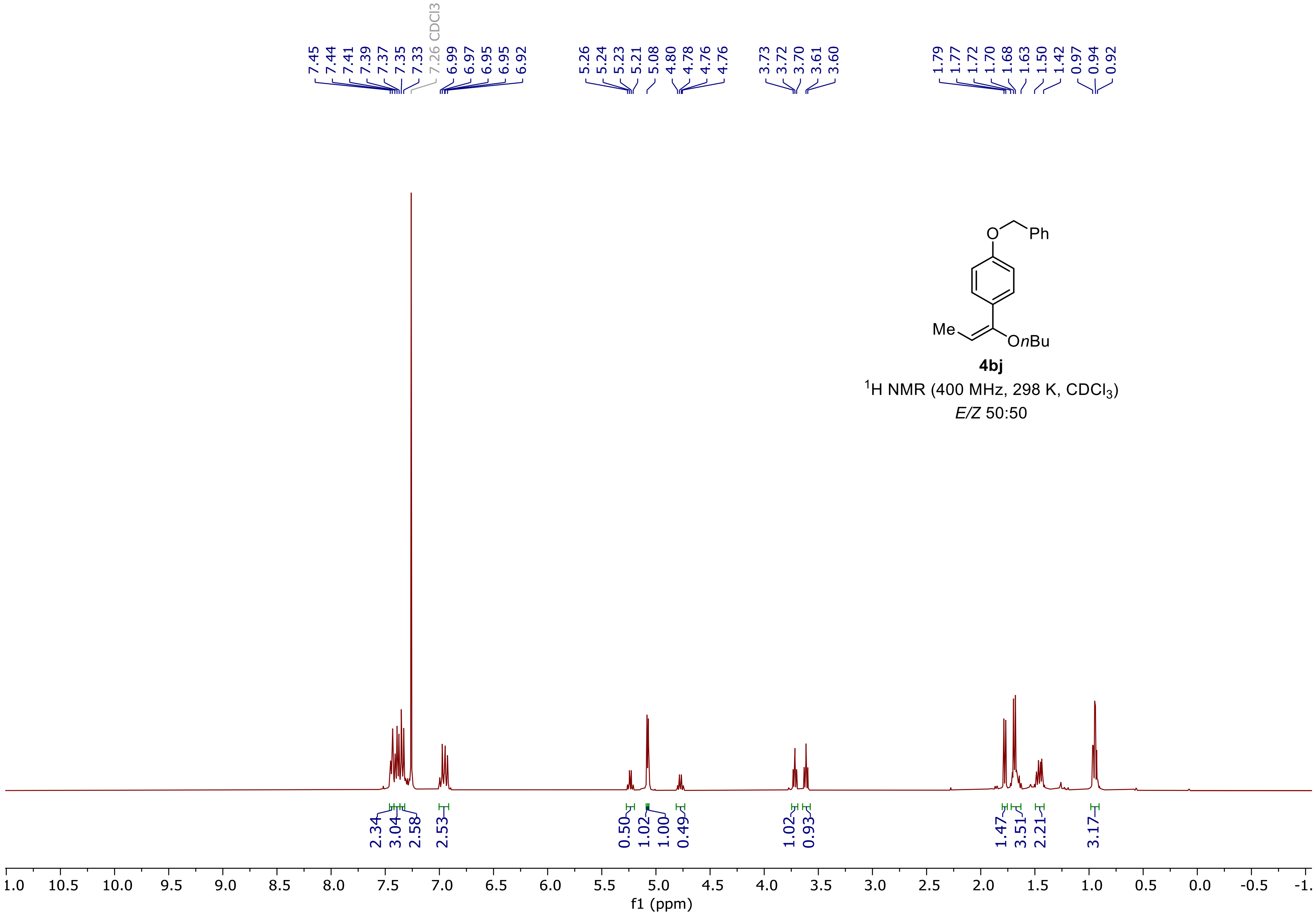

4bj

^{1}H NMR (400 MHz, 298 K, $CDCl_3$)

E/Z 50:50

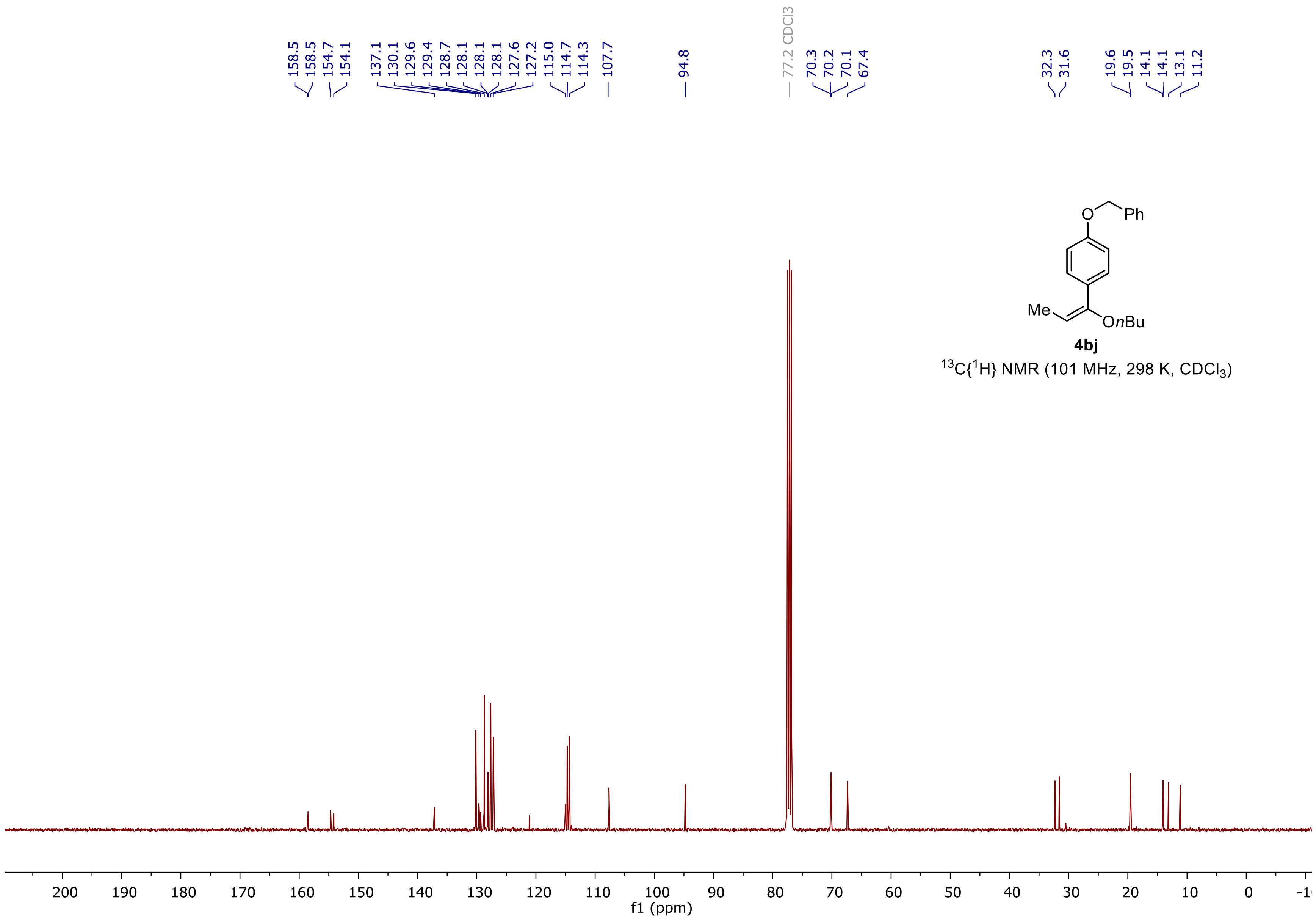

4bj

$^{13}C\{^{1}H\}$ NMR (101 MHz, 298 K, $CDCl_3$)

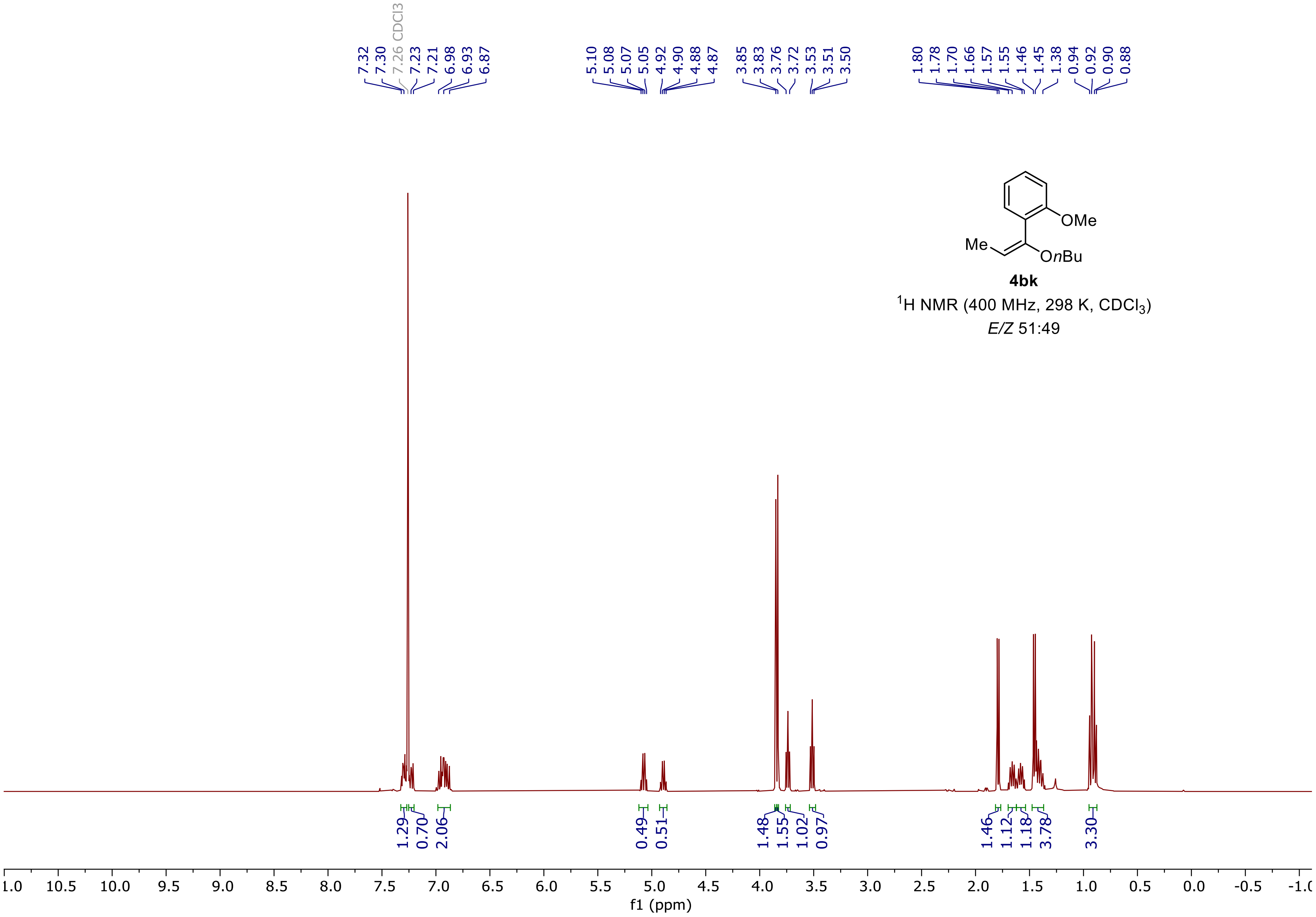

4bk

^{1}H NMR (400 MHz, 298 K, $CDCl_3$)

E/Z 51:49

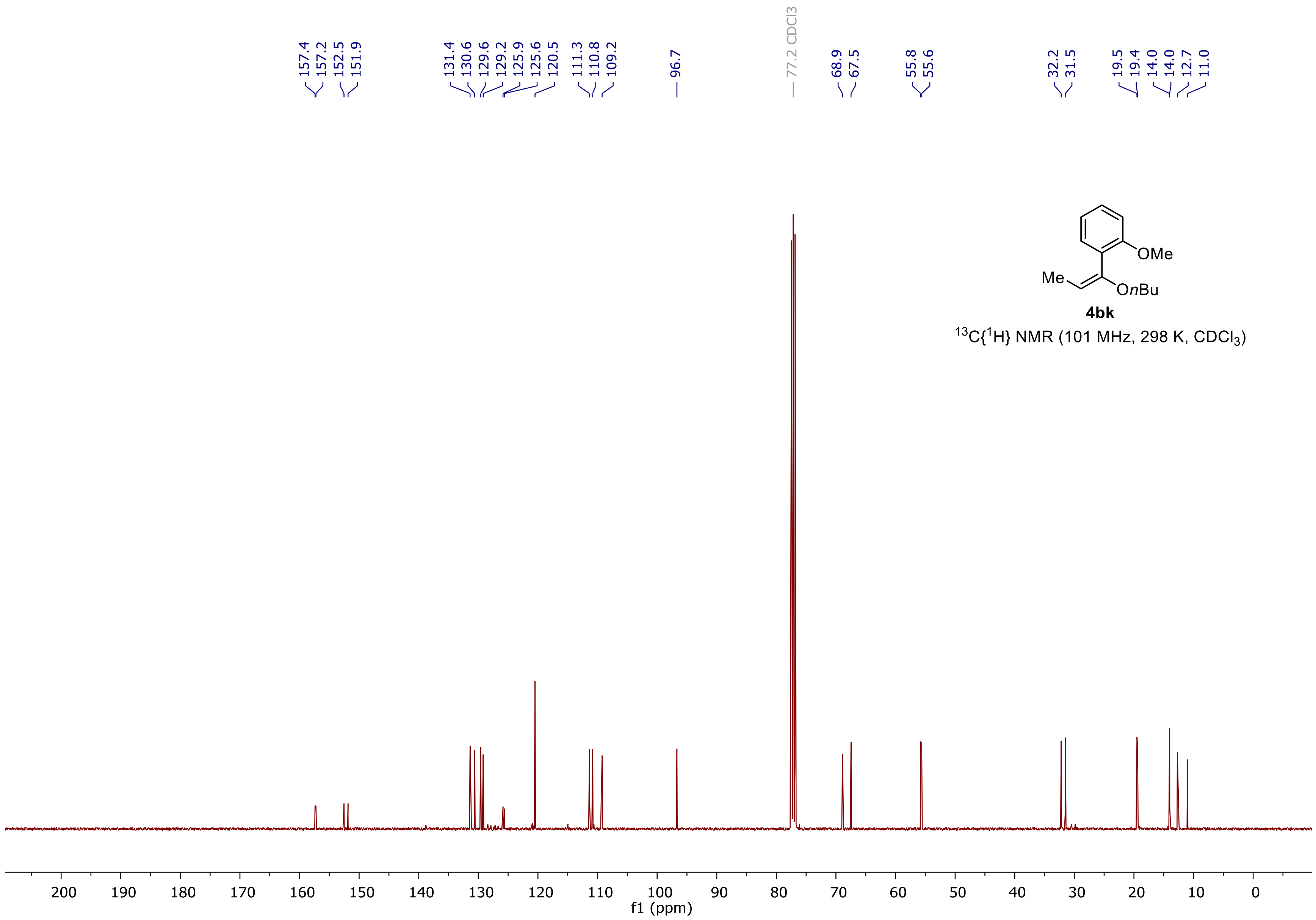

4bk

$^{13}C\{^{1}H\}$ NMR (101 MHz, 298 K, $CDCl_3$)

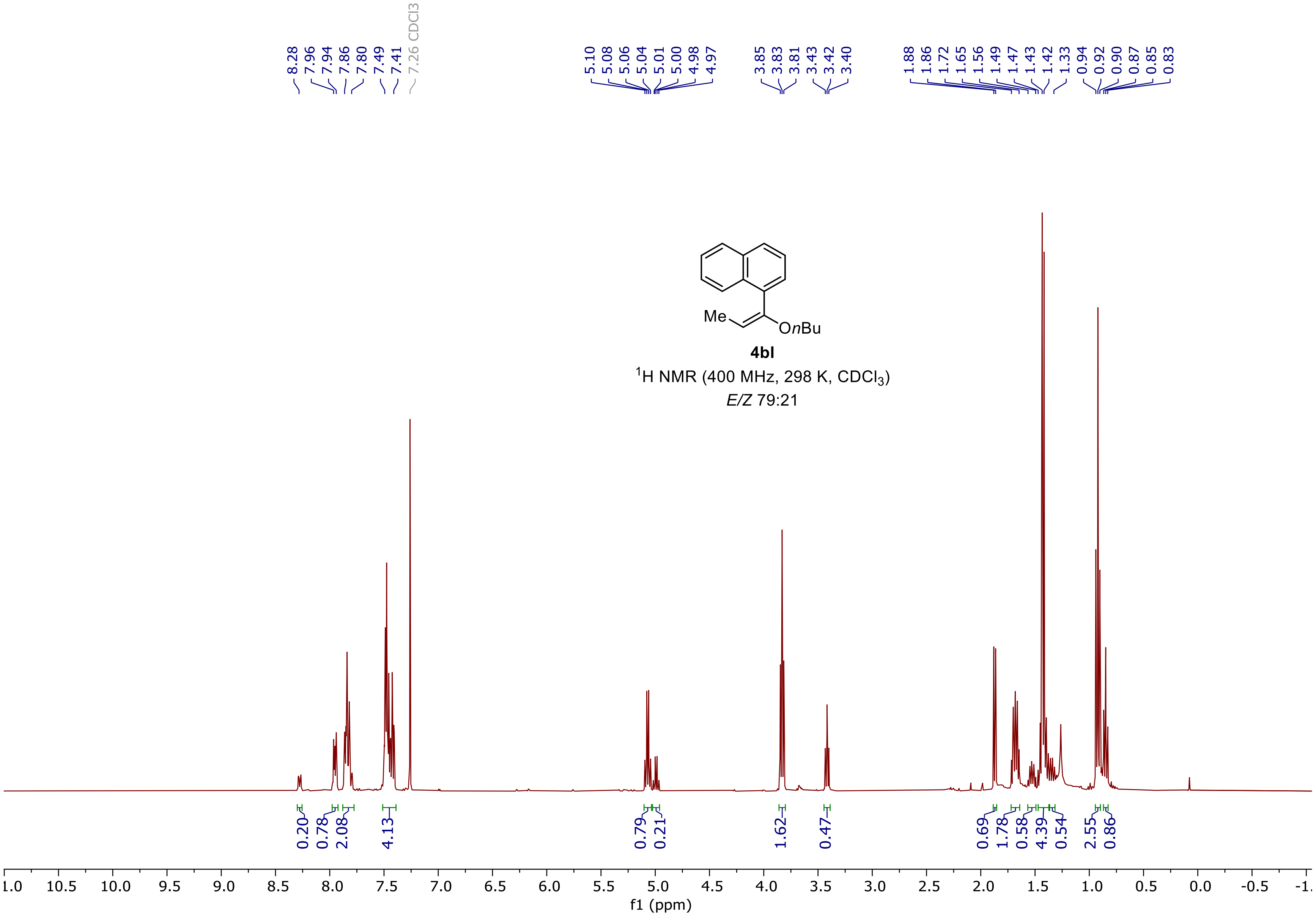

8.28
7.96
7.94
7.86
7.80
7.49
7.41
7.26 CDCl3
5.10
5.08
5.06
5.04
5.01
5.00
4.98
4.97
3.85
3.83
3.81
3.43
3.42
3.40
1.88
1.86
1.72
1.65
1.56
1.49
1.47
1.43
1.42
1.33
0.94
0.92
0.90
0.87
0.85
0.83
Me
OnBu
4bl
^{1}H NMR (400 MHz, 298 K, $CDCl_3$)
E/Z 79:21
0.20
0.78
2.08
4.13
0.79
0.21
1.62
0.47
0.69
1.78
0.58
4.39
0.54
2.55
0.86
f1 (ppm)

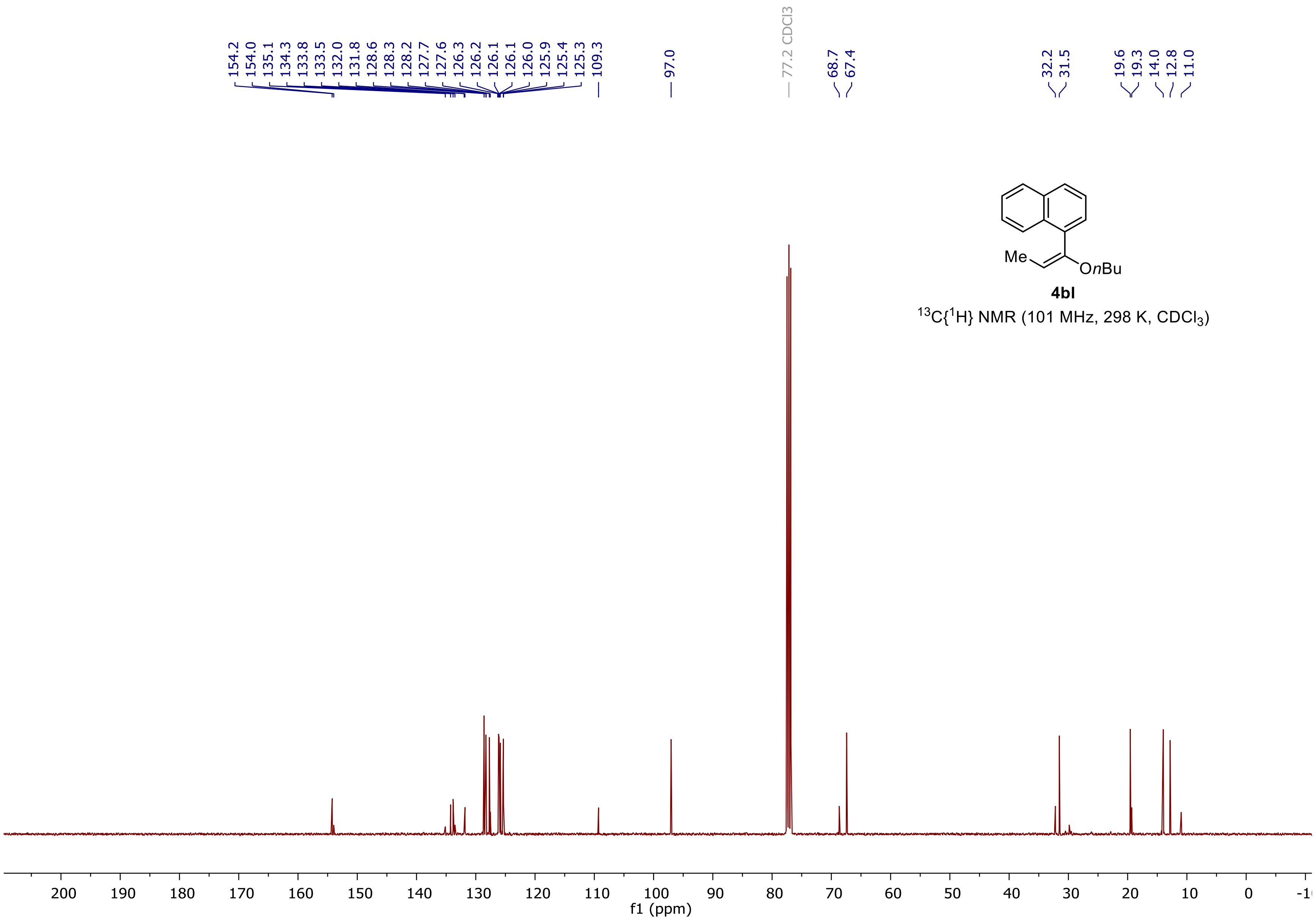

4bl

$^{13}C\{^{1}H\}$ NMR (101 MHz, 298 K, $CDCl_3$)

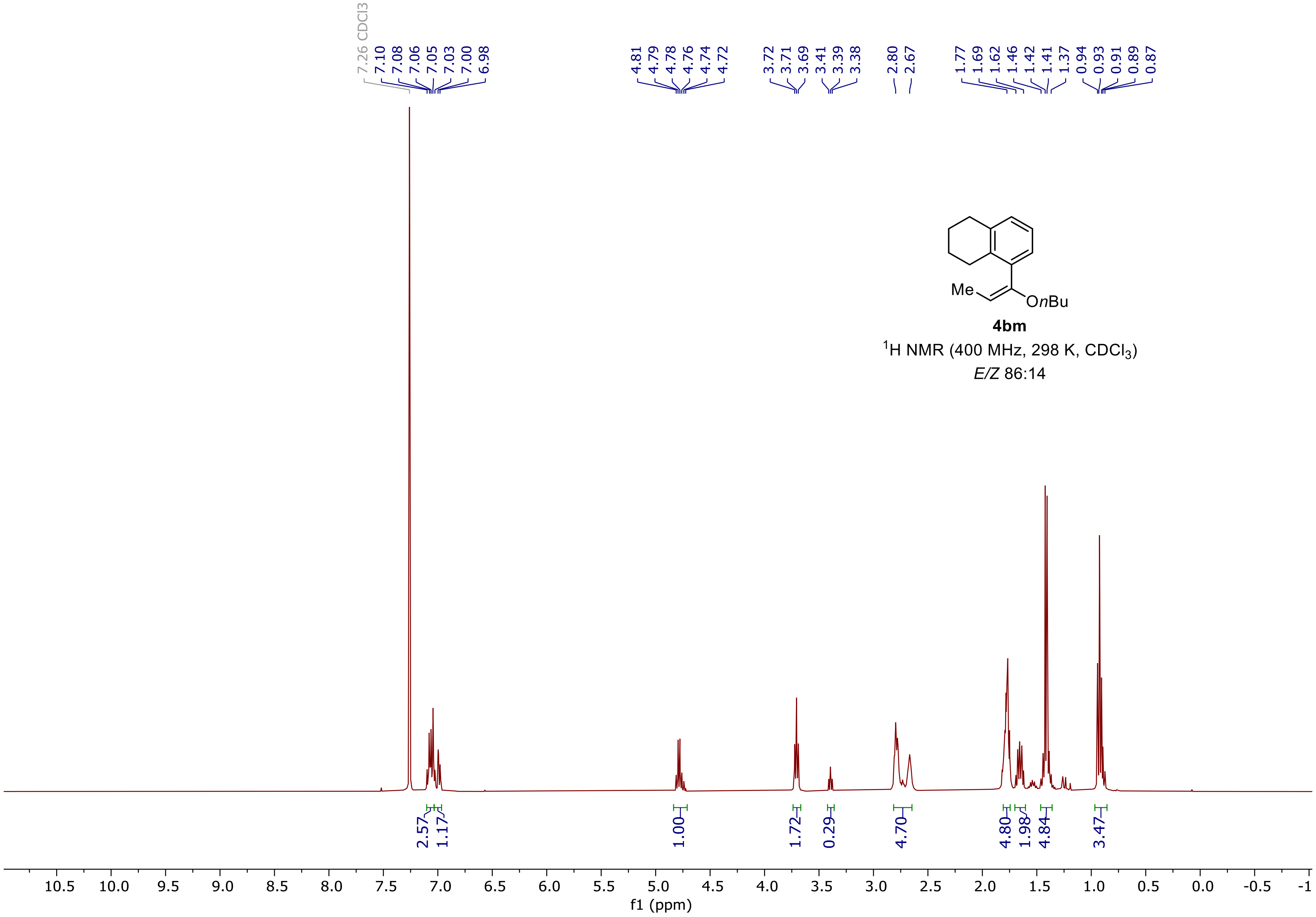

4bm

^{1}H NMR (400 MHz, 298 K, $CDCl_3$)

E/Z 86:14

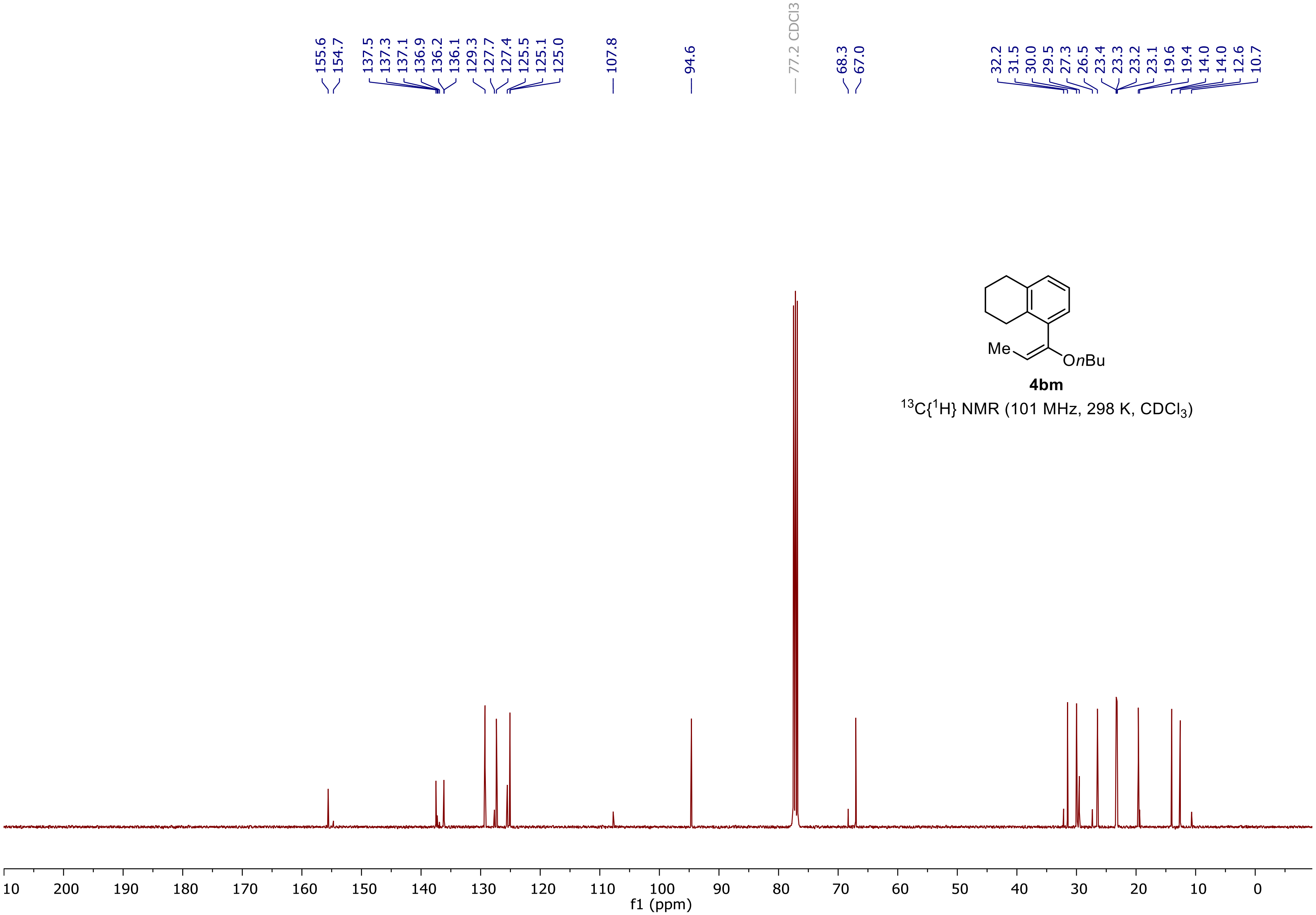

4bm

$^{13}C\{^{1}H\}$ NMR (101 MHz, 298 K, $CDCl_3$)

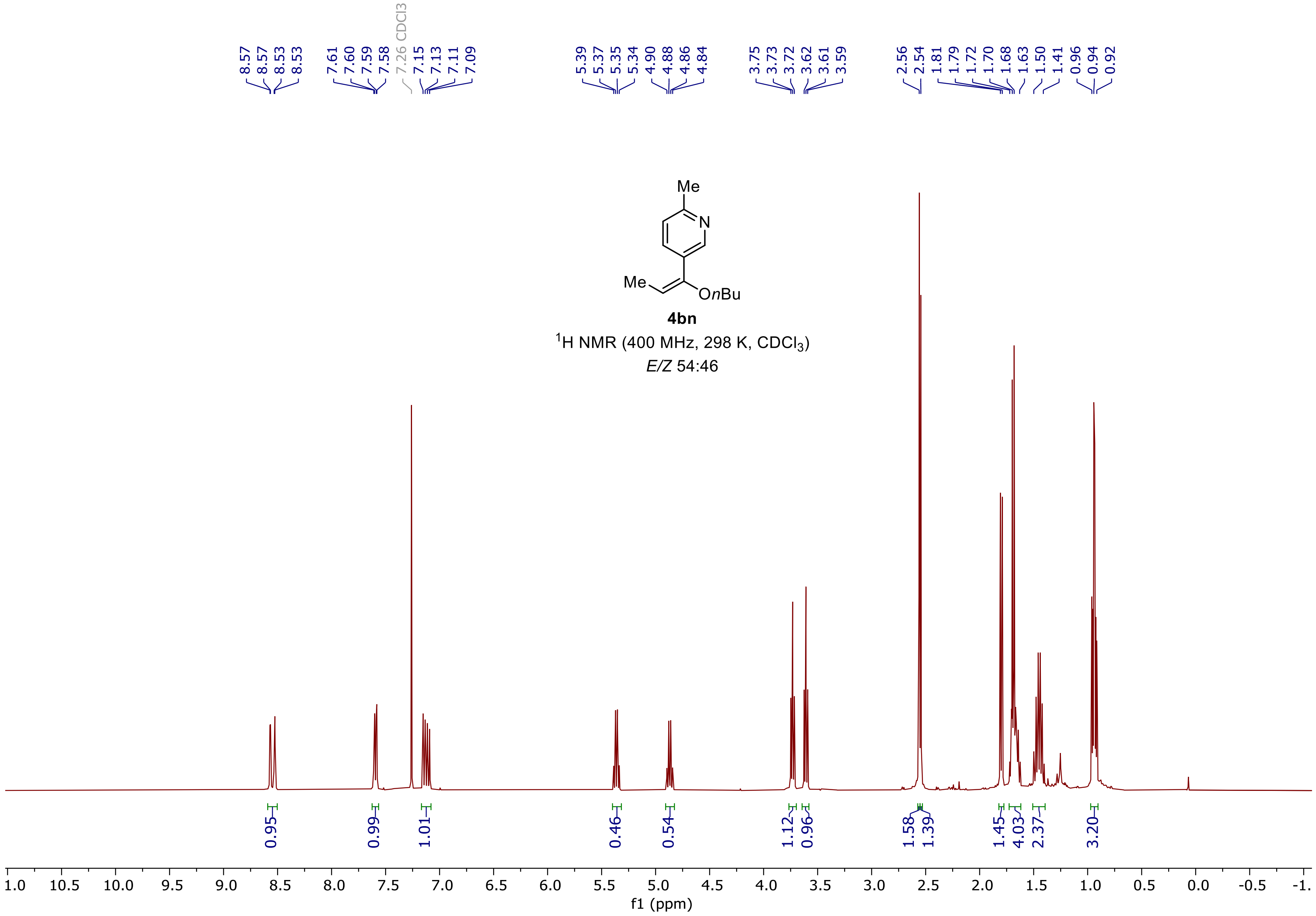
8.57
8.57
8.53
8.53
7.61
7.60
7.59
7.58
7.26 CDCl3
7.15
7.13
7.11
7.09
5.39
5.37
5.35
5.34
4.90
4.88
4.86
4.84
3.75
3.73
3.72
3.62
3.61
3.59
2.56
2.54
1.81
1.79
1.72
1.70
1.68
1.63
1.50
1.41
0.96
0.94
0.92
Me
N
Me
OnBu
4bn
1H NMR (400 MHz, 298 K, CDCl3)
E/Z 54:46
0.95
0.99
1.01
0.46
0.54
1.12
0.96
1.58
1.39
1.45
4.03
2.37
3.20
f1 (ppm)

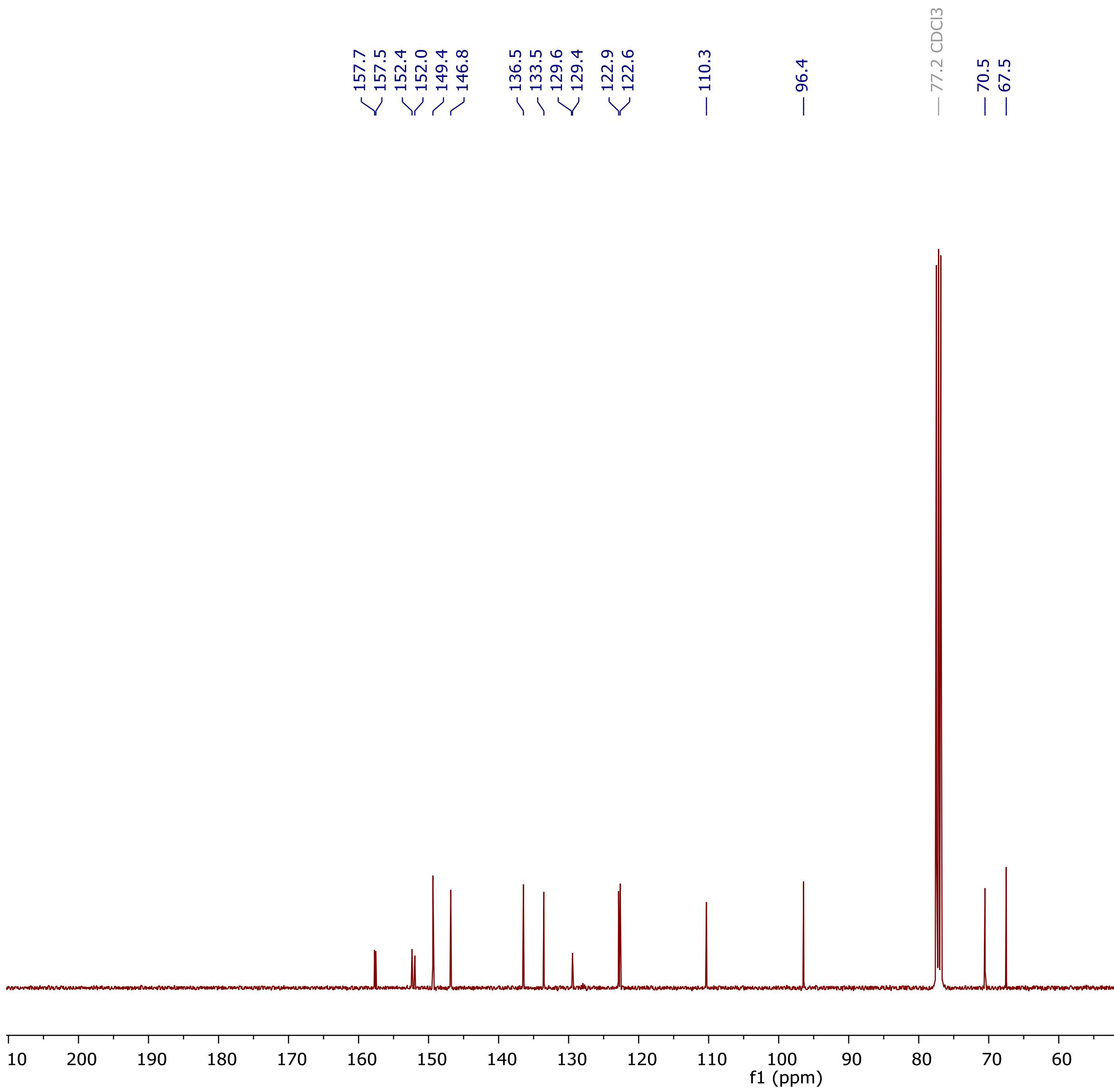

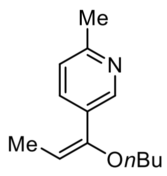

4bn

$^{13}C\{^{1}H\}$ NMR (101 MHz, 298 K, $CDCl_3$)

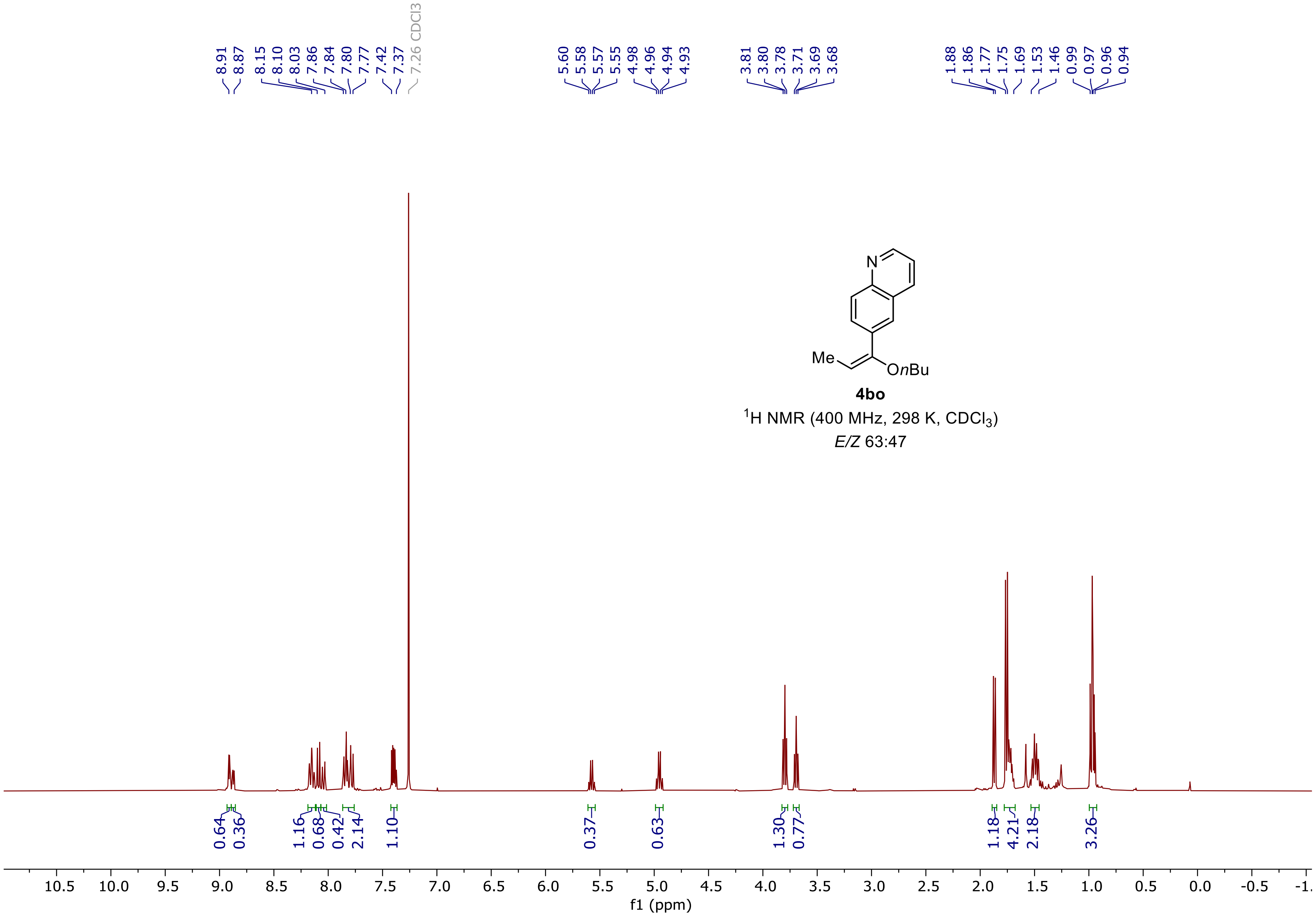

4bo

^{1}H NMR (400 MHz, 298 K, $CDCl_3$)

E/Z 63:47

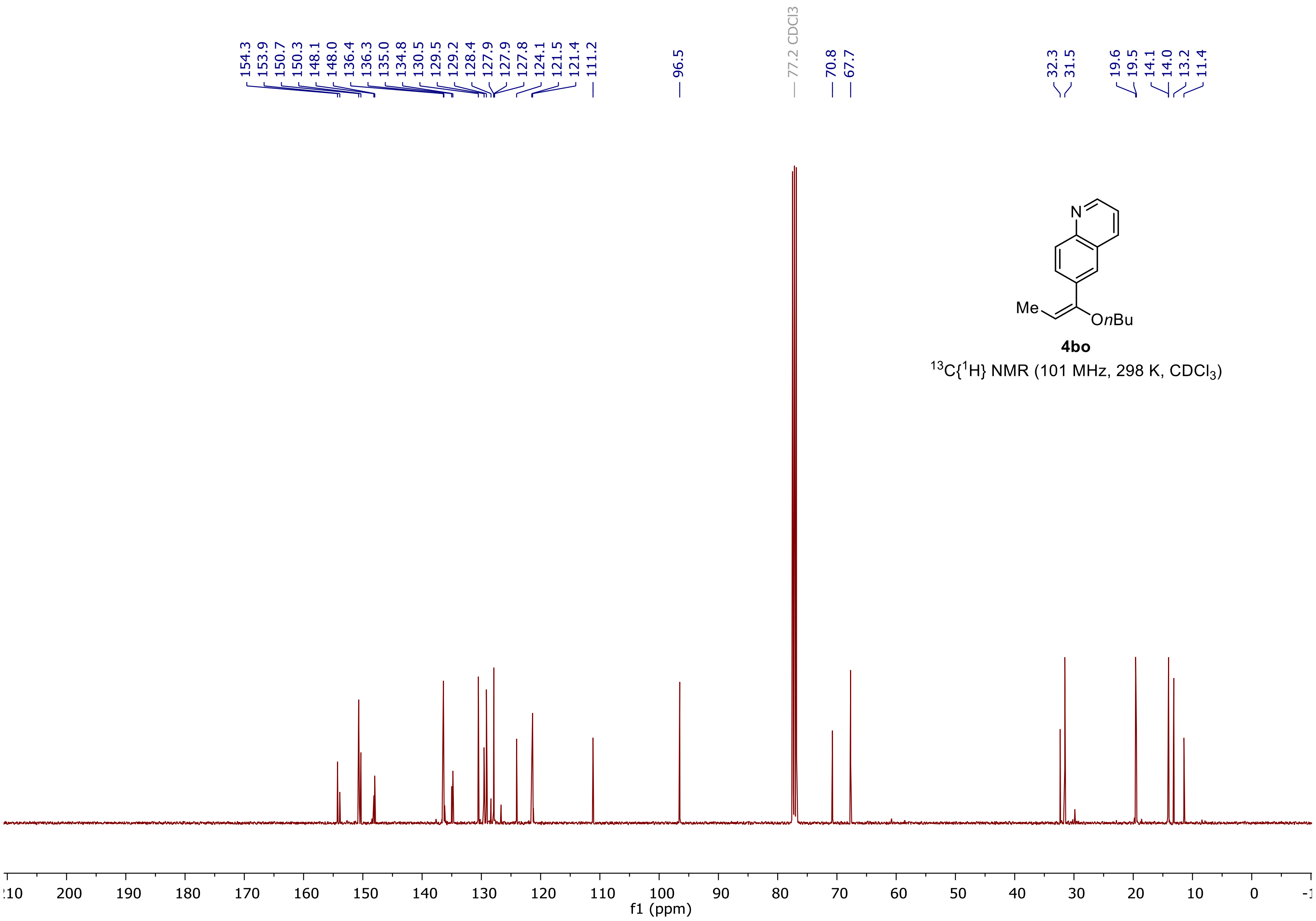

4bo

$^{13}C\{^{1}H\}$ NMR (101 MHz, 298 K, $CDCl_3$)

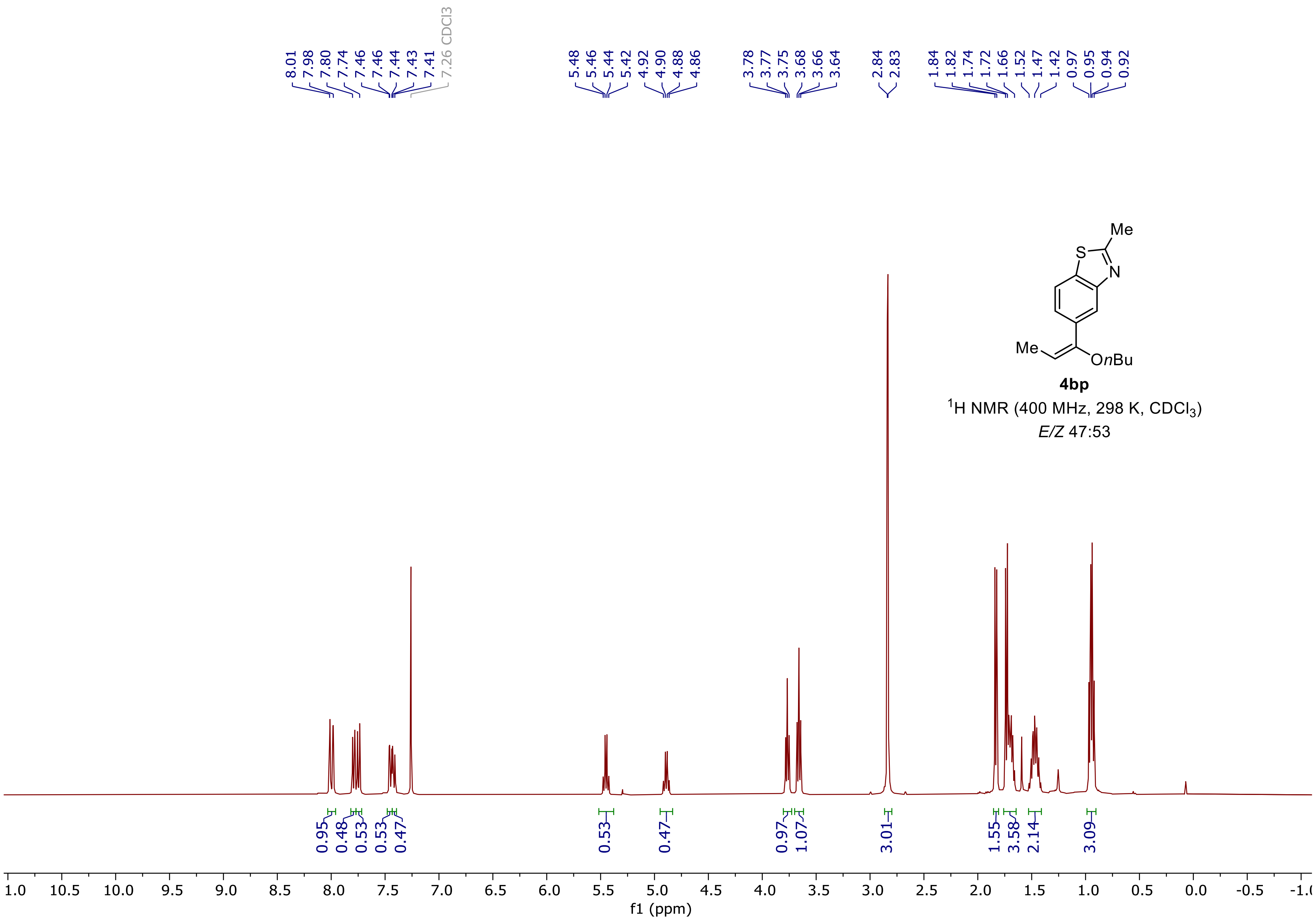

4bp

^1H NMR (400 MHz, 298 K, $CDCl_3$)

E/Z 47:53

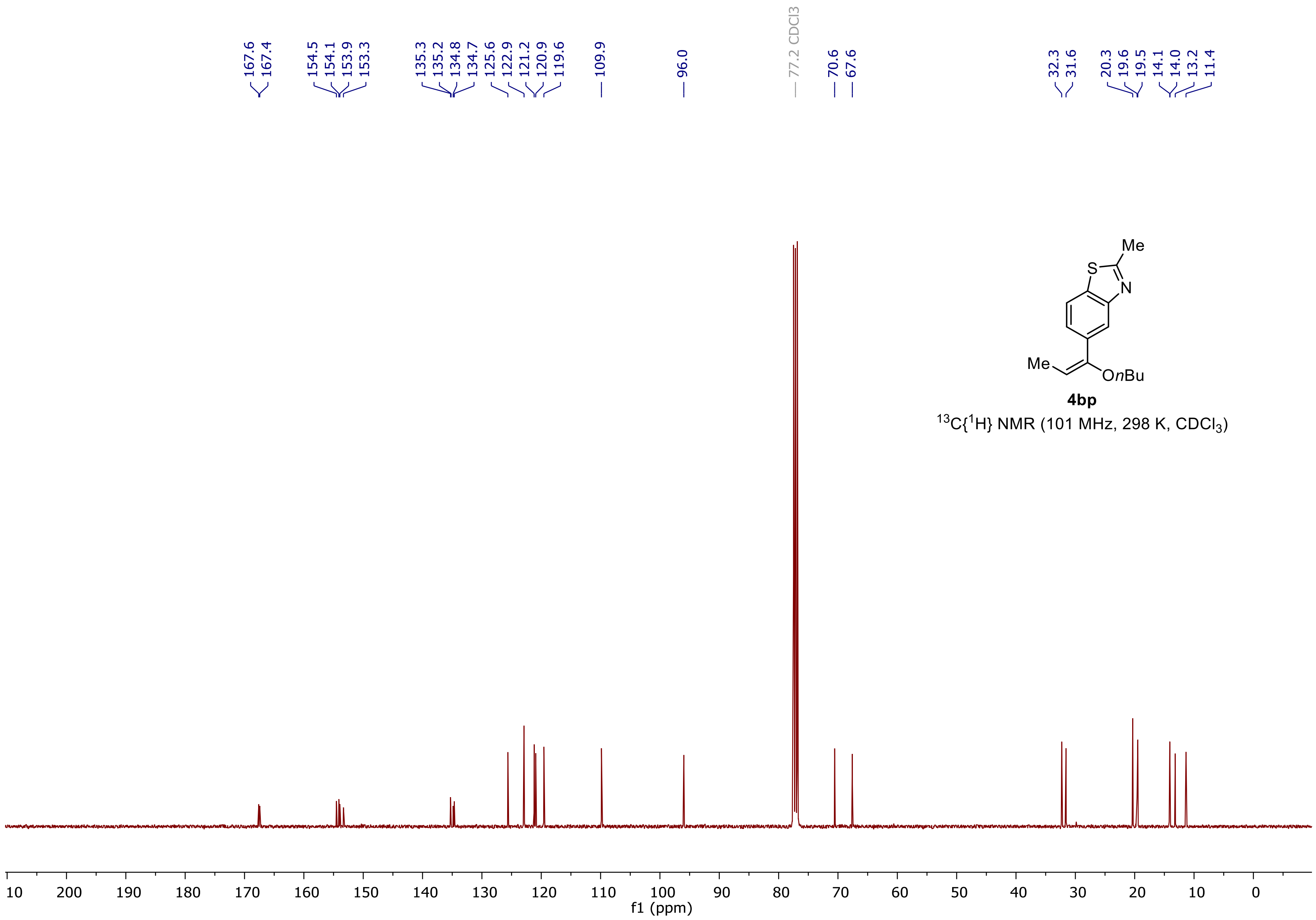

4bp

$^{13}C\{^{1}H\}$ NMR (101 MHz, 298 K, $CDCl_3$)

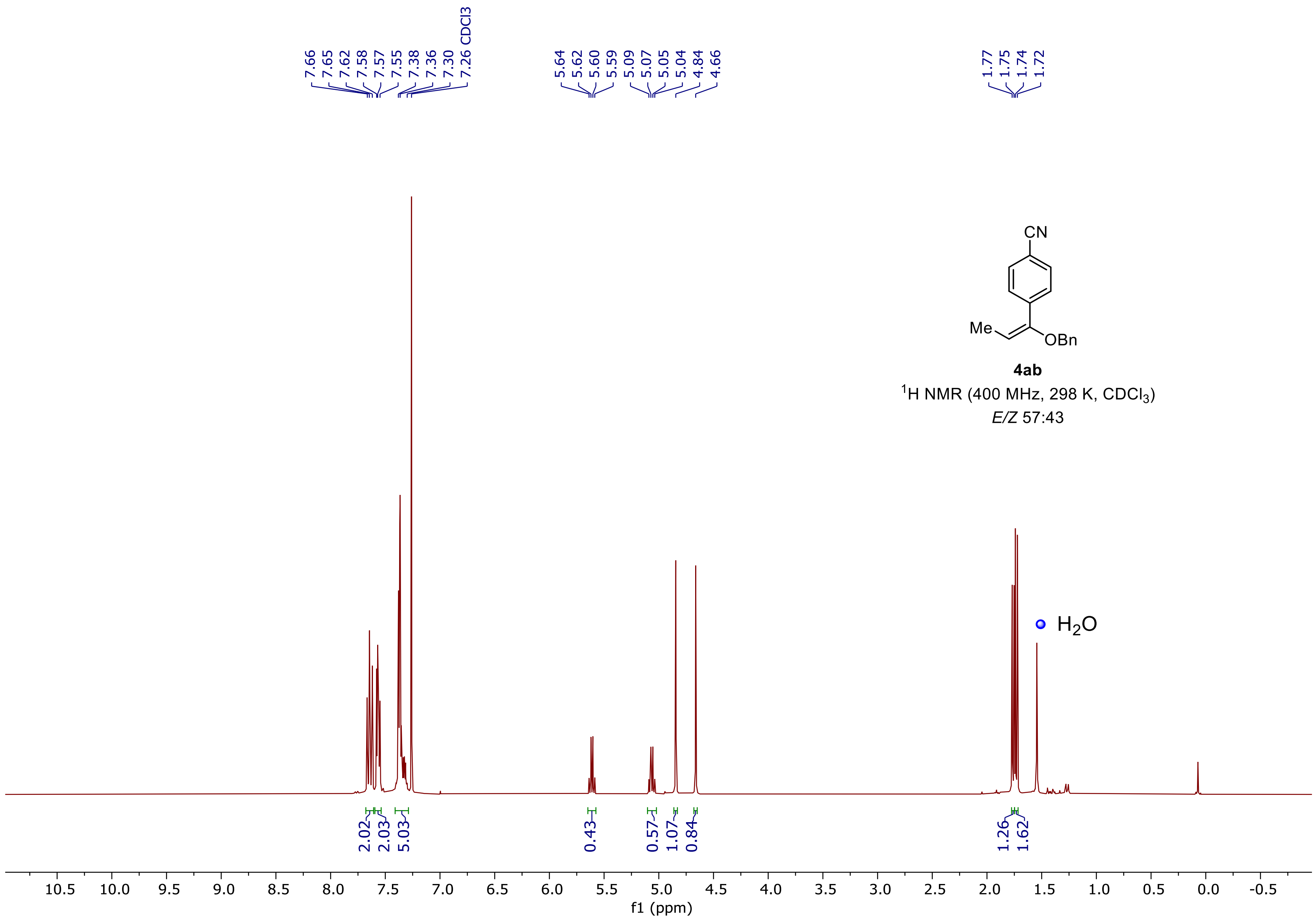

7.66 7.65 7.62 7.58 7.57 7.55 7.38 7.36 7.30 7.26 CDCl3
5.64 5.62 5.60 5.59 5.09 5.07 5.05 5.04 4.84 4.66
1.77 1.75 1.74 1.72
CN
Me
OBn
4ab
1H NMR (400 MHz, 298 K, CDCl3)
E/Z 57:43
H2O
2.02 2.03 5.03 0.43 0.57 1.07 0.84 1.26 1.62
10.5 10.0 9.5 9.0 8.5 8.0 7.5 7.0 6.5 6.0 5.5 5.0 4.5 4.0 3.5 3.0 2.5 2.0 1.5 1.0 0.5 0.0 -0.5
f1 (ppm)

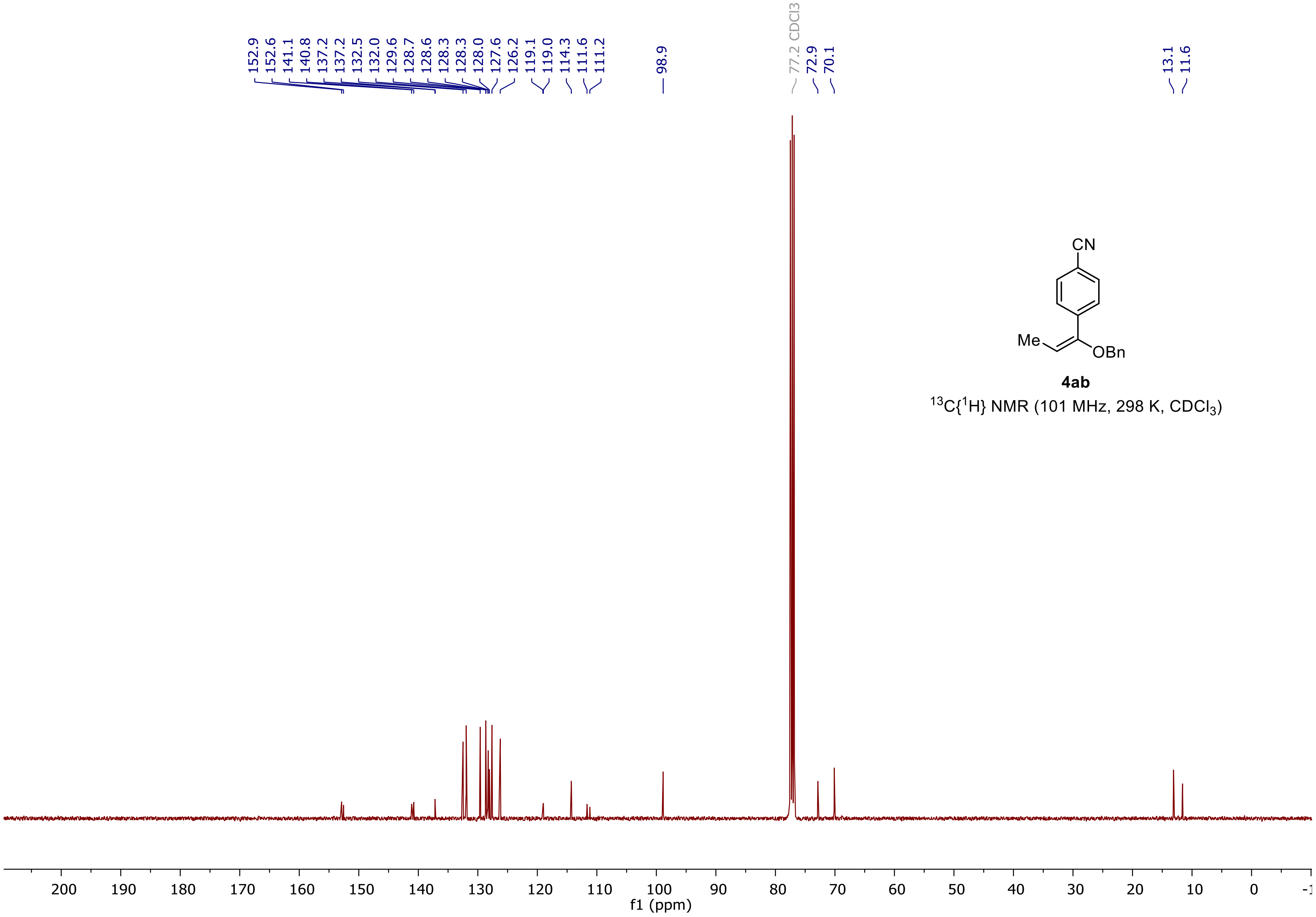

4ab

$^{13}C\{^{1}H\}$ NMR (101 MHz, 298 K, $CDCl_3$)

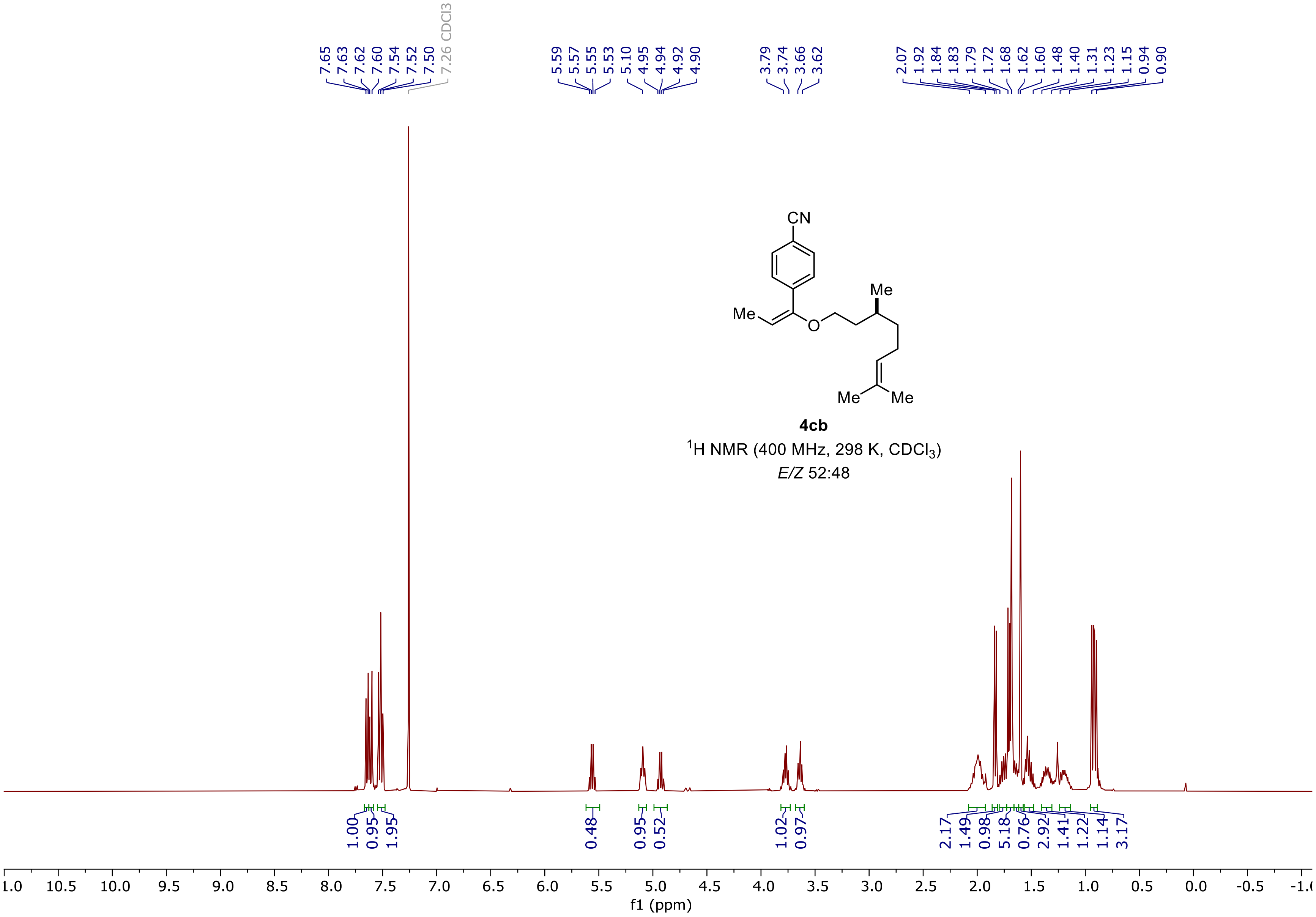

4cb

^{1}H NMR (400 MHz, 298 K, $CDCl_3$)

E/Z 52:48

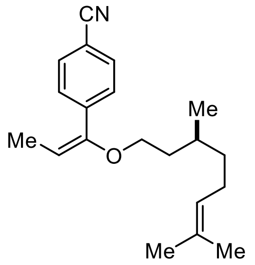

4cb

$^{13}C\{^1H\}$ NMR (101 MHz, 298 K, $CDCl_3$)

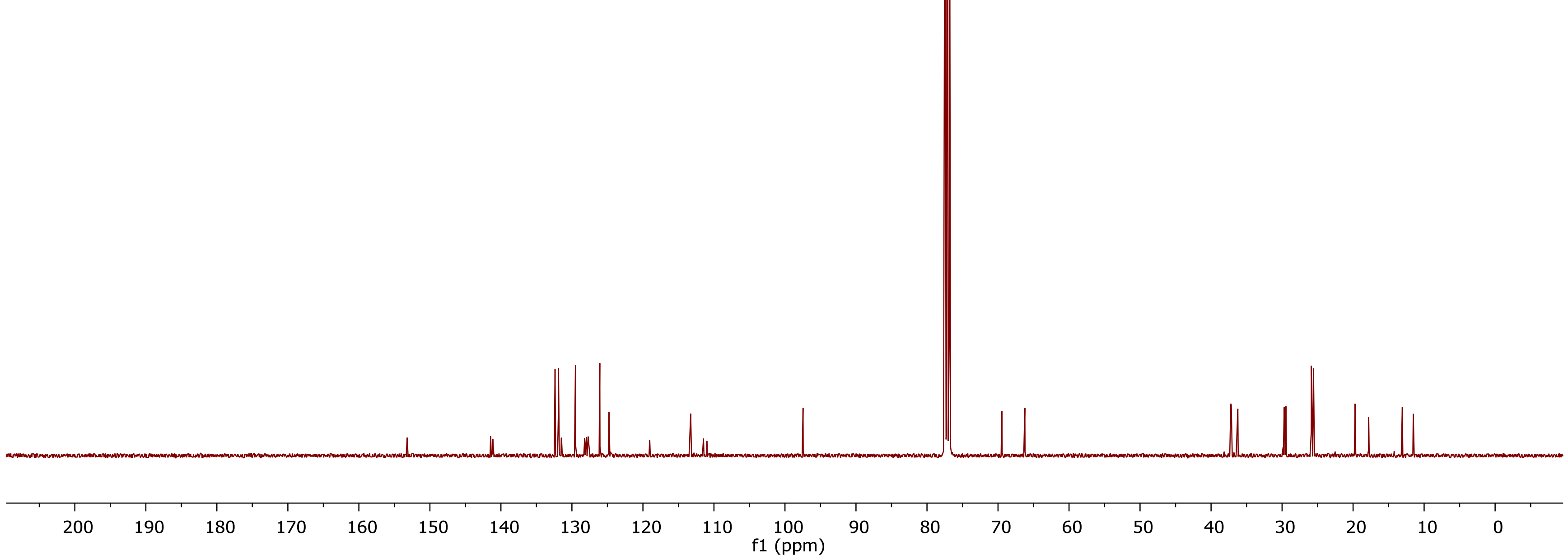

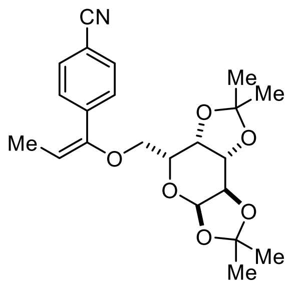

4db

^{1}H NMR (400 MHz, 298 K, $CDCl_3$)

E/Z 68:32

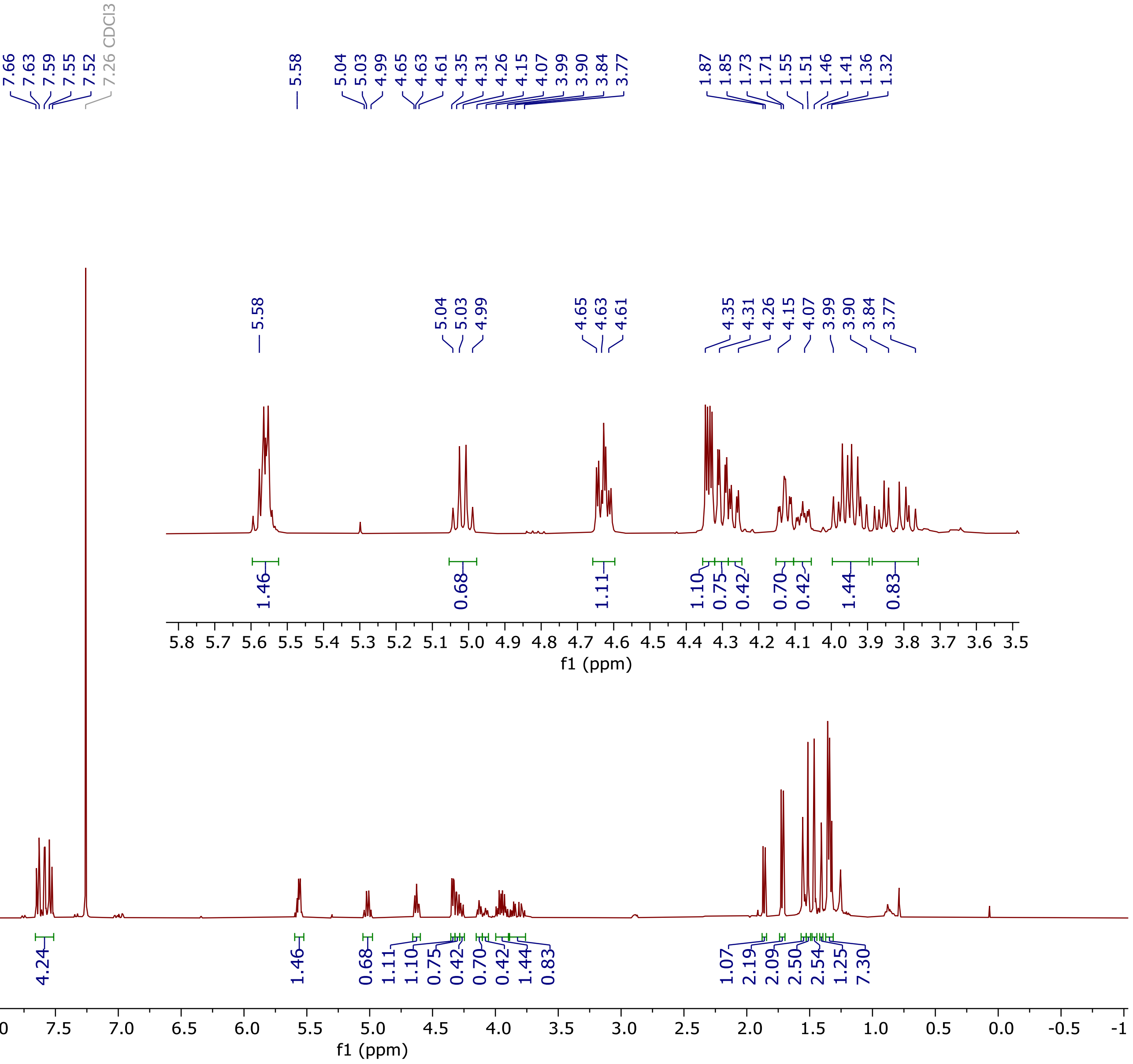

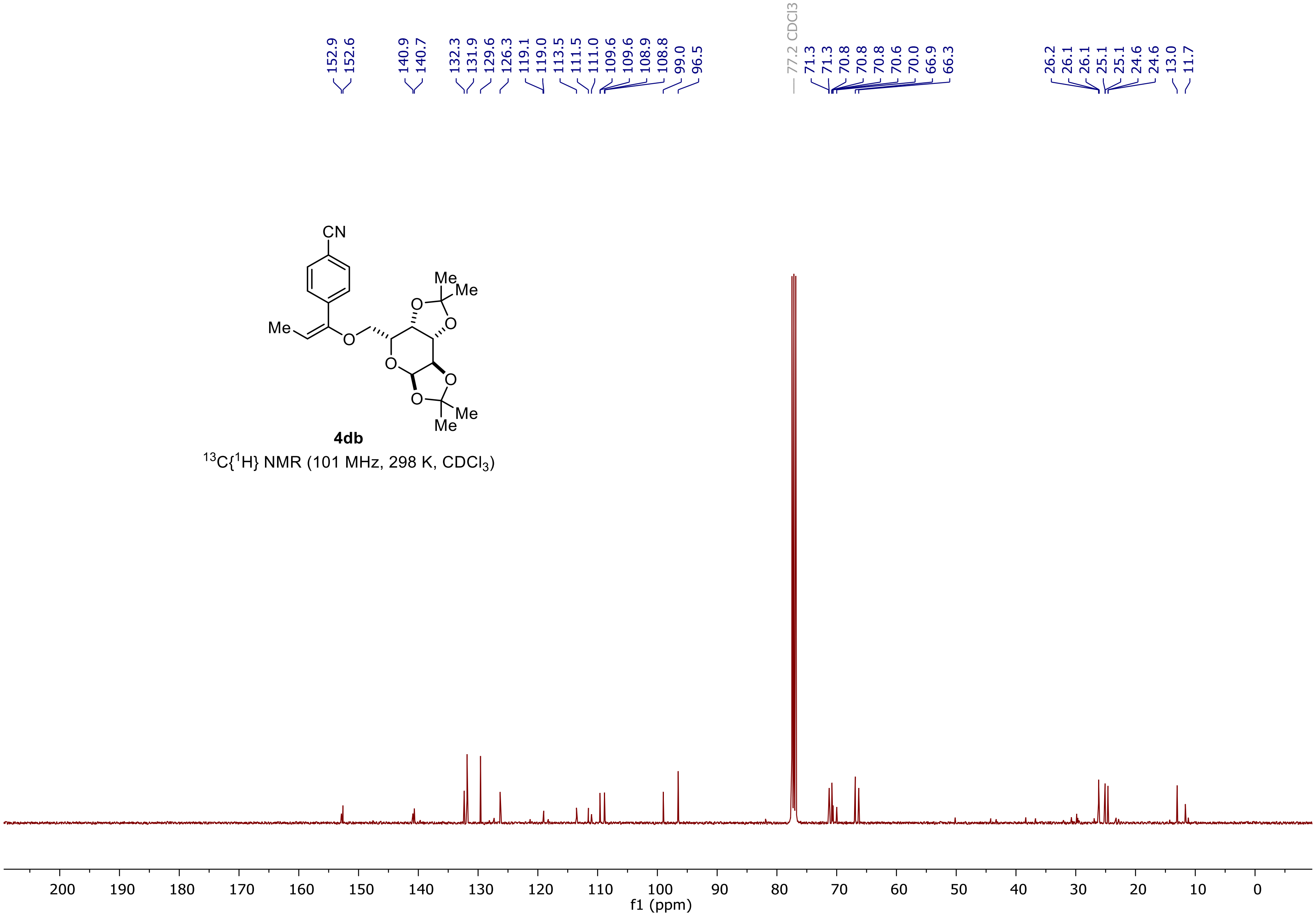

4db

$^{13}C\{^1H\}$ NMR (101 MHz, 298 K, $CDCl_3$)

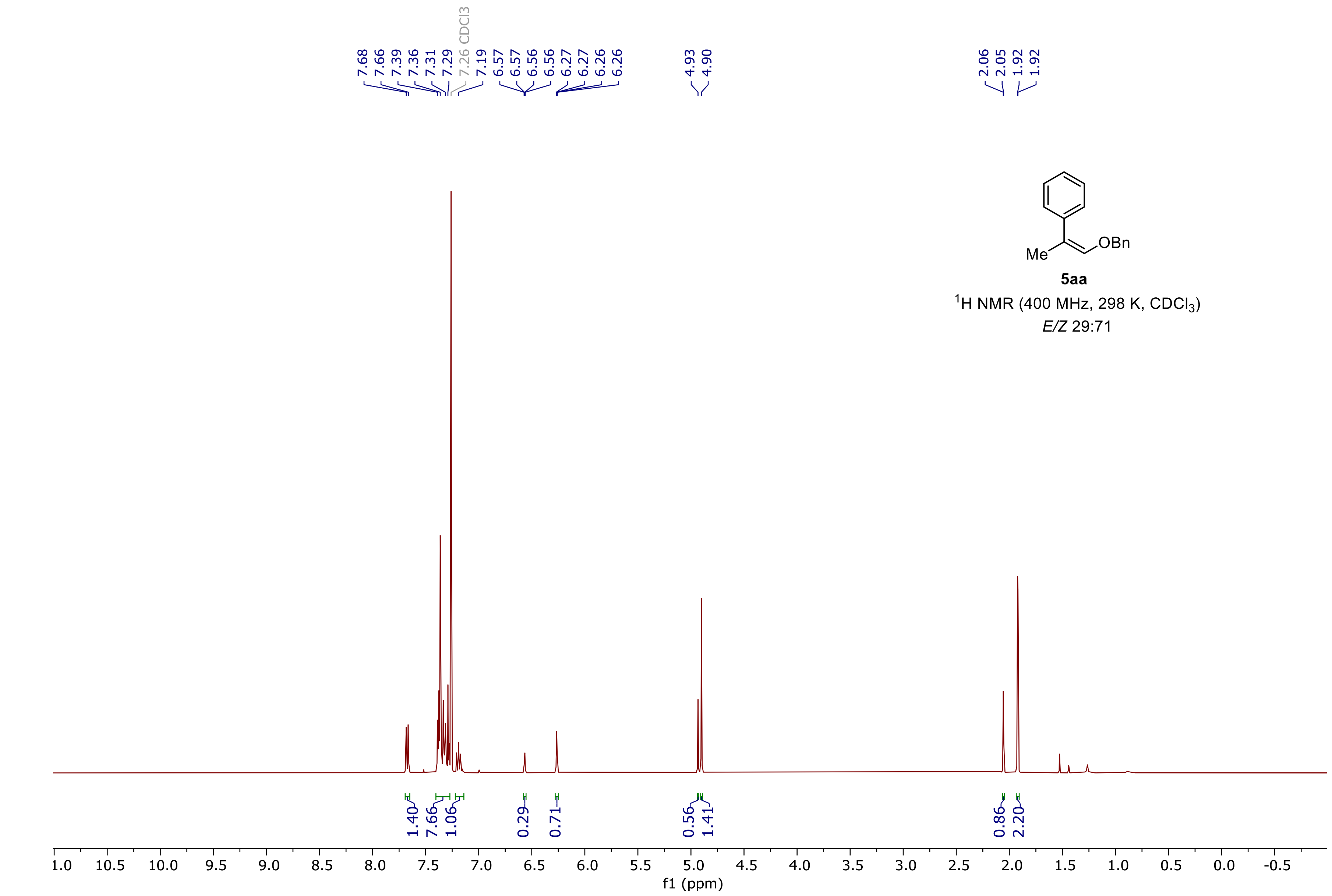

5aa

^{1}H NMR (400 MHz, 298 K, $CDCl_3$)

E/Z 29:71

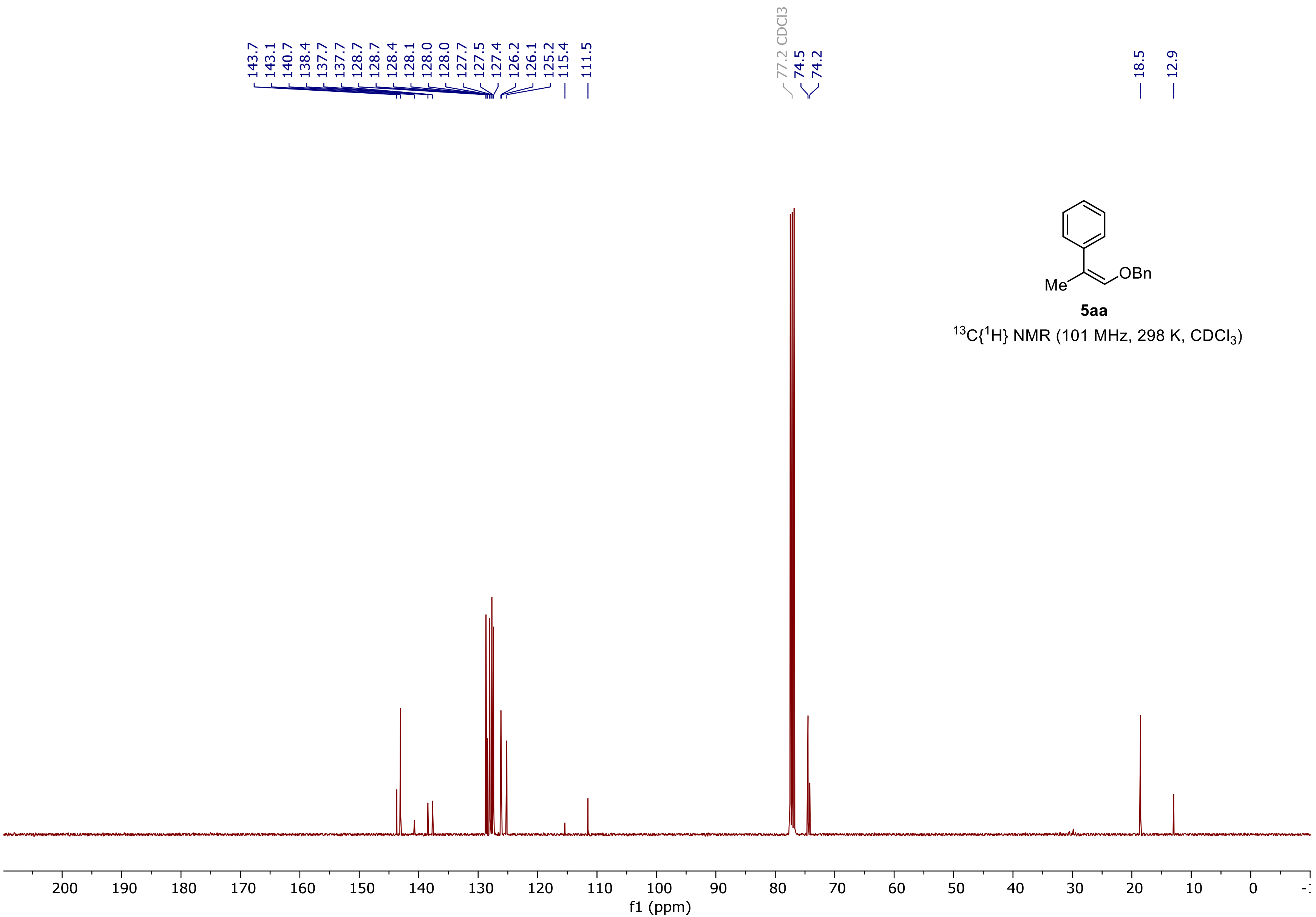

5aa

$^{13}C\{^{1}H\}$ NMR (101 MHz, 298 K, $CDCl_3$)

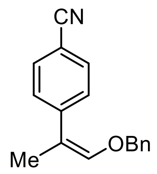

5ab

^{1}H NMR (400 MHz, 298 K, $CDCl_3$)

E/Z 44:56

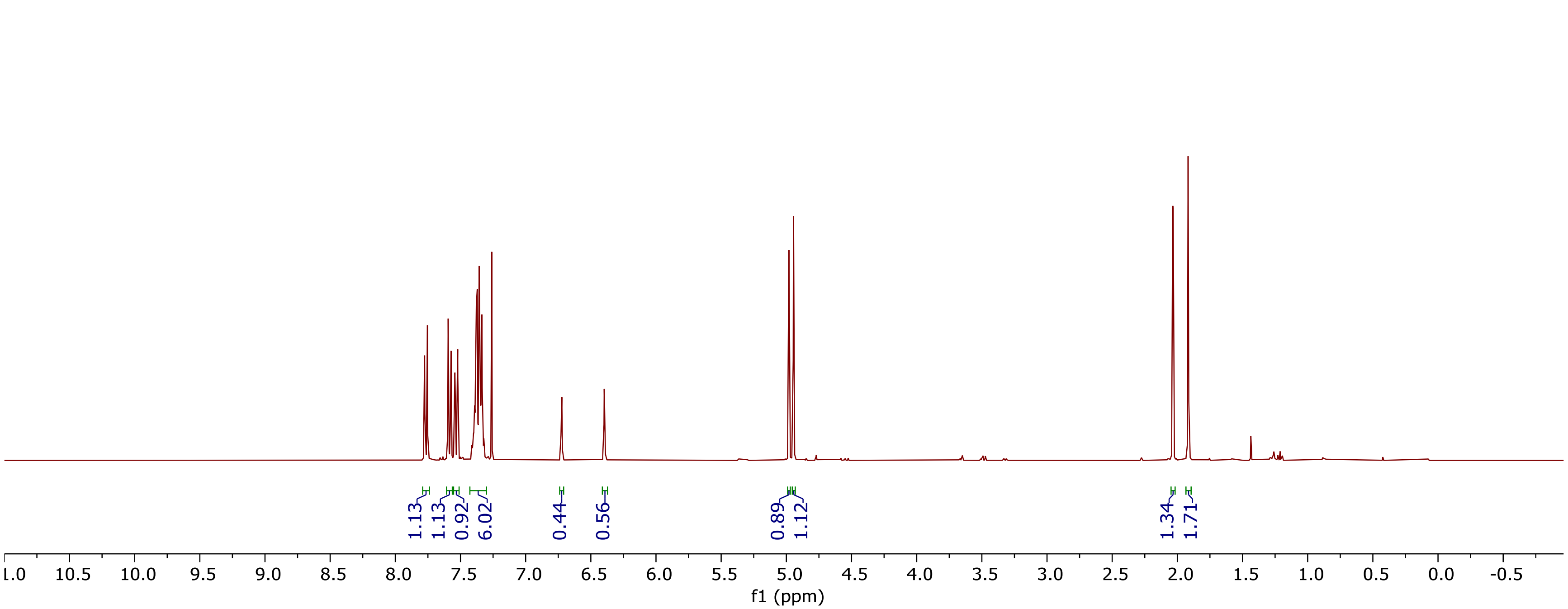

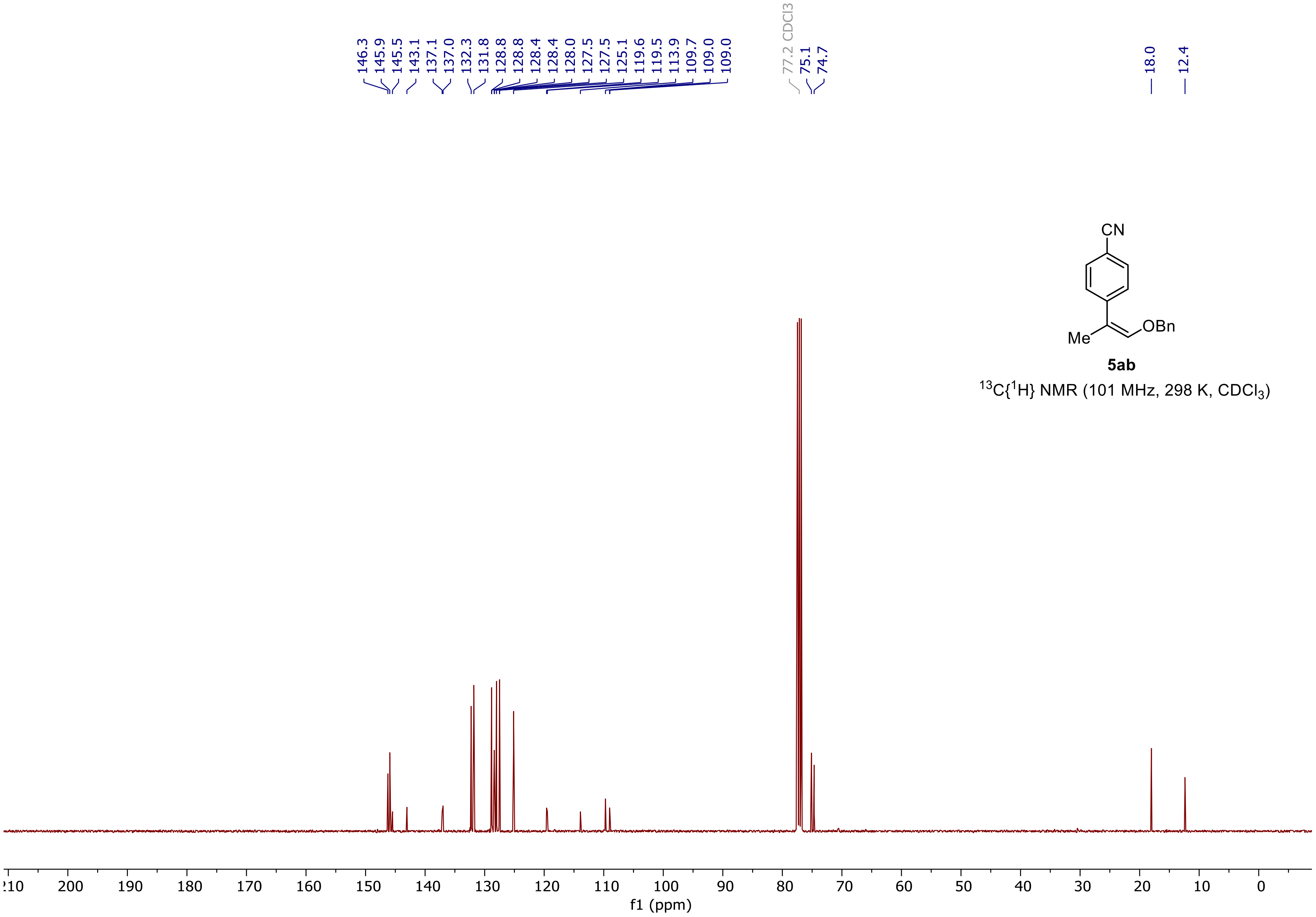

5ab

$^{13}C\{^{1}H\}$ NMR (101 MHz, 298 K, $CDCl_3$)

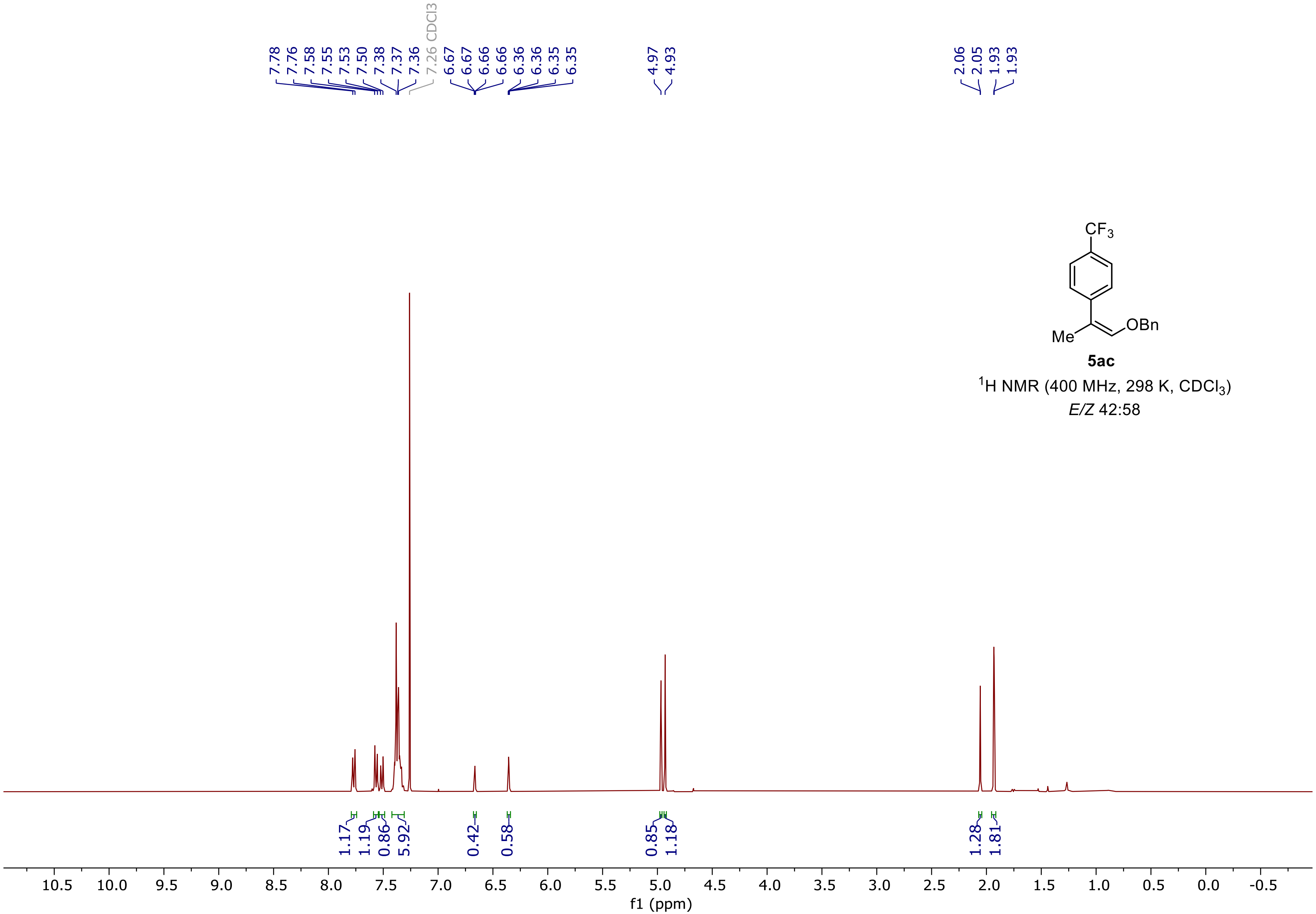

5ac

^{1}H NMR (400 MHz, 298 K, $CDCl_3$)

E/Z 42:58

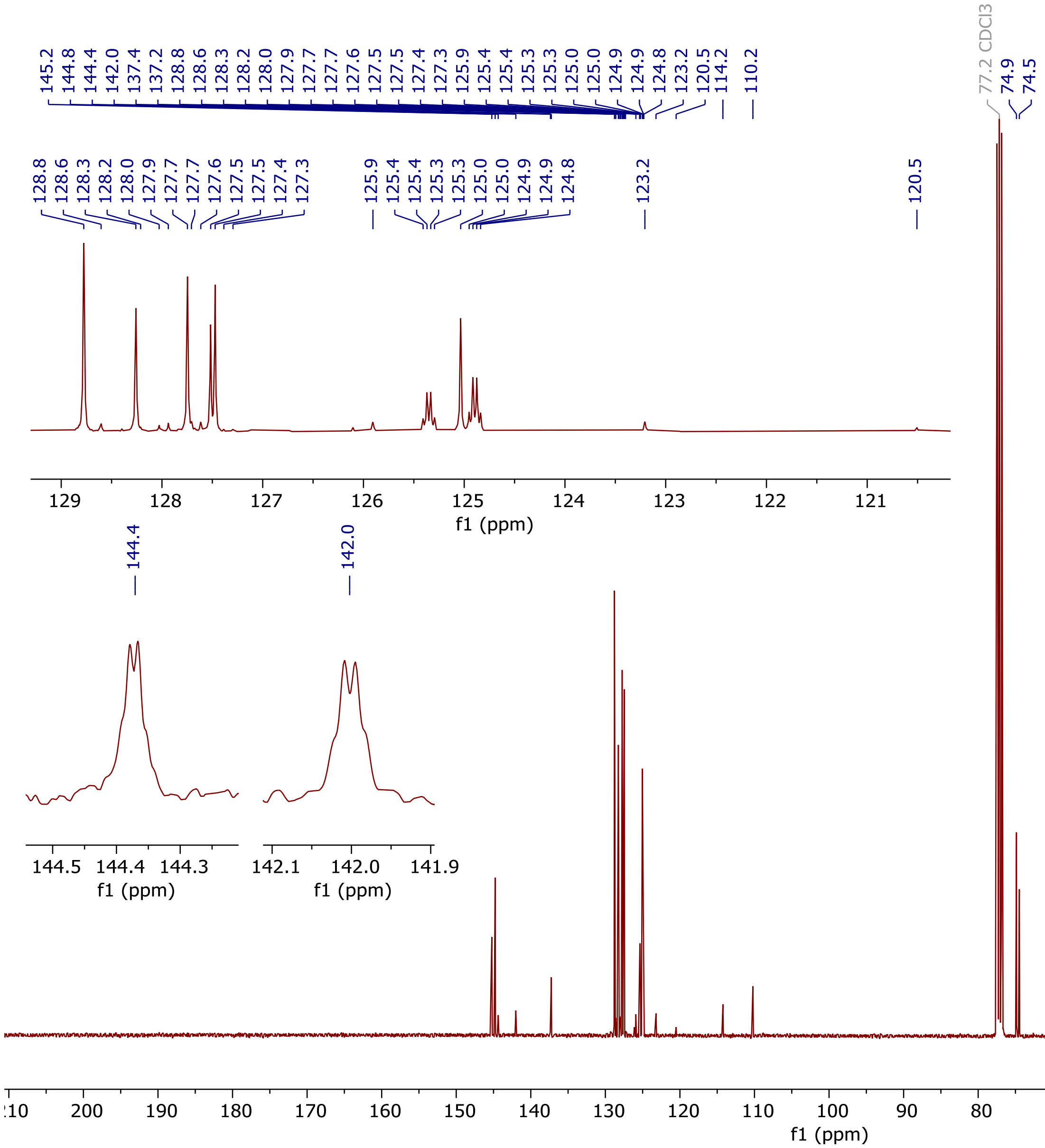

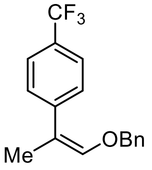

5ac

$^{13}C\{^{1}H\}$ NMR (101 MHz, 298 K, $CDCl_3$)

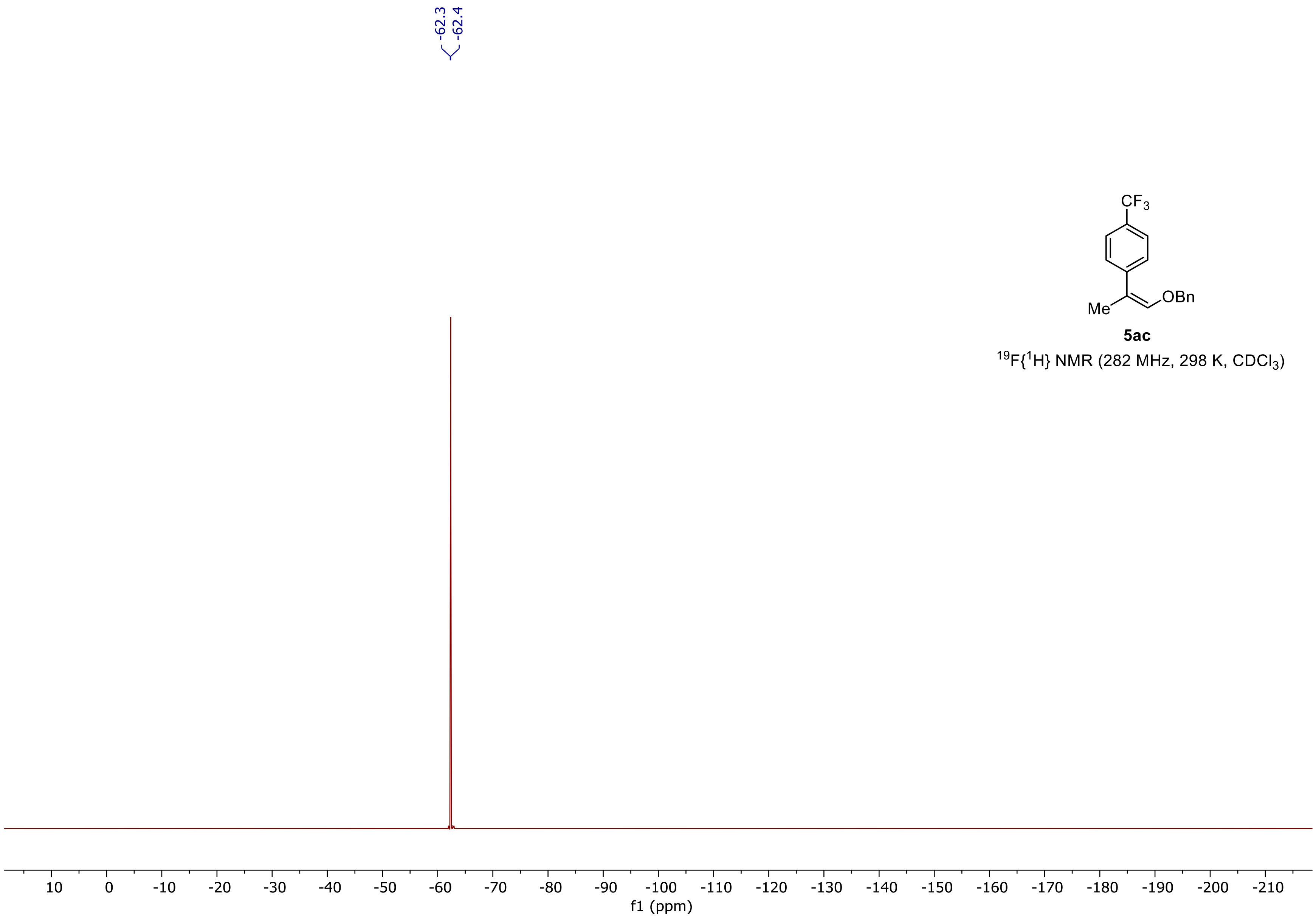

5ac

$^{19}F\{^1H\}$ NMR (282 MHz, 298 K, $CDCl_3$)

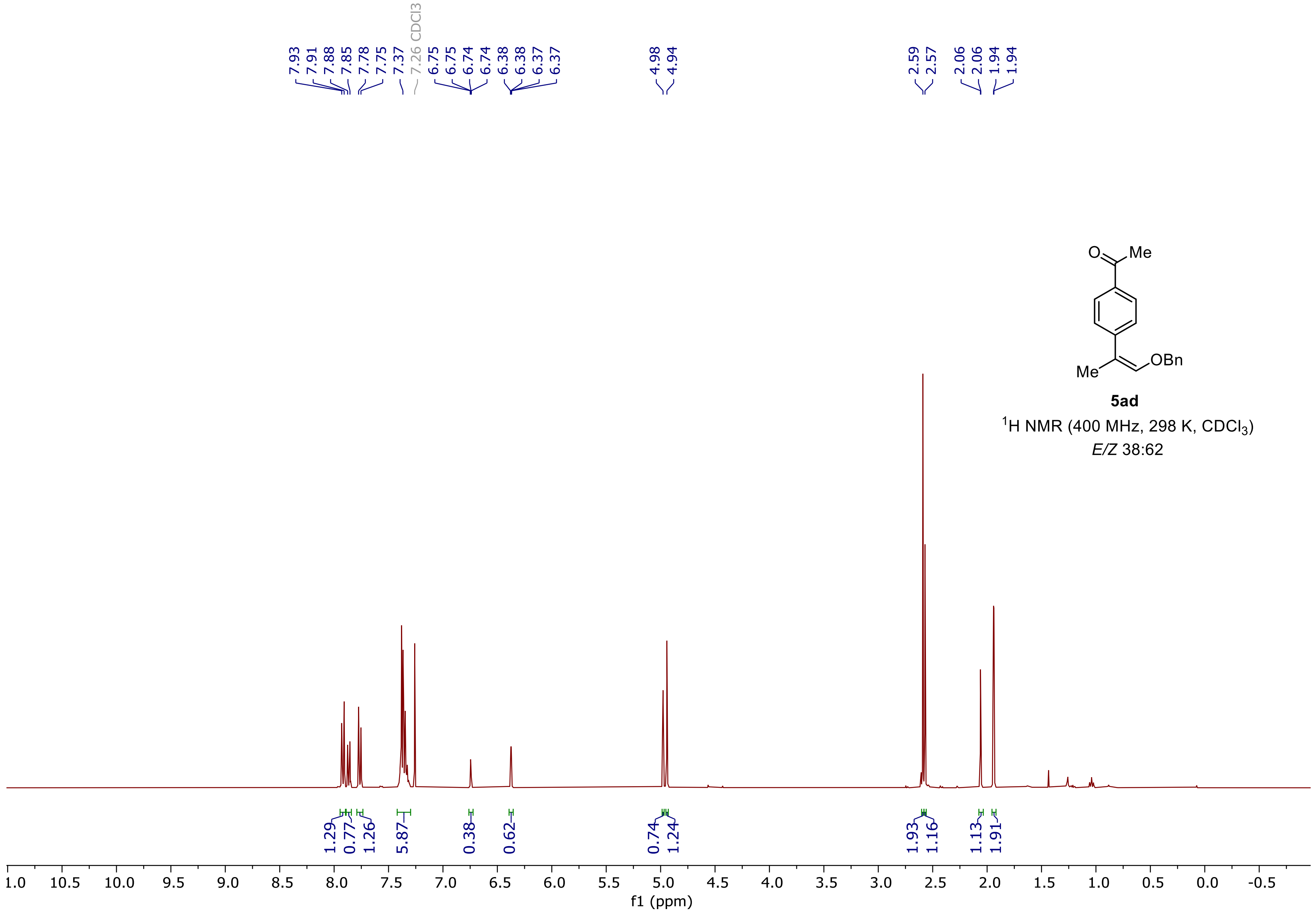

5ad

^{1}H NMR (400 MHz, 298 K, $CDCl_3$)

E/Z 38:62

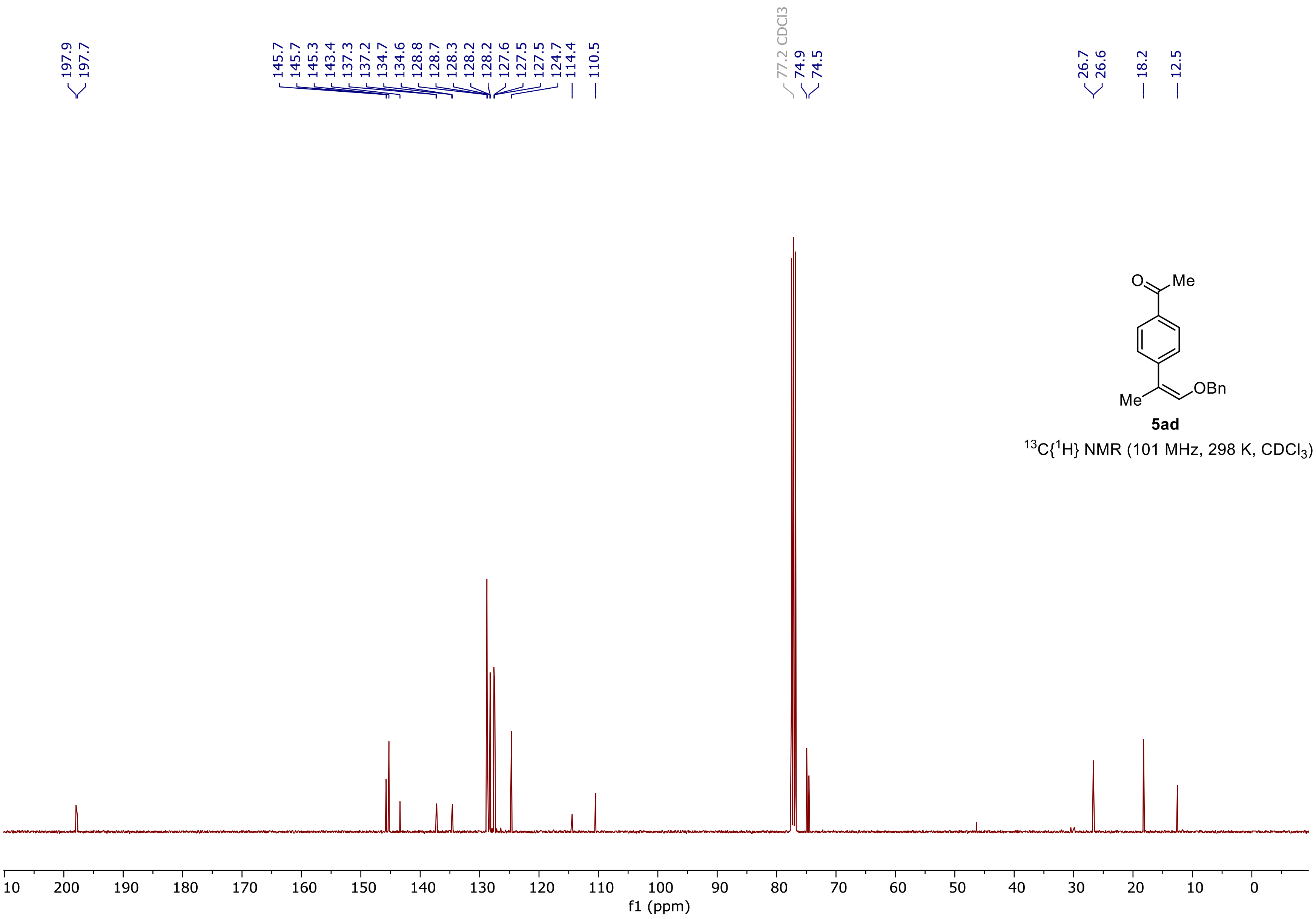

5ad

$^{13}C\{^{1}H\}$ NMR (101 MHz, 298 K, $CDCl_3$)

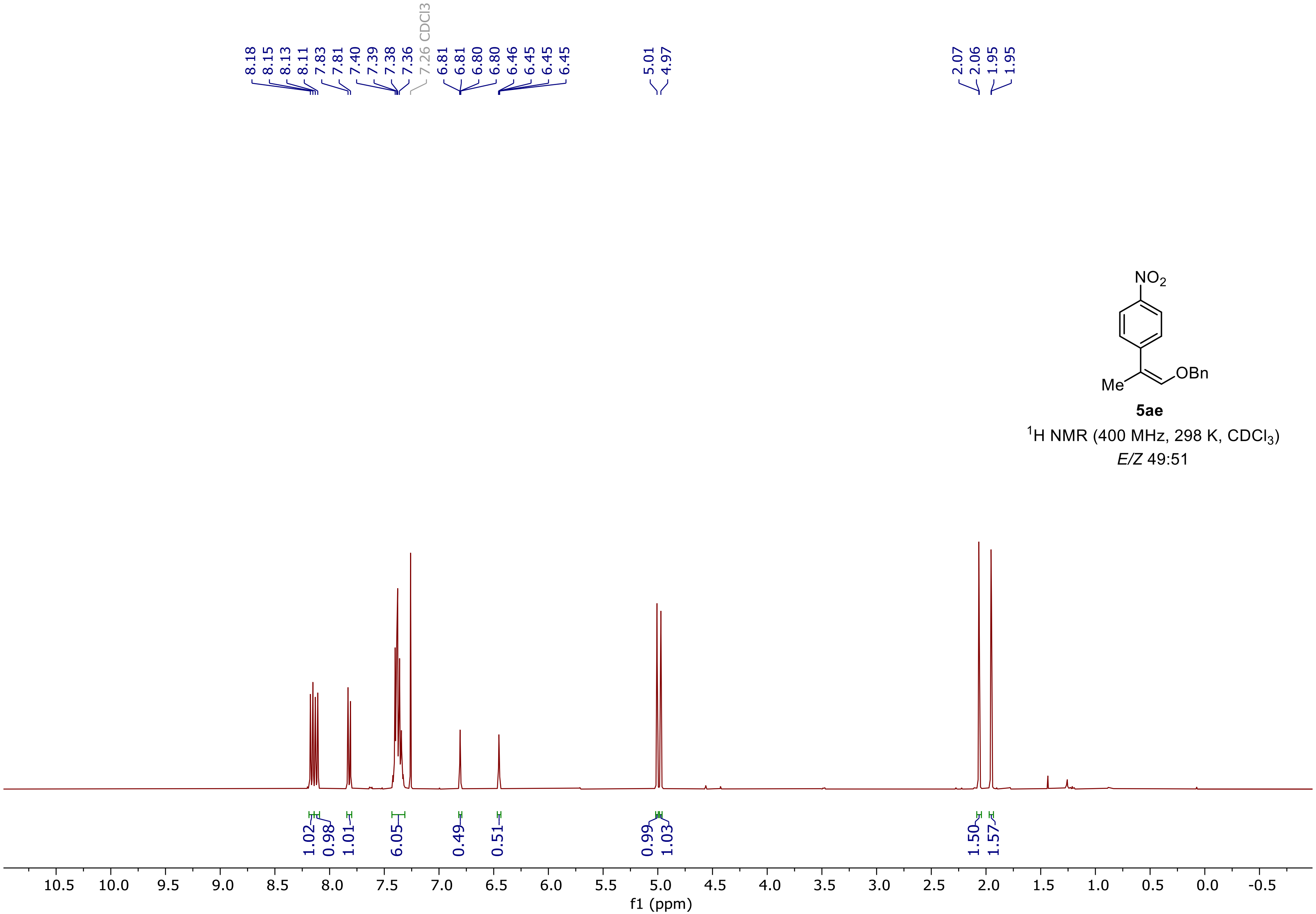

5ae

^{1}H NMR (400 MHz, 298 K, $CDCl_3$)

E/Z 49:51

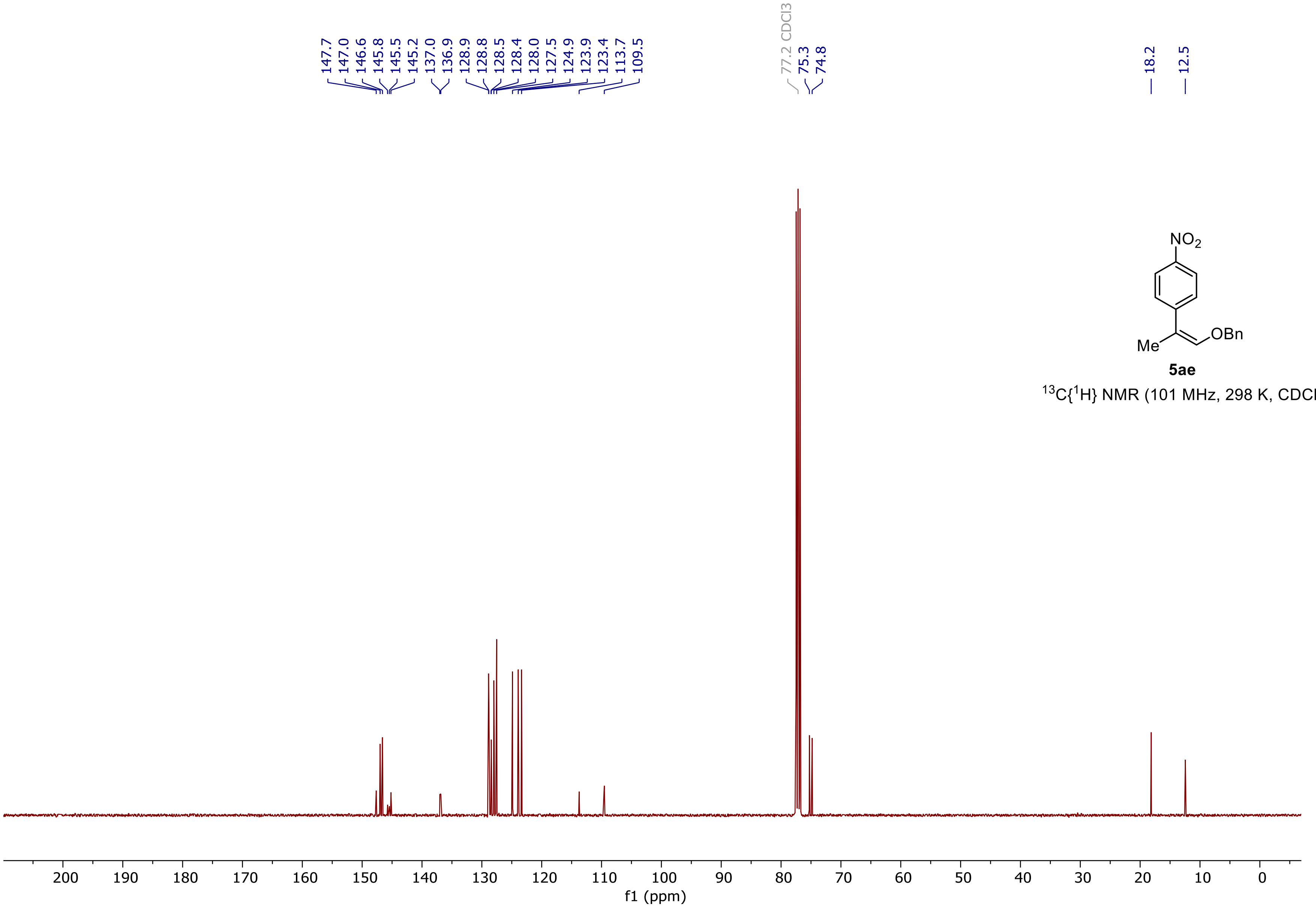

5ae

$^{13}C\{^{1}H\}$ NMR (101 MHz, 298 K, $CDCl_3$)

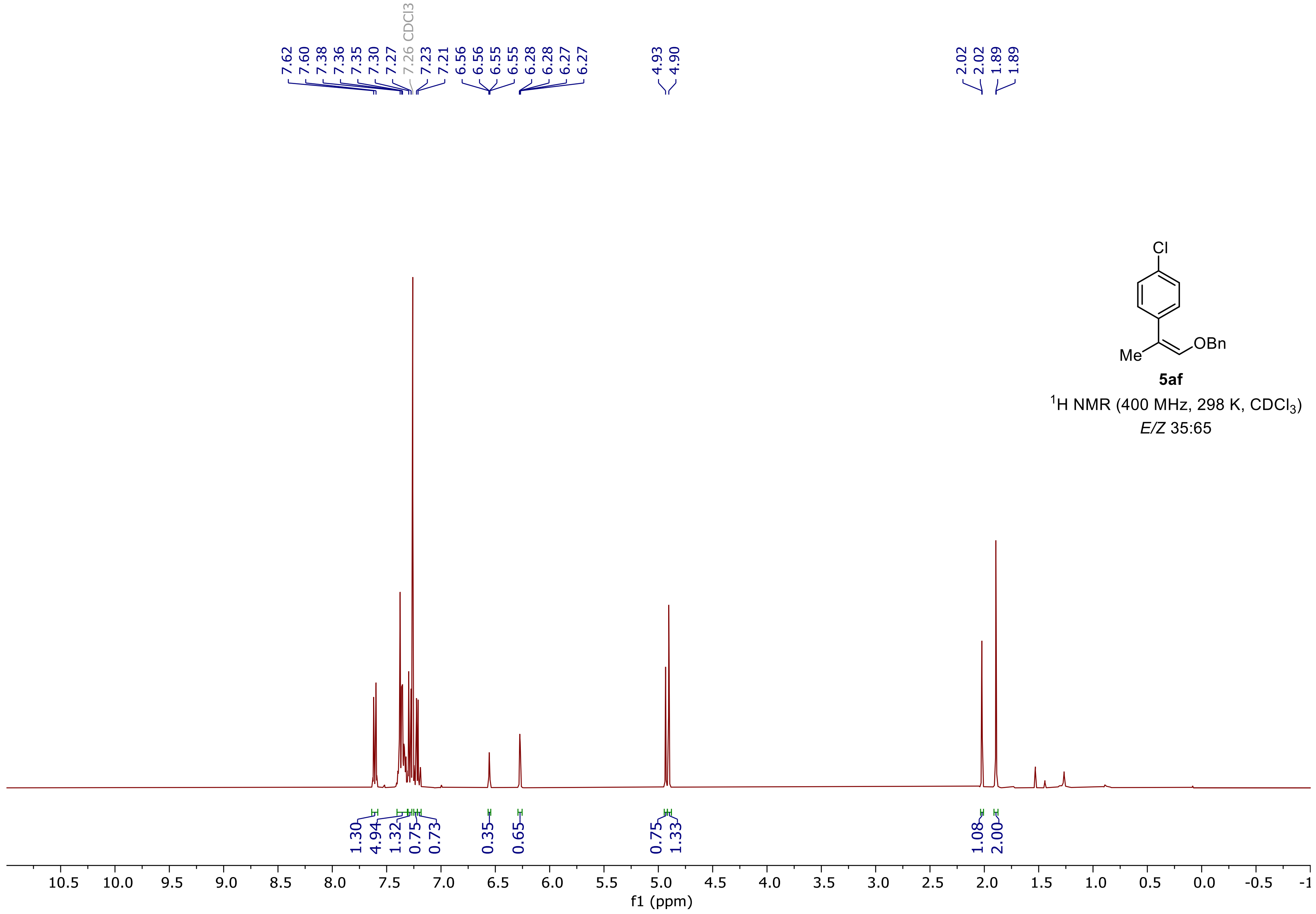

5af

^{1}H NMR (400 MHz, 298 K, $CDCl_3$)

E/Z 35:65

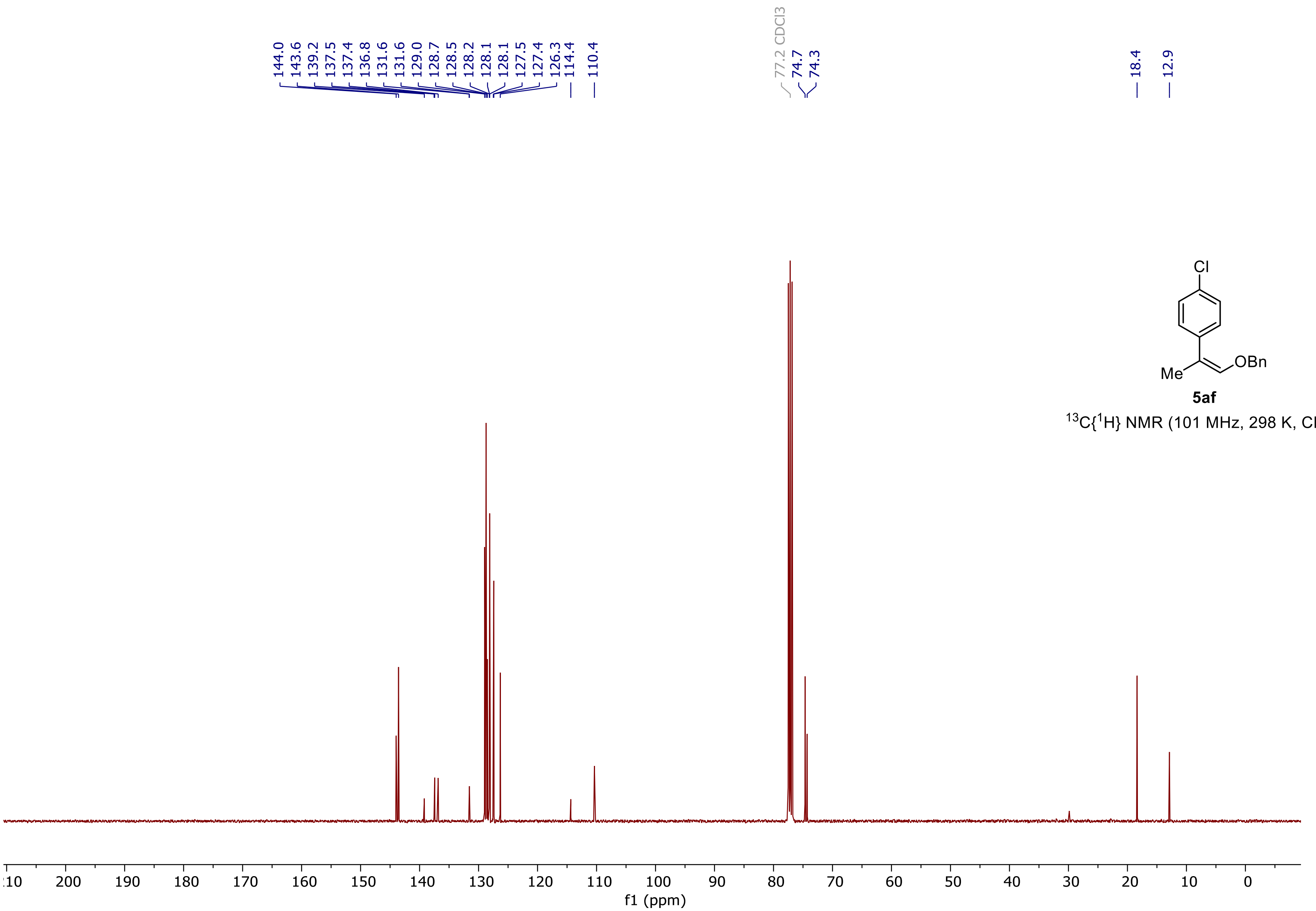

5af

$^{13}C\{^{1}H\}$ NMR (101 MHz, 298 K, $CDCl_3$)

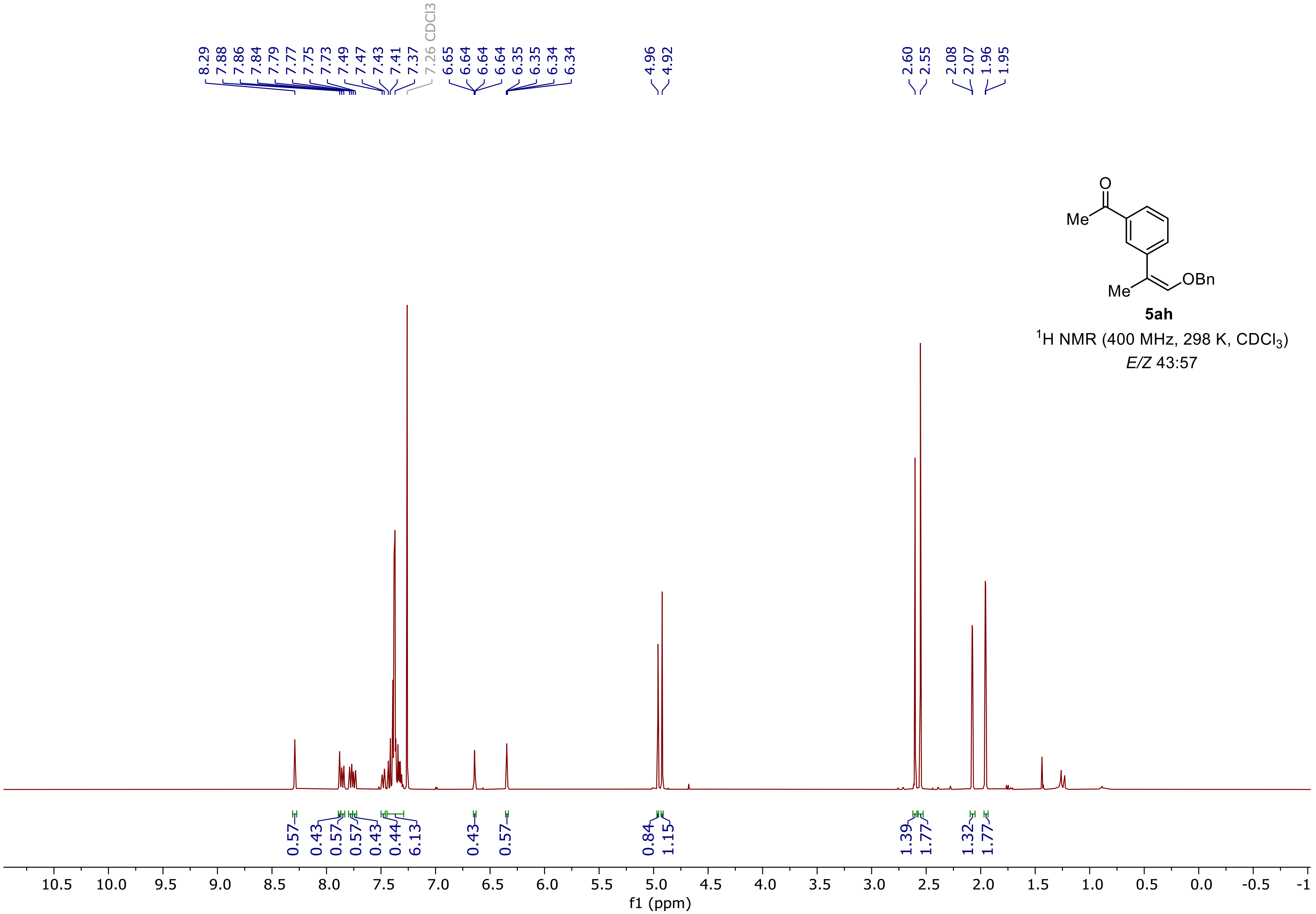

5ah

^{1}H NMR (400 MHz, 298 K, $CDCl_3$)

E/Z 43:57

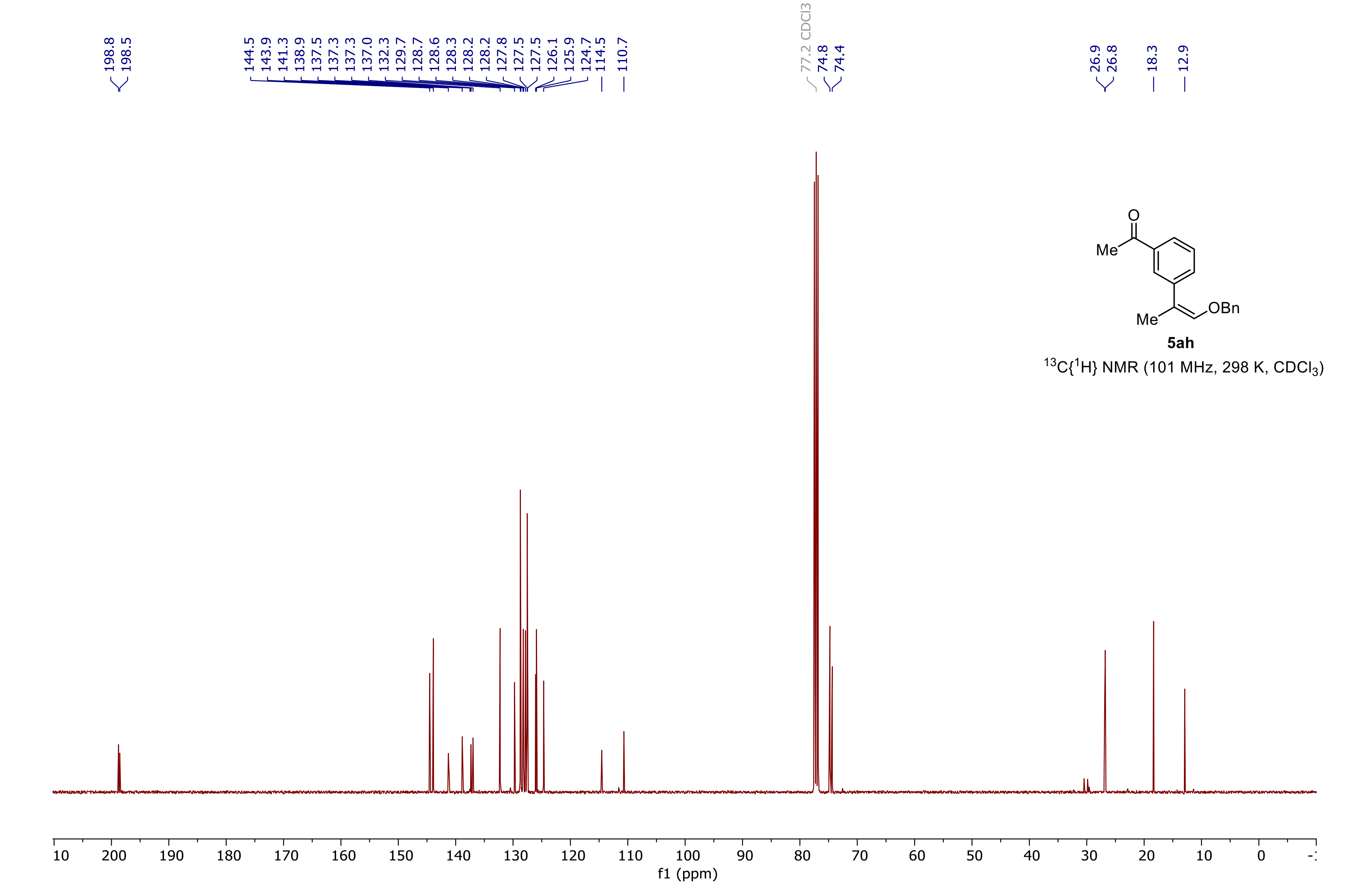

5ah

$^{13}C\{^{1}H\}$ NMR (101 MHz, 298 K, $CDCl_3$)

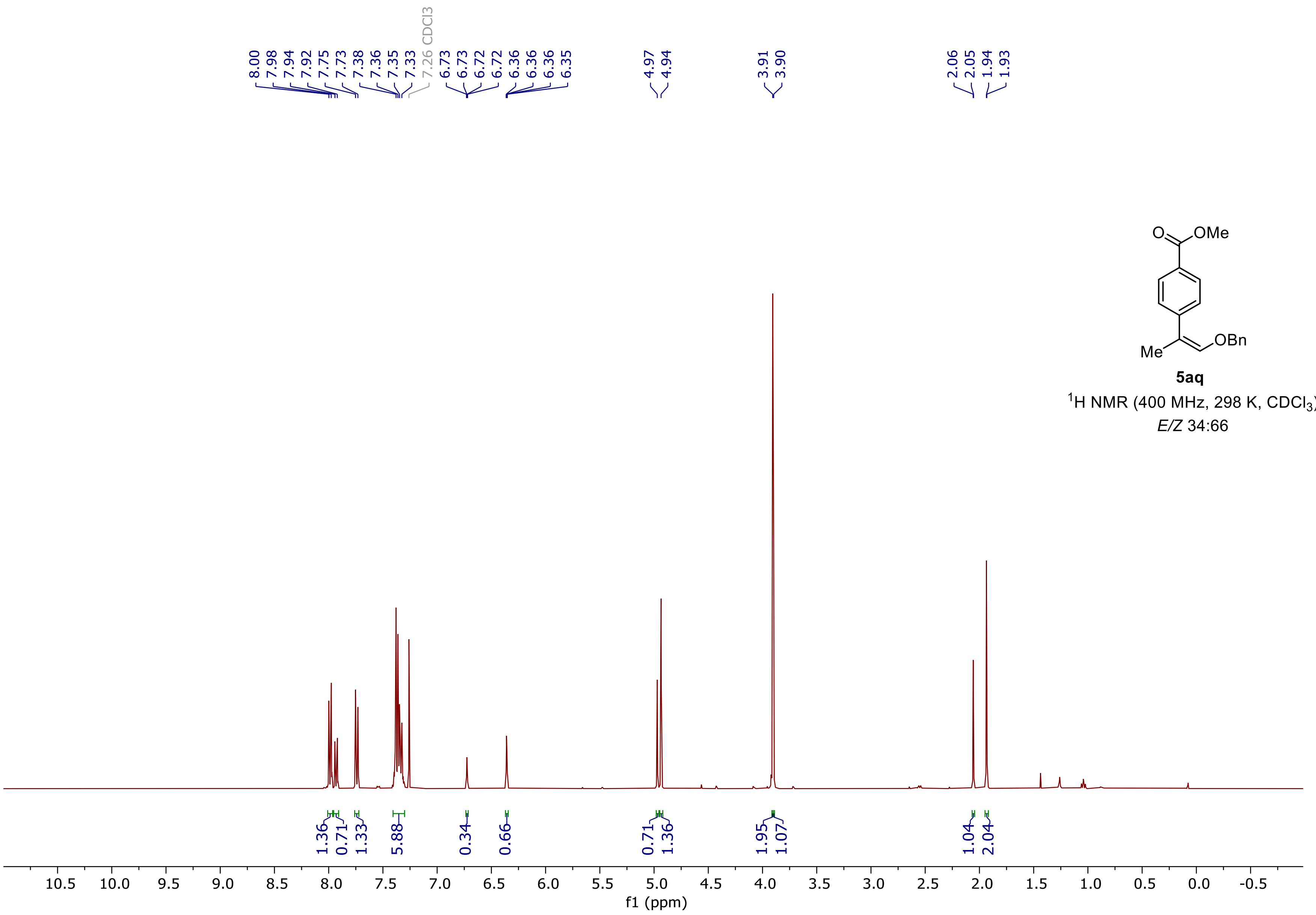

5aq

^{1}H NMR (400 MHz, 298 K, $CDCl_3$)

E/Z 34:66

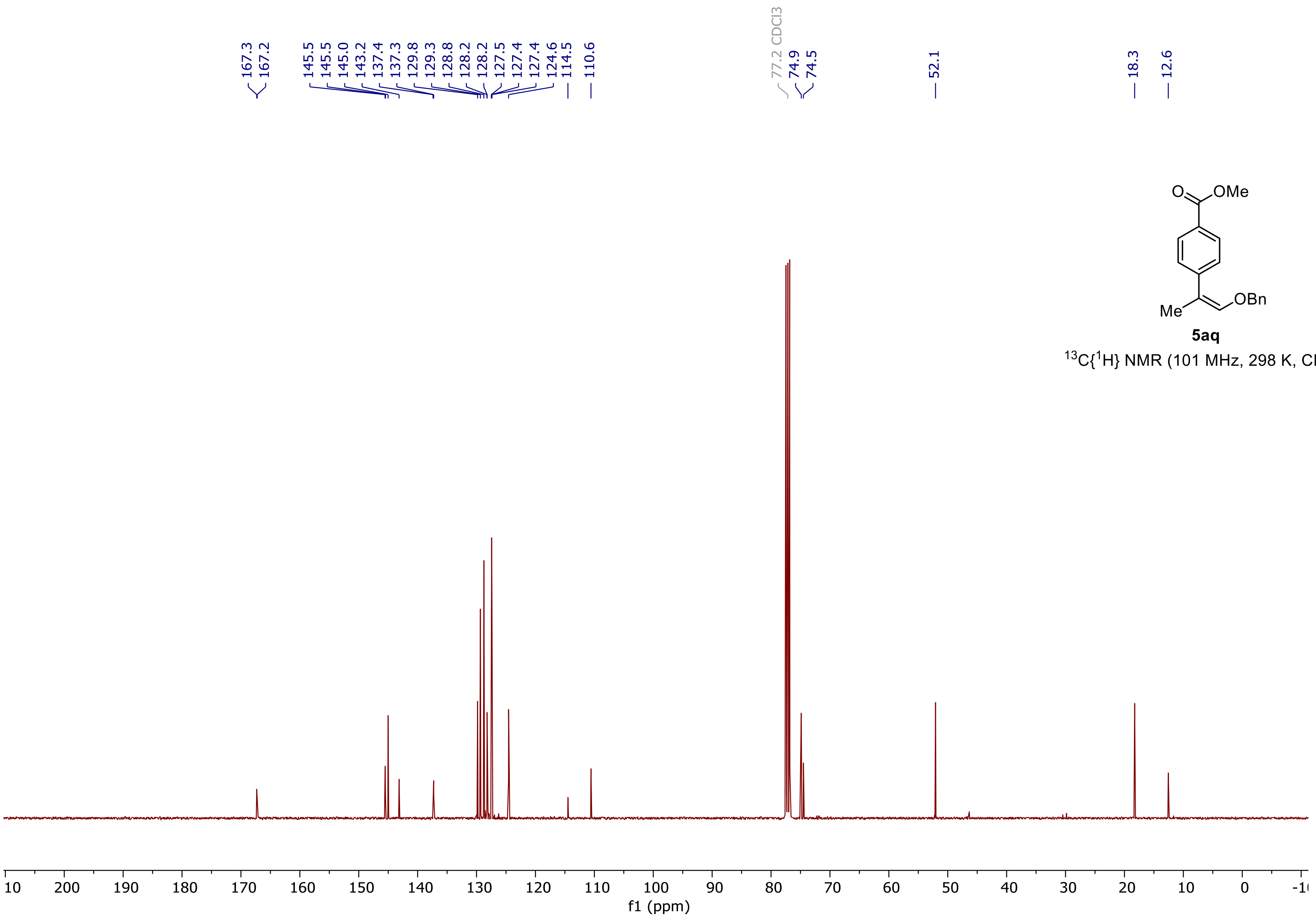

5aq

$^{13}C\{^{1}H\}$ NMR (101 MHz, 298 K, $CDCl_3$)

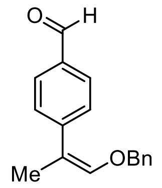

5ar

^1H NMR (400 MHz, 298 K, $CDCl_3$)

E/Z 43:57

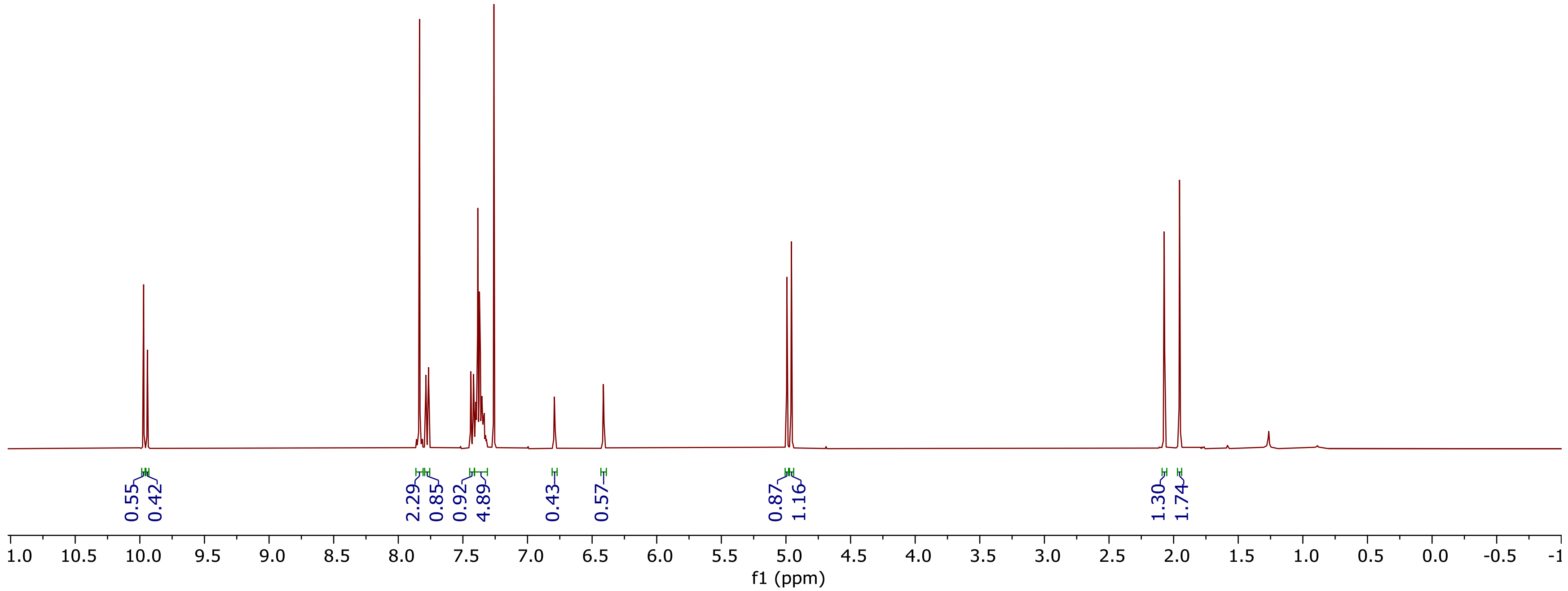

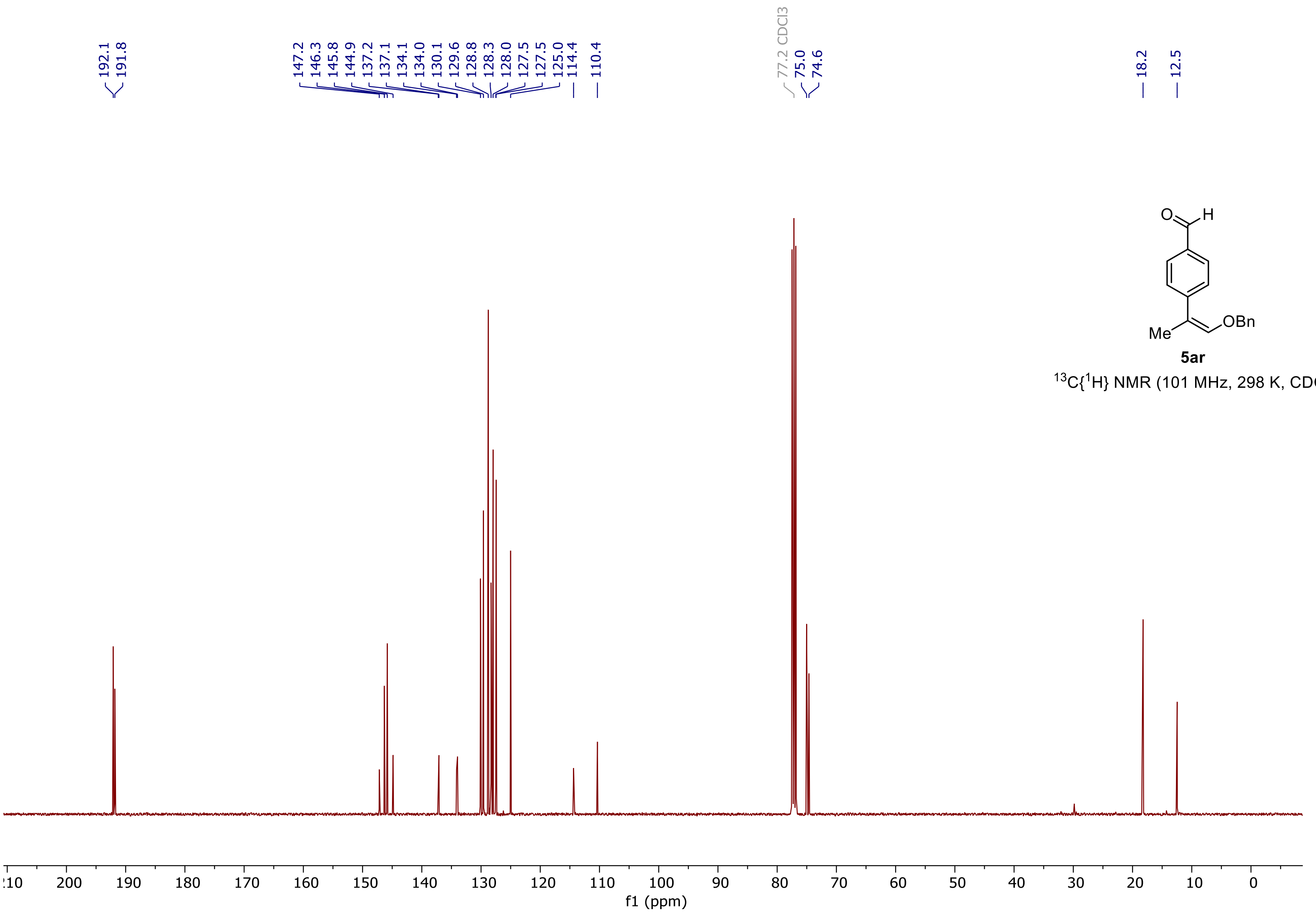

5ar

$^{13}C\{^{1}H\}$ NMR (101 MHz, 298 K, $CDCl_3$)

5as

^{1}H NMR (400 MHz, 298 K, $CDCl_3$)

E/Z 37:63

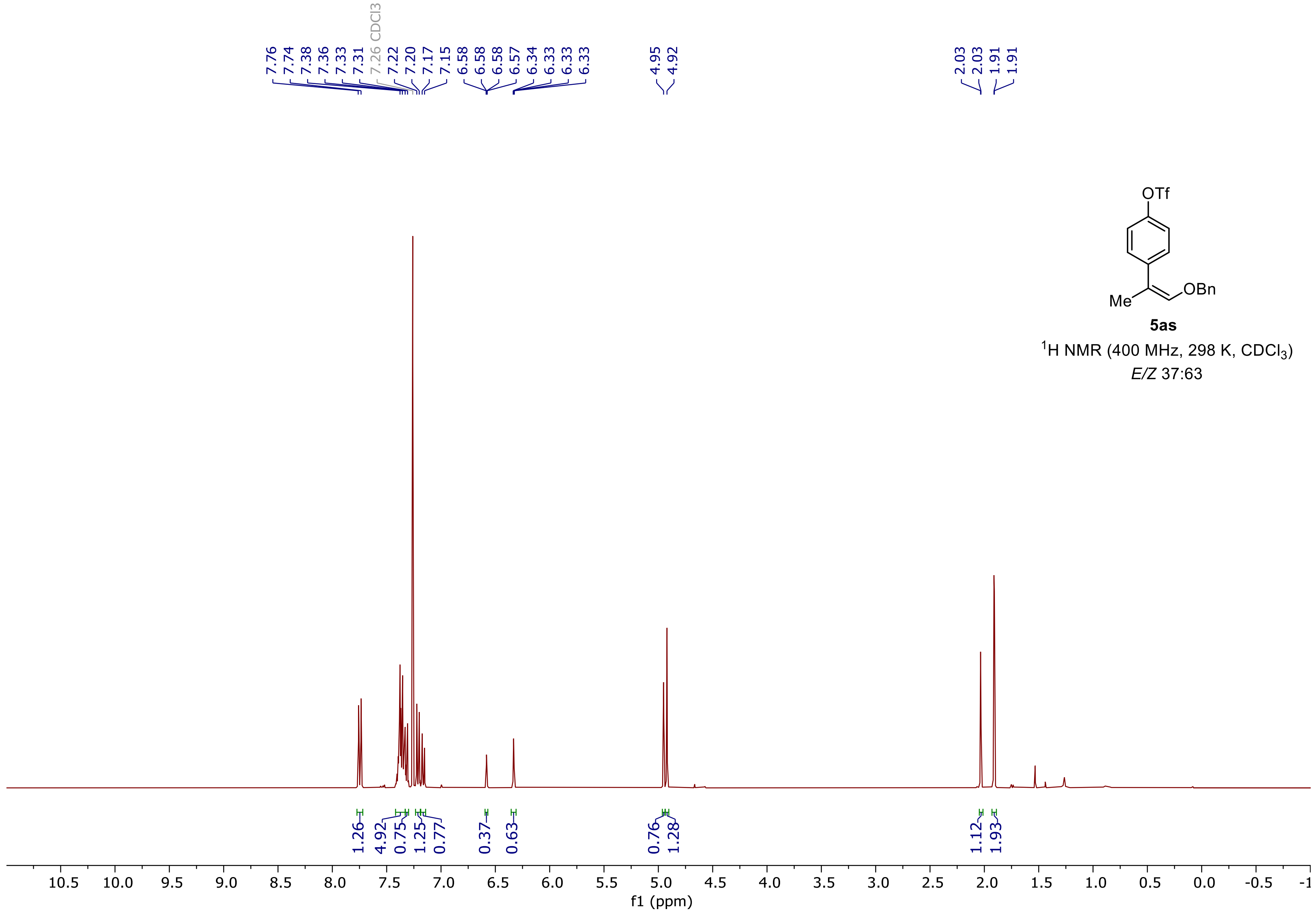

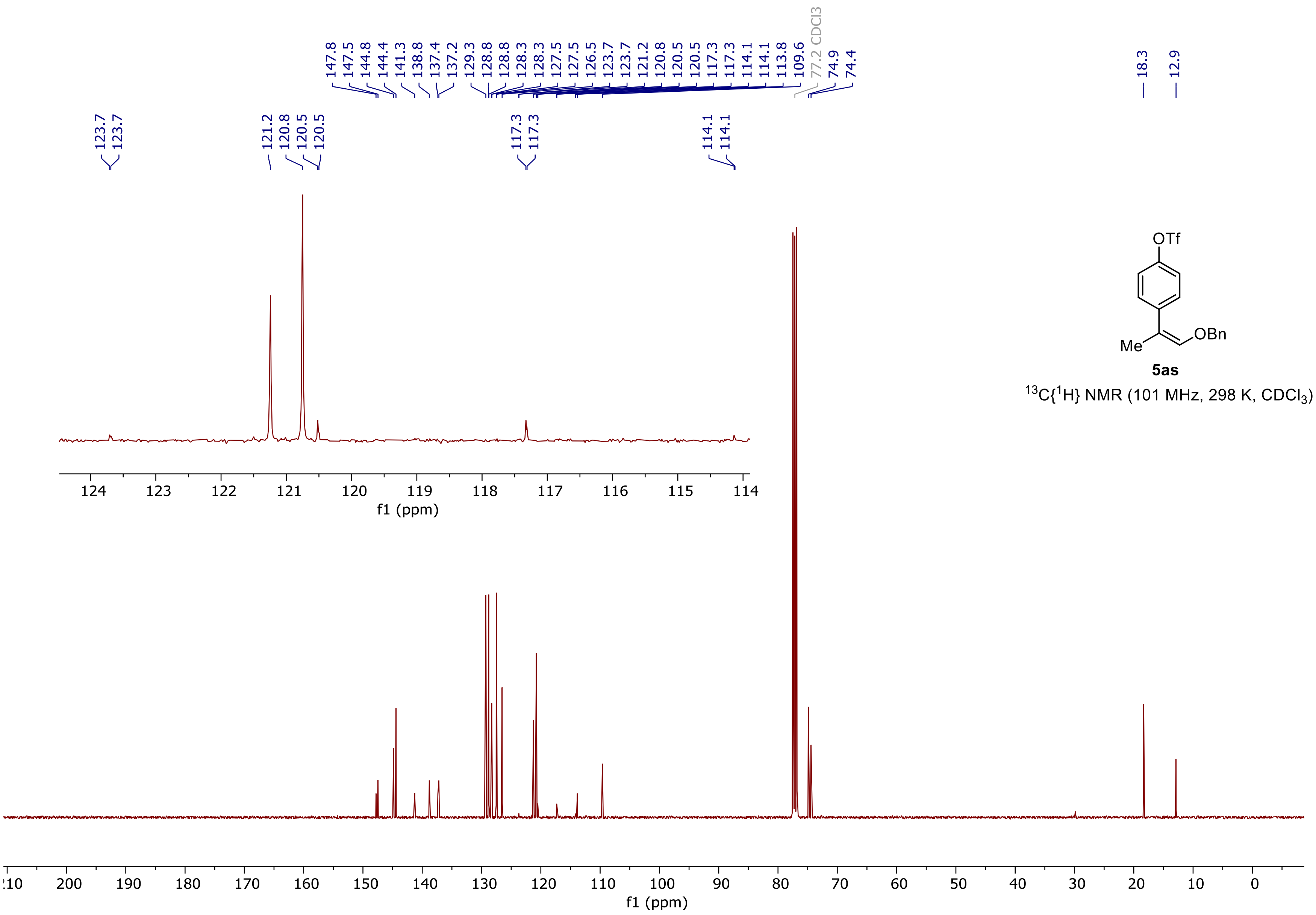

5as

$^{13}C\{^{1}H\}$ NMR (101 MHz, 298 K, $CDCl_3$)

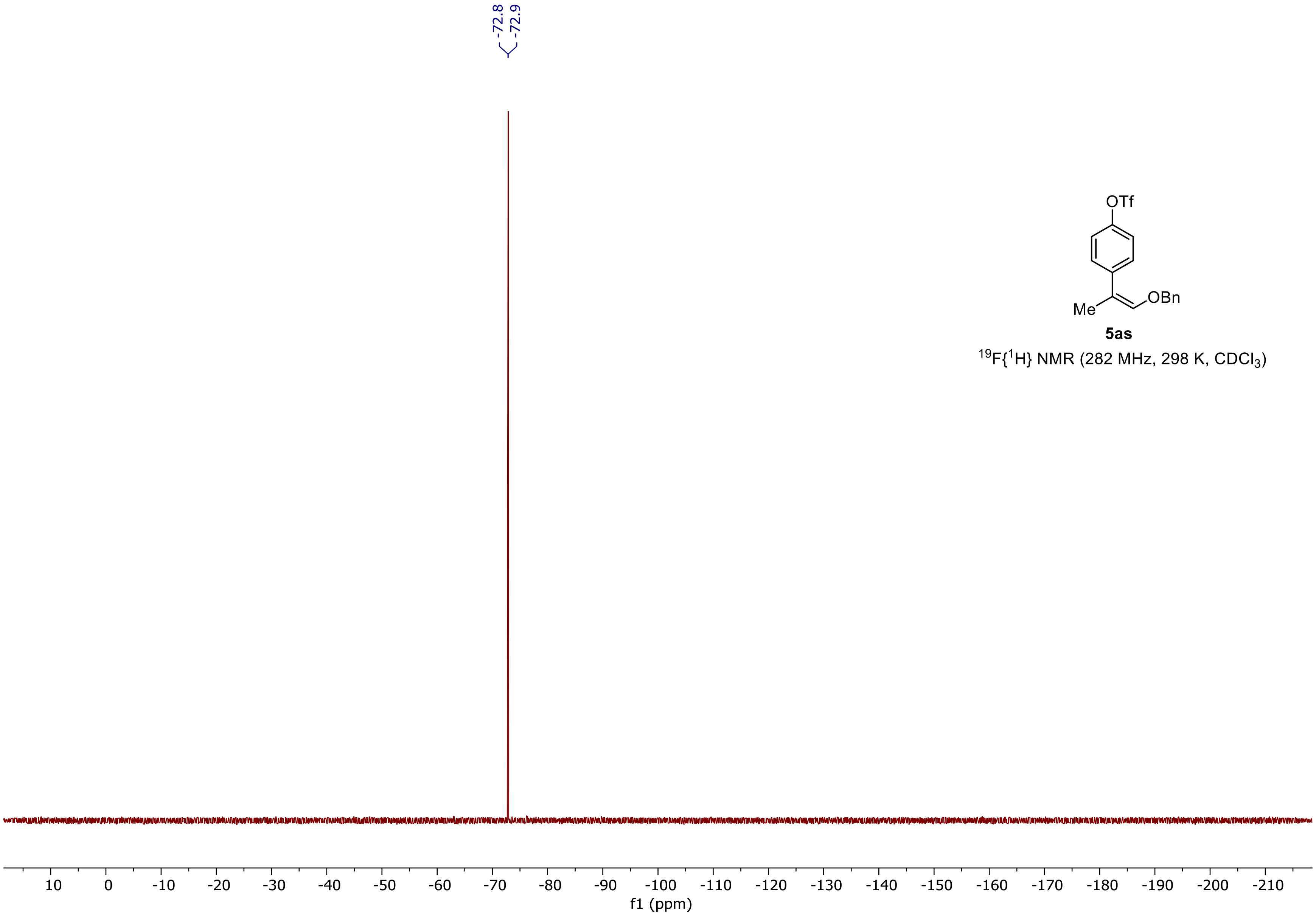

5as

$^{19}F\{^{1}H\}$ NMR (282 MHz, 298 K, $CDCl_3$)

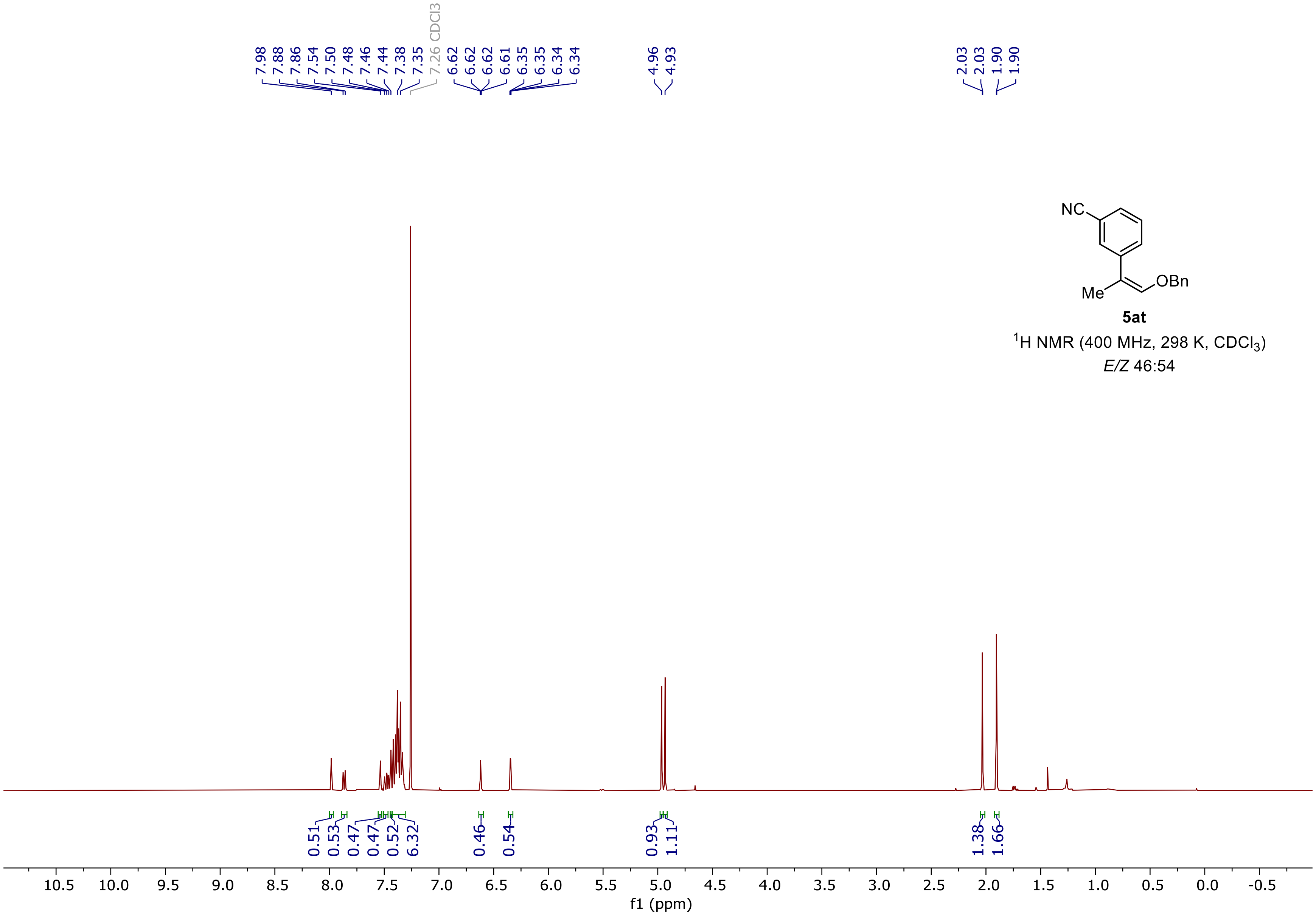

5at

^{1}H NMR (400 MHz, 298 K, $CDCl_3$)

E/Z 46:54

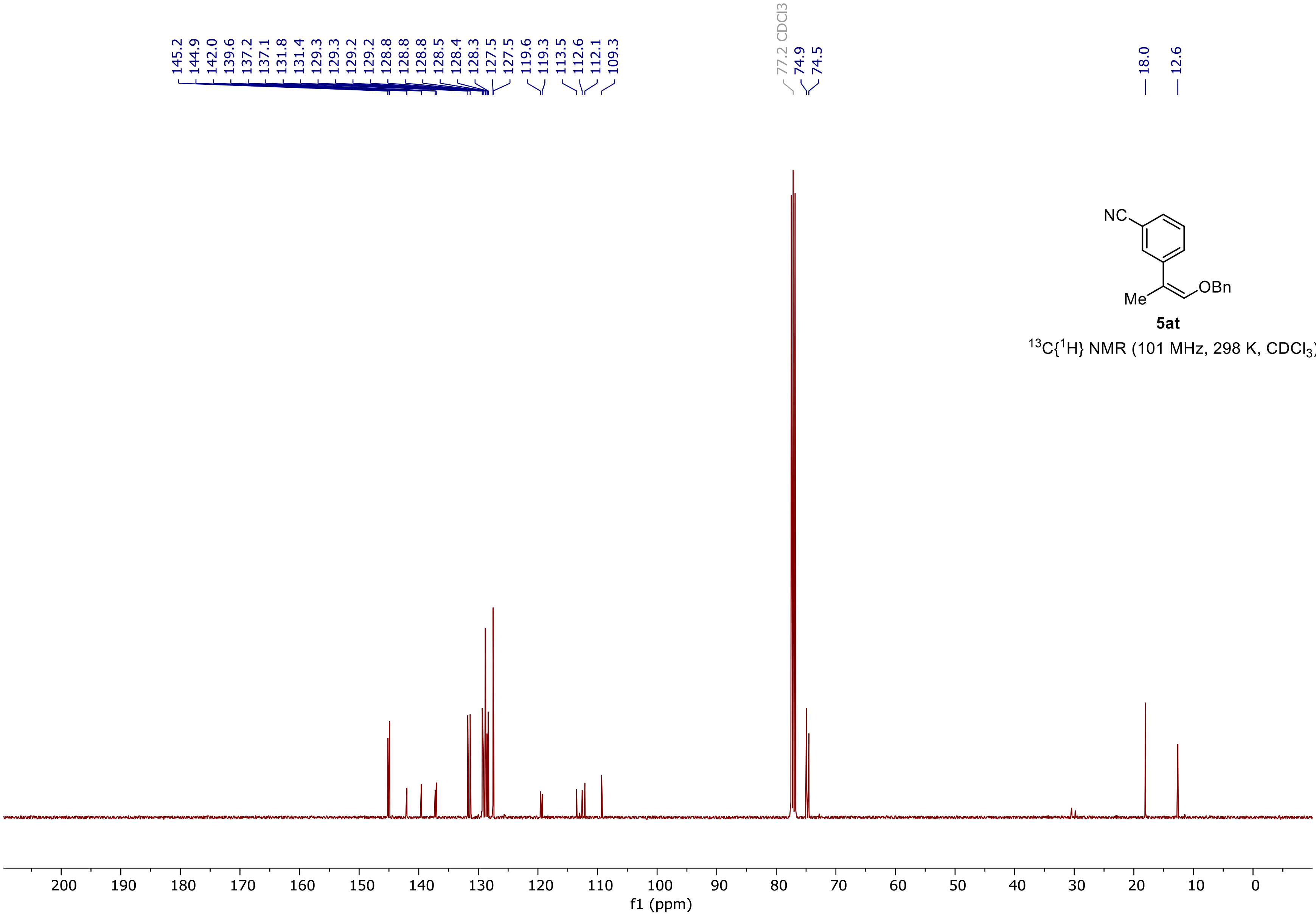

5at

$^{13}C\{^{1}H\}$ NMR (101 MHz, 298 K, $CDCl_3$)

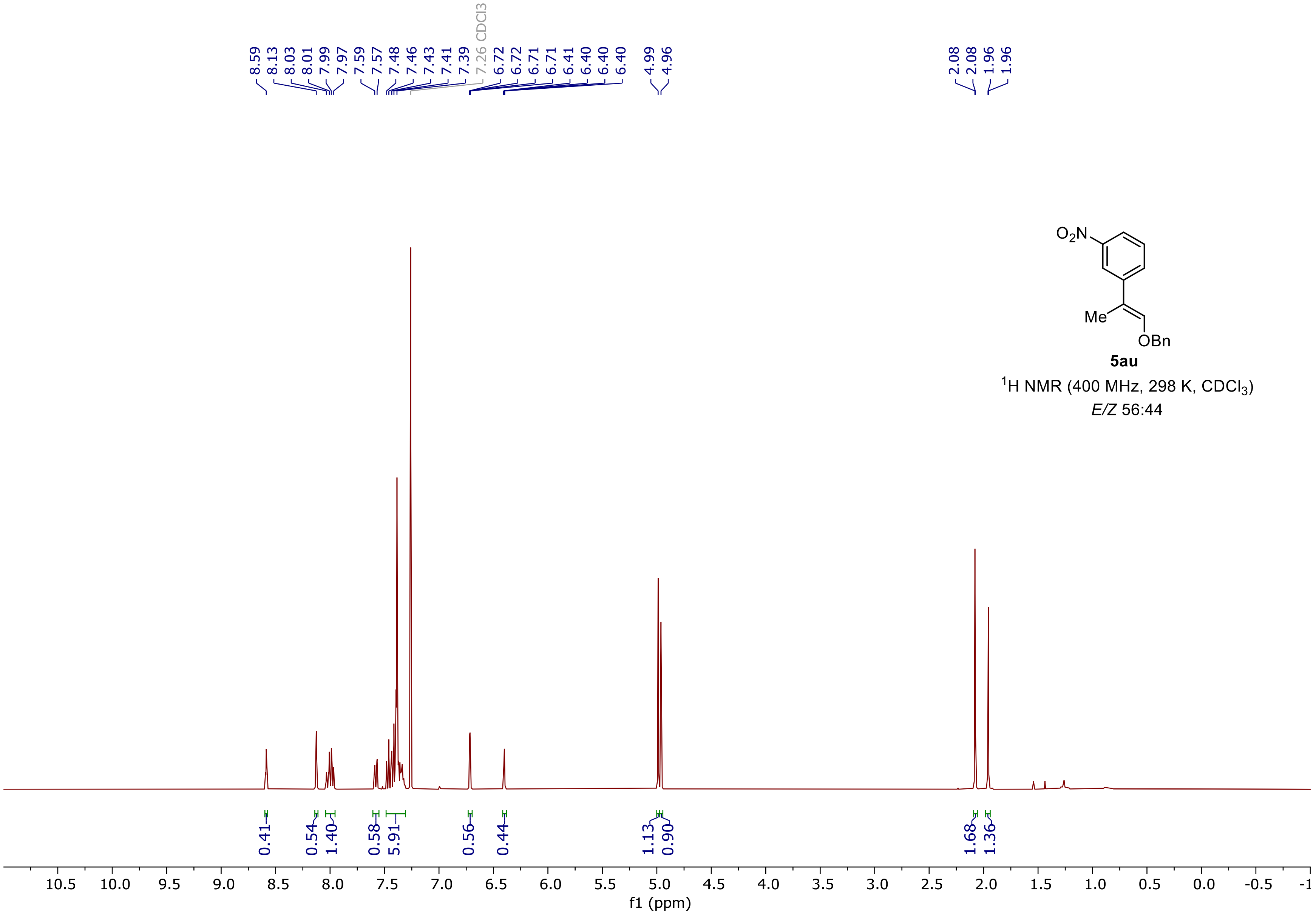
8.59
8.13
8.03
8.01
7.99
7.97
7.59
7.57
7.48
7.46
7.43
7.41
7.39
7.26 CDCl3
6.72
6.72
6.71
6.71
6.41
6.40
6.40
6.40
4.99
4.96
2.08
2.08
1.96
1.96
O2N
Me
OBn
5au
1H NMR (400 MHz, 298 K, CDCl3)
E/Z 56:44
0.41
0.54
1.40
0.58
5.91
0.56
0.44
1.13
0.90
1.68
1.36
10.5
10.0
9.5
9.0
8.5
8.0
7.5
7.0
6.5
6.0
5.5
5.0
4.5
4.0
3.5
3.0
2.5
2.0
1.5
1.0
0.5
0.0
-0.5
-1
f1 (ppm)

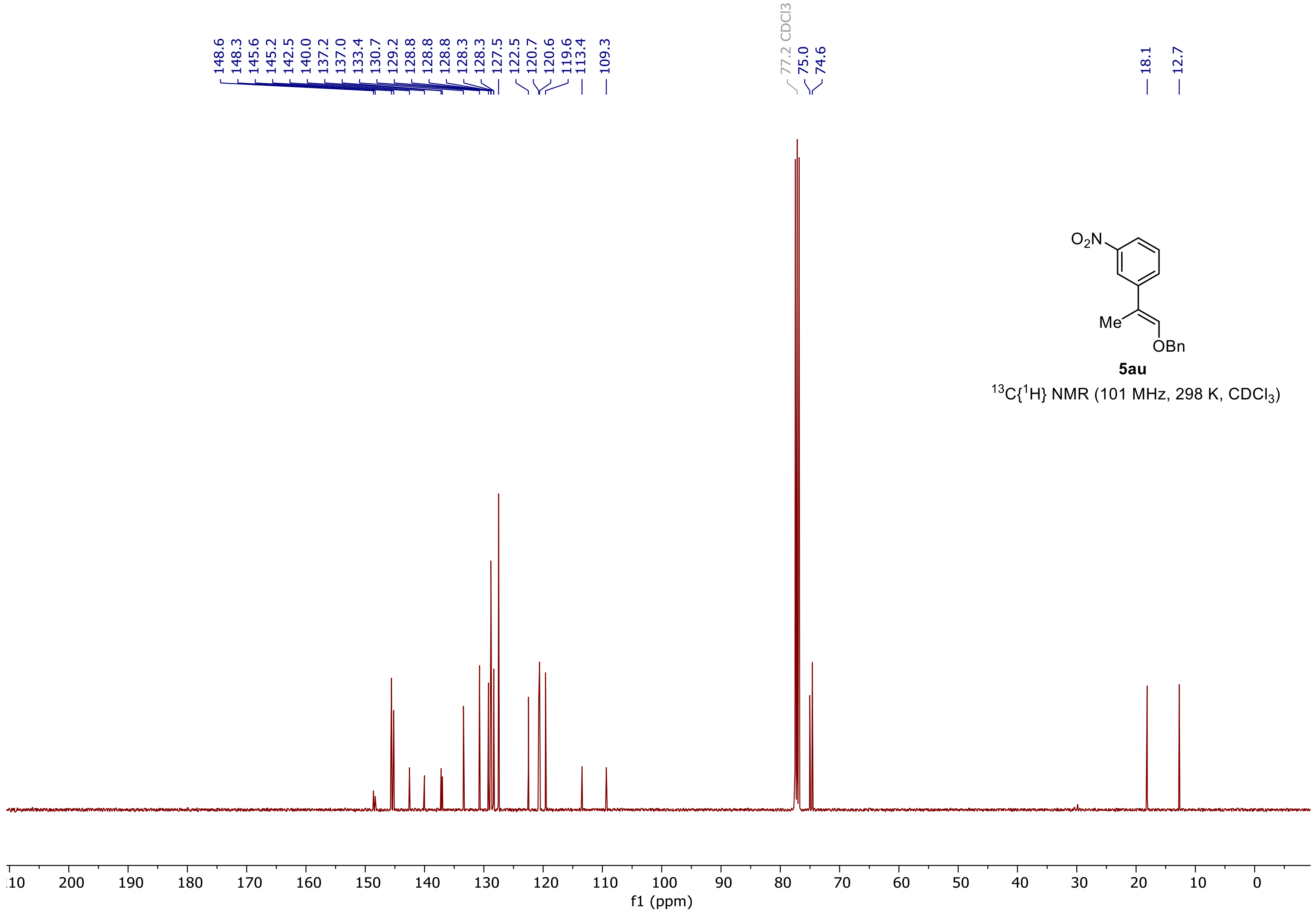

5au

$^{13}C\{^1H\}$ NMR (101 MHz, 298 K, $CDCl_3$)

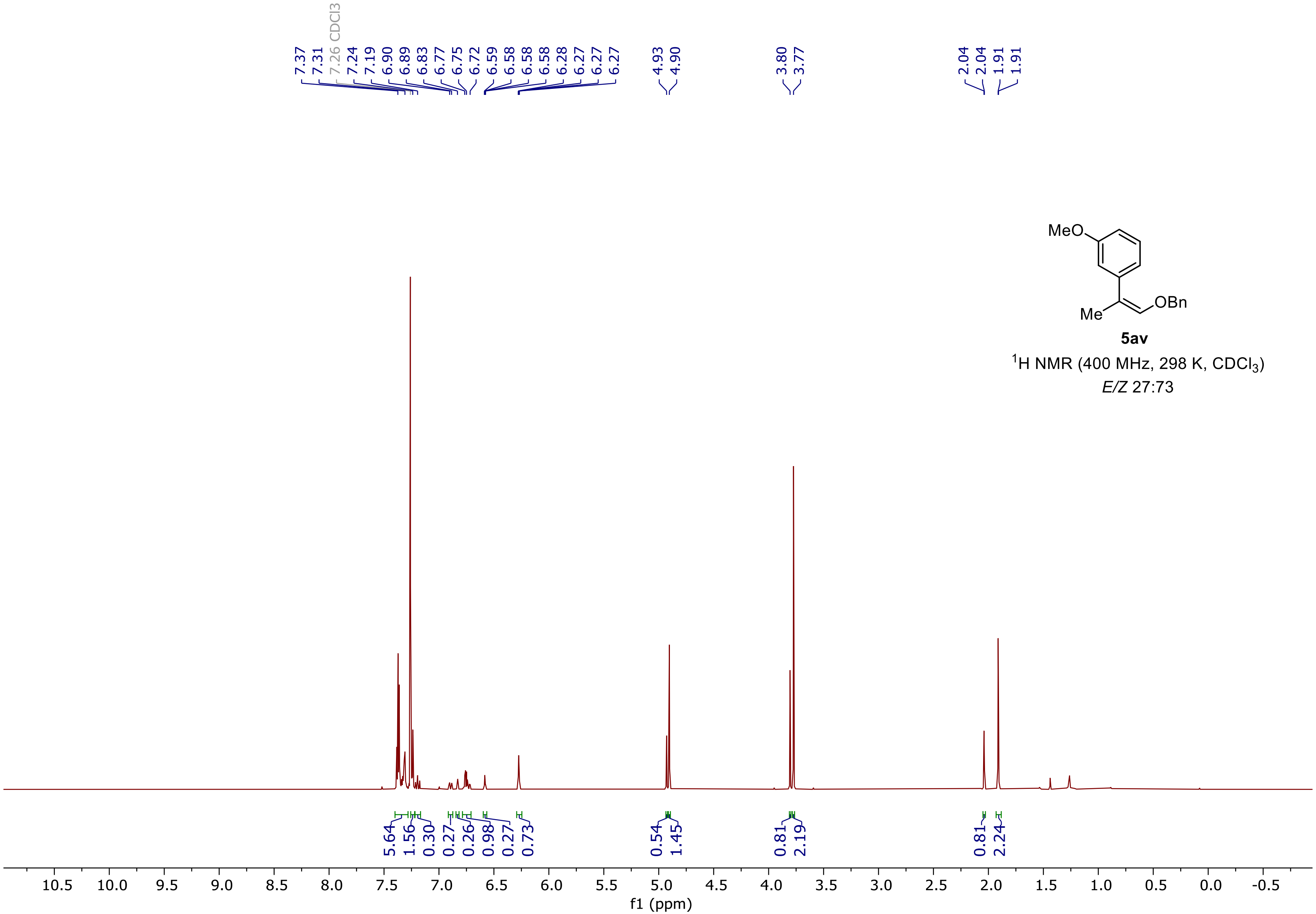

5av

^{1}H NMR (400 MHz, 298 K, $CDCl_3$)

E/Z 27:73

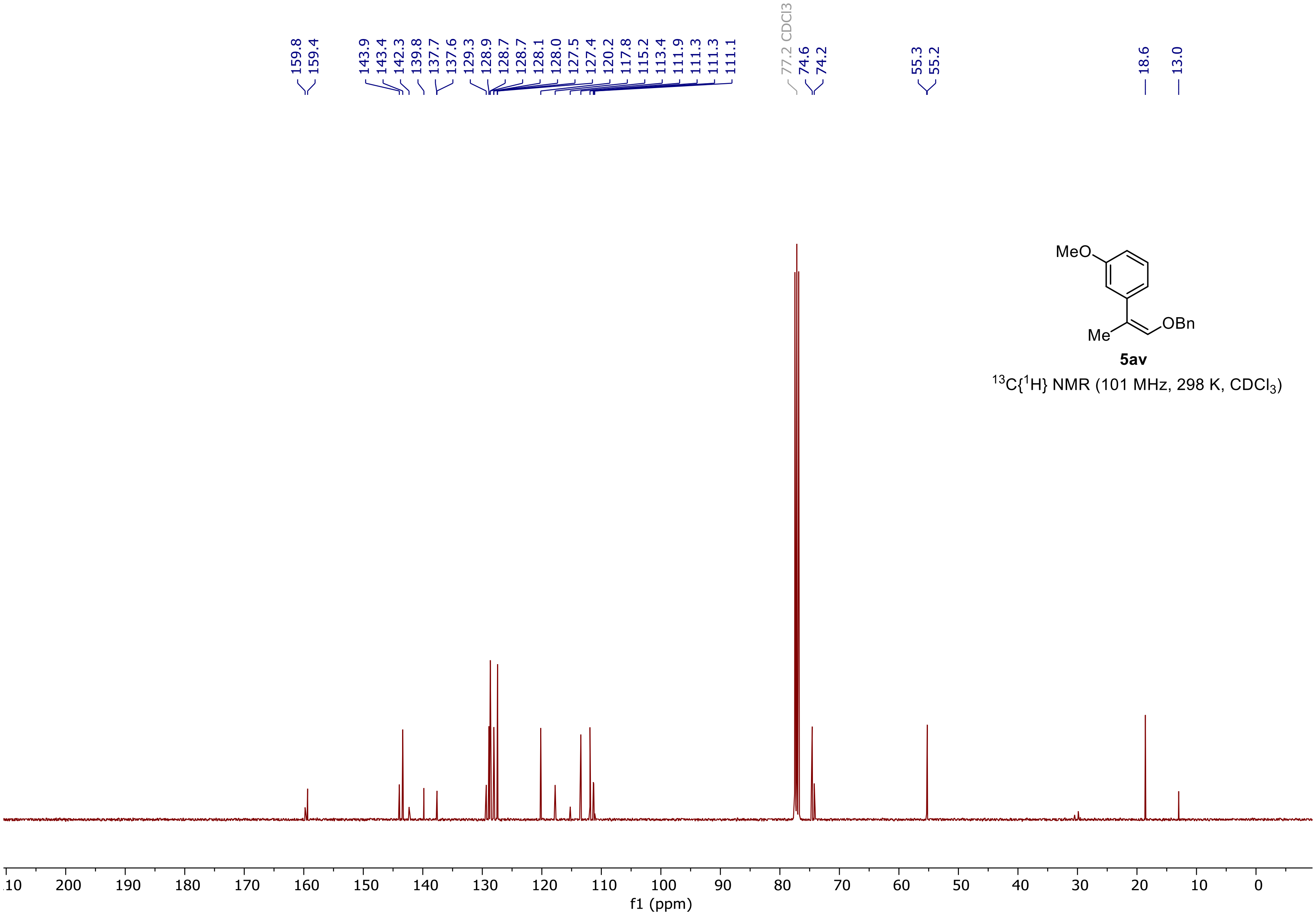

5av

$^{13}C\{^{1}H\}$ NMR (101 MHz, 298 K, $CDCl_3$)

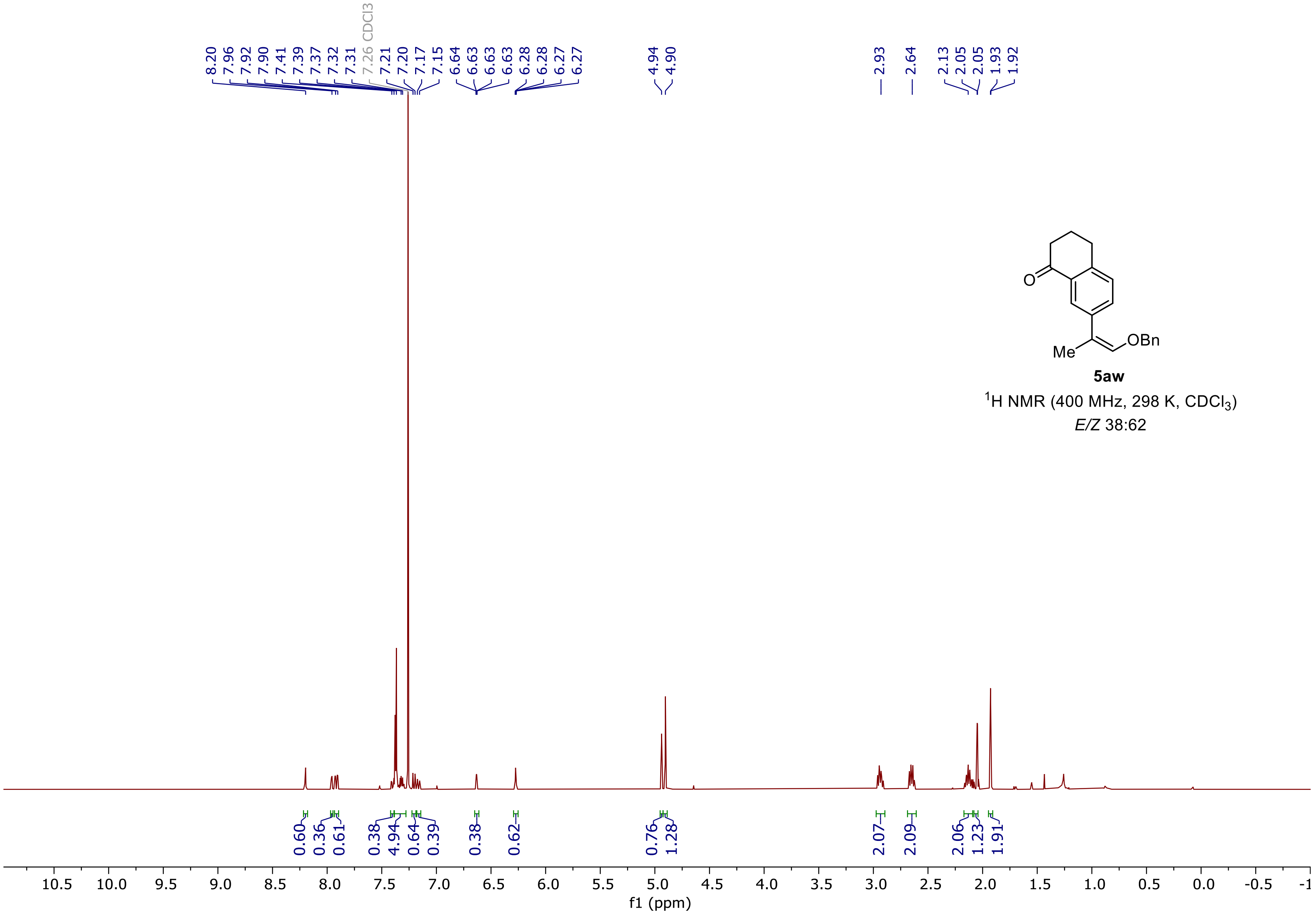

5aw

^{1}H NMR (400 MHz, 298 K, $CDCl_3$)

E/Z 38:62

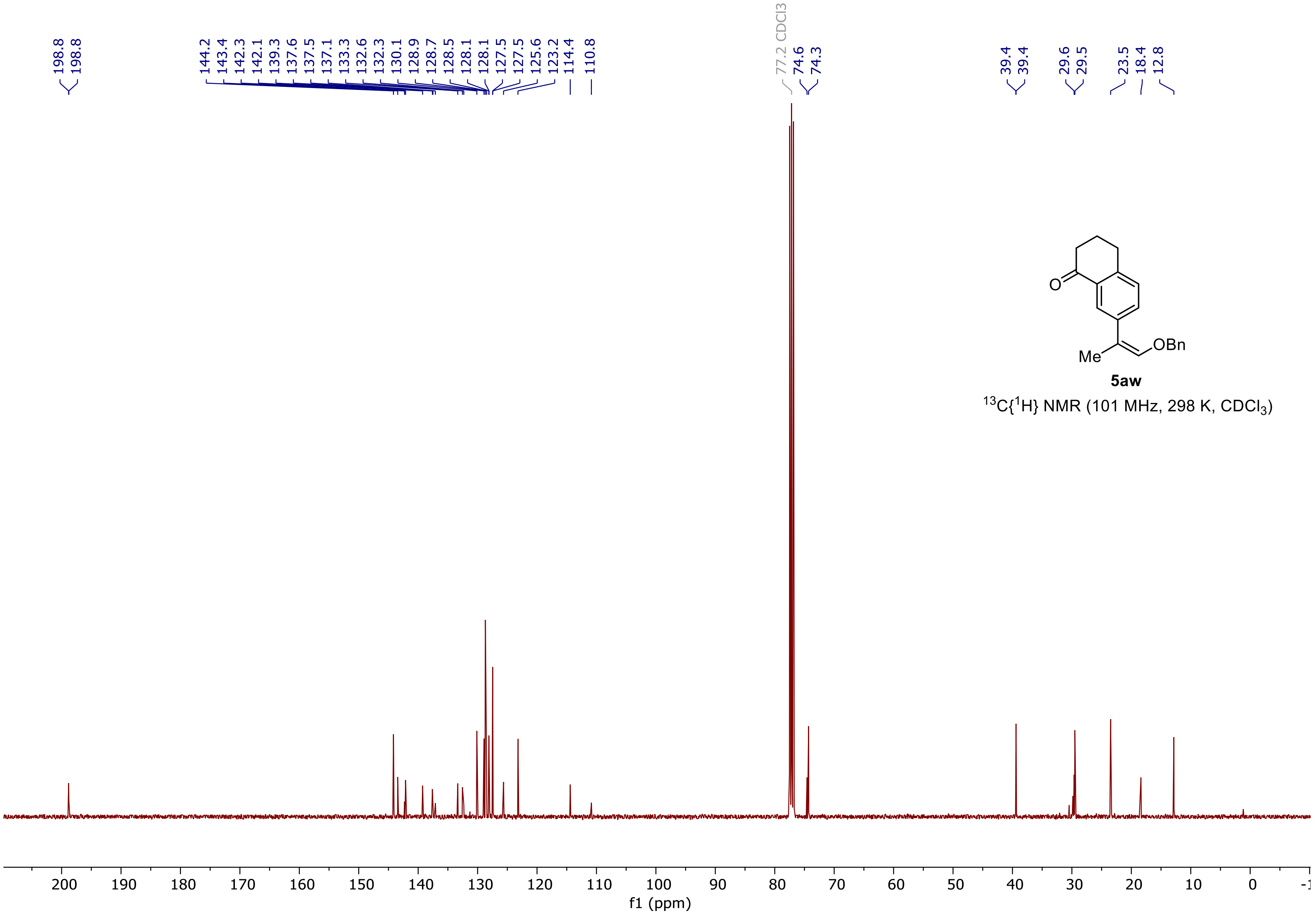

5aw

$^{13}C\{^{1}H\}$ NMR (101 MHz, 298 K, $CDCl_3$)

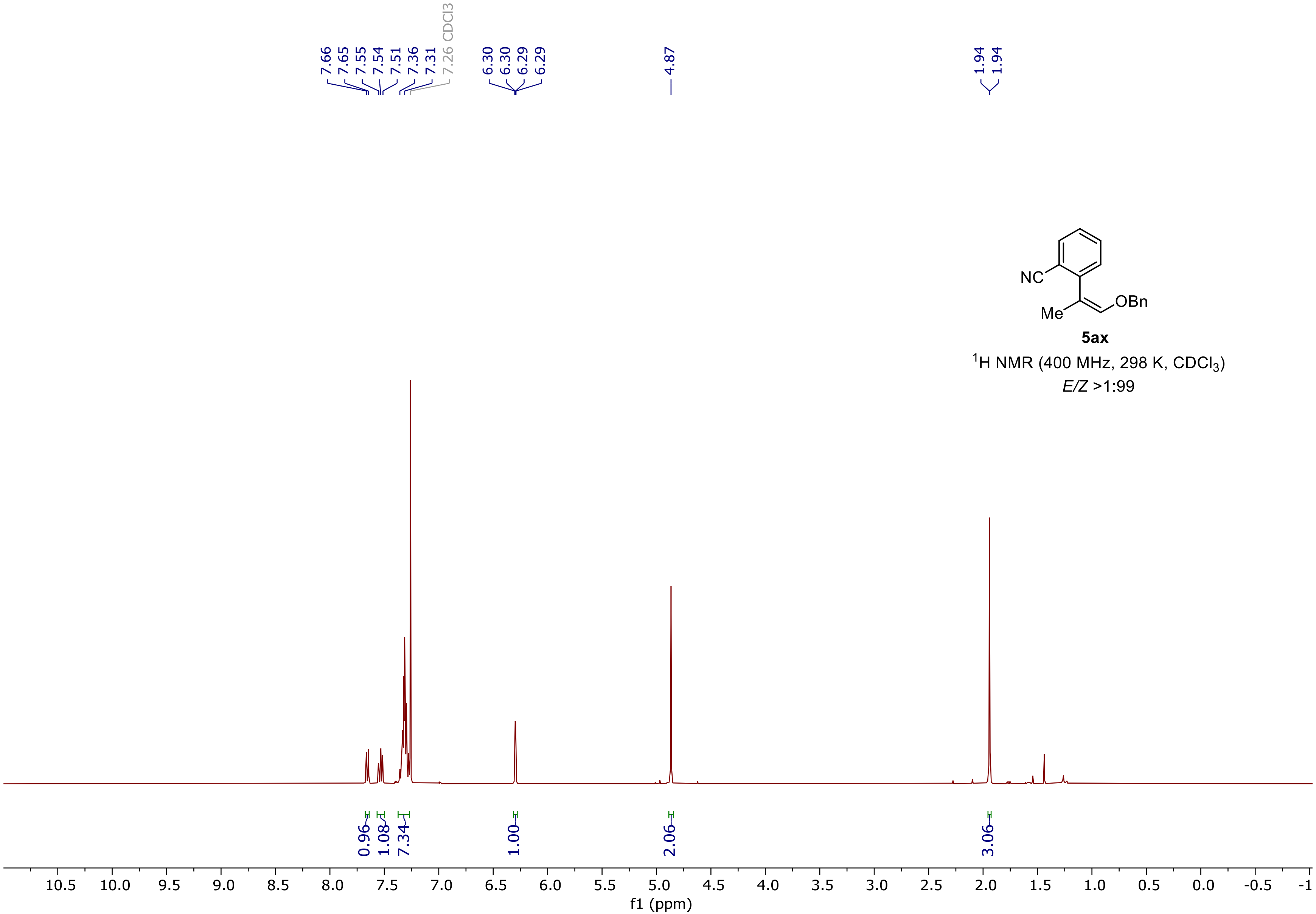

5ax

^{1}H NMR (400 MHz, 298 K, $CDCl_3$)

E/Z >1:99

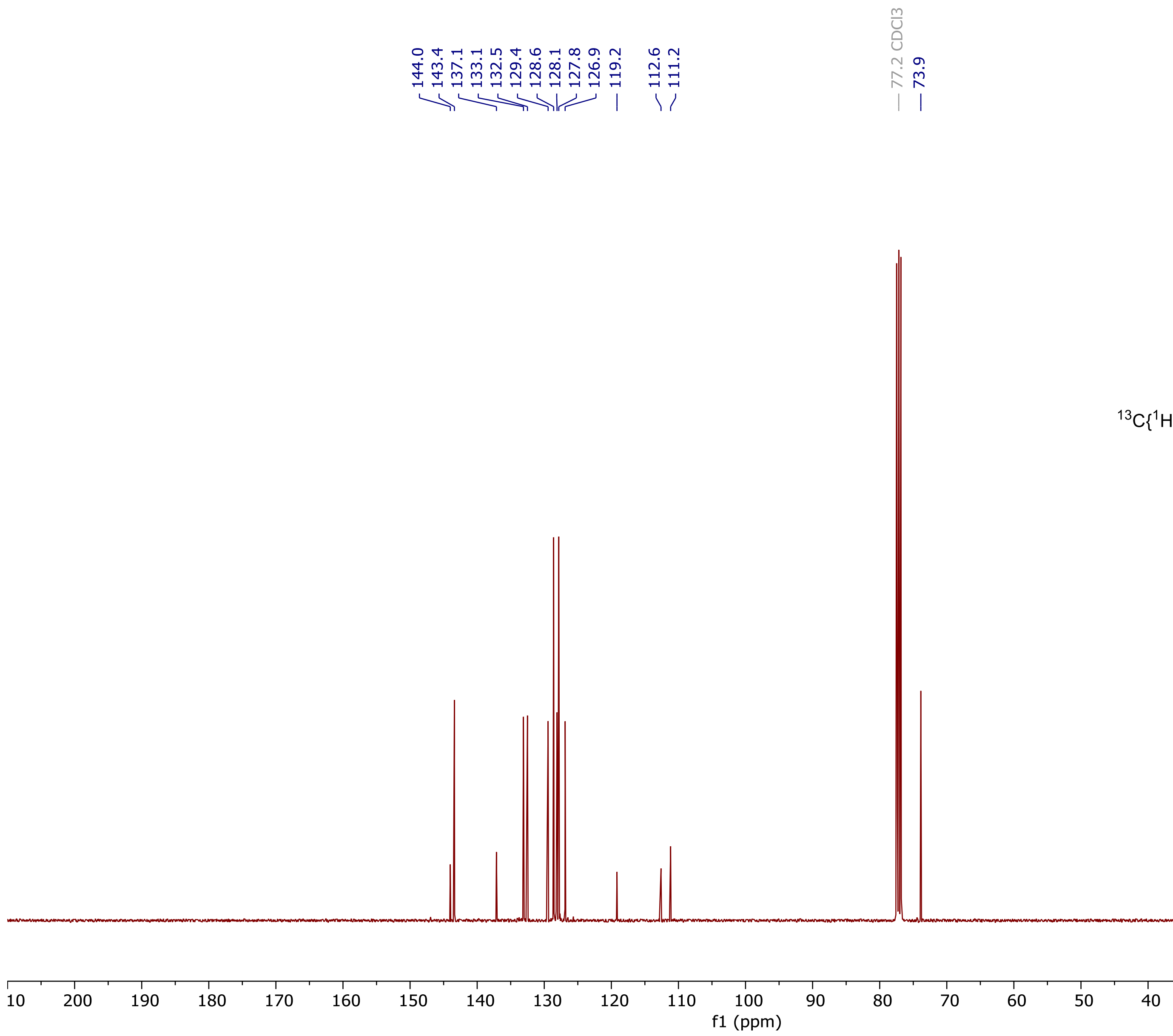

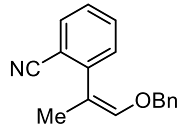

5ax

$^{13}C\{^{1}H\}$ NMR (101 MHz, 298 K, $CDCl_3$)

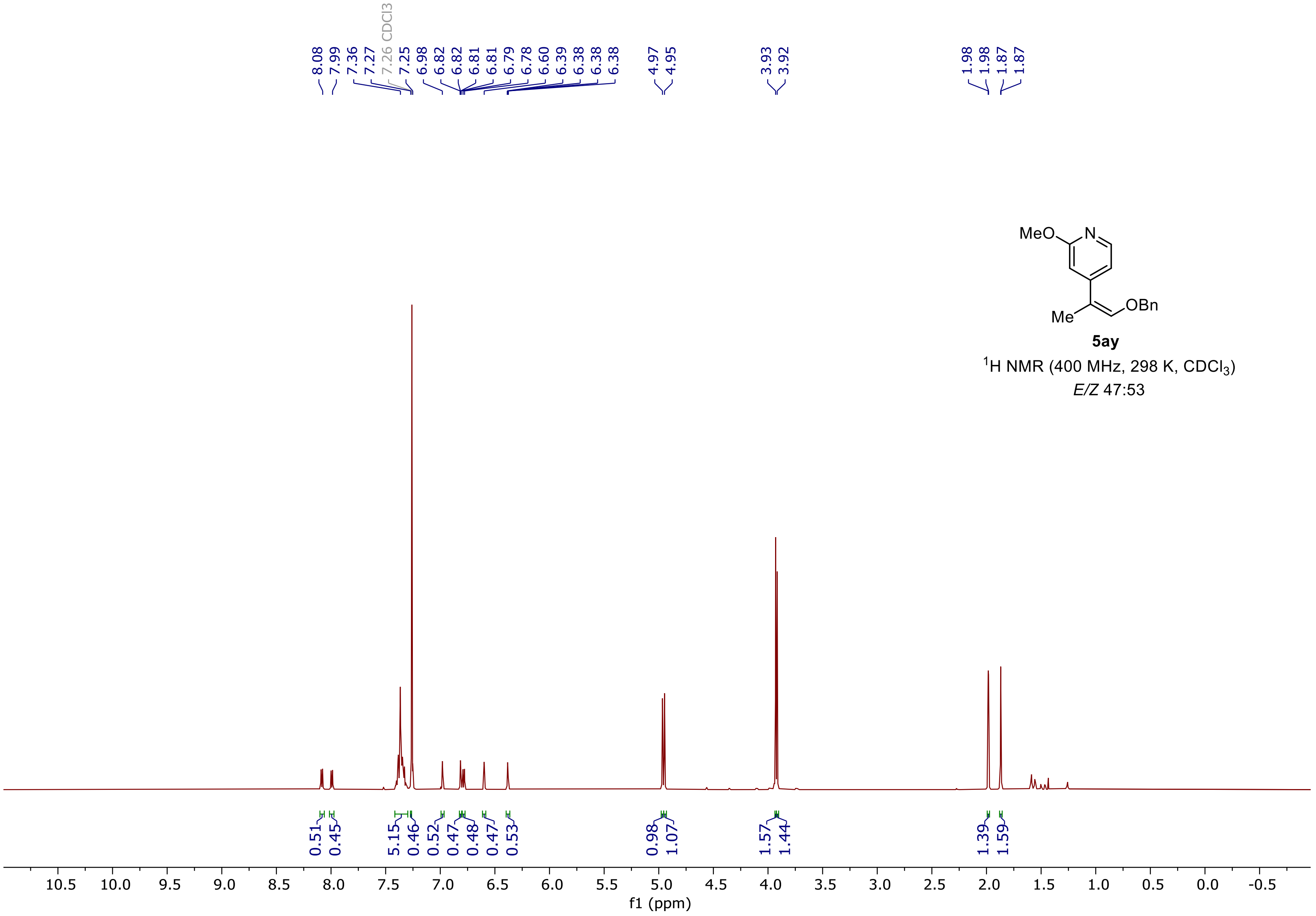

5ay

^{1}H NMR (400 MHz, 298 K, $CDCl_3$)

E/Z 47:53

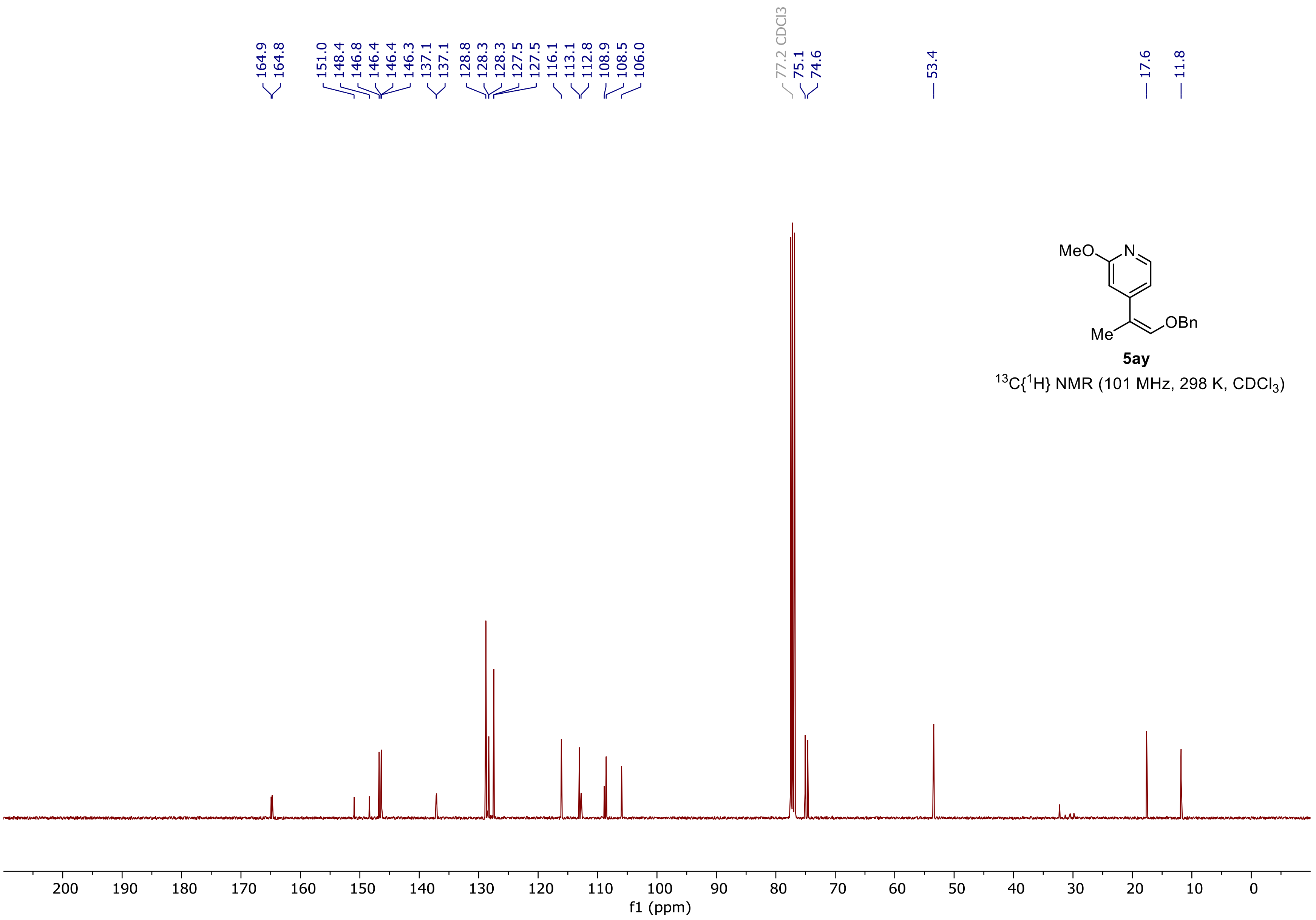
164.9
164.8
151.0
148.4
146.8
146.4
146.4
146.3
137.1
137.1
128.8
128.3
128.3
127.5
127.5
116.1
113.1
112.8
108.9
108.5
106.0
77.2 CDCl3
75.1
74.6
53.4
17.6
11.8
MeO
N
OBn
Me
5ay
13C{1H} NMR (101 MHz, 298 K, CDCl3)
200
190
180
170
160
150
140
130
120
110
100
90
80
70
60
50
40
30
20
10
0
f1 (ppm)

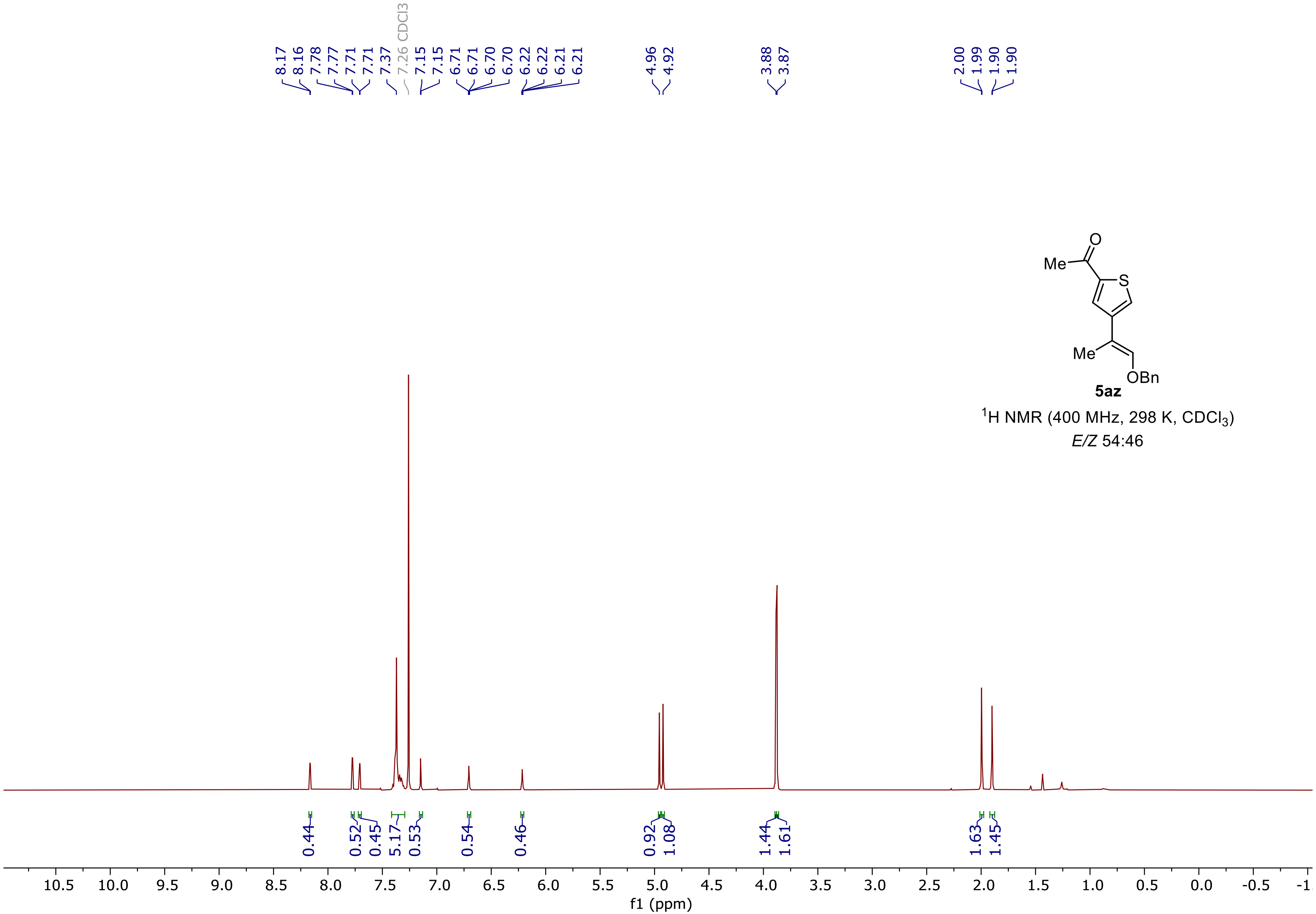

5az

^{1}H NMR (400 MHz, 298 K, $CDCl_3$)

E/Z 54:46

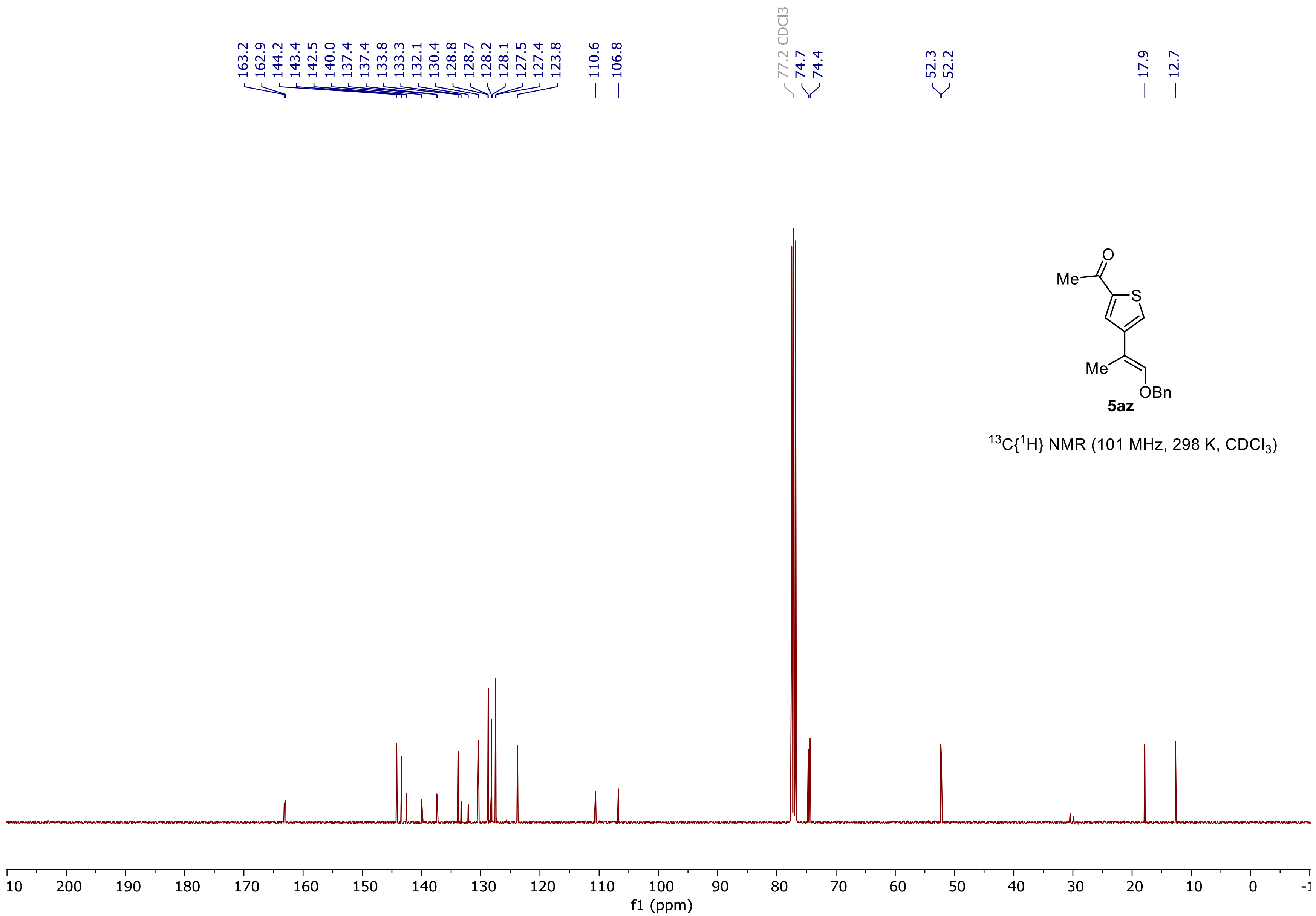
163.2
162.9
144.2
143.4
142.5
140.0
137.4
137.4
133.8
133.3
132.1
130.4
128.8
128.7
128.2
128.1
127.5
127.4
123.8
110.6
106.8
77.2 CDCl3
74.7
74.4
52.3
52.2
17.9
12.7
O
Me
S
Me
OBn
5az
$^{13}C\{^{1}H\}$ NMR (101 MHz, 298 K, $CDCl_3$)
f1 (ppm)

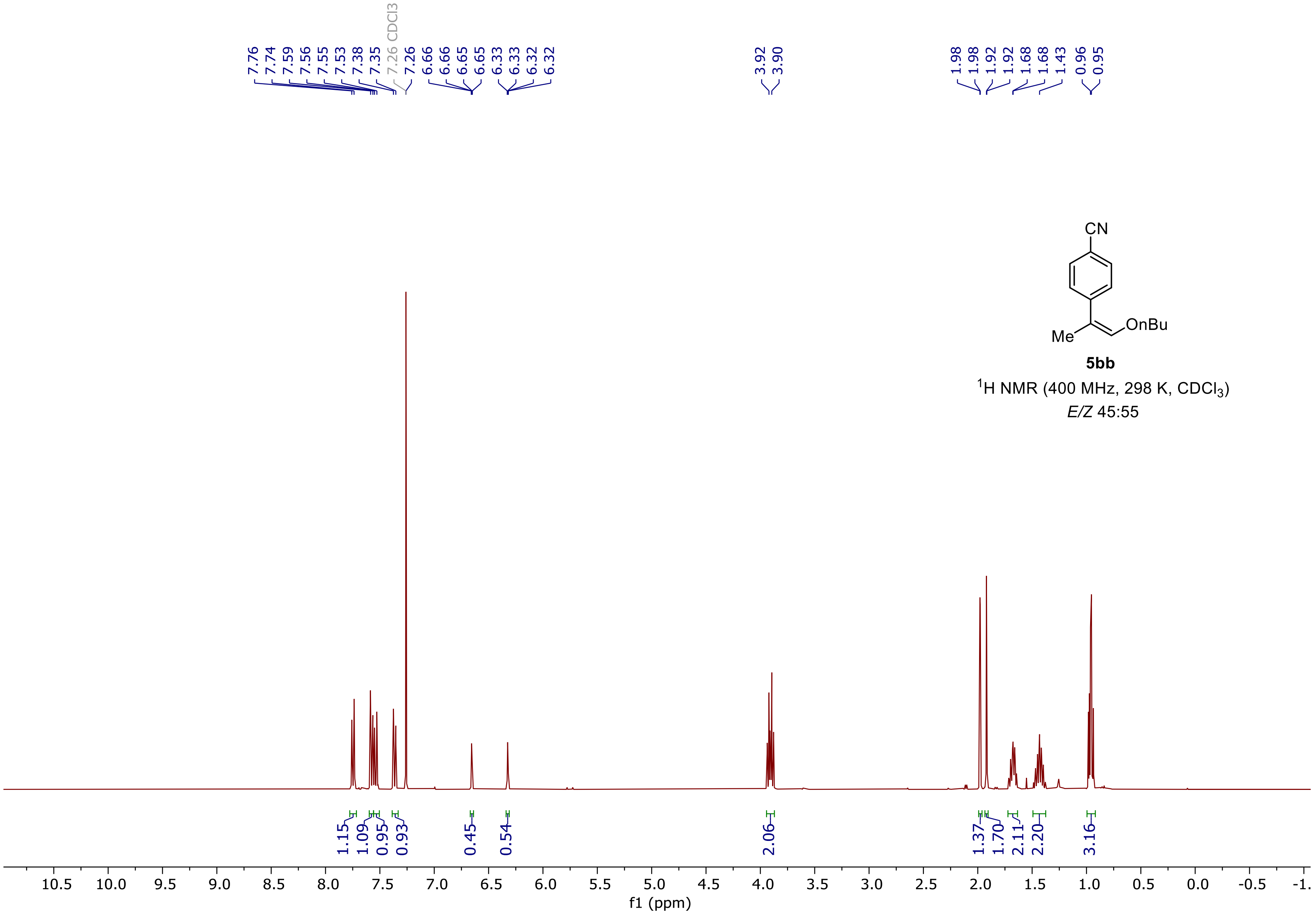

5bb

^{1}H NMR (400 MHz, 298 K, $CDCl_3$)

E/Z 45:55

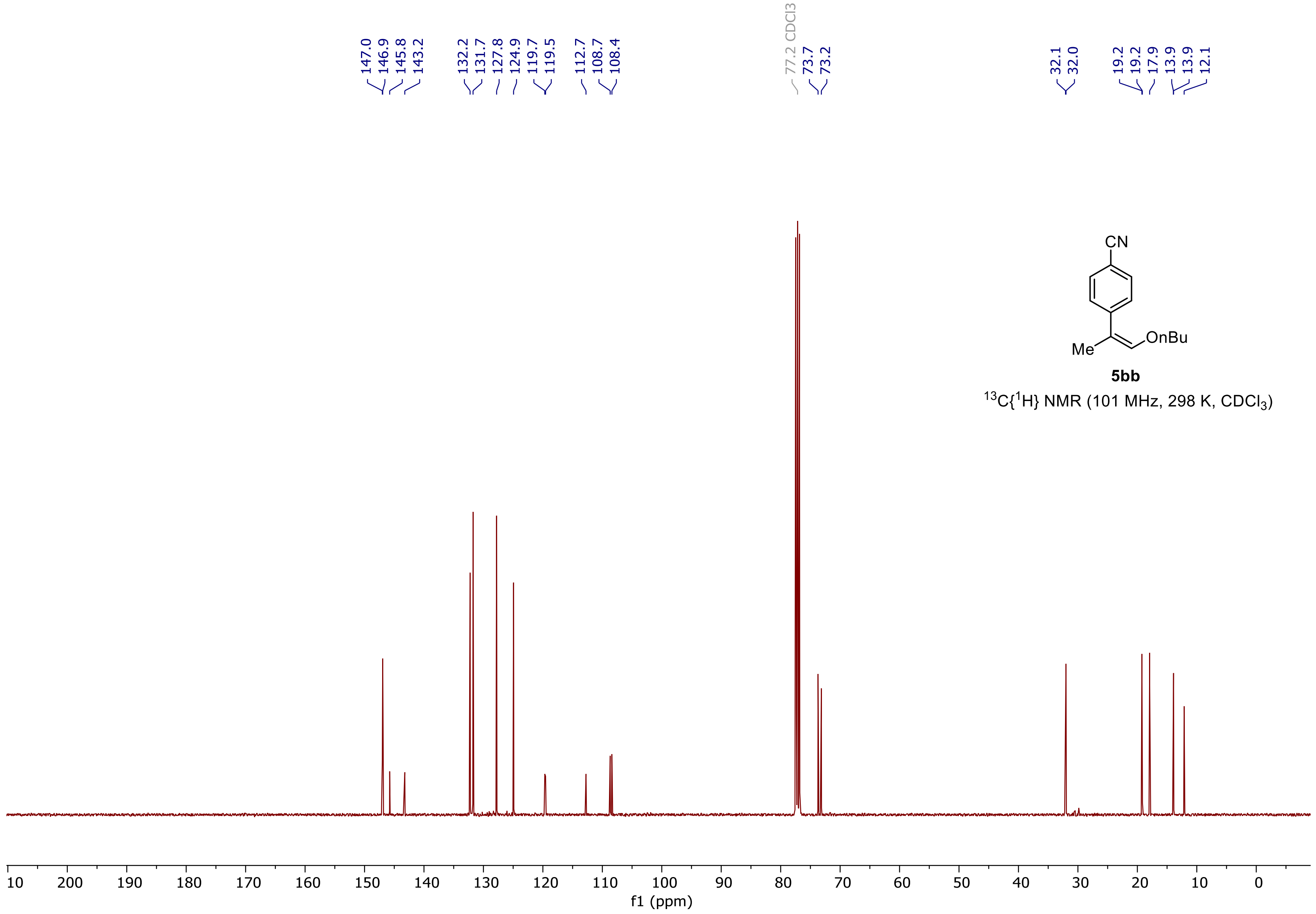

5bb

$^{13}C\{^{1}H\}$ NMR (101 MHz, 298 K, $CDCl_3$)

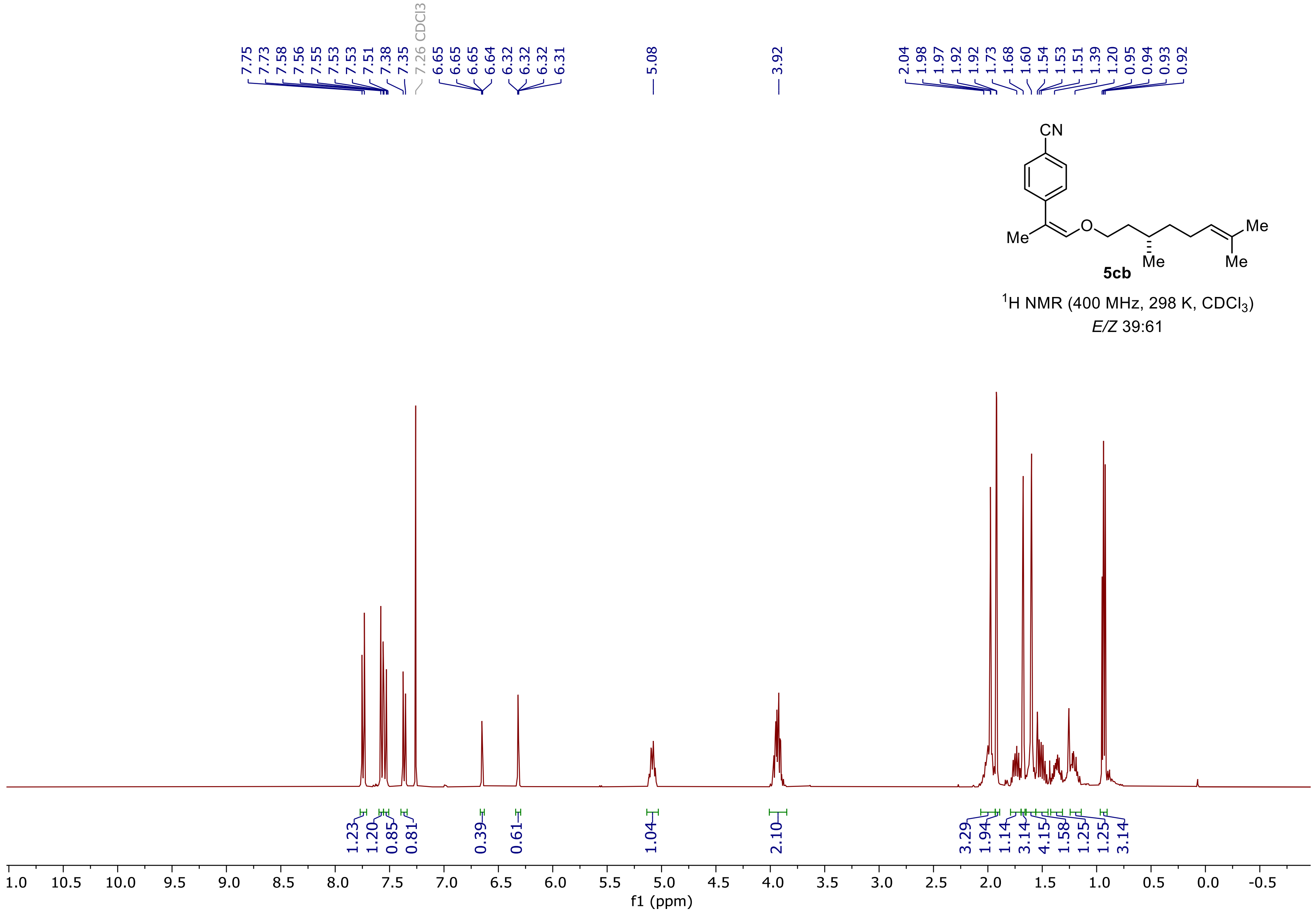
7.75 7.73 7.58 7.56 7.55 7.53 7.53 7.51 7.38 7.35 7.26 CDCl3 6.65 6.65 6.65 6.64 6.32 6.32 6.32 6.31
5.08
3.92
2.04 1.98 1.97 1.92 1.92 1.73 1.68 1.60 1.54 1.53 1.51 1.39 1.20 0.95 0.94 0.93 0.92
CN
O
Me
Me
Me
Me
5cb
^{1}H NMR (400 MHz, 298 K, $CDCl_3$)
E/Z 39:61
1.23 1.20 0.85 0.81 0.39 0.61 1.04 2.10 3.29 1.94 1.14 3.14 4.15 1.58 1.25 1.25 3.14
1.0 10.5 10.0 9.5 9.0 8.5 8.0 7.5 7.0 6.5 6.0 5.5 5.0 4.5 4.0 3.5 3.0 2.5 2.0 1.5 1.0 0.5 0.0 -0.5
f1 (ppm)

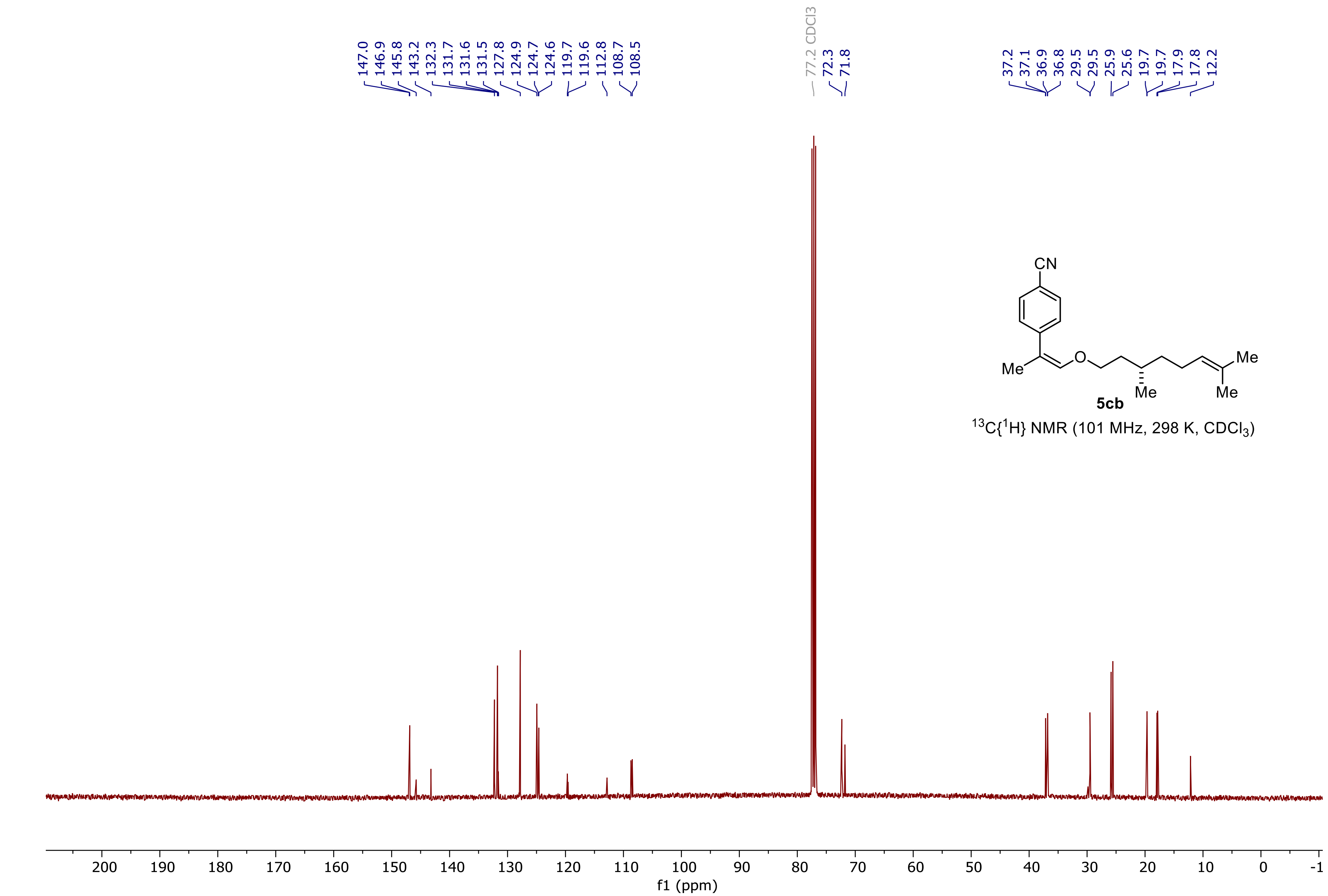
147.0
146.9
145.8
143.2
132.3
131.7
131.6
131.5
127.8
124.9
124.7
124.6
119.7
119.6
112.8
108.7
108.5
77.2 CDCl3
72.3
71.8
37.2
37.1
36.9
36.8
29.5
29.5
25.9
25.6
19.7
19.7
17.9
17.8
12.2
CN
O
Me
Me
Me
Me
5cb
$^{13}C\{^{1}H\}$ NMR (101 MHz, 298 K, $CDCl_3$)
200 190 180 170 160 150 140 130 120 110 100 90 80 70 60 50 40 30 20 10 0 -1
f1 (ppm)

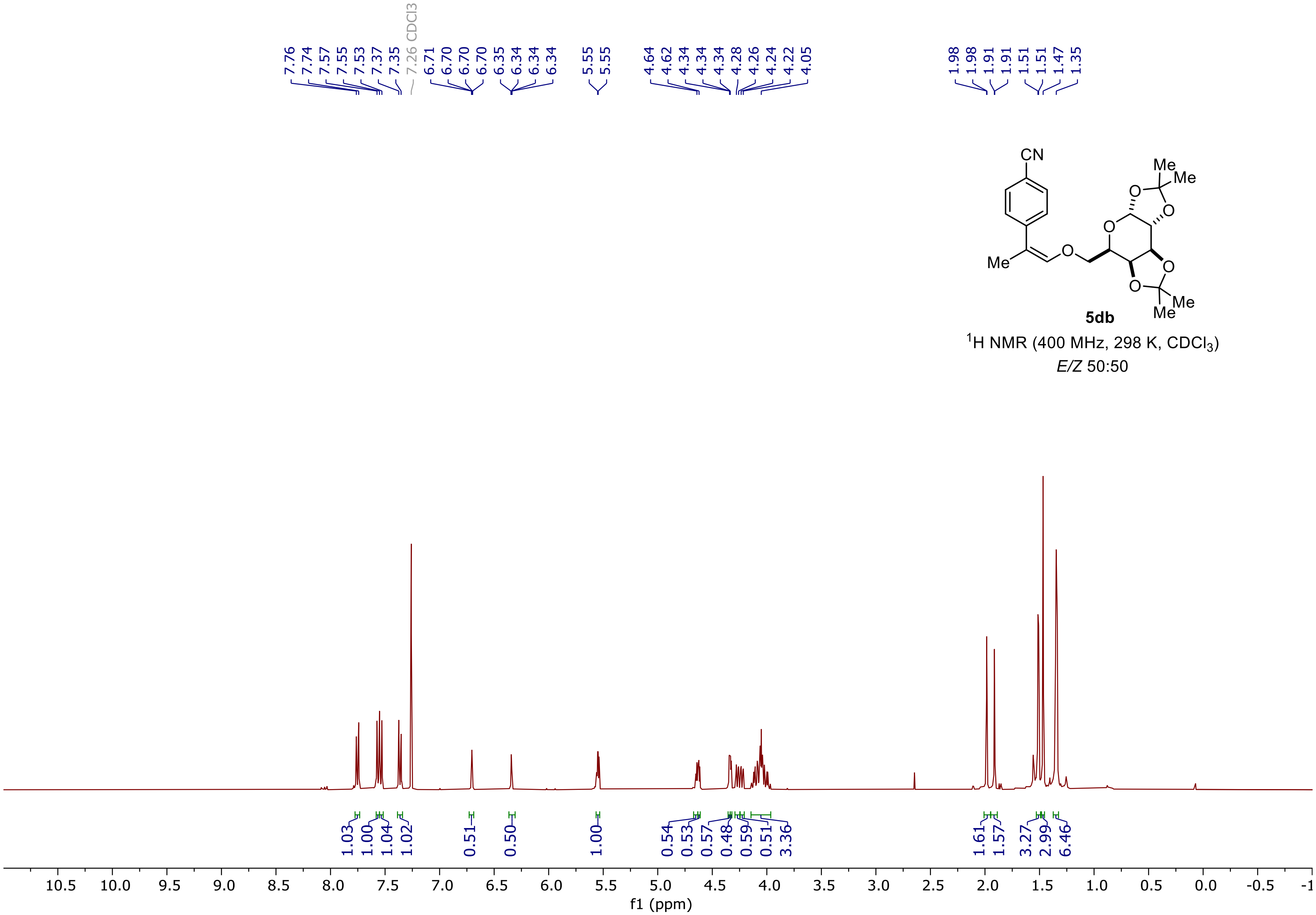

5db

^{1}H NMR (400 MHz, 298 K, $CDCl_3$)

E/Z 50:50

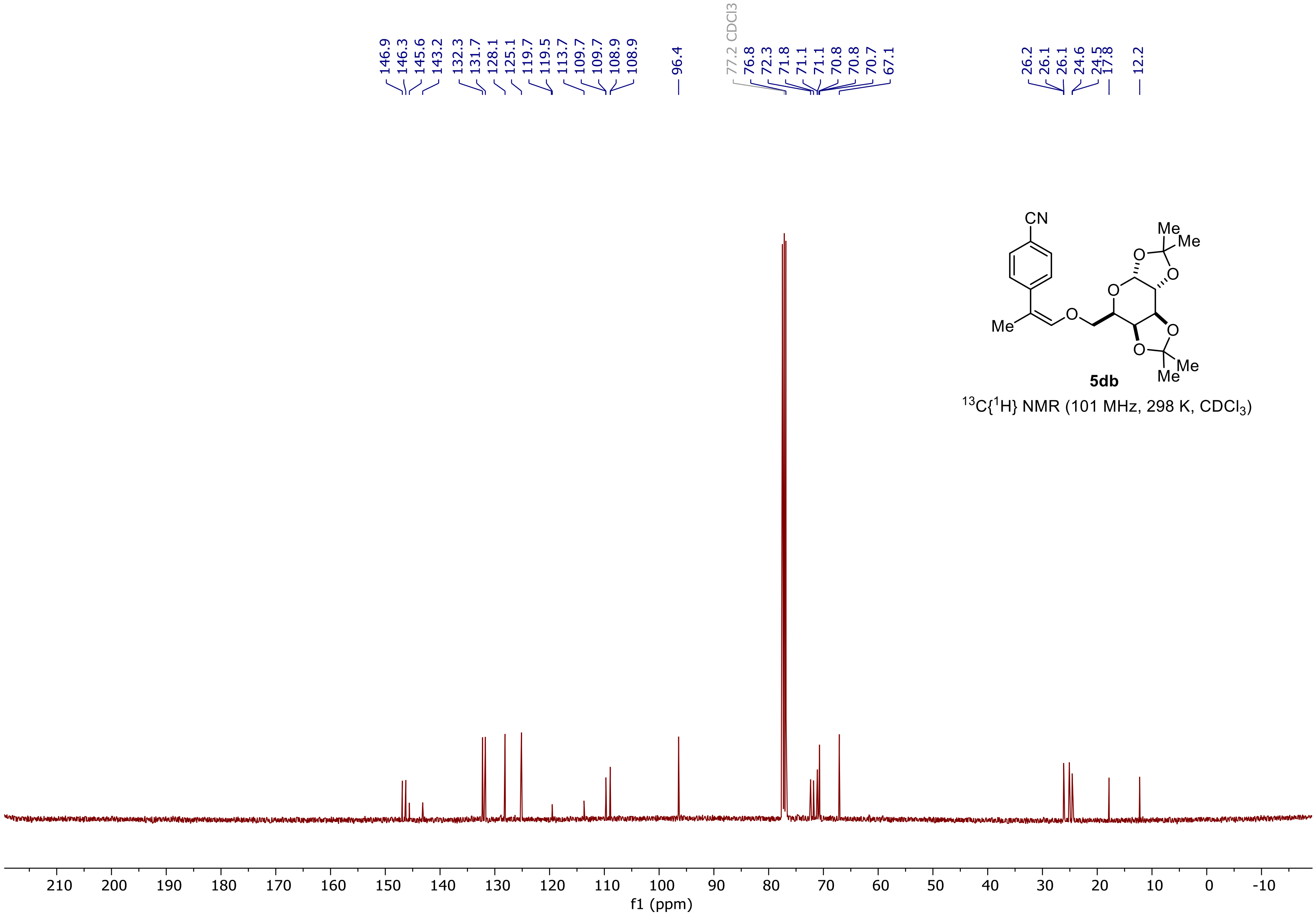

5db

$^{13}C\{^{1}H\}$ NMR (101 MHz, 298 K, $CDCl_3$)

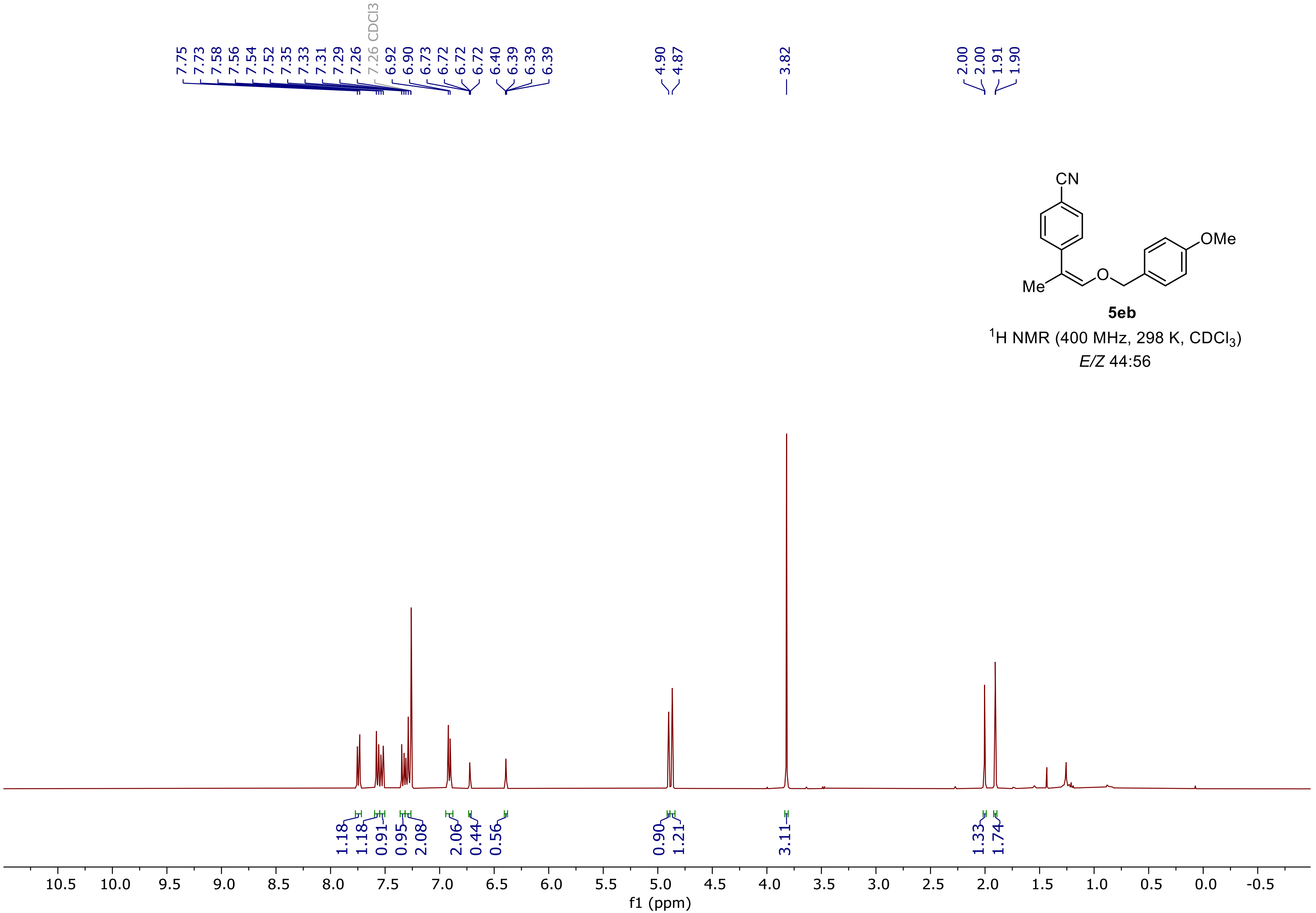

5eb

^{1}H NMR (400 MHz, 298 K, $CDCl_3$)

E/Z 44:56

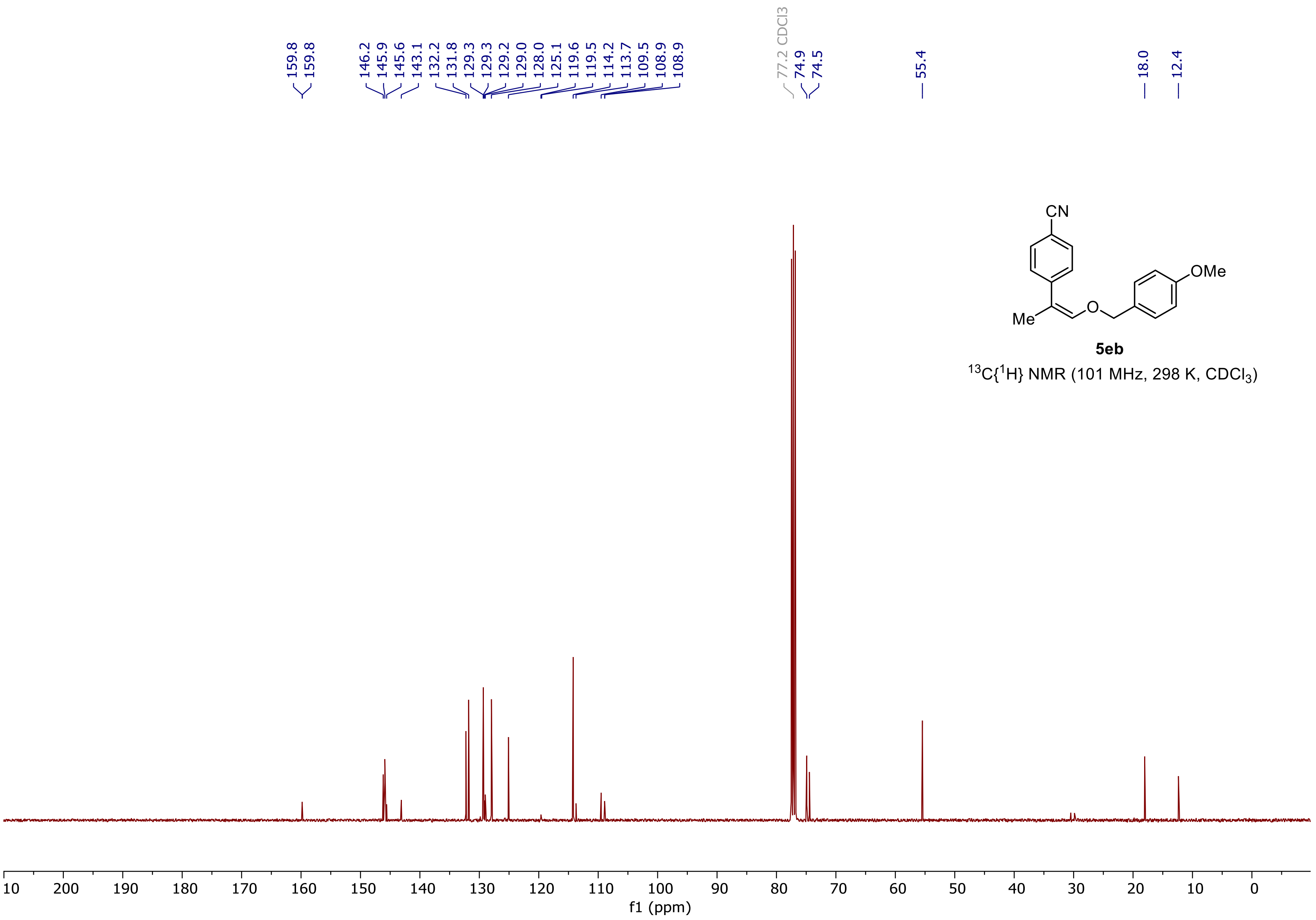

5eb

$^{13}C\{^1H\}$ NMR (101 MHz, 298 K, $CDCl_3$)

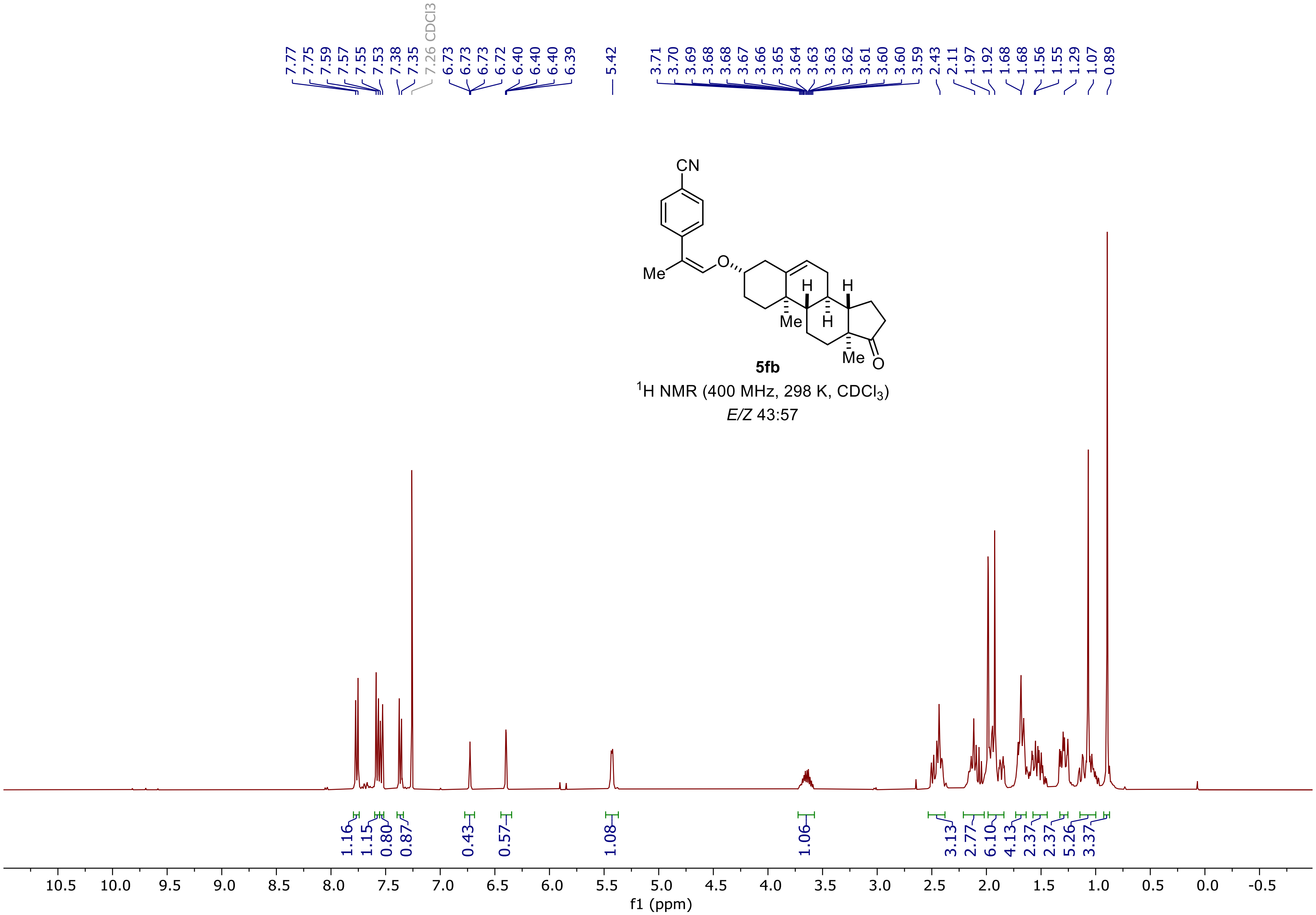
7.77
7.75
7.59
7.57
7.55
7.53
7.38
7.35
7.26 CDCl3
6.73
6.73
6.73
6.72
6.40
6.40
6.40
6.39
5.42
3.71
3.70
3.69
3.68
3.68
3.67
3.66
3.65
3.64
3.63
3.63
3.62
3.61
3.60
3.60
3.59
2.43
2.11
1.97
1.92
1.68
1.68
1.56
1.55
1.29
1.07
0.89
CN
Me
O
H
H
Me
H
Me
O
5fb
^{1}H NMR (400 MHz, 298 K, CDCl$_3$)
E/Z 43:57
1.16
1.15
0.80
0.87
0.43
0.57
1.08
1.06
3.13
2.77
6.10
4.13
2.37
2.37
5.26
3.37
10.5
10.0
9.5
9.0
8.5
8.0
7.5
7.0
6.5
6.0
5.5
5.0
4.5
4.0
3.5
3.0
2.5
2.0
1.5
1.0
0.5
0.0
-0.5
f1 (ppm)

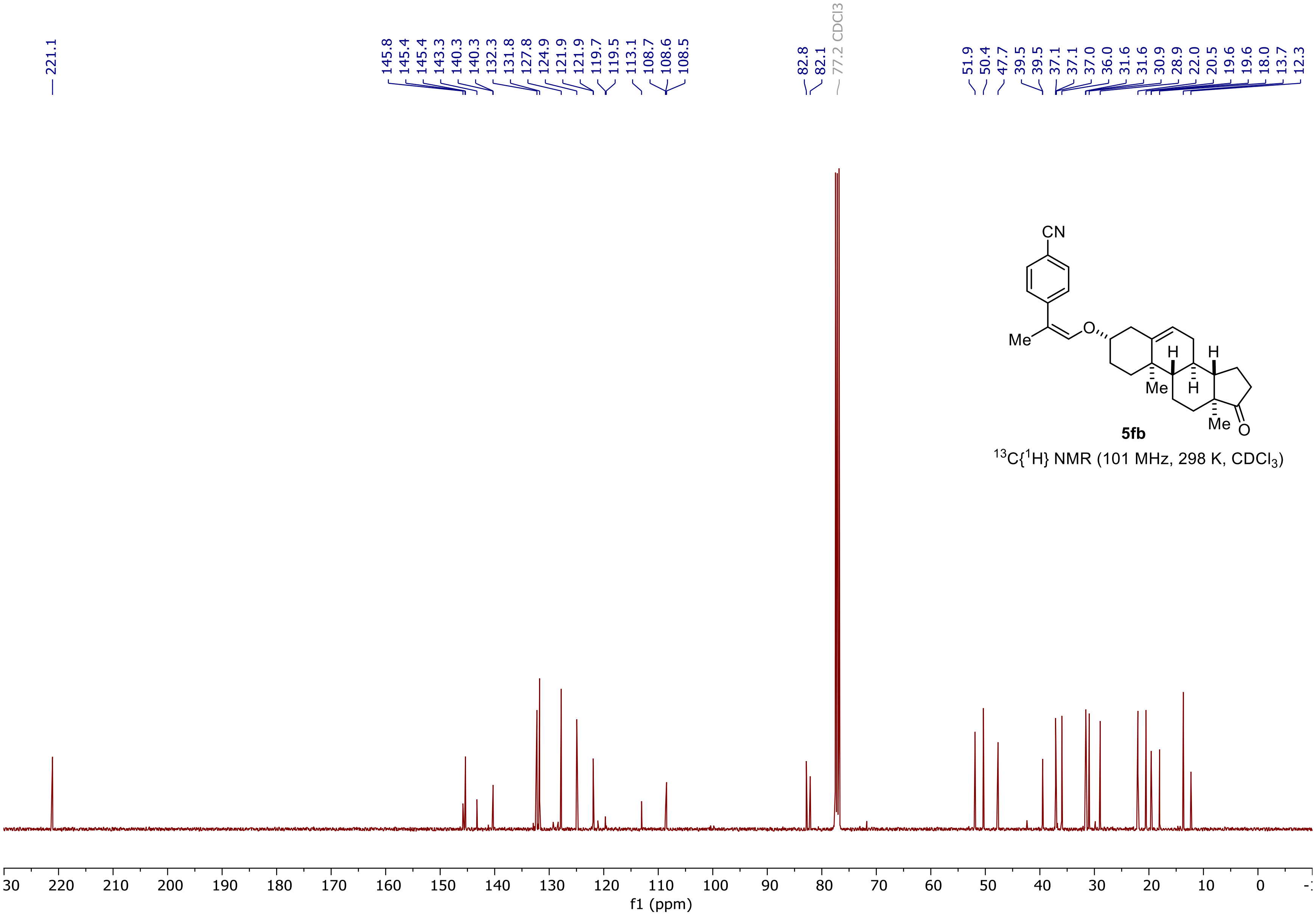

5fb

$^{13}C\{^{1}H\}$ NMR (101 MHz, 298 K, $CDCl_3$)

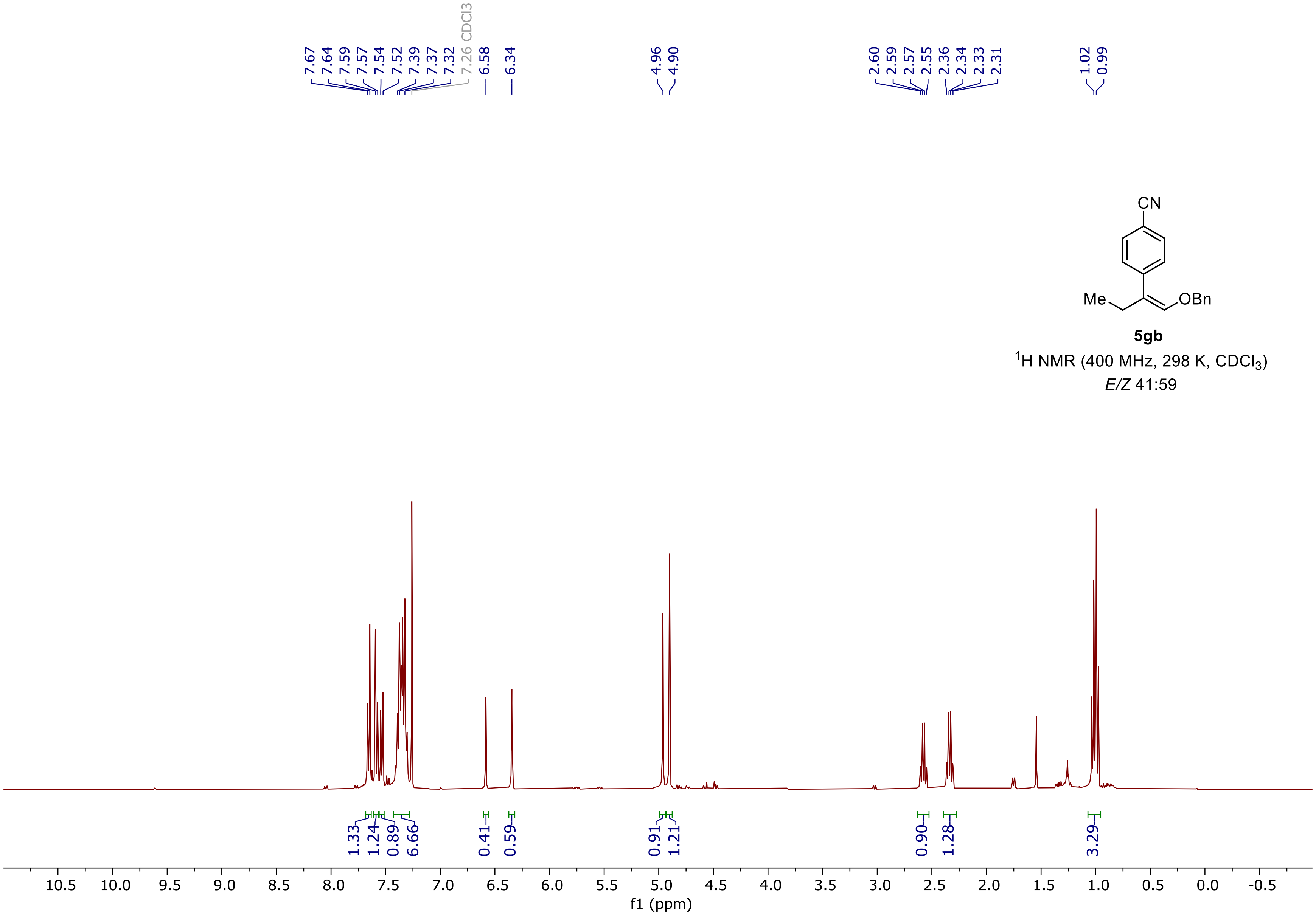

5gb

^{1}H NMR (400 MHz, 298 K, $CDCl_3$)

E/Z 41:59

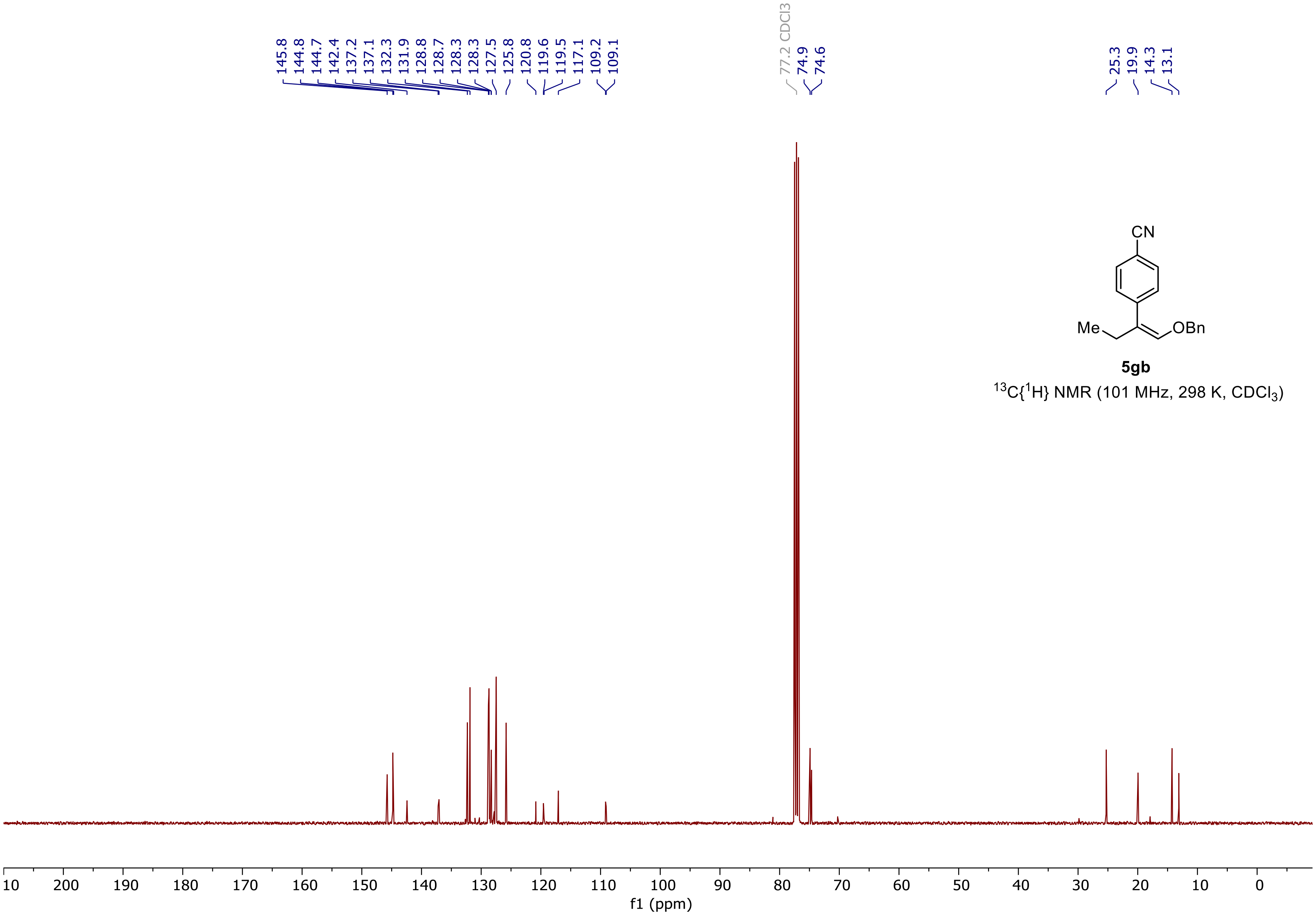

5gb

$^{13}C\{^{1}H\}$ NMR (101 MHz, 298 K, $CDCl_3$)

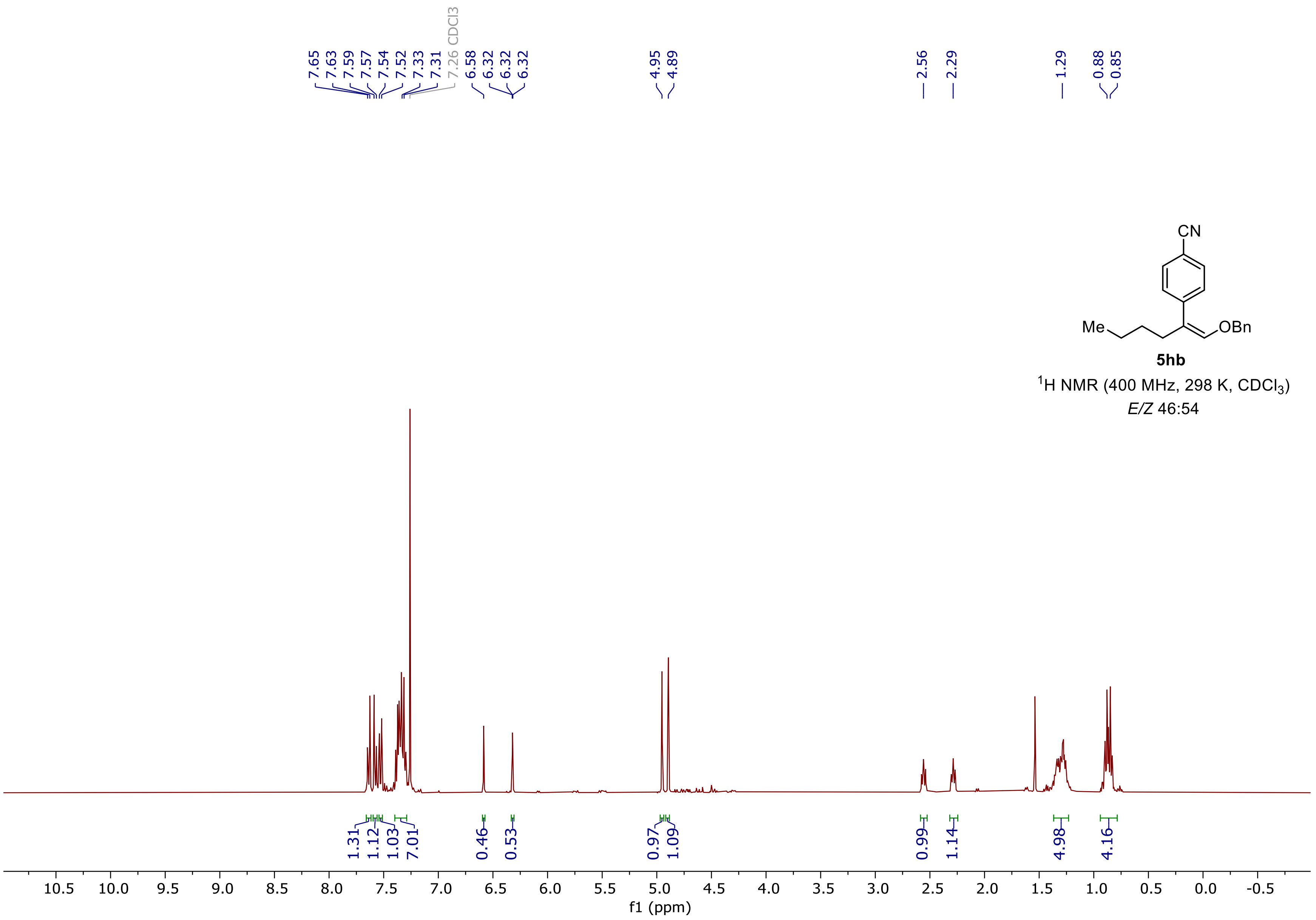
7.65
7.63
7.59
7.57
7.54
7.52
7.33
7.31
7.26 CDCl3
6.58
6.32
6.32
6.32
4.95
4.89
2.56
2.29
1.29
0.88
0.85
CN
Me
OBn
5hb
1H NMR (400 MHz, 298 K, CDCl3)
E/Z 46:54
1.31
1.12
1.03
7.01
0.46
0.53
0.97
1.09
0.99
1.14
4.98
4.16
10.5
10.0
9.5
9.0
8.5
8.0
7.5
7.0
6.5
6.0
5.5
5.0
4.5
4.0
3.5
3.0
2.5
2.0
1.5
1.0
0.5
0.0
-0.5
f1 (ppm)

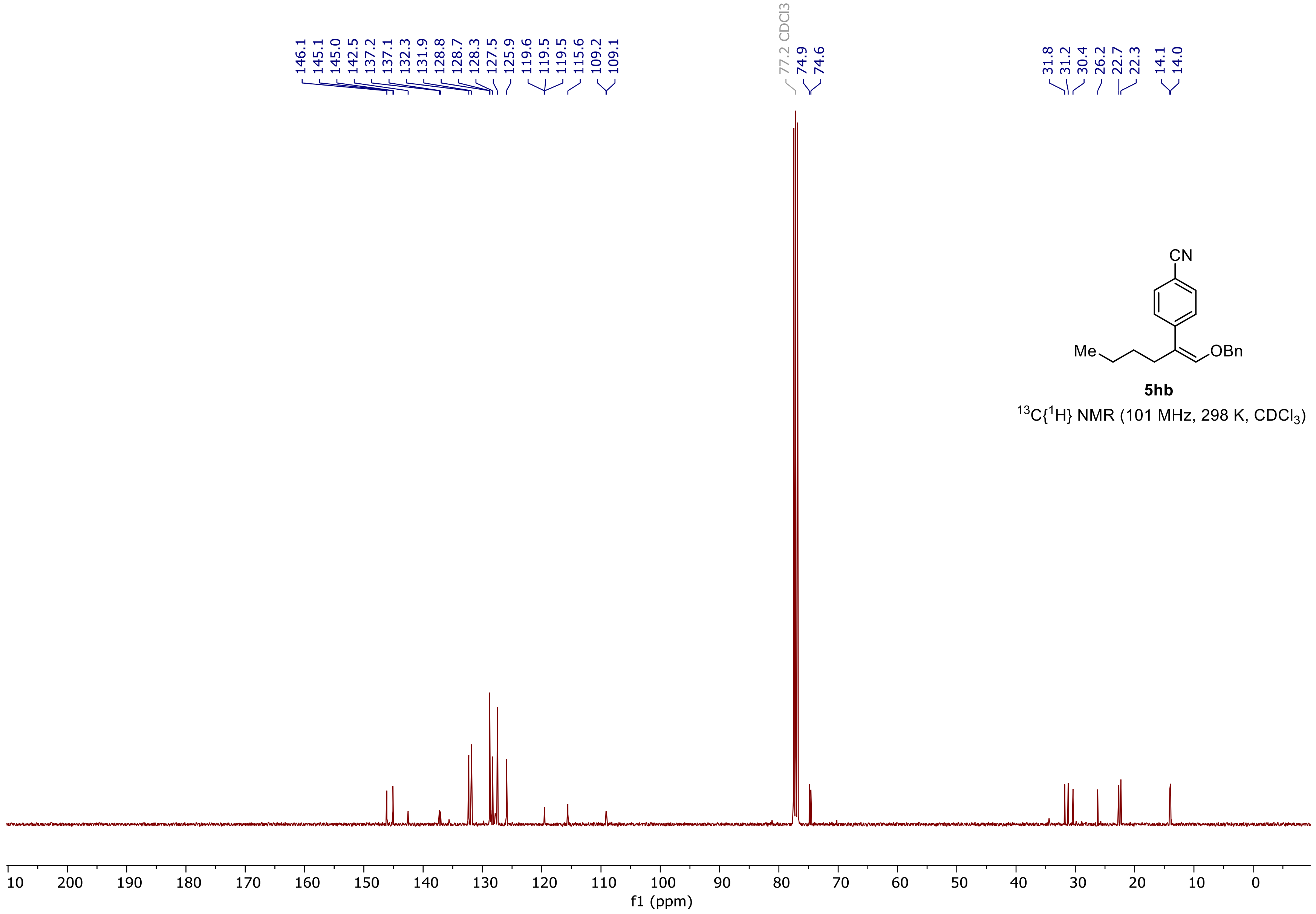

5hb

$^{13}C\{^{1}H\}$ NMR (101 MHz, 298 K, $CDCl_3$)
